# Heterogeneity in definitions of surgical site infection after cranial surgery limits the validity of research findings in neurosurgery: a systematic review

**DOI:** 10.1007/s10143-025-03218-5

**Published:** 2025-01-16

**Authors:** Nika Majidi, Avanthika Sivakumar, Antonia Vogt, Sruthi Ranganathan, Keyoumars Ashkan, Stephen J. Price, Keng Siang Lee

**Affiliations:** 1 Department of Neurosurgery, King’s College Hospital, London, UK; 2 School of Medicine and Biomedical Sciences, University of Oxford, Oxford, UK; 3 Department of Medicine, University of Cambridge, Cambridge, UK; 4 Division of Neurosurgery, Department of Clinical Neuroscience, University of Cambridge, Cambridge, UK; 5 Department of Basic and Clinical Neurosciences, Maurice Wohl Clinical Neuroscience Institute, Institute of Psychiatry, Psychology and Neuroscience (IoPPN), King’s College London, London, UK

**Keywords:** Craniotomy, Neurosurgery, Neuro-oncology, Skull base, Surgical site infection, Systematic review

## Abstract

Surgical site infections after cranial surgery (SSI-CRAN) are serious adverse events considering the vicinity of the wound to the central nervous system. Variability in outcome definitions can hinder the ability to produce reliable evidence. This systematic review aimed to investigate whether there is variation in SSI-CRAN definitions across studies and its impact on the identification of effective treatments for patients after cranial surgery. Systematic searches of Medline, Embase and Cochrane Central were undertaken. Studies that reported data about SSI-CRAN were included. Our final dataset consisted of 519 studies reporting SSI-CRAN. Subspecialties included were neuro-oncology (*n* = 103,19.8%), neurotrauma (*n* = 92,17.7%), paediatric neurosurgery (*n* = 56,10.8%), functional neurosurgery (*n* = 52,10.0%), neurovascular (*n* = 38,7.3%), skull base (*n* = 37,7.1%), cerebrospinal fluid dynamics (*n* = 11,2.1%), and general neurosurgical cases (*n* = 149,28.7%). A hundred and sixty-nine (32.6%) studies reported SSI-CRAN definitions. Twenty-six different indicators for SSI-CRAN were used, with a median of nine (range: one to 16) utilised per study. Only 21 (4.0%) studies considered the varying definitions of SSI-CRAN in their conclusions, indicating that a lack of consistent definitions across studies impacted the ability to draw clear conclusions. There are discrepancies in the definition of SSI-CRAN across the literature. This heterogeneity has hindered the ability to draw conclusions about the effects of interventions, with only 4.0% of authors accounting for this in their findings. It is important to be cautious when reviewing studies that report SSI-CRAN outcomes without acknowledging this heterogeneity, as the validity of their reported evidence may be limited. The implementation of a new core indicator set could support uniform reporting of indicators along with regulation of reported SSI-CRAN outcomes, thereby facilitating more effective evidence synthesis.

**Supplementary Information:**

The online version contains supplementary material available at 10.1007/s10143-025-03218-5.

## Introduction

Surgical site infections (SSIs) after cranial surgery (SSI-CRAN) are serious adverse events considering the vicinity of the wound to the central nervous system along with the risk of surgical meningitis [1]. SSI-CRAN are associated with significant patient morbidity and mortality [2], extended length of stays, greater hospital costs and delays to vital adjuvant therapy [3]. Given these devastating sequelae of SSI-CRAN, identifying modifiable risk factors and interventions is highly desirable. Systematic reviews synthesising data from primary studies are valuable for determining interventions that minimise the risk of adverse patient outcomes with SSI-CRAN [4]. 

Numerous risk factors have been identified in previous studies [5, 6]; with varying estimates of the effects of the same intervention [7–10]. This is likely attributable to significant methodological heterogeneity in the current evidence base, including differences in inclusion criteria and SSI definitions [11].

Variability in defining a single outcome is not uncommon and has been observed across multiple medical fields [12–20]. In 2009, the most cited Cochrane reviews highlighted that variability in definitions and outcome measurements hinders the ability of systematic reviews to effectively analyse evidence from all relevant studies [21].

Importantly, in the context of SSI-CRAN, the extent of heterogeneity in definitions used in neurosurgical intervention studies, and the degree to which this variability has been acknowledged in assessing intervention effectiveness, remains unclear. Consequently, the aim of this systematic review was to identify how SSI-CRAN is defined across the literature reporting outcomes of interventions for patients after cranial surgery. We also sought to assess whether authors accounted for heterogeneity in the definition of SSI-CRAN, and its impact when formulating conclusions about treatment effectiveness [22–24].

## Methods

The review was conducted according to the Preferred Reporting Items for Systematic Reviews and Meta-Analyses (PRISMA) guidelines [25]. The protocol was registered on the PROSPERO international prospective register of systematic reviews (registration number CRD42024513890).

### Search strategy

Searches of the following three electronic databases were undertaken: Ovid Medline, Ovid Embase, and Cochrane Central Register of Controlled Trials (CENTRAL). Searches were performed in each database from its inception until 24th February 2024. The concepts of “surgical site infection”, and “neurosurgery/craniotomy” were used in addition to synonyms and related terms. The full search strategy used for the databases is presented in Supplementary Table 1.

In addition, the reference lists of included studies were scrutinised to identify relevant studies that may have been inadvertently overlooked in the search strategy [26].

### Study selection

All titles, abstracts, and subsequently full texts were screened against the pre-defined eligibility criteria by three reviewers (NM, AV and AS). A full list of inclusion and exclusion criteria can be found in Supplementary Table 2. Disagreements were addressed by discussion, and where agreement could not be reached, the senior reviewer assisted with decision making (KSL). Agreement among the reviewers on study inclusion were evaluated with Cohen’s kappa statistic [22]. 

### Data extraction

A pro-forma was produced and piloted to ensure standardised data extraction. Extracted data to characterise included studies were: citation, first author, year of publication, DOI, country, pathology and intervention investigated.

For each study, data were extracted regarding study methods and indicators used to define SSI-CRAN:

i) Whether SSI-CRAN was defined in the study methods or results, i.e. it reported a diagnostic tool (consensus statement) or the indicators used for diagnosis.
ii) A verbatim report of each indicator used to define SSI-CRAN. We accepted author-defined SSI-CRAN, whether the indicators were those typically used to define SSI-CRAN or not. Each indicator was categorised under a label to allow summary and comparison of data. The number of indicators used to define SSI-CRAN were noted for each study.
iii) Whether the same indicators were used to define SSI-CRAN across studies.
iv) Whether numerical values were reported for indicators to determine presence of surgical site infection.
v) Whether a method for combining data from several indicators to determine presence of SSI-CRAN was specified (e.g. a count of the number of indicators present or a weighted scoring system).
i) Conclusions from included studies regarding intervention effectiveness, and whether variation in SSI-CRAN definition is accounted for by the primary authors in relation to effectiveness.

### Categorising indicators

Examination of the verbatim data from (ii) to describe the SSI-CRAN indicators used in each study demonstrated that for some indicators of SSI-CRAN, terminology used to describe the same indicator varied across studies. To enable a count of the indicators used, and identify common indicators used across studies, a process was undertaken to group such indicators under a consistent label. For example, if different studies had described an indicator as ‘wound microscopy from swab’, ‘bacteria in wound identified using swab’, ‘swab of wound pus’, these indicators were assigned the same label of positive ‘wound swab culture’. A small number of studies defined SSI-CRAN using an indicator that represented a group of signs or symptoms, for example ‘clinical signs’ or ‘clinical symptoms of infection’. These indicators were highlighted during the extraction phase using their verbatim terminology and were not included in the table of indicators since it is not known what signs and symptoms the authors referred to.

Two reviewers (NM and AS) independently extracted data to ensure accuracy, completeness, and reliability of data extraction. Discrepancies or disagreements about extracted material were addressed and resolved by senior reviewer (KSL).

### Data analysis and reporting

A narrative synthesis of data, with descriptive analyses where appropriate, was undertaken. The quality of included studies was assessed using the Joanna Briggs Institute (JBI) checklist for non-randomised experimental studies.

## Results

### Study selection

**Fig. 1:**
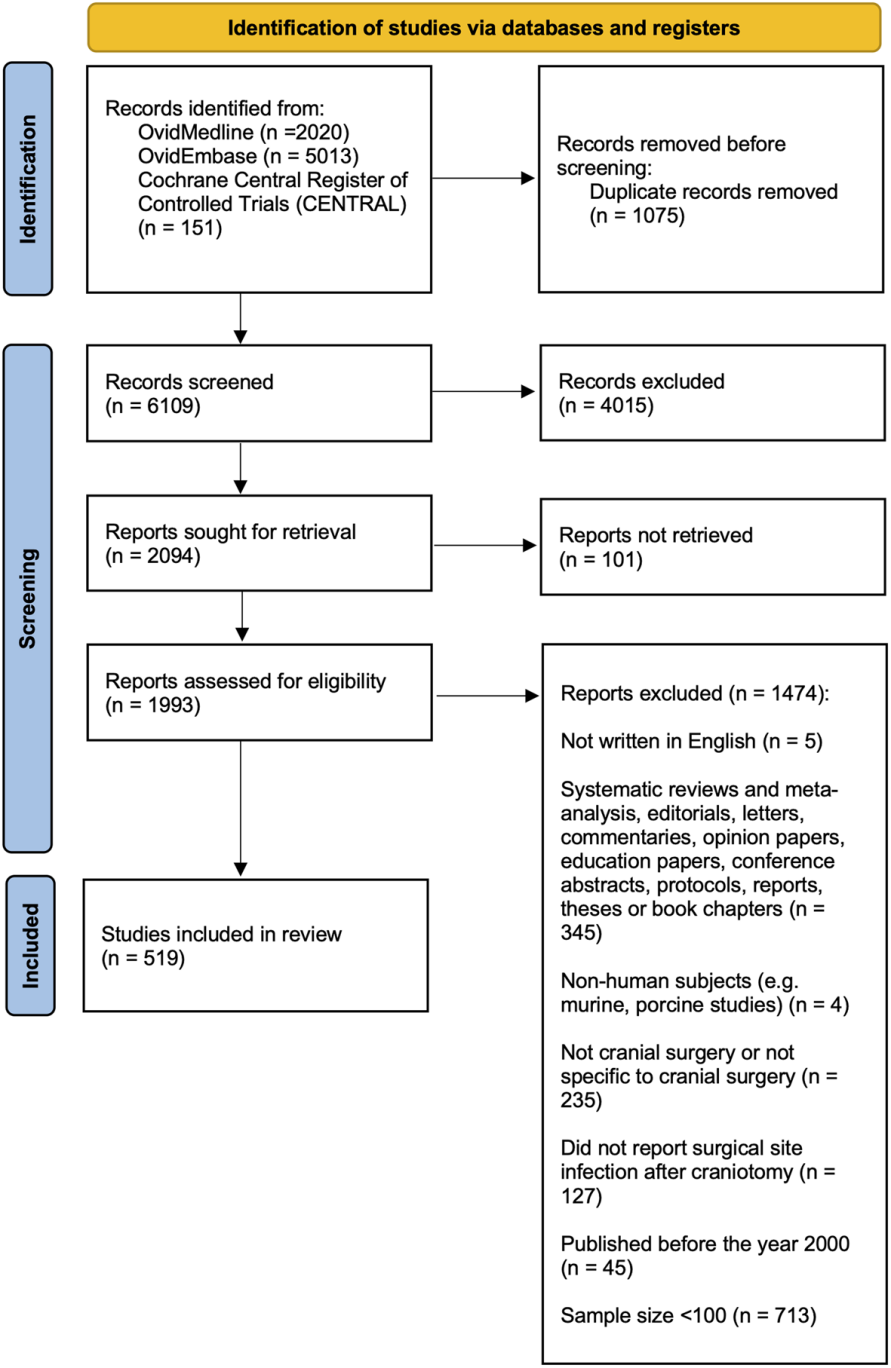
PRISMA flow diagram for studies included and excluded from the systematic review

Following screening of 6119 unique articles, 519 studies reporting data on SSI-CRAN outcomes were included in the final dataset (Fig. 1). The reliability of study selection was substantial at both the title and abstract (k = 0.78), and full text stages (k = 0.87) [27].

### Characteristics of included studies

The characteristics of the 519 primary studies, including their Digital Object Identifiers (DOIs), are presented in Supplementary Table 3.

A hundred and three (19.8%) of the included studies were in neuro-oncology, 92 (17.7%) in neurotrauma, 56 (10.8%) in paediatric neurosurgery (10.8%), 52 (10.0%) in functional surgery), 38 (7.3%) in neurovascular, 37 (7.1%) in skull base, 11 (2.1%) in CSF (cerebrospinal fluid) dynamics (2.1%), and 149 (28.7%) in general neurosurgical cases. The remaining were of overlapping subspecialities.

On assessing the risk of bias using the JBI checklist, 167 (32.2%) studies attained a full score of 11. Seventy (13.5%) studies attained a score of 10, 204 (39.3%) attained a score of 9, 48 (9.3%) attained a score of 8, 24 (4.6%) scored 7, and six (1.1%) scored 6.

### Whether SSI-CRAN was defined in the study

A hundred and sixty-nine (32.6%) of the 519 included studies provided a definition of SSI-CRAN in their methods, with 163 (31.4%) studies specifying indicators that represented SSI-CRAN. Across the 519 studies reporting SSI-CRAN, a definition of SSI-CRAN was not provided in 350 (67.4%) studies reporting an SSI-CRAN outcome. There was no association between studies with higher overall quality and clearer definitions of SSI-CRAN (Supplementary Table 4).

### Indicators used to define SSI-CRAN

It is well established that different signs are relevant at various depths of SSI. The major categories of wounds – superficial, deep and organ space infections – should be considered separately to minimise the possibility of overlooking the presence of infection. Superficial infections typically present with localised signs that correspond to the following SSI indicators: erythema and purulence, while deep infections involving underlying tissue layers present with systemic signs that correspond to SSI indicators such as fever and increased white blood cell count. Organ space infections have been characterised by indicators such as meningitis or ventriculitis.

Eighty-seven (51.5%) of the included studies defining SSI-CRAN referred to the varied signs of SSI-CRAN at different depths following cranial surgery.

**Fig. 2:**
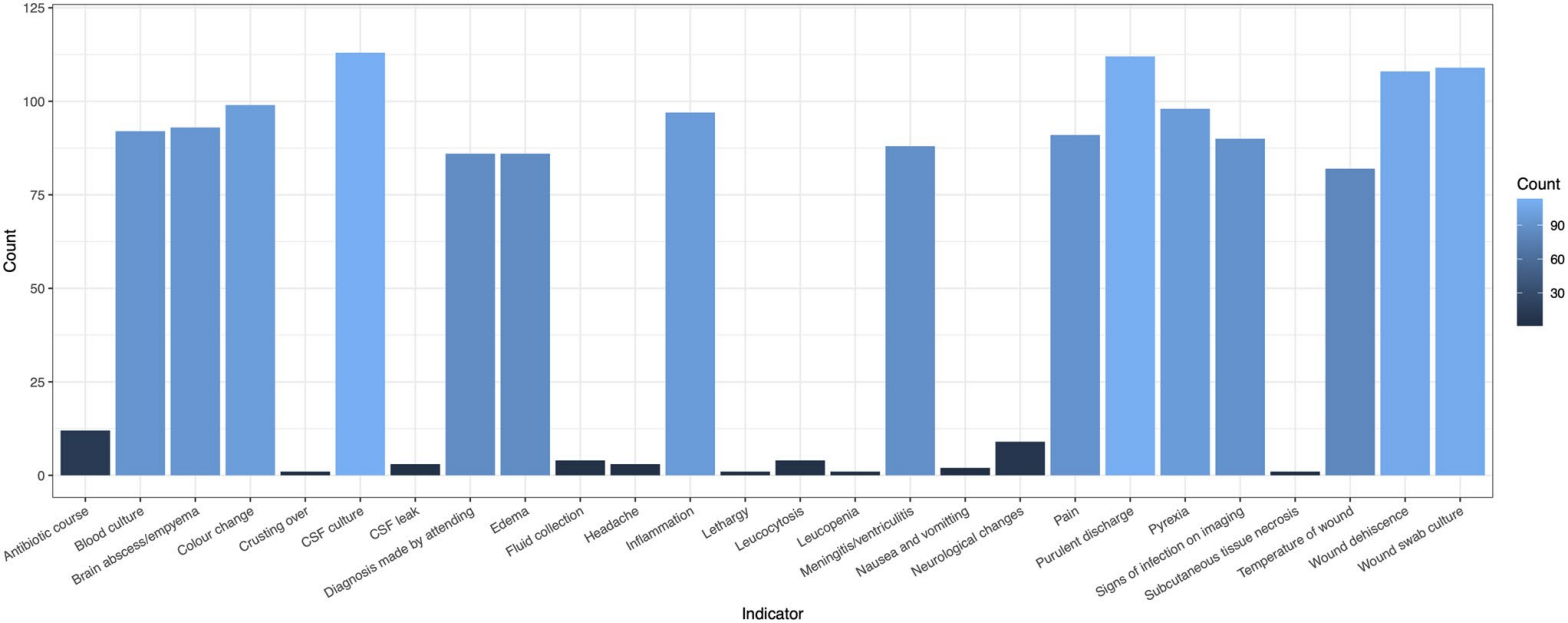
Number of studies employing the relevant indicators to diagnose SSI-CRAN (*n* = 519 studies)

The indicators used to define SSI-CRAN in the 163 studies (31.4%) that reported the use of one or more specific indicators are presented in Supplementary Table 5. Twenty-six different indicators were used to define SSI-CRAN across all these studies (Fig. 2). The median number of indicators across the included studies was nine, with a range of one to 16 indicators.

From the studies defining SSI-CRAN, the most frequently reported indicators were the presence of pus or purulent discharge (*n* = 112; 66.3% of studies defining SSI), presence of bacteria in the wound, identified from swab of pus or exudate (*n* = 109; 64.5%), wound dehiscence or wound reopened by surgeon (*n* = 108, 63.9%), change in colour or erythema of wound (*n* = 99; 58.6%), oedema (*n* = 86; 50.9%), pyrexia (*n* = 98; 58.0%), pain (*n* = 91; 53.8%) and positive CSF culture (*n* = 113; 66.9%).

Seven (4.3%) of the 163 studies defining indicators for SSI-CRAN used indicators that represented a group of signs and symptoms: cellulitis was used as an indicator to define SSI-CRAN in five (3.0%) studies and clinical symptoms or findings in two (1.2%) studies.

Several studies employed non-specific indicators including ‘clinician diagnosis of SSI-CRAN’ (*n* = 87, 53.0%), ‘cellulitis’ and/or ‘meningitis’ (*n* = 96, 58.9%) and ‘clinical signs’ which were not specified (n = 2, 1.2%).

Administration of antibiotics was used to define presence of SSI-CRAN in 12 of the 163 (7.4%) studies.

Seventeen (10.4%) studies reported the use of only one indicator. Of these, 10 (58.5%) used a positive culture (eight for CSF culture, two for wound swab culture) as an indicator of SSI. From the remaining seven studies, six (85.7%) used the requirement for reoperation and one (14.3%) used blood results including CRP as the sole indicator for SSI.

### Whether the same indicators were used to define SSI-CRAN across studies

Of the 169 studies providing a definition for SSI-CRAN, 82 (48.5%) studies used the same definition of SSI-CRAN, guided by the Centers for Disease Control and Prevention (CDC) guidelines (definitions provided in Supplementary Table 6). These guidelines outlined 15 indicators and covered three categories of SSI: superficial SSI, deep SSI and organ-space SSI [28]. It requires a follow-up of 30 days post-operatively, extended to 90 days if an implant is present. However, it was noted that many studies that applied these guidelines frequently altered this follow-up period. It is also important to note that the CDC guidelines include a ‘diagnosis made by surgeon or attending physician’ as part of their criteria which may contribute to the variations in the incidence of SSI reported across these studies.

Other guidelines used by authors included the National Surgical Quality Improvement Program (NSQIP) guidelines for SSI following deep brain stimulator insertion (*n* = 1, 0.6%), the UK Health Protection Agency guidance (*n* = 1, 0.6%), and the Protocol for Surveillance of SSI published by Public Health England (*n* = 2, 1.2%) which assessed for 11 indicators covering superficial, deep, and organ-space SSIs (Supplementary Table 6).

In 10 of the 17 (58.8%) studies that used a single indicator to define SSI-CRAN, it was defined as the presence of an organism isolated from culture (eight based on CSF culture and two on wound swab or tissue culture). Among the 17 studies that used two indicators to define SSI-CRAN, two (11.8%) used the same indicators to define it: wound dehiscence or wound requiring reoperation alongside the initiation of an antibiotic course to treat suspected infection.

### Numerical values for indicators used to determine the presence of SSI-CRAN

Out of the 109 studies using a positive wound swab or tissue culture as an indicator for SSI-CRAN, only one (0.9%) study stated that SSI-CRAN was defined as > 10^5^ colony forming units per gram of tissue. The remaining studies did not clearly report what numerical values were used to detect the presence of bacteria in the wound.

### Whether a method for combining data from several indicators to determine presence of SSI-CRAN was specified (e.g. a count of the number of indicators present or a weighted scoring system)

A total of 144 (27.7%) studies used more than one indicator to define SSI-CRAN. Of these, none reported a method for rating or combining data from the multiple indicators used to determine whether SSI-CRAN was present.

### Primary study conclusions about intervention effectiveness and impact of heterogeneity of SSI-CRAN definition on them

Of the 169 primary studies that defined indicators for SSI-CRAN, 160 (95.0%) reported the effectiveness of the intervention assessed in their study, of which 21 (13.1%) studies suggested that their results should be interpreted with caution due to insufficient quality of the data, limiting the ability to draw effective conclusions surrounding SSI-CRAN outcomes. Four (2.4%) primary studies, which defined SSI-CRAN, did not specify conclusions on the effectiveness of the intervention for SSI-CRAN.

Of the 169 primary studies that reported definitions for SSI-CRAN, 18 (11.0%) addressed the heterogeneity of SSI-CRAN definitions in their conclusions summarising the effectiveness of the assessed intervention. In 145 studies that reported criteria for SSI-CRAN, the authors did not take into consideration the varying definitions of SSI across literature in their findings.

Three of the 350 (0.8%) studies which provided no definition of SSI addressed this heterogeneity of SSI-CRAN definitions in their findings. Three main types of conclusions were identified across the 21 studies that referred to heterogeneity of SSI-CRAN definition in their conclusions. Across the 21 studies that acknowledged the varying definitions of SSI-CRAN, three main types of conclusions were drawn. In two studies, the authors specified they could not accurately analyse the findings of the intervention’s efficacy for SSI-CRAN due to the discrepancies in SSI definition present across literature. In 11 studies, the authors advised to interpret their conclusions with caution due this heterogeneity. The conclusion of two studies indicated no significant effect of the intervention was found, with the authors suggesting this may have resulted from the various differences in SSI-CRAN definitions.

Of the 350 studies that did not specify any indicators for SSI-CRAN, 175 (50.0%) stated the effectiveness of the intervention for SSI-CRAN, of which three (1.7%) mentioned that their results surrounding SSI-CRAN should be interpreted cautiously due to the insufficient quality of the data.

No conclusion regarding the effectiveness of the intervention was drawn from the other 181 (51.7%) studies.

From the 498 studies that did not mention data in their results regarding variations of SSI-CRAN definition across literature, only two (0.4%) authors considered the variation in the indicators used in their own study. In two studies, the authors specified that they could not assess their findings as a result of the discrepancy in SSI-CRAN definition, whilst in 11 studies, the authors advised to cautiously review their conclusions due to this heterogeneity in definition.

## Discussion

### Summary of findings

This systematic review aimed to examine whether there is variation in SSI-CRAN definitions across neurosurgical studies. It also aimed to determine whether primary authors have accounted for heterogeneity in definitions of SSI-CRAN when drawing conclusions about intervention effectiveness for patients that have undergone cranial surgery. Of the 519 included studies, only 163 (31.4%) provided a definition of SSI-CRAN and described the indicators used to define it. There was substantial variation in both the number and types of indicators used to define SSI-CRAN across the studies included. Twenty-six different indicators of SSI-CRAN were used across the included studies. The choice of indicators was influenced by both patient demographic and study type. For example, retrospective studies had a smaller range of SSI-CRAN indicators in comparison with prospective studies.

Our findings of variation in SSI-CRAN definitions, align with observations from other clinical fields [29]. Synthesis of data where there is heterogeneity in outcome definition may reduce the validity of systematic review findings. It is therefore prudent to question the findings of future systematic review that have not accounted for heterogeneity in SSI-CRAN definitions before it is applied to clinical practice.

### Implications for research and practice

This systematic review highlights the requirement for future research involving systematic reviews to assess the effectiveness of interventions for SSI-CRAN, whilst accounting for the variability in the definitions of the measured outcome. Additionally, greater standardisation for reporting SSI-CRAN is required whilst assessing outcomes of interventions, in order to support evidence synthesis. From this review, it is evident that a difficulty faced in achieving this standardisation is the lack of an objective method for determining the presence of SSI-CRAN. A proposed solution is the use of an agreed minimum set of indicators, such as those outlined in the CDC Guidelines, to aid in consistent reporting of SSI-CRAN across literature. This approach would enhance the validity of evidence synthesis in future trials [29]. 

Further to the above, a proportion of studies had made use of the CDC guidelines as a standard definition for determining SSI-CRAN. Therefore, another proposed definition for determining the presence of SSI could be in reference to these guidelines. However, a frequent limitation faced with the use of these guidelines is the post-operative timeframe used to identify SSI-CRAN. Several studies adopted an extended timeframe beyond the period specified in the CDC guidelines. One study reported that 75% of cases for SSI-CRAN were identified on an average of 49 days post-operatively, with 37% presenting outside of the 30-day period of surveillance window recommended by the CDC guidelines for superficial SSI. The authors acknowledged the extended surveillance period and suggested longer periods of surveillance for cranial procedures to improve the detection of SSI-CRAN [30].

Another potential avenue to explore in future studies is the implementation of a numerical cut-off for confirming the presence of SSI-CRAN such as the presence of > 10^5^ colony forming units per gram of tissue in swab or tissue cultures. Only one study clearly mentioned the use of this as an indicator. Wider use of this indicator could allow easier comparison between studies.

### Strengths and limitations of the review

This systematic review adhered to a pre-specified, registered protocol, with any deviations from the protocol clearly documented. Three databases were searched to identify relevant studies, making the literature search comprehensive. The reporting of this systematic review conforms to the PRISMA guidelines [22]. 

The limitations of this systematic review include the selection of only publications written in English, due to resource constraints. Consequently, there is a possibility that appropriate studies published in other languages were not included in our review. To reduce the risk of selection bias, international publications were included. The search terminology used may have led to the exclusion of some publications that could otherwise be relevant to this review. This restriction was, however, necessary to maintain a clear focus on evaluating papers that specifically reported SSI following cranial surgery, while avoiding studies where the infection site was unclear, such as those involving ventriculo-peritoneal shunt infections.

## Conclusion

This study indicates significant variability in the definitions of SSI-CRAN across the included studies assessing outcomes following cranial surgery. This variability in the definitions of SSI-CRAN undermines the validity of the available evidence for identifying effective interventions for SSI-CRAN [22]. As a result, standardising SSI-CRAN outcome assessments is required in order to facilitate effective evidence synthesis, which will allow improved understanding of the intervention effectiveness following cranial surgery. A proposed solution is the development and implementation of a core set of indicators to identify SSI-CRAN consistently across the literature.

## Electronic supplementary material

Below is the link to the electronic supplementary material.

Supplementary Material 1

Supplementary Material 2

Supplementary Material 3

Supplementary Material 4

## Data Availability

No datasets were generated or analysed during the current study.
