# Supplementary material for: Heterogeneity in definitions of surgical site infection after cranial surgery limits the validity of research findings in neurosurgery: a systematic review
Source: Neurosurg Rev. 2025 Jan 16;48(1):59. doi: 10.1007/s10143-025-03218-5 (PMC11739257; doi:10.1007/s10143-025-03218-5)
Supplement: Supplementary file 1 — Supplementary Material 1 [file 10143_2025_3218_MOESM1_ESM.docx]

Supplementary Table 1. Search strategy used for the three electronic databases 24^th^ February 2023

| **EMBASE search** | | **5013 articles** | |
| --- | --- | --- | --- |
| No. | Search term | | |
| **Surgical site infection concept** | | | |
| 1 | Exp surgical infection/ | | |
| 2 | (SSI or surgical site infection).tw. | | |
| 3 | 1 or 2 | | |
| **Neurosurgery concept** | | | |
| 4 | exp brain/ or exp skull/ | | |
| 5 | (brain or cranial or skull).tw | | |
| 6 | 4 or 5 | | |
| 7 | Exp surgery/ or surgery.tw | | |
| 8 | 6 and 7 | | |
| 9 | Exp brain surgery/ or exp neurosurgery/ or exp craniotomy/ | | |
| 10 | (neurosurgery or cranial surgery or craniotomy).tw | | |
| 11 | 9 or 10 | | |
| 12 | 8 or 11 | | |
| **Combined concepts** | | | |
| 13 | 3 and 12 | | |
| **OVID Medline search** | | | **2020 articles** |
| No. | Search term | | |
| **Surgical Site Infection concept** | | | |
| 1 | Exp Surgical Wound Infection/ | | |
| 2 | (SSI or surgical site infection).tw. | | |
| 3 | 1 or 2 | | |
| **Neurosurgery concept** | | | |
| 4 | exp brain/ or exp skull/ | | |
| 5 | (brain or cranial or skull).tw | | |
| 6 | 4 or 5 | | |
| 7 | Exp surgery/ or surgery.tw | | |
| 8 | 6 and 7 | | |
| 9 | Exp neurosurgical procedures/ or exp neurosurgery/ or exp craniotomy/ | | |
| 10 | (neurosurgery or cranial surgery or craniotomy).tw | | |
| 11 | 9 or 10 | | |
| 12 | 8 or 11 | | |
| **Combined concepts** | | | |
| 13 | 3 and 12 | | |
| **Cochrane Central Register of Controlled Trials (CENTRAL**) | | **151 articles** | |
| No. | Search term | | |
| **Surgical site infection concept** | | | |
| 1 | MeSH descriptor: [Surgical Wound Infection] explode all trees | | |
| 2 | (SSI or surgical site infection):ti,ab,kw | | |
| 3 | #1 or #2 | | |
| **Neurosurgery concept** | | | |
| 4 | MeSH descriptor: [neurosurgical procedures] explode all trees | | |
| 5 | MeSH descriptor: [neurosurgery] explode all trees | | |
| 6 | MeSH descriptor: [craniotomy] explode all trees | | |
| 7 | (neurosurgery or cranial surgery or craniotomy):ti,ab,kw | | |
| 8 | #4 or #5 or #6 or #7 | | |
| **Combined concepts** | | | |
| 12 | #3 and #8 | | |

Supplementary Table 2. Inclusion and exclusion criteria used to select studies for the review

| Inclusion criteria | Exclusion criteria |
| --- | --- |
| Primary (interventional/observational or retrospective/prospective) studies assessing the incidence and/or risk factors or interventional for surgical site infection after cranial surgery | - Not written in English - Systematic reviews and meta-analyses, editorials, commentaries, opinion papers, letters, education papers, conference abstracts, protocols, reports, theses or book chapters - Non-human subjects (e.g. murine, porcine studies) - Not specifically cranial surgery - Did not report SSI-CRAN after cranial surgery - Published before 2000 - Sample size <100 |

Supplementary Table 3. Summary of 519 included studies

| First author, year | DOI  (or PMID) | Country | Study design | Type of patients | Type of cranial surgery | Primary aim of study | Intervention and comparator reviewed | Is SSI-CRAN an outcome of interest stated in the methods? | If defined, the criteria applied for SSI-CRAN | What are the findings relating to SSI-CRAN? | Do the conclusions take account of variations in diagnostic criteria? |
| --- | --- | --- | --- | --- | --- | --- | --- | --- | --- | --- | --- |
| Abdullah K.G. et al., 2015 | [10.3171/2014.12.JNS142092](https://doi.org/10.3171/2014.12.jns142092) | USA | Retrospective cohort | Adult | General neurosurgical (craniotomy) | To assess whether topical vancomycin, applied in powder form directly to the subgaleal space during closure, would reduce cranial wound infection rates. | Use of topical vancomycin vs control (no topical vancomycin). | Yes | Not defined | A total of 6 patients experienced infection (overall incidence of 4%): 5 controls and 1 of 75 patients who received vancomycin. The overall incidence of infection in the control group was 6.7% and in the experimental group was 1.3% | No |
| Abebe F.T. et al., 2022 | [10.1016/j.inat.2022.101602](https://doi.org/10.1016/j.inat.2022.101602) | Ethiopia | Prospective cohort | Paediatric | Neuro-oncology | To evaluate the rate, indications and predictors of unplanned reoperation after craniotomy for brain tumors in two teaching hospitals. |  | Yes | Not defined | The most common indication of reoperation was postoperative surgical site infection (25%, 8 cases). | No |
| Abecassis I. et al., 2021 | [10.3171/2020.11.JNS203567](https://doi.org/10.3171/2020.11.jns203567) | USA | Retrospective cohort | Adult | Trauma | To define the implementation of the Kempe incision for decompressive craniotomy, report clinical outcomes, and quantify the volume of bone removed compared  with the reverse-question mark incision. | Kempe incision Vs reverse-question mark incision | Yes | Not defined | Wound infection rates and length of surgery were comparable between the two incision types. | No |
| Abode-Iyamah K.O. et al., 2018 | [10.3171/2016.12.JNS161967](https://doi.org/10.3171/2016.12.jns161967) | USA | Retrospective cohort | Adult | General neurosurgical (cranioplasty) | To determine whether intrawound VP (vancomycin powder) is associated with decreased risk of SSIs, to evaluate VP’s safety, and to identify risk factors for SSIs after cranioplasty among patients undergoing first-time cranioplasty. |  | Yes | CDC guidelines | 15 out of 258 (5.8%) patients  acquired SSIs. Ninety-two patients (35.7%) received intrawound VP (VP group) and 166 (64.3%) did not (no-VP group). Risk factors for SSI from were diabetes, multiple craniotomy procedures before the cranioplasty, prior same-side craniotomy, and prosthetic implants. | Yes: "The definitions of SSI used  in the studies evaluated by Yadla et al. varied, and most of  the studies did not use the NHSN definition, which may  explain, in part, the difference between the results of our  study and those of the systematic review." |
| Abode-lyamah K.O. et al., 2018 | [10.3171/2017.9.JNS1780](https://doi.org/10.3171/2017.9.jns1780) | USA | Retrospective cohort | Adult | Functional (DBS insertion) | To identify possible risk factors for DBS-related infection and analyse the efficacy of prophylactic intrawound vancomycin powder. | Use of vancomycin powder vs control (not used). | Yes | CDC guidelines | Nine SSIs (3.7%) were detected within 90 days of DBS placement, and 16 (6.5%) were detected within 1 year. The median time to the onset of all 16 SSIs was 79 days; for the 9 SSIs that occurred fewer than 90 days after the procedure, the median time to onset was 50 days. Eleven infections (68.8%) affected scalp wounds; 9 of these occurred at or near burr holes and 2 were associated with lead extensions. | No |
| Abulhasan Y.B. et al., 2018 | [10.1016/j.wneu.2018.04.061](https://doi.org/10.1016/j.wneu.2018.04.061) | Canada | Retrospective cohort | Adult | Trauma | To describe the epidemiology of healthcare associated infections after SAH.  To identify clinical and disease-specific risk factors as predictors of developing hospital acquired infections (HAIs).  To analyse the association of HAIs with patient-related outcomes. |  | Yes | CDC guidelines | Reported 0.7% frequency of infections. | No |
| Adeleye A., 2016 | [10.1007/s10143-016-0700-4](https://doi.org/10.1007/s10143-016-0700-4) | Nigeria | Prospective cohort | Adults and Paediatrics | General neurosurgical (cranial surgery) | The primary outcome measure was the rate and types  of surgical site infections (SSI). |  | Yes | Not defined | SSIs occurred in only 10 cases (3.3 %). The type of surgery, redo or primary, did not have any significant association with the in-hospital outcome or with the presence of SSI. | No |
| Adeleye A., 2017 | [10.1080/02688697.2017.1407746](https://doi.org/10.1080/02688697.2017.1407746) | Nigeria | Retrospective cohort | Adults and paediatrics | General neurosurgical (cranial surgery) | To audit SSI following major cranial surgery in a sub-Saharan African academic neurosurgical unit. |  | Yes | CDC guidelines | Surgical site infections occurred in 9 cases, 4.3%. The presence of SSI was associated with a longer length of stay in hospital. Majority of the SSI  were by Gram negative organisms. | No |
| Agarwal N. et al., 2017 | [10.1093/neuros/nyx273](https://doi.org/10.1093/neuros/nyx273) | USA | Prospective cohort | Not reported | General neurosurgical (craniotomy) | To investigate the effects of increased physician awareness on infection incidence and surgical device cost containment. | Intervention of education and nasal decolonisation of patients with staphylococcus aureus | Yes | CDC guidelines | The craniotomy infection incidence decreased from a baseline of 3.0% (May 2011- April 2015) to 2.0% (May 2015- April 2016). Therefore, a 33% reduction of post-craniotomy infection incidence was achieved with the implementation of a physician awareness intervention and preoperative bacterial load management. | No |
| Ahmad N. et al., 2008 | [10.1097/SCS.0b013e31816ae358](https://doi.org/10.1097/scs.0b013e31816ae358) | USA | Retrospective cohort | Paediatrics | Paediatrics | To evaluate the application of a  bioabsorbable fixation system in reconstructive  craniofacial procedures in a paediatric population. |  | Yes | "Signs of infection": tenderness, erythema, induration, purulent effluent. | Five patients (3.4%) had signs of infection at the incision site. Two of these 5 patents were treated  in the hospital with incision and drainage and intravenous antibiotics. | No |
| Ahmadi R et al., 2021 | [10.3109/02688697.2016.1161726](https://doi.org/10.3109/02688697.2016.1161726) | Germany | Prospective case-control | Adults and Paediatrics | Neuro-oncology | To evaluate perioperative anaesthesiological and surgical complications related to brain tumour surgery using intra-operative MRI (io-MRI). | Use of io-MRI vs control (without io-MRI) | Yes | Not defined | There was no significant difference in the number of reported SSIs with 11 infections reported in each the MRI and control cohorts. | No |
| Al-Sharydah A.M. et al., 2020 | [10.15537/smj.2020.6.25095](https://doi.org/10.15537/smj.2020.6.25095) | Saudi Arabia | Retrospective cohort | Adults and Paediatrics | CSF dynamics | To assess the incidence of infection following  various cerebrospinal fluid (CSF) shunt procedures among patients with hydrocephalus and related conditions, based on the age of the patient, and to determine the types of pathogens responsible for shunt infections. |  | Yes | “Positive microbiological culture within 120 hours after  surgery, a postoperative fever (temperature >38.2°C),  and available CNS imaging data." | Of the patients with shunt infections, 32.3% had a positive CSF culture. Incidence of infection highest among patients with an EVD followed by patients with an VPS and an ETV. The incidence of infection differed significantly based on the type of procedure conducted. | No |
| Al-Tamini Y.Z. et al., 2012 | [10.3109/02688697.2011.633640](https://doi.org/10.3109/02688697.2011.633640) | UK | Retrospective cohort | Adult | Trauma | To compare the survival of acrylic and titanium cranioplasties used in our department. | Titanium vs Acrylic cranioplasties | Yes | Not defined | "11 out of the 13 failures were due to wound infection." | No |
| Alan N. et al., 2015 | [10.1016/j.jocn.2015.03.009](https://doi.org/10.1016/j.jocn.2015.03.009) | USA | Retrospective cohort | Adult | Neuro-oncology | To study the impact of preoperative steroids on 30-day morbidity and mortality of craniotomy for definitive resection of malignant brain tumours. |  | Yes | Not defined | Reports deep SSI in 0.3% of chronic steroid use group preoperatively compared to 0.5% in control group. Superficial SSI occurred in 1% of chronic steroid use group compared to 0.5% in control group. Organ or space SSI occurred in 1.4% of chronic steroid use group compared to 1% in control group. | No |
| Alford E.N. et al., 2020 | [10.1007/s10143-020-01296-1](https://doi.org/10.1007/s10143-020-01296-1) | USA | Retrospective cohort | Adult | Vascular/Other (Microvascular decompression) | To identify risk factors associated with wound-related complications after retrosigmoid  suboccipital approaches for microvascular decompression. |  | Yes | Not defined | 10 patients (5.1%) had a wound infection. 9 patients (4.6%) had wound dehiscence. | No |
| Alkhaibary A. et al., 2019 | [10.1016/j.wneu.2019.09.120](https://doi.org/10.1016/j.wneu.2019.09.120) | Saudi Arabia | Retrospective cohort | Adults and Paediatrics | Other (Craniectomy for intracranial hypertension) | To estimate the incidence of SSI and determine its possible risk factors for patients who underwent cranioplasty using bone flaps subcutaneously preserved in abdominal pockets. |  | Yes | Positive wound/tissue culture with clinical signs and symptoms of infection | The incidence of SSI was noted in 15.7%. The most common predictors of infection in patients requiring cranioplasty were blood glucose levels and a large defect size. | No |
| Alwadei A. et al., 2019 | [10.1016/j.wneu.2019.01.262](https://doi.org/10.1016/j.wneu.2019.01.262) | Saudi Arabia | Retrospective cohort | Adults and Paediatrics | General neurosurgical (Craniotomy) | To examine the possible  benefits and harms of suturing the dura compared with no  dural closure and the occurrence of postoperative infection,  cerebrospinalfluid (CSF) leak, and postcraniotomy headaches |  | Yes | Not defined | The open group experienced a greater incidence of infection and CSF leak (6 in the open group vs. 2 in the closed group), but without statistical significance. | No |
| Alzoubi F. et al., 2015 | [10.1179/1754762814Y.0000000090](https://doi.org/10.1179/1754762814y.0000000090) | Jordan | Retrospective cohort | Adults and Paediatrics | Other (cochlear implant) | To assess the postoperative surgical complications, morbidity, and outcome of the lazy S-shaped postauricular incision with a modified double-flap technique for  cochlear implant surgery. |  | Yes | Not defined | Four (1.2%) minor complications including one case of wound infection and discharge that required  hospitalisation for intravenous antibiotic administration. | No |
| Ammanuel S.G. et al., 2021 | [10.3171/2020.10.JNS201255](https://doi.org/10.3171/2020.10.jns201255) | USA | Retrospective cohort | Adult | General neurosurgical (craniotomy) | To assess the efficacy of preoperative chlorhexidine gluconate (CHG) showers on SSI rates following cranial surgery. |  | Yes | CDC guidelines | No significant differences (p = 0.11) were observed between the rate of SSI of the 892 patients in the preimplementation cohort (0.2%) and that of the 2234 patients in the postimplementation cohort (0.8%). | No |
| Ammar R. et al., 2022 | [10.1016/j.inat.2021.101421](https://doi.org/10.1016/j.inat.2021.101421) | Tunisia | Retrospective cohort | Adult | General neurosurgical (craniotomy) | To analyse clinical, epidemiological, radiological characteristics and outcome of patients undergoing decompressive craniectomy after traumatic brain injury (TBI) and to define predictive factors associated with poor prognosis. |  | Yes | Not defined | 7 cases of local infection reported. | No |
| Andren K. et al., 2018 | [10.1007/s00415-017-8680-z](https://doi.org/10.1007/s00415-017-8680-z) | Sweden | Retrospective cohort | Adults and Paediatrics | Trauma | To describe the long-term outcome of idiopathic normal pressure hydrocephalus patients, the incidence and influence of reoperation due to complications, and the influence of vascular risk factors and vascular comorbidity on outcome. |  | Yes | Not defined | 6.4% of patients had reoperations due to infections. | No |
| Anegbe A.O. et al., 2019 | [10.1007/s00381-019-04219-8](https://doi.org/10.1007/s00381-019-04219-8) | Nigeria | Retrospective cohort | Paediatric | Paediatric/Trauma | To determine the profile of and risk factors for the development of intracranial infections in children with myelomeningocele presenting for care in our facility. |  | No | Not defined | One significant risk factor for intracranial infection in this study was infection at the myelomeningocele site. | No |
| Ansari S. et al., 2020 | [10.1093/ons/opaa217](https://doi.org/10.1093/ons/opaa217) | USA | Retrospective cohort | Adult | Neuro-oncology | To present a series of patients treated with the supraorbital eyebrow craniotomy approach to assess outcomes, the impact of endoscopy, and describe a modified pericranial flap aimed at  reducing postoperative frontalis paresis and hypesthesia. |  | No | Not defined | 2 cases of wound infection (1.6%). No patients had meningitis, although  2 patients developed superficial wound infections treated with  antibiotics. | No |
| Aras M. et al., 2014 | [0.1016/j.clineuro.2014.08.019](https://doi.org/10.1016/j.clineuro.2014.08.019) | Turkey | Retrospective cohort | Adults and Paediatrics | Trauma | To examine and retrospectively analyse the medical records of the patients treated in the clinics of our hospital due to cranial gunshot wounds during the war. |  | Yes | Not defined | SSI developed in 5 patients. | No |
| Arita H. et al., 2014 | [10.1007/s11060-013-1273-5](https://doi.org/10.1007/s11060-013-1273-5) | Japan | Retrospective cohort | Adults | Neuro-oncology | To describe a retrospective analysis of the relationship between clinical characteristics and the outcome of surgery for brain metastases, and we discuss the indications for and the role of surgery. |  | No | Not defined | One instance of surgical site infection | No |
| Arnautovic K.I. et al., 2020 | [10.1016/j.wneu.2020.11.102](https://doi.org/10.1016/j.wneu.2020.11.102) | USA | Prospective cohort | Adult | Other (Chiari malformation decompression) | To measure the surgical outcomes related to  resolution/ improvement of headaches, neurological outcomes, and syringomyelia compared with  published adult CM-I studies from 2000–2019. |  | No | Not defined | Four patients developed superficial  wound infection (all female): 3 needed re-operation (debridement, washout, closure), and one  was healed only with antibiotics and dressing changes. | No |
| Arnone G. et al., 2020 | [10.1055/s-0040-1715811](https://doi.org/10.1055/s-0040-1715811) | USA | Retrospective cohort | Adult | Skull base | To report the incidence and management of venous sinus compromise following skull  base surgery around the sigmoid sinus. |  | No | Not defined | Reported 2 cases (15.4%) of surgical infection in venous sinus compromise group and 7 cases (6.9%) of infection in no venous sinus compromise group. | No |
| Arocho-Quinones E.V. et al., 2019 | [10.1016/j.wneu.2019.04.003](https://doi.org/10.1016/j.wneu.2019.04.003) | USA | Retrospective case-control | Adults and Paediatrics | Functional (DBS insertion) | To evaluate the effectiveness of an infection prevention bundle (IPB) in minimising infections after surgeries for neuromodulation implants. |  | Yes | CDC guidelines | 11 cases vs 1 cases of infection between pre and post IPB group following 90-day surveillance. 15 cases vs 3 cases following 1 year surveillance. | No |
| Arts S. et al., 2018 | [10.3171/2017.7.PEDS17155](https://doi.org/10.3171/2017.7.peds17155) | The Netherlands | Prospective case-control | Paediatric | Paediatric/Skull base | To compare minimally invasive endoscopic and open surgical procedures, to improve informed consent of parents, and to establish a baseline for further targeted improvement of surgical care.  To evaluate the complication rate and blood transfusion rate of craniosynostosis surgery. | Minimally invasive vs open surgical procedures | No | Not defined | 1 wound infection was recorded. | No |
| Attenello F. et al., 2008 | [10.1245/s10434-008-0048-2](https://doi.org/10.1245/s10434-008-0048-2) | USA | Retrospective cohort | Adult | Neuro-oncology | To characterise Gliadel-associated morbidity in our 10- year experience with Gliadel wafers for treatment of malignant glioma. |  | Yes | Not defined | SSI was observed in eight patients (3%). Patients in Gliadel versus non-Gliadel cohorts had similar incidences of perioperative surgical site infection (2.8% vs. 1.8). | No |
| Aum D.J. et al., 2023 | [10.1111/epi.17679](https://doi.org/10.1111/epi.17679) | USA | Retrospective cohort | Paediatric | Paediatric | The study aims to describe the surgical outcomes of corpus callosotomy (CC) in a large cohort of patients  with medically refractory epilepsy, compare anterior and complete CC, and compare a less invasive LITT approach to open craniotomy for surgical intervention. |  | No | Not defined | 4 cases of wound infection reported. | No |
| Bakhsheshian J. et al., 2018 | [10.1016/j.wneu.2018.07.134](https://doi.org/10.1016/j.wneu.2018.07.134) | USA | Retrospective cohort | Adult | Trauma | To analyse the effect  of the safety-net burden on the FTR (failure to rescue) rates in patients undergoing  surgical management of traumatic brain injury. |  | Yes | Not defined | An evaluation of major complication types revealed that infectious and pulmonary complications comprised a significant proportion of overall noted complications. | No |
| Ban S.P. et al., 2018 | [10.1136/neurintsurg-2021-017352](https://doi.org/10.1136/neurintsurg-2021-017352) | Korea | Prospective cohort | Adult | Vascular/Trauma | To evaluate the effect of middle meningeal artery (MMA) embolisation on chronic subdural hematoma and  compare the treatment outcomes of MMA embolization and conventional treatment. | MMA emobilisation vs conventional treatment (close non-surgical follow-up and haematoma removal) | No | Not defined | Surgical infection occurred in 3 out of 469 patients in the control (conventional treatment) group. | No |
| Barone D.G. et al., 2014 | [10.1002/14651858.CD009685.pub2](https://doi.org/10.1002/14651858.cd009685.pub2) | UK | Retrospective case-control | Adult | Neuro-oncology | To compare image guided surgery with surgery either not using any image guidance or to compare surgery using two different forms of image guidance, |  | Yes | Not defined | No wound infections were reported. | No |
| Bartek Jr J. et al., 2017 | [10.1016/j.wneu.2017.07.044](https://doi.org/10.1016/j.wneu.2017.07.044) | Sweden | Retrospective cohort | Adult | Trauma | To investigate predictors of recurrence and moderate to severe complications after burr-hole surgery for chronic subdural hematoma (cSDH). |  | No | Not defined | Reported cases of wound infections in table (4 cases of wound infection reported). | No |
| Bass D. et al., 2019 | [10.1016/j.wneu.2019.08.066](https://doi.org/10.1016/j.wneu.2019.08.066) | USA | Retrospective cohort | Adult | Functional (Epilepsy) | To determine whether use of an osteoplastic  bone flap technique would reduce the infection rate in patients undergoing neurosurgical procedures for medically intractable epilepsy. | Standard free flaps Vs osteoplastic flaps. | Yes | Not defined | Infection occurred in 24 (17%) patients who underwent standard  craniotomy whilst there were no infections in the osteoplastic cohort.  Standard free flaps were associated with an increased rate of infection at the craniotomy site. | No |
| Batzdorf U. et al., 2013 | [10.3171/2012.10.JNS12305](https://doi.org/10.3171/2012.10.jns12305) | USA | Retrospective cohort | Adults and Paediatrics | Other (Chiari malformation decompression) | To show the relationship between clinical outcome in patients who underwent surgical  decompression for Chiari malformation (CM) and postoperative imaging studies, with particular emphasis on the subarachnoid cisterns of the posterior fossa. |  | Yes | Not defined | 0 wound infections reported | No |
| Baum G.R. et al., 2017 | [10.3171/2016.9.JNS16367](https://doi.org/10.3171/2016.9.jns16367) | USA | Prospective cohort | Adults and Paediatrics | Trauma | To survey the neurosurgical community and determine the most frequent EVD insertion practices. |  | Yes | Not defined | The majority of responders considered a positive CSF culture (42.4%) and positive CSF Gram stain (34.0%) to be the most significant indicators of infection. | No |
| Beckman J. et al., 2015 | [10.3171/2014.12.PEDS13675](https://doi.org/10.3171/2014.12.peds13675) | USA | Retrospective cohort | Paediatric | Paediatric | To evaluate the effectiveness of concentrated bacitracin  powder applied directly to wounds prior to closure during cranial shunt surgery and to evaluate the association between shunt infection and other risk factors. | Bacitracin powder application | Yes | Not defined | A total of 47 infections out of 539 shunt operations occurred during the study period, resulting in an overall  infection rate of 8.7%. | No |
| Bekar A. et al., 2001 | [10.1007/s007010170057](https://doi.org/10.1007/s007010170057) | Turkey | Retrospective cohort | Not reported, likely adult cohort | General neurosurgical (all cranial neurosurgical procedures) | To assess whether this change in routine, which was implemented in 1992, has affected the rate of postoperative infection in our cranial surgery patients. |  | Yes | CDC guidelines | Reported 13 postoperative wound infections (1.25%), including 9 deep (0.87%) and 4 superficial infections (0.39%). There was no significant difference between the rate of infection in patients whose heads were shaven (12/980) and the  rate in those whose hair was spared (13/1038). | No |
| Bekelis K. et al., 2015 | [10.1016/j.wneu.2015.02.032](https://doi.org/10.1016/j.wneu.2015.02.032) | USA | Retrospective cohort | Adult | Neuro-oncology | To attempt to create a predictive model of complications in patients undergoing benign intracranial tumour resection. |  | Yes | Not defined | “The respective inpatient postoperative risks were 1.3% for death, 22.7% for unfavorable discharge, 4.2% for treated hydrocephalus, 1.1% for cardiac complications, 0.9% for respiratory complications, 0.5% for wound infection, 0.5% for DVT, 2.3% for PE, and 1.5% for ARF.” | No |
| Bekelis K. et al., 2017 | [10.1007/s11060-013-1089-3](https://doi.org/10.1007/s11060-013-1089-3) | USA | Retrospective cohort | Adult | Neuro-oncology | To produce a model to  provide individualised estimates of the risks of post-operative complications based on pre-operative conditions, and  can potentially be utilised as an adjunct in the decision making for surgical intervention in brain tumour patients. |  | Yes | Not defined | The 30-day postoperative risks was 2.4 % for deep surgical site infection. The following risk factors were independently associated with a higher risk of the deep surgical site  infection within 30 days: disseminated cancer, weight loss,  tumor-related neurologic deficit  and history of stroke. | No |
| Berghauser Pont L.M.E. et al., 2012 | [10.1227/NEU.0b013e31823672ad](https://doi.org/10.1227/neu.0b013e31823672ad) | The Netherlands | Retrospective cohort | Adult | Skull base | To report our single-center experience with the surgical treatment of CSDH (chronic subdural haematoma) in patients on preoperative corticosteroids and to assess possible predictors of outcome |  | Yes | Not defined | 38.6% vs 10.9% complication rate in recurrent vs no recurrent CSDH groups. | No |
| Bhaskar I.P. et al., 2014 | [10.1016/j.wneu.2013.01.013](https://doi.org/10.1016/j.wneu.2013.01.013) | Australia | Retrospective cohort | Adults and Paediatrics | Trauma | Describe characteristics of patients who develop infections after autogenous cranioplasty. |  | Yes | Not defined | Seventeen patients (median age, 25 years) developed deep infection necessitating explantation of the bone flap | No |
| Bhatti M. et al., 2012 | [10.3109/02688697.2012.743968](https://doi.org/10.3109/02688697.2012.743968) | UK | Prospective cohort | Adult | General neurosurgical (Craniotomy) | To look at the infection rate in adults undergoing craniotomies without hair removal and compare the results with the usual practice of pre-operative shaving/clipping. |  | Yes | CDC guidelines | A total of three patients developed surgical-site infection. One infection occurred in the glioma and two in the meningioma subgroup. | No |
| Bhimani A.D. et al., 2019 | [10.1016/j.wneu.2018.09.079](https://doi.org/10.1016/j.wneu.2018.09.079) | USA | Retrospective case control | Adult | Neuro-oncology | To identify whether a 2-stage approach, intracranial electrode placement followed by ATL (anterior temporal lobectomy), is worthwhile versus ATL alone. | intracranial electrode placement followed by ATL vs ATL alone | Yes | Not defined | Reported 2.5% of cases having organ space SSI, 1.4% superficial SSI, 0.4% deep SSI. | No |
| Bhimani A.D. et al., 2018 | [10.1016/j.wneu.2018.04.077](https://doi.org/10.1016/j.wneu.2018.04.077) | USA | Retrospective cohort | Adult | General neurosurgical (Craniotomy) | To provide a contemporary surgical profile of risk factors and complications for Chiari I malformations in adults. |  | Yes | Not defined | Total of 36 SSIs - 10 superficial, 6 deep, 9 organ space. | No |
| Bjellvi J. et al., 2015 | [10.3171/2014.9.JNS132679](https://doi.org/10.3171/2014.9.jns132679) | Sweden | Prospective cohort | Adults and Paediatrics | General neurosurgical cases | To investigate major and minor complications related to epilepsy surgery in a large, prospective series |  | Yes | Not defined | 1 case of infection as a major complication, and 18 as a minor complication. | No |
| Bjerknes S. et al., 2014 | [10.1371/journal.pone.0105288](https://doi.org/10.1371/journal.pone.0105288) | Norway | Retrospective cohort | Adult | Functional (DBS insertion) | To assess the frequency and possible risk factors of DBS related infections at the author’s centre. To analyse treatment, the clinical and microbiological characteristics of the infections. |  | Yes | CDC guidelines /Guideline for Prevention of SSI | Of the 588 procedures performed 33 (5.6%) led to an infection. Most infections (52%) developed within the first month and 79% within three months. Staphylococcus aureus infections were the most frequent (36%), and more likely to have earlier onset, pus formation, a more aggressive development and lead to hardware removal. | Yes: "Previous studies have reported very  different frequencies of infectious complications, varying from 0% to more than 15% of operated patients. These numbers are difficult to interpret for several reasons, including the lack of consensus regarding definition and criteria of infection, varying  follow-up time, few included patients, different operating techniques and varying peri- and postoperative use of prophylactic  antibiotics. Some centers only include those patients whose infections need surgical intervention and exclude those with superficial infections that can be cured with conservative therapy  alone" |
| Blomstedt P. et al., 2005 | [10.1007/s00701-005-0576-5](https://doi.org/10.1007/s00701-005-0576-5) | Sweden and UK | Retrospective cohort | Adult | Functional (DBS insertion) | To analyse hardware-related complications encountered in this consecutive series of patients treated over a period of ten years with nonmicroelectrode- guided DBS. |  | No | Not defined | 1.5% hardware infection rate reported. | No |
| Bonfield C.M. et al., 2014 | [10.3171/2014.6.PEDS13682](https://doi.org/10.3171/2014.6.peds13682) | USA | Retrospective cohort | Paediatric | Paediatric/ Trauma | To investigate the characteristics, injuries, complications, and outcomes of the patients in whom surgical intervention was needed for skull fractures. |  | No | Not defined | Eight patients (38.0%) had complications related to the surgical procedure, including wound infection. 32 patients (42.8%) in the TBI group had complications: 3 (9.4%) were surgery related, and included cranioplasty resorption and infection. | No |
| Borger V. et al., 2021 | [10.3171/2020.7.JNS20284](https://doi.org/10.3171/2020.7.JNS20284) | Germany | Retrospective case control | Adults and Paediatrics | Functional (temporal lobe resection for epilepsy) | The aim of this study was to evaluate the seizure outcome in patients with drug-refractory temporal lobe epilepsy who underwent resective temporal lobe surgery and to determine features associated with unfavorable postsurgical seizure outcome. |  | Yes | Not defined | Surgical site infections were the most frequent complication (in 9 [5.6%] of 161 patients). | No |
| Branch L.G. et al., 2017 | [10.1097/SCS.0000000000003166](https://doi.org/10.1097/scs.0000000000003166) | USA | Retrospective cohort | Paediatric | Paediatric/ Skullbase | To evaluate our long-term experience with the use of absorbable plating fixation systems for pediatric cranial reconstructions for treatment of craniosynostosis |  | Yes | Not defined | Reported that 4 patients developed SSI. | No |
| Brokinkel B. et al., 2021 | [10.1016/j.clineuro.2020.106315](https://doi.org/10.1016/j.clineuro.2020.106315) | Germany | Retrospective cohort | Adult | Neuro-oncology | To analyse correlations between clinical, radiological and histological variables and the development of new postoperative seizures in a series of 752 patients who underwent surgery for primary diagnosed intracranial meningioma. |  | Yes | Not defined | Reported 36 cases of SSI (6%). SSI was not correlated with postoperative seizures. | No |
| Brommeland T. et al., 2015 | [10.1186/s13049-015-0155-6](https://doi.org/10.1186/s13049-015-0155-6) | Norway | Retrospective cohort | Adults and Paediatrics | Trauma | To identify possible predictive parameters for post-operative complications in cranioplasty. |  | Yes | Not defined | SSI with subsequent removal of the skull implant occurred in 8 out of 87 (9.2 %) cases, of which one was synthetic. | No |
| Buang S.S. et al., 2012 | PMID: 23082448 | Malaysia | Prospective cohort | Adult | General neurosurgical (Craniectomy, craniotomy, cranioplasty, burrhole) | To determine the incidence and risk factors of SSI after craniotomy. |  | Yes | CDC guidelines/ National Healthcare Safety Network Surveillance Definitions | Total of 30 SSI cases out of 390 cases. Included 19 superficial wound infections, 9 bone flap osteitis and 2 with organ/space infections. Mean time between surgery and onset of infection was 11.8 +/- 21.8 days. | No |
| Buchanan I.A. et al., 2018 | [10.1016/j.wneu.2018.08.102](https://doi.org/10.1016/j.wneu.2018.08.102) | USA | Retrospective cohort | Adult | General neurosurgical (Craniotomy) | To use a large hetereogenous patient sample to determine SSI incidence  after nonemergent craniotomy and identify factors associated with readmission and subsequent need for wound washout. |  | Yes | Not defined | There were 2079 cases of SSI (2.2%) and 835 reoperations for washout (0.89%) within 30 days of admission and there were 2761 cases of SSI (3.6%) and 1220 reoperations for washout (1.58%) within 90 days. Several factors were predictive of SSI, including tumour operations, external ventricular drain (EVD), age, length of stay, diabetes, discharge to an intermediate-care facility, insurance type, and hospital bed size. | Yes: "SSIs are one of the most common health care associated infections, with a fiscal footprint equivalent to approximately $3 billion dollars annually. Their reported incidence after craniotomy is variable, ranging from 0.8% to 5.6%. Various risk factors have been implicated; however, many of the data are inconsistent. This factor is likely attributable to immense variation in study inclusion criteria and SSI definitions used throughout the literature" |
| Byoun H.S. et al., 2019 | [10.1016/j.clineuro.2019.105503](https://doi.org/10.1016/j.clineuro.2019.105503) | South Korea | Retrospective cohort | Adults and Paediatrics | Vascular | To investigate the safety and unexpected finding of the intraoperative neuromonitoring (IONM) including somatosensory evoked potentials (SSEPs) and motor evoked potentials (MEPs) during microsurgical clipping of an unruptured anterior choroidal artery (AChA) aneurysm. |  | No | Not defined | 1 case of SSI was recorded. | No |
| Cacciola F. et al., 2001 | [10.1179/joc.2001.13.Supplement-2.119](https://doi.org/10.1179/joc.2001.13.supplement-2.119) | China | Retrospective case-control | Adult | General Neurosurgical (Craniotomy) | To investigate the efficacy of antibiotic prophylaxis and the risk factors for postoperative infections in clean neurosurgery. |  | Yes | CDC guidelines | Reported 31 cases of infections in antibiotic prophylaxis group, and 39 cases of infections in no prophylaxis group. Antibiotic prophylaxis in clean craniotomies had no preventive effect on postoperative infection. | No |
| Campbell E. et al., 2017 | [10.1007/s00381-017-3358-5](https://doi.org/10.1007/s00381-017-3358-5) | UK | Retrospective cohort | Paediatric | Paediatric | To record the 30-day and inpatient morbidity and mortality in paediatric patients in a tertiary neuroscience centre over a 2-year period. The intentions were to establish the frequency of significant adverse events, review the current published rates of morbidity in paediatric neurosurgical patients and propose three clinical indicators for future comparison. |  | Yes | Not defined | Reported 30 cases of SSI or a suspected infection for which treatment was commenced; the overall SSI rate was 5.5%. | No |
| Campioli C. et al., 2022 | [10.1017/ash.2021.258](https://doi.org/10.1017/ash.2021.258) | USA | Retrospective case series | Adult | General neurosurgical (Craniotomy) | To describe demographic characteristics, risk factors, and antibiotic prophylaxis choice in patients with craniotomy complicated with SSI. |  | Yes | CDC guidelines | 5,328 patients undergoing craniotomy were identified during the study period; 59 (1.1%) suffered an SSI. Compared with non-SSI cases, patients with SSI had a significantly higher frequency of emergency procedures. | No |
| Cao Y. et al., 2020 | [10.1186/s13756-020-00784-9](https://doi.org/10.1186/s13756-020-00784-9) | China | Retrospective case-control | Adult | General neurosurgical (Craniotomy) | To conduct an indirect treatment comparison of alternative antibiotic prophylaxis for patients undergoing craniotomy. |  | Yes | Not defined | Cephalosporins, clindamycin, vancomycin and penicillin can significantly reduce the incidence of intracranial infections after craniotomy. However, there was no significant difference between the above four antibacterial drugs in the indirect comparison. | No |
| Carlson J.D. et al., 2019 | [10.1016/j.wneu.2019.04.233](https://doi.org/10.1016/j.wneu.2019.04.233) | USA | Retrospective cohort | Adult | Functional (DBS generator replacement) | To analyse complication rates after generator replacements in end-stage Parkinson’s disease patients. |  | Yes | "Purulent discharge from a wound that required surgical treatment" | "Infections occurred in the perioperative period (<90 days) after the initial lead implantation (n = 6 of 232 cases, 2.5%) and generator replacement surgery (n = 1 of 172 cases, 0.6%)”. “Infections occurred in a delayed fashion, after either type of surgery (n = 11 of 404 cases, 2.7%). Delayed infections occurred an average of 7.6 ± 9.5 months after a previous operation.” | No |
| Catapano J. et al., 2019 | [10.1016/j.wneu.2019.07.183](https://doi.org/10.1016/j.wneu.2019.07.183) | USA | Retrospective cohort | Adult | Trauma | To report the external ventricular drain (EVD) associated infection rate among patients with aneurysmal subarachnoid hemorrhage managed with a unique standardised treatment protocol without an occlusive EVD dressing. |  | Yes | An EVD-related infection was defined as a “CSF culture with positive results”. | A total of 347 CSF studies were performed with no EVD-associated infections. There were 3 CSF samples with false-positive Gram stain results but no growth on concurrent or multiple repeat cultures. | No |
| Cater D.T. et al., 2021 | [10.3171/2021.10.PEDS21291](https://doi.org/10.3171/2021.10.peds21291) | USA | Retrospective cohort | Paediatric | Paediatric | To determine the association of postoperative dexmedetomidine with markers of pain in children undergoing Chiari malformation decompression. |  | No | Not defined | There was no difference between cohorts in the rate of postoperative complications, including pseudomeningocele formation, surgical site infection, aseptic meningitis, hydrocephalus, CSF leak and the need for reoperation. | No |
| Chacon-Quesada T. et al., 2021 | [10.1007/s10143-021-01513-5](https://doi.org/10.1007/s10143-021-01513-5) | Germany | Retrospective cohort | Adults and Paediatrics | General neurosurgical (all cases) | To analyse the rate of SSIs after the beginning of COVID-19 hygiene measures. |  | Yes | Not defined | The SSI rate was 3.6% (03/2019–09/2019, 50 cases of SSIs) and 2.2% (09/2019–03/2020, 29 cases of SSIs), resulting in a mean of 2.9% before COVID-19 began. After the beginning of COVID-19 hygiene measures, this rate dropped to 1.4% (16 cases of SSIs). This resembles a significant reduction of SSI rates (p=0.003). | No |
| Chaichana K.L. et al., 2015 | [10.1179/1743132815Y.0000000042](https://doi.org/10.1179/1743132815y.0000000042) | USA | Retrospective cohort | Adult | Neuro-oncology | The goals of this study were to calculate the incidence of postoperative infection, evaluate if carmustine wafers changes the risk of infection and identify factors independently associated with an infection following GBM surgery. |  | Yes | CDC guidelines | Four hundred and one patients underwent resection of an intracranial GBM during the reviewed period, and 21 (5%) patients developed an infection at a median time of 40 [28–286] days following surgery. The incidence of infection was not higher in patients who had carmustine wafers, and this remained true in multivariate analyses to account for differences in treatment cohorts. The factors that remained significantly associated with an increased risk of infection were prior surgery, diabetes mellitus, and increasing duration of hospital stay, where the greatest risk occurred with hospital stays w5 days. | No |
| Champeaux C. et al., 2020 | [10.1007/s11060-020-03410-1](https://doi.org/10.1007/s11060-020-03410-1) | France | Retrospective cohort | Adult | Neuro-oncology | To describe the epidemiology of carmustine wafer implantation, search for related complications, long-term survival and associated prognostic factors. |  | No | Not defined | 121 patients out of 1659 developed an SSI. | No |
| Chang S.M. et al., 2003 | [10.3171/jns.2003.98.6.1175](https://doi.org/10.3171/jns.2003.98.6.1175) | USA | Retrospective cohort | Adult | Neuro-oncology | To describe the medical and neurological complications associated with first and second craniotomies for patients with malignant gliomas. |  | No | Not defined | There were no significant differences between the two groups with respect to perioperative rates of wound infection. | No |
| Chaturvedi D. et al., 2019 | 10.1055/s-0039-1680276 | India | Retrospective cohort | Adults and Paediatrics | General neurosurgical (Craniotomy) | To investigate the rate of healthcare associated infections in the neurosurgical ICU. |  | Yes | Not defined | Reported that 2 patients experienced SSI. | No |
| Cheah PP et al., 2017 | [10.21315/mjms2017.24.6.8](https://doi.org/10.21315/mjms2017.24.6.8) | Malaysia | Prospective case-control | Adult | Trauma | To investigate surgical outcomes, specifically SSI, associated with two bone flap preservation methods, frozen versus subcutaneous pockets, as well as other risk factors, such as the timing of the cranioplasty, the indication and types of decompressive craniotomy (unilateral versus bifrontal), the status of the operating surgeon and the number of repeated surgeries before cranioplasty. | Comparison between two bone flap preservation methods: frozen vs subcutaneous pockets | Yes | CDC guidelines | Among the 12 early cranioplasty cases, two (16.7%) were found to have post-cranioplasty SSI. Of the 89 cases in the late cranioplasty group, only four cases of infection (4.5%) were found. | No |
| Chen C. et al., 2016 | 10.1097/MD.0000000000004329 | Taiwan | Retrospective case-control | Adult | Craniotomy | To determine (i) a risk assessment of post-craniotomy meningitis (PCM) and (ii) the current pattern of its infectious agents in our healthcare system. |  | Yes | CDC guidelines/ National Healthcare Safety Network Surveillance Definitions | Seven of 22 PCM patients did not meet the criteria of SSI because they had open wounds on admission. Of the 22 patients with PCM, SSI occurred in 15 patients (68.2%), | No |
| Chen H. et al., 2023 | [10.1007/s00586-023-07729-x](https://doi.org/10.1007/s00586-023-07729-x) | China | Retrospective cohort | Adult | Skullbase | To investigate the incidences, causes, and risk factors for unplanned reoperation within 30 days of craniovertebral junction (CVJ) surgery |  | Yes | Not defined | 14 out of 34 patients underwent unplanned reoperation because of wound infection. | No |
| Chen P et al., 2021 | [10.1155/2021/4948664](https://doi.org/10.1155/2021/4948664) | Papua New Guinea | Retrospective cohort | Adult | Trauma | To evaluate the clinical characteristics and prognosis of traumatic brain injury (TBI) patients from 2016 to 2019 admitted to Port Moresby General Hospital of Papua New Guinea and compare the results with previous researches to analyse current clinical characteristics and prognosis. |  | Yes | Not defined | Postoperative infection happened in 23 (10.2%) patients. | No |
| Chen R. et al., 2023 | [10.1227/neu.0000000000002376](https://doi.org/10.1227/neu.0000000000002376) | China | Retrospective cohort | Adult | General neurosurgical (Cranioplasty) | To analyse the independent risk factors of complications after cranioplasty and elucidate the potential correlation between brain collapse volume and prognosis. |  | Yes | “an incision or intracranial infection with symptoms or physical signs.” | Four total cases of SSI (3.9%) reported. | No |
| Chen Y. et al., 2018 | [10.1016/j.wneu.2018.01.211](https://doi.org/10.1016/j.wneu.2018.01.211) | USA | Retrospective cohort | Adult | Neuro-oncology | To assess glioblastoma reoperation trends nationally in older patients, with emphasis on outcomes. |  | Yes | Not defined | Wound infection occurred in 37 patients (8%) that received one reoperation. Wound infection occurred more frequently in the 1 reoperation cohort as compared to the no reoperation cohort. | No |
| Cheng C.H. et al., 2014 | [10.1016/j.clineuro.2014.06.029](https://doi.org/10.1016/j.clineuro.2014.06.029) | China | Retrospective case-control | Adults and Paediatrics | Trauma | To clarify whether differences in the methods used to store bone flaps influence the incidences of SSI and bone flap resorption following cranioplasty. |  | Yes | Defined by presence of focal erythema, pus-like discharge or wound rupture | 18.2% in cryopreservation group and 11.1% in subcutaneous pocket group developed SSI. | No |
| Chiang H.Y. et al., 2011 | [10.3171/2011.1.JNS10782](https://doi.org/10.3171/2011.1.jns10782) | USA | Prospective cohort | Adult | General neurosurgical (Craniotomy) | To identify the prevalence of bone flaps with positive cultures, to assess the risk of SSI after reimplanting bone flaps with positive cultures, and to identify risk factors for SSI following the initial craniotomies or craniectomies. |  | Yes | CDC guidelines | 21 patients acquired 22 SSIs (5.8%) (1 patient acquired an SSI after each of 2 procedures). Twenty patients (5.4% of all 373 patients and 95.2% of those 21 with SSI) acquired SSIs after their index procedures. | No |
| Chiang H.Y. et al. 2014 | [10.3171/2013.9.JNS13843](https://doi.org/10.3171/2013.9.jns13843) | USA | Retrospective case-control | Adult | General neurosurgical (Craniotomy/ craniectomy) | To identify risk factors for SSIs after cranial procedures and to evaluate outcomes attributed to SSIs |  | Yes | Not defined | 32% of SSIs were caused by Staphylococcus aureus, 88% were deep incisional or organ space infections, and 70% were identified after discharge. | No |
| Chibbaro S. et al., 2011 | [10.1016/j.wneu.2010.10.020](https://doi.org/10.1016/j.wneu.2010.10.020) | France and USA | Prospective cohort | Adult | Trauma | To evaluate the efficacy of the immediate surgical intervention coupled with early cranial reconstruction to achieve a better survival chance and functional prognosis. |  | No | Not defined | Superficial wound infection occurred in 9 patients (in 3 after craniectomy and in the remaining 6 after cranioplasty). None required surgery and successfully managed by intravenous antibiotics. | No |
| Cho J. et al., 2003 | [10.1227/01.neu.0000054219.35102.b4](https://doi.org/10.1227/01.neu.0000054219.35102.b4) | USA | Retrospective case-control | Adults and Paediatrics | Neuro-oncology | To review the authors’ experience with  computer image guidance, linear or sigmoid incisions after minimal shaving, and liquid wound  dressing with 2-octyl cyanoacrylate for tumour craniotomy or craniectomy in our attempt to optimise craniotomy or craniectomy for tumour. | Technically enhanced methods vs traditional methods. | Yes | Not defined | Reported two infections within 2 weeks of initial craniotomy in the study group and 14 wound complications in the control group. Reported eight early wound complications, including six infections and two cases of wound dehiscence, and six late complications, including three wound infections. | No |
| Choque-Velasquez J. et al., 2020 | [10.1016/j.wneu.2020.01.137](https://doi.org/10.1016/j.wneu.2020.01.137) | Finland | Retrospective case-control | Adults and Paediatrics | Skullbase | To establish the better performance of the paramedian supracerebellar infratentorial approach in terms of clinical safety in surgically treated pineal cysts and pineal region tumours. |  | No | Not defined | Wound infections in the midline approach group were more serious with two patients undergoing surgical removal of the bone flap. Two patients with preliminary shunt surgery that underwent a midline approach many days later developed shunt infections after the approach. | No |
| Chotai S. et al., 2023 | [10.3171/2022.4.JNS22290](https://doi.org/10.3171/2022.4.jns22290) | USA | Retrospective cohort | Adults and Paediatrics | Neuro-oncology | To determine outcomes following various dural closure techniques for supratentorial meningiomas |  | Yes | Not defined | Reported 11 SSIs in sutured dural repair and 5 in non-sutured repair. | No |
| Clark A.J. et al., 2011 | [10.3171/2010.10.JNS101042](https://doi.org/10.3171/2010.10.jns101042) | USA | Retrospective case-control | Adult | Neuro-oncology | To determine if antiangiogenic treatment was associated with increased rate of wound-healing complications. | Use of preoperative bevacizumab vs no bevacizumab used vs postoperative bevacizumab used. | Yes | Not defined | Wound infection was the most common wound-healing complication (26 cases). | No |
| Clune J.E. et al., 2010 | [10.1097/SCS.0b013e3181cf6103](https://doi.org/10.1097/scs.0b013e3181cf6103) | USA | Retrospective cohort | Paediatric | Paediatric | To determine if perioperative corticosteroid shortens hospital stay after fronto-orbital advancement. | Corticosteroid use vs no corticosteroid use. | Yes | Not defined | Infection rates did not differ between groups. | No |
| Cohen-Inbar O. et al., 2014 | [10.1055/s-0034-1371516](https://doi.org/10.1055/s-0034-1371516) | Israel | Retrospective case-control | Adults and Paediatrics | CSF dynamics | To evaluate the effect of laparoscopic implantation of distal peritoneal ventriculoperitoneal shunt catheter will have on complications incidence and patient's prognosis. | Laparoscopic vs mini laparotomy approach | Yes | Not defined | The number of shunt operations and revisions has a direct influence on the likelihood of developing a surgical wound infection. In patients who underwent two prior operations, the risk of infection is increased by the factor of 4.61. | No |
| Colombo F. et al., 2023 | [10.1055/s-0043-1774720](https://doi.org/10.1055/s-0043-1774720) | UK | Retrospective cohort | Adult | General neurosurgical (Craniotomy) | To clarify which perioperative measures play a role in reducing surgical infection rates further. |  | Yes | Defined as per Section 3 of the Protocol for Surveillance of SSI published by Public Health England. | Postoperative infections were observed in 5.2% of cases. No postoperative infections occurred within 4 months in patients receiving perioperative hair wash and intrawound vancomycin powder. Craniotomy size, lack of perioperative hair wash, and vancomycin powder use were predictive of postoperative infection. | No |
| Cosgrove G.R. et al., 2007 | [10.3171/jns.2007.106.1.52](https://doi.org/10.3171/jns.2007.106.1.52) | USA | Prospective cohort | Adult | Other (Cranial surgery with dural repair) | To evaluate the safety and efficacy of a novel polyethylene glycol (PEG) hydrogel sealant in patients undergoing elective cranial surgery with documented cerebrospinal fluid (CSF) leakage after sutured dural repair. |  | Yes | CDC guidelines | Deep SSIs occurred in 7.2% of cases. The volume of sealant, duration of surgery, length of durotomy, use of intraoperative shunts or drains, and smoking status were associated with deep SSIs. | No |
| Cote D. et al., 2016 | [10.1055/s-0036-1592306](https://doi.org/10.1055/s-0036-1592306) | USA | Retrospective cohort | Adult | Neuro-oncology | Using the National Surgical Quality Improvement Program (NSQIP) registry, patients undergoing craniotomy for brain tumour from 2006 and 2014 were analysed to identify risk factors for postoperative VTE. | . | No | Not defined | Wound infection occurred in 1% of cases. Superficial SSI occurred in 0.6%, deep SSI in 0.5% and organ SSI in 1% of cases. | No |
| Cote D.J. et al., 2019 | [10.1016/j.wneu.2019.05.022](https://doi.org/10.1016/j.wneu.2019.05.022) | USA | Retrospective cohort | Adults and Paediatrics | Vascular | To determine the incidence and risk factors for adverse events after microvascular decompression. |  | Yes | Not defined | 1.7% incidence of SSI reported. | No |
| Coulter I.C. et al., 2014 | [10.1007/s00701-014-2081-1](https://doi.org/10.1007/s00701-014-2081-1) | UK | Retrospective cohort | Adult | General neurosurgical (Cranioplasty) | To evaluate the morbidity associated with cranioplasty and investigate its potential effect on outcome. |  | Yes | Infection was defined by the need for antibiotics and the need to remove an infected implant during the period of follow-up. | Thirty-six patients (21.7%) developed infection requiring antibiotics, with 27 (16.3%) requiring removal of the cranioplasty. Nine of 25 patients (36%) with bi-frontal defects developed an infection whereas 21 of the 153 patients (16.4%) with a defect other than bi-frontal developed an infection. | No |
| Covell M.M. et al., 2023 | [10.1016/j.ejso.2023.107044](https://doi.org/10.1016/j.ejso.2023.107044) | USA | Retrospective cohort | Adult | Neuro-oncology | To evaluate the predictive accuracy of preoperative lab values on postoperative metastatic brain tumour resection outcomes using data queried from a large prospective international surgical registry, representing over 700 hospitals in 11 countries. |  | Yes | Not defined | Reported 20 superficial, 18 deep incisional, and 53 organ space infections. | No |
| Das K.K. et al., 2020 | [10.1016/j.wneu.2020.05.259](https://doi.org/10.1016/j.wneu.2020.05.259) | India | Retrospective cohort | Adult | Neuro-oncology | To better understand the clinical behaviour, radiological features, and surgical outcomes of interhemispheric epidermoid tumours. |  | No | Not defined | Reported 1 case of wound infection. | No |
| Dasenbrock H. et al., 2017 | [10.3171/2016.2.PEDS15604](https://doi.org/10.3171/2016.2.peds15604) | USA | Retrospective cohort | Adult | Neuro-oncology | To evaluate the rate of, reasons for, and predictors of unplanned reoperation after craniotomy for tumour in a nationally accrued population. | . | No | Not defined | The most common reasons for cranial reoperation were intracranial hematoma evacuation (22.5%), superficial or intracranial surgical site infections (11.9%), re- resection of tumor (8.4%), decompressive craniectomy (6.1%), and repair of cerebrospinal fluid leakage (5.6%). Dependent functional status, morbid obesity, leukocytosis, and longer operative time were predictors of reoperation for infection. | No |
| Dasenbrock H. et al., 2017 | [10.3171/2016.2.JNS152345](https://doi.org/10.3171/2016.2.jns152345) | USA | Retrospective cohort | Adults and Paediatrics | Neuro-oncology | To use a national registry to evaluate the association of body mass index (BMI) and hypoalbuminemia with 30-day outcomes after craniotomy for tumour. |  | Yes | Not defined | Reported significantly increased odds of SSI in those with Class II or III obesity. | No |
| Dasenbrock H. et al., 2016 | [10.1093/neuros/nyw062](https://doi.org/10.1093/neuros/nyw062) | USA | Retrospective cohort | Adult | Neuro-oncology | To utilise the prospective National Surgical Quality Improvement Program 2011-2013 registry to evaluate the predictors of unplanned 30-d readmission and post discharge mortality after cranial tumour resection. |  | Yes | Not defined | The most common reasons for readmission were SSI (17.0%), infectious complications (11.0%), venous thromboembolism (10.0%), and seizures (9.4%). | No |
| Dasenbrock H. et al., 2017 | [10.1161/STROKEAHA.117.016702](https://doi.org/10.1161/strokeaha.117.016702) | USA | Retrospective cohort | Adult | Vascular | To evaluate the suitability of readmission as a quality indicator in the aneurysmal subarachnoid hemorrhage (SAH) population. |  | No | Not defined | Reported 5 cases of SSI in clipping and 2 cases of SSI in coiling group. | No |
| Dasenbrock H. et al., 2017 | [10.1093/neuros/nyx089](https://doi.org/10.1093/neuros/nyx089) | USA | Retrospective cohort | Adult | Neuro-oncology | To evaluate the rate of, reasons for, and predictors of unplanned reoperation after craniotomy for tumour in a nationally accrued population. |  | Yes | Not defined | The second most common cranial reoperation was SSIs (11.9% of cranial reoperations 0.6% of patients, n = 68), of which the majority (n = 36, 52.9%) were debridement of superficial infections, while the others (n = 30, 44.2%) comprised drainage of an intracranial abscess, and a minority were unspecified (n = 2, 2.9%). | No |
| Davies B.M. et al., 2016 | [10.1308/rcsann.2016.0143](https://doi.org/10.1308/rcsann.2016.0143) | UK | Retrospective cohort | Adult | General neurosurgical (All neurosurgical cases) | To assess whether preoperative skin preparation using a combination of chlorhexidine and povidone-iodine preoperative antisepsis was associated with a lower SSI rate than either agent alone in clean cranial neurosurgery. | Preoperative use of chlorhexidine or povidone-iodine as a single agent, vs a combination of the two agents | Yes | UK Health Protection Agency guidance | 94 out of 2603 cases (3.6%) were complicated by SSI.  Longer operation times and younger patient age were significantly associated with the occurrence of SSIs. | No |
| Davies B.M. et al., 2015 | [10.3109/02688697.2015.1071321](https://doi.org/10.3109/02688697.2015.1071321) | UK | Retrospective cohort | Not reported | General neurosurgical (All cranial neurosurgical cases) | To review the findings following the introduction of the Public Health England methodology for cranial neurosurgery and consider its appropriateness. |  | Yes | Public Health England Guidelines | 82 (3.5%) patients had a confirmed SSI. | No |
| Dechaene V. et al., 2023 | [10.1016/j.ijid.2023.10.008](https://doi.org/10.1016/j.ijid.2023.10.008) | France | Retrospective cohort | Adult | General neurosurgical (Craniotomy) | To assess clinical and microbiological features, management, and outcome of bone flap-related osteomyelitis after cranioplasty. |  | Yes | Not defined | Most patients had early surgical site infection (n = 78, 54.2%), mainly presenting as wound abnormalities (n = 115, 79.9%). | No |
| Delgado-Lopez P.D. et al., 2009 | [10.1016/s1130-1473(09)70154-x](https://doi.org/10.1016/s1130-1473(09)70154-x) | Spain | Retrospective cohort | Adult | Trauma | To discuss the effectiveness and safety of corticotherapy in Chronic Subdural Haematology. |  | No | Not defined | 2-7% of patients developed a superficial wound infection. | No |
| DeLong MR. et al., 2014 | [10.1001/jamaneurol.2014.1272](https://doi.org/10.1001/jamaneurol.2014.1272) | USA | Retrospective cohort | Adult | Functional (DBS insertion) | To describe the risk of adverse outcomes among older patients undergoing DBS insertion for Parkinson’s Disease and to assess the postoperative complications. |  | Yes | Not defined | Wound infection was observed in 64 patients (3.6%). | No |
| Di L. et al., 2023 | [10.3171/2022.3.JNS212399](https://doi.org/10.3171/2022.3.jns212399) | USA | Retrospective cohort | Adult | Neuro-oncology | To compare survival outcomes for patients with left-sided eloquent newly diagnosed glioblastoma undergoing supramximal resection versus gross-total resection. |  | No | Not defined | One patient exhibited a postoperative wound infection. | No |
| Dickinson H. et al., 2015 | [10.3171/2014.8.JNS1498](https://doi.org/10.3171/2014.8.jns1498) | USA | Retrospective cohort | Adult | Neuro-oncology | This study 1) delineates reasons for readmission, 2) explores factors associated with readmissions, and 3) describes their impact on the survival of glioblastoma patients. | . | Yes | Not defined | Of the 8 patients readmitted for infectious reasons, 3 were readmitted for SSI | No |
| Dinevski N. et al., 2017 | [10.1016/j.wneu.2017.03.093](https://doi.org/10.1016/j.wneu.2017.03.093) | Switzerland | Retrospective cohort | Adult | General neurosurgical (all neurosurgical procedures) | To determine the rate of SSI in neurosurgical procedures involving a intraoperative MRI scanner. |  | Yes | CDC guidelines – with an altered follow-up period of 90 days | 6 out of 109 craniotomy patients developed an SSI (5.5%) with 1 superficial SSI, 2 cases of bone flap osteitis, 1 intracranial abscess and 2 cases of meningitis/ ventriculitis. 2 patients out of the 86 transsphenoidal skull base surgeries developed non-CNS intranasal SSIs (3%) and 4 developed meningitis (5%). | No |
| Donnelly BM et al., 2023 | [10.1007/s00701-023-05764-7](https://doi.org/10.1007/s00701-023-05764-7) | USA | Retrospective cohort | Adults and Paediatrics | Trauma | To report the outcomes of the contents of the medical center’s bone bank freezer: culture data, reasons for discardment of flaps, and infection rates after reimplantation. |  | Yes | Not defined | Postcranioplasty infections were seen in 3 (12%) patients who had reimplantation of their flap. | No |
| Doshi P., 2011 | [10.1159/000323372](https://doi.org/10.1159/000323372) | India | Retrospective cohort | Adult | Functional (DBS insertion) | To evaluate the incidence of surgical and hardware-associated complications of deep brain stimulation (DBS) for a range of movement disorders. |  | Yes | Not defined | 7 patients developed forms of infection or erosion-related complications. 1 patient had a wound breakdown over the connector site leading to infection, necessitating explantation of the system. | No |
| Dowlati E et al., 2022 | [10.3171/2022.3.JNS212637](https://doi.org/10.3171/2022.3.jns212637) | USA | Retrospective case-control | Adult | General neurosurgical (Craniotomy) | To compare in-hospital and surgical site complication rates in patients who had bone flaps preserved in the abdominal subcutaneous tissue (SQ) versus those whose bone flaps were discarded. | Bone flaps implanted in the abdominal wall pocket vs those with their bone flaps discarded | Yes | Not defined | 12 patients with SSI reported in the SQ group, 8 patients with SSI reported in the discarded group. | No |
| Drexler R. et al., 2024 | [10.1227/neu.0000000000002689](https://doi.org/10.1227/neu.0000000000002689) | Multiple | Retrospective cohort | Adult | Vascular | To define standardised outcome benchmarks in patients who underwent clipping of unruptured intracranial aneurysm. |  | Yes | Not defined | 0% rate of SSI amongst low-risk group, and 6.8% rate of SSI among high-risk cohort. | No |
| Ebel F. et al., 2022 | [10.3390/diagnostics12123045](https://doi.org/10.3390/diagnostics12123045) | Switzerland | Retrospective cohort | Adult | Trauma | To assess the influence of drainage suction in the surgical treatment of chronic subdural haemorrhage on the recurrence rate. |  | Yes | Not defined | Surgical infections were significantly higher in the passive than in the active drain group. | No |
| Eichberg D. et al., 2018 | [10.1080/02688697.2018.1490943](https://doi.org/10.1080/02688697.2018.1490943) | USA | Retrospective cohort | Adult | Other (Dural closure) | To present the institution’s experience using dehydrated amniotic membrane allograft during dural closure in 155 patients. | . | No | Not defined | Reported 1 case of superficial wound infection requiring washout without craniectomy. | No |
| Ellens N.R. et al., 2019 | [0.1093/neuros/nyy090](https://doi.org/10.1093/neuros/nyy090) | USA | Retrospective cohort | Adults and Paediatrics | Trauma | To assess the accuracy and complication rates of midlevel practitioners and neurosurgeon EVD placement. |  | Yes | Infection defined by positive CSF culture | No significant difference in infection rate between midlevel practitioners and neurosurgeon EVD placement. | No |
| Elliott R.E. et al., 2013 | [10.3171/2013.4.JNS121829](https://doi.org/10.3171/2013.4.jns121829) | USA | Retrospective cohort | Adult | General (Craniotomy) | To identify preoperative variables that predict long-term seizure freedom among patients with mesial temporal sclerosis after single-stage anterior temporal lobectomy and amygdalohippocampectomy. |  | No | Not defined | 1 patient had an infection requiring bone flap removal. | No |
| Elsamadicy A.A. et al., 2018 | [10.1016/j.jocn.2017.09.021](https://doi.org/10.1016/j.jocn.2017.09.021) | USA | Retrospective cohort | Adults and Paediatrics | General (Craniotomy) | To identify the drivers of 30-day unplanned readmission in consecutive patients undergoing craniotomies and craniectomies. |  | Yes | Not defined | 4.9% wound infection reported. | No |
| Elward A. et al., 2015 | [10.1097/INF.0000000000000889](https://doi.org/10.1097/inf.0000000000000889) | USA | Retrospective cohort | Paediatrics | Paediatrics | The aim of this study is to determine risk factors for SSI among paediatric patients undergoing craniotomy and spinal fusion. |  | Yes | Centers for Disease Control and Prevention/ National Healthcare Safety Network Surveillance definitions | Previous craniotomy, longer procedure duration, longer interval between antibiotic administration and incision and a longer interval to antibiotic redosing were associated with an increased risk of craniotomy SSI. | No |
| Engelhardt M. et al., 2005 | [10.1055/s-2005-836476](https://doi.org/10.1055/s-2005-836476) | Germany | Prospective cohort | Adults and Paediatrics | General (Craniotomy) | To analyse predisposing factors for dural tears during trepanation in order to optimise the design of a robot-assisted trepanation system. |  | No | Not defined | Postoperative cerebral fluid leakage was seen in two patients, wound infections occurred in three patients. | No |
| Englot D.J. et al., 2014 | [10.3171/2014.7.PEDS13658](https://doi.org/10.3171/2014.7.peds13658) | USA | Retrospective cohort | Paediatric | Paediatric | To analyse factors associated with persistent seizures after surgery to better understand the reasons for surgical failures in this population, and to help guide treatment strategies going forward. |  | No | Not defined | 2 wound infections occurred out of 115 surgeries. | No |
| Ening G. et al., 2015 | [10.1016/j.clineuro.2015.01.006](https://doi.org/10.1016/j.clineuro.2015.01.006) | Germany | Retrospective cohort | Adult | Neuro-oncology | To assess treatment associated complications, evaluating the impact on survival and defining risk factors. |  | No | Not defined | Reported 10 wound infections in total. | No |
| Estes E.M. et al., 2023 | [10.1016/j.clineuro.2023.107864](https://doi.org/10.1016/j.clineuro.2023.107864) | USA | Retrospective cohort | Adult | Skullbase | To use a large national registry to evaluate the effect of frailty on postoperative outcomes of patients undergoing epilepsy surgery. |  | Yes | Not defined | 18 cases of SSI observed. | No |
| Falowski S. et al., 2012 | [10.1159/000338254](https://doi.org/10.1159/000338254) | USA | Retrospective cohort | Adult | Functional (DBS insertion) | To analyse hardware complications based on patient diagnosis and lead location. |  | Yes | Not defined | 1.9% infection rate reported. | No |
| Falowski S.M. et al., 2015 | [10.1016/j.wneu.2015.01.018](https://doi.org/10.1016/j.wneu.2015.01.018) | USA | Retrospective cohort | Adults and Paediatrics | Functional (electrode placement in epilepsy) | To assess the potential contribution of specific aspects of surgical technique to the reduction of complication rates. | . | Yes | Not defined | Reported one case of infection (0.8%). The infection rate was lower than rates reported for neurologic tissue infections (0% vs. 2.3%) and for superficial infections (0.8% vs. 3.0%) in the meta-analysis. | No |
| Fan M.C. et al., 2018 | [10.1016/j.wneu.2017.10.112](https://doi.org/10.1016/j.wneu.2017.10.112) | China | Retrospective cohort | Adults and Paediatrics | Trauma | To clarify the clinical outcomes of cranioplasty with cryopreserved bone flaps and identify risk factors related to bone flap infection and resorption after cranioplasty with cryopreserved bone flaps. |  | Yes | 1. Any case in which infection was suspected and antibiotics therapy was administered  2. Requirement of reoperation to remove graft. | The bone graft infection rate was greater in emergency craniectomy cases (8.81% vs. 2.59%) and in patients with diabetes (10.53% vs. 3.07%). | No |
| Farber S.H. et al., 2011 | [10.1227/NEU.0b013e31821bc435](https://doi.org/10.1227/neu.0b013e31821bc435) | USA | Retrospective cohort | Adult | CSF dynamics | To determine whether a categorical conversion to antibiotic impregnated shunt (AIS) shunt systems reduced the incidence of shunt infection in a series of entirely adult patients | AIS catheters vs non-AIS catheters | Yes | Clinical suspicion of shunt infection (fever, change in mental status, increased white blood cell count, signs of meningismus), the presence of a CSF profile (leukocytosis, positive CSF bacterial cultures) | Overall, 13 patients (2.6%) experienced CSF shunt infection. | No |
| Farber S.H. et al., 2010 | [10.1016/j.wneu.2010.07.014](https://doi.org/10.1016/j.wneu.2010.07.014) | USA | Retrospective case-control | Adult | CSF dynamics | To determine if use of antibiotic-impregnated shunt (AIS) systems to reduce cerebrospinal fluid (CSF) shunt infections in adult patients with hydrocephalus has been cost-effective at one institution. | AIS catheters vs non-AIS catheters | Yes | Not defined | The incidence of shunt infection was decreased in the AIS (1.2%) vs non-AIS (4.0%) cohorts. | No |
| Farrokhi F.R. et al., 2019 | [10.1016/j.jocn.2019.08.026](https://doi.org/10.1016/j.jocn.2019.08.026) | USA | Retrospective cohort | Adult | Functional (DBS insertion) | To explore possible associations between common preoperative clinical risk factors and complications, including unplanned return to the operating room. |  | Yes | 1) Requiring surgical removal of DBS hardware  2) Requiring medical treatment for infection | Patients who developed a surgical site infection were more likely to report history of smoking before DBS surgery (16% vs 5%). | No |
| Fattahi A. et al., 2018 | [10.1080/02688697.2018.1476673](https://doi.org/10.1080/02688697.2018.1476673) | Iran | Retrospective cohort | Adults and Paediatrics | General (All neurosurgical cases) | To introduce our routine protocol of surgical site infection (SSI) prophylaxis. |  | Yes | Not defined | There were no reports of SSI. | No |
| Fenoy A.J. et al., 2012 | [10.3171/2012.1.JNS111798](https://doi.org/10.3171/2012.1.jns111798) | USA | Retrospective cohort | Both | Functional (DBS insertion) | To review the incidence and management of all hardware-related wound dehiscences and infections in a large patient series. |  | Yes | 1. Within 12 months of an original implantation  2. Superficial wound infections: induration, redness, persistent crusting over a hardware component, cellulitis,  purulent drainage.  3. requiring surgical revision  4. cultures from hardware or from fluid in contact with hardware. | The rate presentation of infection or erosion occurring under 12 months from DBS implantation, requiring additional surgery, was 1.24% (9/728) per patient. The only variables that were significantly different between self-limited infections and those requiring reoperation were the time after implantation to clinical presentation, initial antibiotic choice. | Yes: However, interpretation of these rates is difficult because the definition of infection is variable and is sometimes  inclusive and/or exclusive of erosions and/or dehiscences  without infection or infections that are self-limited. |
| Fenoy A.J. et al., 2014 | [10.3171/2013.10.JNS131225](https://doi.org/10.3171/2013.10.jns131225) | USA | Retrospective cohort | Adults and Paediatrics | Functional (DBS insertion) | To assess the incidence of various surgical complications occurring both during and after DBS device implantation for movement disorders in an effort to better quantify patient risk, define management plans, and develop methods for risk avoidance. To corroborate the low procedural complication risk of DBS reported by others. |  | No | Not defined | Among wound complications, infection was the most common, occurring in a total of 23 cases (3.1%), 10 (1.4%) of which were self-limited and 13 (1.7%) of which required a return to surgery for debridement and/or device removal. | Yes: The risk of infectious complications has been reported  to range from 0% to 15% in various studies a rate unfortunately marred by the lack  of standardised definition. |
| Fernandez C.S. et al., 2022 | [10.5603/PJNNS.a2022.0030](https://doi.org/10.5603/pjnns.a2022.0030) | Spain | Retrospective cohort | Adults and Paediatrics | Trauma | To analyse the association between the number of burr-holes and the clinical outcome of patients. To discuss the main complications related to surgical site infections, recurrences and acute rebleedings that require reintervention. |  | Yes | Not defined | SSI occurred in 1.73% of surgeries with 1 burr hole, and in 6.25% of surgeries with 2 burr holes. | No |
| Ferreira de Andrade A. et al., 2020 | PMCID: PMC7364413 | Brazil | Prospective cohort | Adult | Trauma | To analyse a series of patients underwent to surgical treatment for acute subdural hematoma (ASDH). |  | No | Not defined | Reported complications in 5 patients: 2 surgical site infections (with removal of bone flap). | No |
| Fialkov J.A. et al., 2001 | [10.1097/00001665-200107000-00009](https://doi.org/10.1097/00001665-200107000-00009) | Canada | Retrospective cross-sectional | Adult | Other: Craniofacial surgery | To assess the rate of, and possible risk factors for, postoperative craniofacial infection. |  | Yes | Clinical diagnosis (two or more signs of local infection: redness, swelling, purulent discharge) plus one or more of the following: 1. Hospital admission for treatment of infection. 2. Intravenous or oral antibiotic treatment. 3. Surgical intervention for drainage, irrigation, and/or debridement. 4. Microbiological confirmation (pathogenic organism present in 10>5 cfu/ml). | Reported 23 out of 280 total cases of postoperative infection (8.2%). The most common site for postoperative infection was the mandible (infection rate = 16.7%). | No |
| Filho N.O. et al., 2016 | [10.3171/2015.9.PEDS1559](https://doi.org/10.3171/2015.9.peds1559) | El Salvador | Retrospective cohort | Paediatric | Neuro-oncology | To determine whether the occurrence of hyperglycemia during the perioperative period of elective neurosurgery for the resection of tumours of the CNS in children is associated with increased morbidity. |  | Yes | Not defined | Main complication observed was infection, which occurred in 16 patients (15.2%). | No |
| Findlay M.C. et al., 2023 | [10.1227/neu.0000000000002397](https://doi.org/10.1227/neu.0000000000002397) | USA | Retrospective cohort | Adult | Skullbase | To determine whether race predicts worse outcomes after anterior cranial fossa surgery. |  | Yes | Not defined | Reported 5 cases of infection in white people vs 7 cases in minority groups. | No |
| Flanagan L.S. et al., 2022 | [10.1002/lary.29893](https://doi.org/10.1002/lary.29893) | USA | Retrospective cohort | Both | Skullbase | To investigate the impact of preoperative hematocrit on complications following ventral skull base surgery. |  | Yes | Not defined | 8.1% infections in non-anaemic patients and 16.7% in anaemic patients. | No |
| Forcadas-Berdusan M et al. 2011 | [10.1684/epd.2011.0413](https://doi.org/10.1684/epd.2011.0413) | Spain | Retrospective cohort | Adults and Paediatrics | Functional (temporal lobe resection for epilepsy) | To investigate the outcome of temporal lobe epilepsy surgery and identify the variables which predict a good prognosis with respect to seizures in postoperative follow-up after two and four years. |  | Yes | Not defined | Reported 15 cases (13%) of CNS infection post-operatively and 1 case of infection following craniectomy. | No |
| Foster K.A. et al., 2017 | [10.1016/j.wneu.2016.02.071](https://doi.org/10.1016/j.wneu.2016.02.071) | USA | Retrospective case-control | Adults and Paediatrics | Trauma | To compare complication rates of calcium phosphate (CaP) cement and titanium mesh cranioplasty in patients undergoing retromastoid craniectomy. | CaP cement vs titanium mesh cranioplasty. | Yes | Not defined | Reported 0.6% experienced wound infection. | No |
| Foster M. et al., 2021 | [10.3171/2020.9.PEDS20556](https://doi.org/10.3171/2020.9.peds20556) | UK | Retrospective case control | Paediatric | Paediatric neuro-oncology | To quantify surgical morbidity after paediatric brain tumour surgery using existing morbidity and outcome measures. |  | Yes | Not defined | Reported 9 cases (2%) of wound infection. 7 cases of shunt infection. | No |
| Frizon L.A. et al., 2017 | [10.1111/ner.12605](https://doi.org/10.1111/ner.12605) | USA | Retrospective cohort | Adult | Functional (DBS insertion) | To establish the infection rate at the initial surgery and for each subsequent replacement of DBS. |  | Yes | 1. Wound tenderness or purulent discharge  2. Elevated temperature or inflammatory markers  3. Evidence of hardware exposure at the pulse generator site (i.e., erosion) | For all 697 patients, the infection rate at the first surgery was 2.01%; at the second surgery, it was 0.44%; and at the third surgery, it was 1.83%. When considering only patients that underwent at least three replacement surgeries the infection rate did not change in a significant manner with subsequent interventions compared to the first replacement. | No |
| Fuentes A.M. et al., 2021 | [10.1016/j.clineuro.2021.106757](https://doi.org/10.1016/j.clineuro.2021.106757) | USA | Retrospective cohort | Adults and Paediatrics | Skullbase | To determine differences in inpatient complications and hospitalisation data among patients treated with the surgical techniques reported. |  | No | Not defined | Reported 2 patients had an infection after posterior fossa decompression, no infections when this was paired with a duraplasty. | No |
| Fujimoto Y. et al., 2008 | [10.1016/j.surneu.2007.06.091](https://doi.org/10.1016/j.surneu.2007.06.091) | Japan | Retrospective cohort | Adults and Paediatrics | General (Craniotomy) | To evaluate a hydrocolloid dressing for neurosurgical wounds according to the modern concept of wound healing. |  | Yes | Not defined | No SSI case reported. | No |
| Gadgil N. et al., 2018 | [10.1097/SCS.0000000000004654](https://doi.org/10.1097/scs.0000000000004654) | USA | Retrospective cohort | Paediatric | Paediatric/ Skullbase | To evaluate the predictions from the risk calculator compared to authors’ single institution experience in craniosynostosis surgery. |  | No | Not defined | 3 SSIs were observed | No |
| Garzon-Muvdi T et al. 2015 | [10.1227/NEU.0000000000000625](https://doi.org/10.1227/neu.0000000000000625) | USA | Retrospective cohort | Adult | General (Suboccipital craniectomy) | To determine whether the greater occipital nerve plays an important role in the development of postoperative headaches. |  | No | Not defined | By multivariate analysis, wound infection had statistically significant associations with the development of debilitating postoperative headaches. | No |
| Gazzeri R. et al., 2020 | [10.1016/j.clineuro.2020.105705](https://doi.org/10.1016/j.clineuro.2020.105705) | Italy | Retrospective case-control | Adult | Trauma | To evaluate the postoperative complications of the various surgical techniques of Chronic Subdural Haemorrhage. | Single burr hole with subdural drainage (Group Ia), single burr hole with subgaleal drainage (Group Ib), craniotomy with subdural drainage (Group IIa), and craniotomy with subgaleal drainage (Group IIb). | Yes | Not defined | 2.9% SSI rate reported. | No |
| George B. et al., 2017 | [10.1093/neuros/nyx024](https://doi.org/10.1093/neuros/nyx024) | France | Randomised control | Adult | Skullbase | To compare the efficacy and safety of adjunctive TachoSil with current practice for the prevention of postoperative CSF leaks in patients undergoing elective skull base surgery involving dura mater closure. |  | No | Not defined | Reported 7 SSIs in the TachoSil group compared to 9 in current practice. | No |
| Gil Z. et al., 2003 | [10.1067/mhn.2003.14](https://doi.org/10.1067/mhn.2003.14) | Israel | Retrospective | Adults and Paediatrics | Skull base | To evaluate surgical wound infection rates in patients undergoing skull base surgery without hair removal. | . | Yes | CDC guidelines | Surgical wound infection rate was 1.1% (2 cases of 175): 1 case for anterior and 1 case for lateral or posterior procedures. It was similar for clean operations (lateral and posterior) and clean-contaminated (anterior) procedures and was less than or similar to the rates reported for skull base procedures with hair removal. | No |
| Giovanni S. et al., 2014 | [10.1016/j.clineuro.2014.05.005](https://doi.org/10.1016/j.clineuro.2014.05.005) | Italy | Retrospective case-control | Adult | Neuro-oncology | To compare postoperative CSF-leak complication in patients undergoing duraplasty with galea-pericranium with sealants, galea-pericranium without sealants and synthetic patch duraplasty with sealants. | Galea-pericranium graft without sealant Vs Galea-pericranium graft with sealant Vs dural patch with sealant. | Yes | Not defined | Wound infection rate: galea-pericranium only group 0%, galea-pericranium + sealant group: 0% (0 of 92 patients), synthetic patch with sealant group 1.08% (1 of 92 patients). | No |
| Girgis F. et al., 2015 | [10.1017/cjn.2015.46](https://doi.org/10.1017/cjn.2015.46) | USA | Retrospective cohort | Adults and Paediatrics | General (Cranioplasty) | To determine if fever or leukocytosis at presentation were indicative of infection, as well as to identify any factors that may limit its applicability. |  | Yes | 1. Evidence of frank purulence as described in operative records  2. the presence of positive intra-operative cultures from a non-superficial source (e.g. subcutaneous tissue, bone, epidural, subdural, intracranial collections.) | In 27 total cases of surgical site infection, only two had a fever and four had leukocytosis at presentation. This yielded a false-negative rate for fever of 92.6% and for leukocytosis of 85.2%. 22 (81.5%) cases generated positive intra-operative cultures. Median interval to infection was 99 days from initial cranioplasty to time of infectious presentation. | No |
| Goel N.J. et al., 2018 | [10.1016/j.wneu.2018.06.153](https://doi.org/10.1016/j.wneu.2018.06.153) | USA | Retrospective cohort | Adults and Paediatrics | General (Craniotomy) | To assess the independent effect of complications on 30-day mortality in patients undergoing elective craniotomy. |  | Yes | Not defined | Reported 2% SSI rate. | No |
| Goldschlager T. et al., 2007 | [10.1016/j.jocn.2006.12.002](https://doi.org/10.1016/j.jocn.2006.12.002) | Australia | Prospective cohort | Adults and Paediatrics | Vascular | To review the results of a junior general neurosurgeon performing aneurysm surgery and compare these to the remainder of his low-volume unit. |  | Yes | Not defined | Reported 3 patients had infection, with 1 having a positive CSF culture | No |
| Golebiowski A. et al., 2015 | [10.1007/s00701-014-2286-3](https://doi.org/10.1007/s00701-014-2286-3) | Norway | Retrospective cohort | Adult | General (All neurosurgical cases) | To explore possible associations between duration of surgery and extracranial complications. To assess the risk of surgical site infections (SSIs) (cranial and intracranial infections) in relation to duration of surgery. |  | No | Not defined | Both extracranial complications and SSIs were significantly more common following longer lasting procedures. | No |
| Gonzalez-Vargas P.M. et al., 2020 | [10.1016/j.inat.2019.100606](https://doi.org/10.1016/j.inat.2019.100606) | Spain | Retrospective cohort | Adults and Paediatrics | Trauma | To identify different factors that are associated with lower Glasgow Outcome Scale (GOS) values and to establish with statistical significance several elements that influence the GOS at 12 months of follow-up in subdural haematoma treatment. |  | No | Not defined | Reported 0.7% patients experienced SSI. | No |
| Gorgulho A. et al., 2009 | [10.3171/2008.6.17603](https://doi.org/10.3171/2008.6.17603) | USA | Retrospective case-control | Adult | Functional (DBS insertion) | To compare the incidence of infection in patients undergoing surgery in the conventional versus MR imaging–equipped theatre. | Conventional vs MR imaging equipped theatre | No | Guideline for Prevention of Surgical Site Infection | Reported 20 cases of infection. The difference in infection risk was also not statistically significant between the two groups. | No |
| Goshtasbi K. et al., 2020 | [10.1016/j.clineuro.2020.106192](https://doi.org/10.1016/j.clineuro.2020.106192) | USA | Retrospective cohort | Adult | Neuro-onc ology | To evaluate whether increased body mass index (BMI), age, or frailty influence vestibular schwannoma short-term surgical morbidity. |  | No | Not defined | Reported 15 cases of wound infection in obese patients compared to 9 cases in non-obese patients. | No |
| Gottsche J. et al., 2019 | [10.1055/s-0039-1698391](https://doi.org/10.1055/s-0039-1698391) | Germany | Retrospective cohort | Adults and Paediatrics | General (Cranioplasty) | To investigate the frequency and time of occurrence of complications following cranioplastic procedures in children and adults. | . | No | Not defined | Postsurgical complications after cranioplasty including surgical site infection and postoperative bleeding leading to further surgery occurred in 61.1% of the paediatric cases and in 17.8% of the adult population. Reported 1 case (5.6%) of SSI in children group and 3 cases (2.5%) in adult group. | No |
| Govindaswamy A. et al., 2022 | [10.4103/ajns.AJNS_268_18](https://doi.org/10.4103/ajns.ajns_268_18) | India | Retrospective cohort | Adults and Paediatrics | Trauma | To investigate the prevalence of Postoperative central nervous system infections (PCNSIs) and antibiotic resistance profiles of causative organisms in trauma patients following neuroinvasive procedures. |  | Yes | CDC guidelines | 961 patients were screened for PCNSIs. The estimated prevalence of PCNSIs which is a type of organ/space SSI was 7.2% (6.3–8.3). Males were predominantly affected (85.0%). | No |
| Goyal-Honavar A. et al., 2022 | [10.1016/j.jocn.2022.06.024](https://doi.org/10.1016/j.jocn.2022.06.024) | India | Prospective | Adult | General (All neurosurgical cases) | To determine the incidence, causes and outcomes of postoperative fever in neurosurgical patients.  To evaluate a protocol for management of postoperative fever. |  | Yes | Not defined | The most common causes of fever were urinary tract infections (13.7%), followed by aseptic meningitis (10.8%), wound infections (7.8%), pneumonia (5.8%), bacterial meningitis (3.9%).  In the late postoperative period, wound infections (6 cases) were the most common cause of fever. Wound infections accounted for fever among 55.4% of patients with a wound drain in-situ for 3 days or longer, compared to 9.5% of patients that had drains removed earlier. | No |
| Grau S. et al., 2018 | [10.4103/joacp.JOACP_373_16](https://doi.org/10.4103/joacp.joacp_373_16) | Germany | Retrospective cohort | Adult | General (All cranial neurosurgical cases) | To evaluate brain swelling, intraoperative conditions, surgical course, and postoperative complication rates of propofol‑based vs. volatile‑based anaesthesia. |  | No | Not defined | Most common surgical complication was postoperative wound and bone flap infection. | No |
| Greenberg J.K. et al., 2016 | [10.3171/2015.10.PEDS15369](https://doi.org/10.3171/2015.10.peds15369) | USA | Retrospective cohort | Paediatric | Paediatric/ Skullbase | To investigate CM-I surgical outcomes using population-level administrative billing data. |  | Yes | Not defined | Reported 15 cases of wound infection (1.6%). | No |
| Grossman R. et al., 2013 | [10.1245/s10434-012-2748-x](https://doi.org/10.1245/s10434-012-2748-x) | Israel | Retrospective cohort | Adult | Neuro-oncology | To compare surgical outcome of elderly patients undergoing awake-craniotomy to that of younger patients. |  | No | Not defined | The rate of SSI was slightly decreased in the older population compared to the younger group (1.1 % vs. 2.1 %, respectively). | No |
| Gruenbaum S. et al., 2017 | [10.1213/ANE.0000000000001946](https://doi.org/10.1213/ane.0000000000001946) | USA | Prospective cohort | Adult | General (Craniotomy) | To assess the association of severe intraoperative hyperglycemia (SIH) with the occurrence of composite infections after craniotomy. |  | Yes | CDC guidelines | Reported 10 of 38 (26.3%) hyperglycemic patients developed an infection compared with 12 of 186 (6.5%) normoglycemic patients. | No |
| Grundy T et al. 2019 | [10.1080/02688697.2019.1645298](https://doi.org/10.1080/02688697.2019.1645298) | UK | Prospective | Adult | General (All cranial neurosurgical cases) | To identify the best time point at which SSI should be measured in patients undergoing a neurosurgical procedure to guide SSI surveillance practice. | . | Yes | CDC guidelines | SSIs were measured from day 2 to day 390 after surgery. Total of 86 cases (out of 3531) of SSI. 54 (62.8%) cases were identified within 30 days of surgery. The mean number of days at which SSI was first clinically diagnosed in this series was 53 days. 75% of cases were detected by 49 days with 11 (12.8%) presenting between 30 and 49 days. A total of 32 (37.3%) presented outside of the 30-day period of surveillance. The number of cases requiring reoperation in this series was 56 (65%). 22 cases (26%) that required surgery related to SSI were detected outside of 30 days. | No |
| Guidry BS et al. 2022 | [10.1227/neu.0000000000002053](https://doi.org/10.1227/neu.0000000000002053) | USA | Retrospective cohort | Adults and Paediatrics | Vascular/ Trauma | To determine factors associated with loss-to-follow-up and unplanned readmission, emphasising socioeconomic status. |  | No | Not defined | Of a total of 49 patients who were readmitted, 35 were for a neurosurgical reason (71%), including 5 patients for SSI. | No |
| Gupta A. et al., 2018 | [10.1016/j.wneu.2018.07.200](https://doi.org/10.1016/j.wneu.2018.07.200) | India | Retrospective cohort | Adult | General (Craniotomy) | To analyse the effect of the staphylococcal decolonisation regimen (SDR) and the change in antibiotic prophylaxis on the incidence of postoperative meningitis in patients who had undergone elective craniotomy. | Control vs use of SDR and change in antibiotic prophylaxis | No | Not defined | The rate of superficial scalp wound infections in group with SDR and change in antibiotic prophylaxis (8 of 727) was less than one half of that of the control (14 of 622; 1.1% vs. 2.3%). However, the difference was not statistically significant. | No |
| Gupta S. et al., 2019 | [10.1016/j.wneu.2018.09.081](https://doi.org/10.1016/j.wneu.2018.09.081) | USA | Retrospective cohort | Adult | Neuro-oncology | To analyse the impact of patient-level and surgical factors on readmission and reoperation in the resection of benign cranial nerve tumours. |  | No | Not defined | Reported a rate of 2.6% of SSI. Surgical site infections and meningitis accounted for 56.0% and 20.0% of infection-associated readmissions, respectively. | No |
| Gupta S. et al., 2021 | [10.3389/fonc.2021.662943](https://doi.org/10.3389/fonc.2021.662943) | USA | Retrospective cohort | Adult | Neuro-oncology | To investigate factors that contribute to adverse events following resection of brain metastases in order to inform selection of patients for surgery. |  | Yes | Not defined | SSI occurred in 1.49% of cases. Superficial site infections and sepsis tended to be more common after discharge than before discharge. Unplanned readmission was observed in 12% of cases (427 cases); the most frequent reason was surgical site infections (SSI) (39 cases). | No |
| Guyolla Y.H. et al., 2022 | [10.1016/j.inat.2022.101704](https://doi.org/10.1016/j.inat.2022.101704) | Ethiopia | Retrospective cohort | Adults and Paediatrics | General (All cranial neurosurgical cases) | To describe the frequency of postoperative CSF leak and associated factors after elective cranial procedures. |  | Yes | Not defined | Patients with CSF leaks have a higher risk of developing postoperative infection and prolonged hospital stay. A total of 29 (10 %) patients developed a postoperative infection, of which 22 (7.6 %) and 7 (2.4 %) were CNS infections and SSIs respectively. Patients with CSF leaks have 25 times more likely to develop postoperative infection and 2.6 times more likely to stay longer in the hospital. | No |
| Hale A.T. et al., 2020 | [10.3171/2019.9.PEDS1939](https://doi.org/10.3171/2019.9.peds1939) | USA | Retrospective cohort | Paediatric | Neuro-oncology | To systematically evaluate the influence of various clinical, radiological, and surgical factors contributing to CSF leak, pseudomeningocele, wound infection, persistent hydrocephalus, and 90-day readmission rates. |  | Yes | Requiring debridement within 180 days following surgery | Reported a protective effect of graft dural closure versus primary dural closure (7% vs 35%) on wound infection rates, however, patients with hydrocephalus ultimately requiring permanent CSF diversion (57% vs 26%) were more likely to be diagnosed with wound infection requiring surgical debridement. | No |
| Halpern C.H. et al., 2012 | [10.1016/j.ajic.2011.06.005](https://doi.org/10.1016/j.ajic.2011.06.005) | USA | Retrospective cohort | Adult | Functional (DBS insertion) | To investigate the impact of the use of a 70% ethyl alcohol antiseptic wash on post-operative infection rates in a series of consecutive patients treated with DBS for movement disorders. |  | Yes | Swelling, redness, pain, warmth, drainage, or fluid collection involving the DBS system or the skin incision. | Reported 11 cases of infection (6.47%), all in the group without the preoperative antiseptic wash. The infection rate was 9.02% in the group without the preoperative wash and 0 in the group with the preoperative wash. | No |
| Hamdeh S.A. et al., 2014 | [10.3109/02688697.2013.835376](https://doi.org/10.3109/02688697.2013.835376) | Sweden | Prospective cohort | Adult | General (All neurosurgical cases) | To measure the incidence of SSI at 3- and 12-months following discharge after defined standard neurosurgical procedures.  To characterise the primary risk factors for patients in our clinic developing SSI. |  | Yes | CDC guidelines | Reported 20 out of 466 cases (4.3% of procedures) developed infections within 3 months and another 3 (4.9% of procedures) within 12 months. Risk factors for SSI were meningioma, longer operation time, craniotomy, dural substitute, and staples in wound closure. | No |
| Hammond C.J. et al., 2002 | PMID: 12617235 | UK | Retrospective cohort | Adults and Paediatrics | General (All neurosurgical cases) | To assess the prevalence of MRSA in patients admitted to inpatient wards.  To assess at what prevalence of MRSA is it appropriate to change the prophylactic regimen to include MRSA cover and what antibiotic should be used. |  | Yes | Not defined | Of 107 ‘inpatient’ patients who were screened 16 were MRSA carriers. The MRSA prevalence in patients referred from other hospitals was 15%. | No |
| Han R.H. et al., 2016 | [10.3171/2015.7.PEDS15187](https://doi.org/10.3171/2015.7.peds15187) | USA | Retrospective case-control | Adult | Skullbase | To examine complications in patients undergoing surgery for craniosynostosis using both minimally invasive endoscopic and open approaches. | Minimally invasive vs open surgical procedures | Yes | Not defined | Reported 2 reoperations due to wound infections. | No |
| Hardaway F.A. et al., 2017 | [10.1093/neuros/nyx505](https://doi.org/10.1093/neuros/nyx505) | USA | Retrospective cohort | Adult | Functional (DBS insertion) | To study short and long-term DBS-related infection rates.  To evaluate any potential seasonality associated with DBS-related infections. |  | Yes | Guideline for Prevention of Surgical Site Infection | Reported 13 patients developed infections within 6 months of surgery. The median time to postoperative infection was 33 days. Ten of 13 patients had infections that were localised to the generator site, and 1 patient had an intracranial lead infection.  Across the 5-year period, the infection rate for the summer months (July-September) was highest with a rate of 4.05% (6 of 148 surgeries total). | Yes |
| Hardesty D.A. et al., 2021 | [10.3171/2020.8.JNS202404](https://doi.org/10.3171/2020.8.jns202404) | USA | Retrospective cohort | Adult | General (All cranial neurosurgical cases) | To assess why neurosurgical patients utilise hospital emergency rooms (ERs) with or without subsequent admission in the postoperative setting. |  | No | Not defined | The main contributors to increased rates of readmission to the hospital from the ER were CSF leak and wound infection. | No |
| Hardy S. et al., 2010 | [10.3171/2010.2.JNS09950](https://doi.org/10.3171/2010.2.jns09950) | USA | Retrospective cohort | Not reported | Neuro-oncology | To assess whether SSIs in patients with brain tumours undergoing resection are associated with hyperglycaemia. |  | Yes | CDC guidelines | Glucose level was not a significant factor in postoperative SSI after adjusting for duration of surgery and adherence to antibiotic prophylaxis. However, duration of surgery was significantly associated with postoperative SSI. | No |
| Harland T. et al., 2023 | [10.1227/ons.0000000000000698](https://doi.org/10.1227/ons.0000000000000698) | USA | Retrospective cohort | Adult | Functional (DBS insertion) | To describe a modified surgical technique for interventional MRI-guided stereotactic procedures. |  | No | Not defined | Complications included infection with hardware removal (2.5%) and superficial haemorrhage without permanent neurological sequelae (1.9%). | No |
| Harrop J.S. et al., 2010 | [10.1227/01.NEU.0000370247.11479.B6](https://doi.org/10.1227/01.neu.0000370247.11479.b6) | USA | Retrospective cohort | Adult | Trauma | To compare infection rates after implementing a standardised protocol for ventriculostomy catheter insertion with and without the use of antibiotic-impregnated catheters. | With vs without antibiotic-impregnated catheters. | Yes | 1. Two positive CSF cultures from ventriculostomy catheters.  2. Increase in cerebrospinal fluid white blood cell count. | Reported the baseline infection rate of 6.7%. Antibiotic impregnation of catheters lowered this infection rate to 1.0%. | No |
| Hasegawa H. et al., 2021 | [10.3171/2020.7.JNS201385](https://doi.org/10.3171/2020.7.jns201385) | USA | Retrospective cohort | Adults vs paediatrics | Functional (VNS insertion for epilepsy) | To provide insight into the optimal management of VNS-related SSI (VNS-SSI). |  | Yes | If a patient had undergone VNS-related procedures and developed one or more signs:  1. Purulence  2. Positive culture  3. Superficial signs: wound dehiscence, swelling, drainage, and redness. | A total of 16 patients were found to have VNS-SSI. Among the 16 cases of VNS-SSI, 11 occurred after initial VNS placement and 5 occurred after generator replacement. Wound dehiscence, redness, swelling, and drainage were the typical symptoms of VNS-SSI. | No |
| Hayashi T. et al., 2010 | [10.3171/2010.5.PEDS1018](https://doi.org/10.3171/2010.5.peds1018) | Japan | Prospective case-control | Paediatric | Paediatric/ Trauma | To assess whether reducing bacteria in the operating field and wound leads to a reduction in shunt infection rate. | Group A: control group with no irrigation technique Group B: saline containing amikacin for irrigation Group C: saline only for irrigation | Yes | 1. Positive CSF- or catheter-based cultures  2. At least 1 clinical component of infection: headache, meningitis, fever, elevated peripheral leukocyte count, elevated C-reactive protein  3. CSF white blood cell count > 40/mm3  4. obvious cellulites at the operative site  5. exposure of the shunt hardware through an open incision | Reported 9 cases of shunt infection in the defined post-operative period.  There was a statistically significant difference in the rate of infection between Groups A and B, Groups A and C, and between Group A and Groups B and C combined. No significant difference was observed between the 2 groups in which irrigation was used. | No |
| He J. et al., 2023 | [10.3389/fneur.2023.1153392](https://doi.org/10.3389/fneur.2023.1153392) | China | Retrospective cohort | Adult | Neuro-oncology | To investigate the effects of intraoperative steroid administration on postoperative 30-day mortality in patients undergoing craniotomy for brain tumours. | With vs without intraoperative steroid administration | Yes | Not defined | The administration of intraoperative steroids was not associated with SSI. | No |
| Helal A. et al., 2018 | [10.3171/2018.7.FOCUS18258](https://doi.org/10.3171/2018.7.focus18258) | Egypt | Retrospective cohort | Adults and Paediatrics | Neuro-oncology | To assess whether the application of recent technological advances such as intraoperative MRI, image guidance, and other techniques in routine day-today practice is beneficial in attaining good outcomes. |  | Yes | Not defined | Reported a surgical site infection rate of 3.5%. | No |
| Helmers A.K. et al., 2018 | [10.1016/j.wneu.2018.01.183](https://doi.org/10.1016/j.wneu.2018.01.183) | Germany | Retrospective cohort | Adults and Paediatrics | Functional (DBS insertion) | To investigate complication rates after impulse generator exchange surgery and identify risk factors. |  | Yes | Not defined | Total of 12 wound infections recorded. | No |
| Henderson D. et al., 2022 | [10.25259/SNI_103_2022](https://doi.org/10.25259/sni_103_2022) | UK | Retrospective case series | Adult | Neuro-oncology | To identify prognostic factors associated with resection of intracranial metastases. |  | No | Not defined | SSI occurred in 4%: 5 patients postoperatively (out of 124 total patients). | No |
| Henry R.K. et al., 2021 | [10.1002/lary.29485](https://doi.org/10.1002/lary.29485) | USA | Retrospective cohort | Adult | Skullbase | To investigate the utility of a frailty index in predicting complications after skull base operations. |  | Yes | Not defined | Many local surgical complications do not significantly increase with mFI-5 score including superficial SSI, deep SSI, and graft, prosthesis or flap failure. | No |
| Hill T. et al., 2017 | [10.1016/j.wneu.2017.01.093](https://doi.org/10.1016/j.wneu.2017.01.093) | USA | Retrospective | Not recorded | Functional (EEG for epilepsy) | To assess the efficacy and risks of diagnostic bilateral intracranial EEG (bICEEG) in treatment-resistant epilepsy patients with poorly lateralised epileptogenic zone on non-invasive studies as reflected by progress to resection, Engel outcome and complication rate. | . | No | Not defined | The most common complications were severe infection requiring surgical intervention (2.8%) followed by superficial infection (0.9%). | No |
| Hirsch L.J. et al., 2020 | [10.1111/epi.16442](https://doi.org/10.1111/epi.16442) | USA | Retrospective cohort | Adult | Functional (temporal lobe resection for epilepsy) | To describe seizure outcomes in patients with medically refractory epilepsy who had evidence of bilateral mesial temporal lobe seizure onsets and underwent resection based on chronic ambulatory intracranial EEG  data from a direct brain-responsive neurostimulator system. |  | Yes | Not defined | Two patients developed an implant-site infection (8.3%) and one patient had a scalp dehiscence (4.2%). | No |
| Hng D. et al., 2014 | [10.1055/s-0034-1395383](https://doi.org/10.1055/s-0034-1395383) | Australia | Retrospective cohort | Adults and Paediatrics | General (Craniotomy) | To evaluate the clinical outcomes and complications of cranioplasties using cryopreserved autologous bone flaps performed over a 10-year period and analyse potential risk factors for infection. |  | Yes | Not defined | 11.2% of patients had an infection requiring removal of the bone flap. | No |
| Ho A. et al., 2018 | [10.3171/2018.5.PEDS17719](https://doi.org/10.3171/2018.5.peds17719) | USA | Retrospective cohort | Paediatric | Paediatric | To assess the use of topical vancomycin following open craniotomy in the paediatric population. | Use of topical vancomycin vs control (no topical vancomycin) | Yes | CDC guidelines | 466 open craniotomies were included of which 43% utilised topical vancomycin. There was a 1.5% SSI rate in the non-topical cohort versus 0% in the topical vancomycin cohort. There were no significant differences in risk factors for SSI between cohorts. | No |
| Hoang T. et al., 2023 | [10.1017/ice.2022.112](https://doi.org/10.1017/ice.2022.112) | Canada | Retrospective cohort | Not reported | General (Craniotomy) | To evaluate the utility of autologous bone-flap swab cultures performed at the time of cranioplasty in predicting post-cranioplasty surgical site infection. |  | Yes | CDC guidelines | 16 out of 282 cases (5.6%) developed SSI after cranioplasty. A high percentage of bone-flap swab cultures were positive at the time of craniectomy (66.7%) and cranioplasty (59.5%). Bone-flap swab culture had poor sensitivity, specificity and positive likelihood ratio for predicting post-cranioplasty SSI. | No |
| Hoffman H. et al., 2019 | [10.1016/j.wneu.2019.05.021](https://doi.org/10.1016/j.wneu.2019.05.021) | USA | Retrospective cohort | Adult | Vascular | To analyse annual trends, causes, and predictors for 30-day and 90-day readmissions over a 5-year period in patients who underwent Cerebral Aneurysm Clipping. | . | No | Not defined | Wound infection was the most common cause associated with 30-day readmissions (8.8%) and was the third most common among 90-day readmission (10%). Wound infection occurred in 9 cases (8.8%). | No |
| Honeybul S. et al., 2016 | [10.1080/02688697.2016.1187259](https://doi.org/10.1080/02688697.2016.1187259) | Australia | Retrospective cohort | Adults and Paediatrics | Trauma | To determine which factors influenced the incidence of cranioplasty complications and failure. |  | Yes | Not defined | Forty-two patients (8.2%) developed cranioplasty infection that necessitated removal of the implant. | No |
| Honeybul S. et al., 2012 | [10.1097/PRS.0b013e318267d4de](https://doi.org/10.1097/prs.0b013e318267d4de) | Australia | Retrospective cohort | Adult | Trauma | To investigate the outcome of cranioplasty in patients with a single primary abnormality treated with a single material. |  | Yes | Infection requiring removal of autologous bone in patients who survived for at least one year after the initial cranioplasty. | Thirteen patients (8.5%) developed an infection requiring removal of the autologous bone. Most of the infections developed within the first postoperative month. | No |
| Hoover J.M. et al., 2013 | [10.3171/2013.2.JNS121731](https://doi.org/10.3171/2013.2.jns121731) | USA | Retrospective cohort | Adult | Neuro-oncology | The object of this study was to assess outcomes after surgery for recurrent intracranial glioma. |  | Yes | Not defined | The most common regional complications were wound infection or CSF-related complications. 14 cases of wound infection (4%). | No |
| Horisawa S. et al., 2019 | [10.1212/WNL.0000000000006818](https://doi.org/10.1212/wnl.0000000000006818) | Japan | Retrospective cohort | Adults and Paediatrics | Other (Ventro-oral thalamotomy) | To report the safety and long-term efficacy of ventro-oral thalamotomy for 171 patients with task-specific focal hand dystonia. |  | No | Not defined | Surgical site infection was observed in 2 patients. | No |
| Hu H. et al., 2022 | [10.1016/j.jgar.2023.08.006](https://doi.org/10.1016/j.jgar.2023.08.006) | China | Retrospective cohort | Adult | General (All neurosurgical cases) | To evaluate the epidemiology and clinical features of carbapenem-resistant Enterobacteriaceae (CRE) induced post-operative CNS infection.  To assess the efficacy of various treatment strategies and identify risk factors linked to mortality. |  | Yes | 1. Positive CSF culture after neurosurgery  2. CSF leukocyte count > 100 × 106/L, neutrophils ratio > 70%, and glucose levels < 2.2 mmol/L | 254 cases of post-operative CNS infection reported. | No |
| Huang Y. et al., 2011 | [10.1016/j.injury.2011.11.005](https://doi.org/10.1016/j.injury.2011.11.005) | Taiwan | Retrospective cohort | Adult | General (Cranioplasty) | To identify the risk factors of autologous bone flap infection. |  | Yes | Less than 14 days after cranioplasty:  1. Pus or infected fluid in the subgaleal layer with or without involvement of epidural and subdural spaces  2. Extensive infection necessitating the removal of the bone flap  3. CT scans indicating infection  4. Requirement of surgical debridement  5. Abscess cultures and swab cultures from skull flaps. | Acute bone flap infection was identified in 5 of the 153 cranioplasties (3.3%). | No |
| Huang Y.H. et al., 2013 | [10.1016/j.ijsu.2013.07.013](https://doi.org/10.1016/j.ijsu.2013.07.013) | USA | Retrospective cohort | Adult | Trauma | To identify the relationship between the timing of cranioplasty and neurological outcomes following post-traumatic craniectomy. |  | Yes | Not defined | SSI occurred in 10 patients. | No |
| Huang Y.H. et al., 2011 | [10.1097/TA.0b013e318203208a](https://doi.org/10.1097/ta.0b013e318203208a) | Taiwan | Retrospective cohort | Adult | Trauma | To compare morbidities after craniectomy and cranioplasty in traumatic brain injury patients with and without the use of Neuro-Patch. |  | Yes | CDC guidelines. | The incidence of neurosurgical site infection after craniectomy or cranioplasty showed no intergroup difference. | No |
| Hutchinson P.J. et al., 2020 | [10.1056/NEJMoa2020473](https://doi.org/10.1056/nejmoa2020473) | UK | Randomised trial | Adult | Trauma | To assess the effect of dexamethasone on outcomes in patients with symptomatic chronic subdural hematoma. |  | No | Not defined | Reported 12 out of 699 patients (1.7%) had a surgical site infection: 8 in the dexamethasone group and 4 in placebo group. Two patients had a superficial wound infection and 10 had a subdural empyema. | No |
| Hutter G. et al., 2014 | 10.3171/2014.6.JNS131917 | Switzerland | Randomised clinical trial | Adult | General (Craniotomy) | To investigate whether the addition of TachoSil on top of the dural suture reduces postoperative CSF leakage compared with dural suturing alone.  To assess the frequency and risk factors for dural leakage and potentially related complications after elective craniotomy. |  | Yes | Not defined | Reported 5 patients in the control group, compared with only 1 in the study group, suffered from postoperative wound infection or meningitis, suggesting that the application of TachoSil may be beneficial. | No |
| Im S.H. et al., 2012 | [10.3340/jkns.2012.52.4.396](https://doi.org/10.3340/jkns.2012.52.4.396) | South Korea | Retrospective cohort | Adult | Trauma | To compare the long-term incidences of SSI according to the graft material and cranioplasty timing after craniectomy, and to determine the associated factors of cranioplasty infection. |  | Yes | 1. Prescription for antibiotics more than 2 weeks after cranioplasty without other organ infection  2. Subcutaneous or subgaleal abscess  3. Radiologic records related to subcutaneous, subgaleal, epidural or subdural empyema  4) Requirement of bone flap removal or wound revision and irrigation | There was no significant difference in long-term outcome for cranioplasty infection between cryopreserved and artificial bone grafts. The incidence rates for site infection in cryopreserved and artificial bone grafts were 14.4% and 4.2%, respectively. The incidence rates for site infection for early and late repair were also 14.4% and 4.2%. | No |
| Inoue T. et al., 2020 | [10.1007/s00701-020-04242-8](https://doi.org/10.1007/s00701-020-04242-8) | Japan and Indonesia | Retrospective cohort | Adult | Vascular | To address the necessity of resecting suprameatal tubercle in microvascular decompression for trigeminal neuralgia. |  | No | Not defined | Post-operative wound infection was noted in one patient, in whom bone removal and debridement were required. | No |
| Isaac K.V. et al., 2018 | [10.3171/2018.5.PEDS1846](https://doi.org/10.3171/2018.5.peds1846) | USA | Retrospective case-control | Adult | Skullbase | To compare the effectiveness of two main surgical techniques used for treating sagittal craniosynostosis: endoscopic suturectomy and cranial vault remodelling. | Endoscopic suturectomy vs cranial vault remodelling | Yes | Not defined | 2 cases of SSI (1%) reported. | No |
| Isobe N. et al., 2018 | [10.1016/j.wneu.2018.07.080](https://doi.org/10.1016/j.wneu.2018.07.080) | Japan | Retrospective cohort | Adult | Neuro-oncology | To analyse the clinical characteristics of older patients who underwent surgical resection of meningioma, such as body mass index and serum albumin level as elements associated with frailty, and investigated the factors associated with postoperative deterioration. |  | No | Not defined | 1.9% of patients experienced wound infection. | No |
| Janjua M. et al., 2020 | [10.3171/2019.7.PEDS19272](https://doi.org/10.3171/2019.7.peds19272) | USA | Retrospective cohort | Paediatric | Paediatric/ Neuro-oncology | To evaluate the readmission rates and predictors of readmission after paediatric brain tumour resection. | . | Yes | Not defined | Reported a surgical site infections or septicemia rate of 14%. | No |
| Jeong T.S. et al., 2018 | [10.3340/jkns.2018.0021](https://doi.org/10.3340/jkns.2018.0021) | Korea | Prospective observational | Adult | General (Craniotomy) | To investigate the rates, types, and risk factors of SSI following intracranial neurosurgical procedures. |  | Yes | CDC guidelines | Out of the 1576 cases included, 30 showed infection, for an overall SSI rate of 1.9%. Organ/space infection was the most common, found in 21 out of the 30 cases (70%). Only preoperative hospital stay was significantly associated with the incidence of SSI. | No |
| Jeong T.S. et al., 2020 | [10.1371/journal.pone.0232561](https://doi.org/10.1371/journal.pone.0232561) | South Korea | Retrospective case-control | Adult | Trauma | To determine the safety and feasibility of non-suture duraplasty in the context of decompressive craniectomy in traumatic brain injury patients. | Suture duraplasty vs non-suture duraplasty groups | Yes | CDC guidelines | Wound dehiscence and SSIs were evaluated for the presence of any complications, revealing no significant differences between the two groups. | No |
| Jia C. et al., 2019 | [10.1016/j.clineuro.2018.11.008](https://doi.org/10.1016/j.clineuro.2018.11.008) | China | Retrospective cohort | Adult | Skullbase | To assess the two surgical procedures of posterior fossa decompression in treating Chiari malformation type I complicated by syringomyelia, and to evaluate the postoperative complications, surgical effects and prognosis. |  | Yes | Not defined | Reported 3 vs 2 wound infections in the posterior fossa decompression combined with the resection of tonsils and posterior fossa decompression with duraplasty groups respectively. | No |
| Jiang X. et al., 2014 | [10.5137/1019-5149.JTN.12738-14.0](https://doi.org/10.5137/1019-5149.jtn.12738-14.0) | China | Prospective cohort | Adult | General (All cranial neurosurgical cases) | To determine whether the trend of SSI incidence did change or not following the implementation of the study’s program. |  | Yes | CDC guidelines | Reported 112 SSI cases out of 3042 cases. SSI more likely occurred in patients with older age, undergoing emergency operations, having higher American Society of Anesthesiologists (ASA) scores and clean contaminated wound. | No |
| Jimenez A. et al., 2021 | [10.1016/j.wneu.2021.12.010](https://doi.org/10.1016/j.wneu.2021.12.010) | USA | Retrospective cohort | Adult | Neuro-oncology | To better understand the prognostic factors that influence high-value care outcomes after meningioma surgery. To develop predictive models to determine the patients at risk of experiencing an extended hospital length of stay, non-routine discharge disposition, and/or a 90-day hospital readmission after non-skull base meningioma resection. |  | Yes | Not defined | Reported 20 cases (5.1%) had developed surgical site infections. | No |
| Jimenez-Martinez E. et al., 2019 | [10.1186/s13756-019-0525-3](https://doi.org/10.1186/s13756-019-0525-3) | Spain | Prospective cohort | Adult | General (Craniotomy) | To identify the risk factors for developing SSI-CRAN in a large prospective cohort of adult patients undergoing craniotomy. |  | Yes | CDC guidelines | Among the 595 patients who underwent craniotomy, 91 (15.3%) episodes of SSI-CRAN were recorded, 67 (73.6%) of which were organ/space. The factors associated with SSI-CRAN were ASA score > 2, extrinsic tumour and re-intervention. | Yes: “The high rate of SSI-CRAN  found in the current study could be partly explained by  the use of different definitions and a stricter and longer  patient follow-up than in previous research. The CDC score is a well-established tool for the classification  of surgical site infection and provides homogeneity among  studies; however, it only includes surgical site infection  within 30 days after surgery. In contrast, our data, with a follow up of one year, found the median time for the occurrence of SSI-CRAN to be 39 days; furthermore, most SSI-CRANs were detected in the post-discharge surveillance period, and frequently required hospital readmission. If the follow up was for 30  days the SSI-CRAN rate would be 58 episodes (9.7%), almost half of these episodes would have been lost. These  findings concur with the results of other studies suggesting that limiting follow-up to 30-days would cause several cases to be missed. In our view, the CDC scoring  system should be used, but with a minimum follow-up  time of 3 months.” |
| Jimenez-Martinez E. et al., 2021 | [10.1093/cid/ciaa884](https://doi.org/10.1093/cid/ciaa884) | Spain | Retrospective cohort | Adult | General (Craniotomy) | To determine whether the implementation of a care bundle was effective in preventing SSI-CRANs. |  | Yes | CDC guidelines | A total of 595 and 422 patients were included in the preintervention and intervention periods, respectively. The incidence of SSI-CRANs was lower in the intervention period. The care bundle intervention was independently associated with a reduced incidence of SSI-CRANs. | No |
| Jimenez-Martinez E. et al., 2021 | [10.1186/s13756-021-01016-4](https://doi.org/10.1186/s13756-021-01016-4) | Spain | Retrospective cohort | Adult | General (Craniotomy) | To analyse the economic impact on hospital costs of the implementation of a care bundle for the prevention of SSI-CRAN. |  | Yes | CDC guidelines | The incidence of SSI-CRANs was significantly lower in the care bundle period (15.3% vs. 3.5%). | No |
| Joerger A. et al., 2023 | [10.1007/s00701-023-05870-6](https://doi.org/10.1007/s00701-023-05870-6) | Germany | Retrospective cohort | Adults and Paediatrics | Neuro-oncology | To compare the rates of SSI after brain tumour surgery with and without the use of intraoperative MRI (iOMRI) by a descriptive analysis in a real-world scenario. | Use of intraoperative MRI vs control (no ioMRI) | Yes | Classified as superficial, deep, epidural and intracranial (empyema, abscess), meningitis/ ventriculitis, infected cerebrospinal fluid (CSF) fistula and shunt infection. | The rate of SSI in the different groups did not differ significantly. There was no significant influence of re-resection, prior radiotherapy/chemotherapy, blood loss or duration of surgery was found on the incidence of SSI. | No |
| Jorger A. et al., 2018 | [10.1007/s00701-018-3704-8](https://doi.org/10.1007/s00701-018-3704-8) | Germany | Retrospective cohort | Adult | General (All cranial neurosurgical cases) | To find out if the implementation of the new infection prevention bundle could reduce the rate of SSIs. The benefit of an infection prevention bundle made up of five basic procedures to avoid SSIs following cranial surgery was evaluated. | Control vs use of new infection prevention bundle. | Yes | Not defined | In the control group, 13 (4%) patients suffered from an SSI requiring surgical intervention, compared to 6 patients (2%) in the study group. In the control group, the median of days to reoperation as an indirect measurement for the occurrence of a SSI was 35 days, which was comparable with 32.5 days in the study group. | No |
| Joswig H. et al., 2016 | [10.1016/j.wneu.2016.03.081](https://doi.org/10.1016/j.wneu.2016.03.081) | Switzerland | Retrospective cohort | Adult | Trauma | To test the hypothesis that complication rates and postoperative outcome of supervised residents and experienced board-certified faculty neurosurgeons (BCFNs) are similar in cranioplasty surgery. |  | Yes | Not defined | SSI requiring cranioplasty removal occurred in 27 patients: 11.3%; 19 in the teaching and 8 in the nonteaching group. | No |
| Kaestner S. et al., 2017 | [10.1016/j.pjnns.2016.11.007](https://doi.org/10.1016/j.pjnns.2016.11.007) | Germany | Retrospective cohort | Adult | Trauma | To compare different hydrocephalus types with the revision rates and their time course to give a hint to the question if hydrocephalus could be a temporary condition especially in the case of post-haemorrhagic hydrocephalus. |  | No | Not defined | This study listed shunt infection rates based on the sub-group of procedure but did not directly report these in the text. | No |
| Kalangu K. et al., 2020 | [10.1007/s00381-019-04357-z](https://doi.org/10.1007/s00381-019-04357-z) | Zimbabwe, Namibia, Democratic Republic of Congo | Prospective | Paediatric | Paediatric/ CSF dynamics | To prospectively establish the incidence of shunt infection in the early post-shunt period following the study’s protocol and elucidate on associated risk factors. |  | Yes | CDC guidelines | Included were 211 ventriculoperitoneal shunt procedures performed on 209 children. Shunt infection rate was 1.9% (4 cases in total) per procedure. | No |
| Kalani M.Y.S. et al., 2015 | [10.1016/j.wneu.2014.10.013](https://doi.org/10.1016/j.wneu.2014.10.013) | USA | Retrospective cohort | Adult | Vascular | To define indications for and evaluate outcomes of patients treated with bypass surgery in the modern endovascular era. |  | No | Not defined | 2 cases of wound infection were recorded. | No |
| Kamenova M. et al., 2017 | [10.1016/j.wneu.2017.01.065](https://doi.org/10.1016/j.wneu.2017.01.065) | Switzerland | Prospective case-control | Adult | Trauma | To compare the perioperative and postoperative bleeding and cardiovascular complication rates of patients undergoing burr-hole drainage for chronic subdural haematoma with and without discontinuation of low-dose Acetylsalicylic Acid (ASA). | With vs without discontinuation of low dose ASA | Yes | Not defined | Reported 2 infections in ASA group compared to 6 cases of infection in the control group. | No |
| Karabacak M. et al. 2024 | [10.1089/neu.2023.0122](https://doi.org/10.1089/neu.2023.0122) | USA | Retrospective cohort | Adult | Trauma | To use machine learning (ML) algorithms to predict in-hospital death, non-home discharges, prolonged length of stay, prolonged length of intensive care unit stay, and major complications in patients with acute traumatic epidural haematoma.  To incorporate the resulting ML models into a user-friendly web application for use in the clinical settings. | . | Yes | Not defined | Reported 1 case of deep SSI out of 4586 patients and 1 case of superficial SSI. | No |
| Kassicieh A. et al., 2022 | [10.1016/j.clineuro.2022.107383](https://doi.org/10.1016/j.clineuro.2022.107383) | USA | Retrospective cohort | Adult | General (All cranial neurosurgical cases) | To analyse the effect of interhospital transfer (IHT) and frailty on postoperative outcomes in patients who underwent cranial neurosurgical operations. | Interhospital transfer vs no interhospital transfer | Yes | Not defined | Reported superficial SSI in 36 cases (0.3%) in interhospital transfer group compared to 278 cases (0.7%) in the non-interhospital transfer group. Deep SSI occurred in 29 cases (0.3%) in IHT, 132 (0.3%) in non-IHT. Organ space SSI reported in 131 cases (1.4%) in IHT, 495 cases (1.2%) in non-IHT. | No |
| Kerezoudis P. et al., 2018 | [10.3171/2016.12.JNS162096](https://doi.org/10.3171/2016.12.jns162096) | USA | Retrospective cohort | Adult | Functional (temporal lobe resection for epilepsy) | To assess 30-day morbidity and mortality after temporal lobectomy in cases registered in a national database. |  | Yes | Not defined | Reported 14 patients (6.48%) experienced at least one of the following major complications: sepsis or septic shock (0.93%), organ space infection (1.39%), deep incisional surgical site infection (0.46%) and wound dehiscence (0.93%), | No |
| Kerttula S. et al., 2022 | [10.1007/s00701-022-05337-0](https://doi.org/10.1007/s00701-022-05337-0) | Finland | Retrospective cohort | Adult | Trauma | To study the effect of antithrombotic therapy on the outcome of operatively treated chronic subdural hematomas. |  | No | Not defined | One wound infection reported out of a total of 301 patients. | No |
| Khan M.P. et al., 2021 | 10.53350/pjmhs211592770 | Pakistan | Retrospective cohort | Adults and Paediatrics | Other: surgical treatment for meningomyelocele | To determine the prevalence of early postoperative complications in patients undergoing surgical treatment for meningomyelocele. |  | Yes | Not defined | Surgical site infections were reported in 20 (14.71%) patients. | No |
| Kilgore C.B. et al., 2023 | [10.1016/j.wneu.2023.10.009](https://doi.org/10.1016/j.wneu.2023.10.009) | USA | Retrospective cohort | Adult | Vascular | To assess frailty as a predictor for long-term pain outcomes after microvascular decompression. |  | Yes | Not defined | Reported that 5 frail patients had SSI, while 1 non-frail patient had SSI. | No |
| Kim K.H. et al., 2020 | [10.1016/j.wneu.2020.05.154](https://doi.org/10.1016/j.wneu.2020.05.154) | South Korea | Retrospective cohort | Adult | Other (dural closure, posterior fossa surgery) | To evaluate the efficacy of collagen matrix inlay graft compared with other techniques in a propensity score matched cohort. |  | Yes | Not defined | SSI was reported in 3 (3.0%) patients in the inlay group and 4 (5.5%) patients in the no-inlay group. None of the SSIs were related to the use of the inlay graft. | No |
| Kim M.J. et al., 2021 | [10.3389/fneur.2021.745575](https://doi.org/10.3389/fneur.2021.745575) | Korea | Retrospective cohort | Adult | General (Cranioplasty) | To identify predictive factors for SSI following cranioplasty by reviewing procedures performed over a 10-year period. |  | Yes | 1. Requiring removal of bone flap or implant due to purulent discharge with signs of infection and complicated fluid collection, empyema, or abscess on CT scans.  2. Wound dehiscence with flap exposure requiring surgical revision without removal of bone flap or implant. | SSIs occurred in 17 patients (9.9%), of which 13 (7.6%) required removal of the bone graft, and 4 (2.3%) required wound revision without removal of implant due to wound dehiscence with flap exposure. | No |
| Kim S.P. et al., 2014 | [10.3340/jkns.2014.56.5.410](https://doi.org/10.3340/jkns.2014.56.5.410) | South Korea | Retrospective cohort | Adults and Paediatrics | Trauma | To identify the predictive factors for the development of epidural fluid collection as a complication of cranioplasty, and its outcomes. |  | Yes | Not defined | Reported infection in 5 patients. | No |
| Kim T et al. 2013 | [10.1007/s00701-013-1833-7](https://doi.org/10.1007/s00701-013-1833-7) | Korea | Retrospective cohort | Adults and Paediatrics | General (Craniotomy) | To identify the risk factors for SSIs and to assess the relationship between SSIs and the results of wound-drain-tip culture after elective supratentorial craniotomy. |  | Yes | CDC guidelines | Reported 14 (2.62 %) patients suffered from SSIs. Among the 21 patients with positive wound-drain-tip cultures, 8 (38.1 %) patients experienced SSIs. Underweight group, maintenance wound drain over 3 days, and positive wound-drain-tip culture were significantly associated with postoperative SSIs. | No |
| Kimmell K.T. et al., 2015 | [10.3171/2014.10.JNS14632](https://doi.org/10.3171/2014.10.jns14632) | USA | Retrospective cohort | Both | General (Craniotomy) | To identify risk factors predictive of venous thromboembolism (VTE) in patients undergoing craniotomy. |  | Yes | Not defined | Superficial SSI was associated with VTE as a postoperative event. | No |
| Kinaci A. et al., 2023 | [10.1227/neu.0000000000002345](https://doi.org/10.1227/neu.0000000000002345) | Europe and USA | Retrospective case control | Adult | General (Craniotomy) | To determine the incidence and risk factors of incisional CSF (iCSF) leakage after craniotomy.  To assess the complications after iCSF leakage and the success rate of iCSF leakage treatment. |  | Yes | Wound infection was subdivided into 2 categories:  1. Superficial infection requiring only antibiotics  2. Deep wound infection requiring revision surgery. Meningitis was defined as clinical suspicion for meningitis in combination with antibiotic treatment. | The odds for developing a wound infection and/or meningitis were 15 times higher in patients with iCSF leakage compared with patients without leakage. | No |
| Klekamp J., 2012 | [10.1227/NEU.0b013e31825c3426](https://doi.org/10.1227/neu.0b013e31825c3426) | Germany | Prospective | Adult | Other (Chiari malformation decompression) | This study analyses 371 decompressions focusing on intraoperative findings, analysis of complications, and long-term outcomes. |  | No | Not defined | The rate of postoperative wound infections was 0.5%. Aseptic meningitis was reported in 3.8% cases postoperatively with a trend for a higher rate using autologous rather than alloplastic dura grafts. | No |
| Klieverik V.M. et al., 2023 | [10.1016/j.wneu.2023.04.008](https://doi.org/10.1016/j.wneu.2023.04.008) | Netherlands | Retrospective cohort | Adult | General (Craniotomy) | To develop a prediction model for cranioplasty implant survival in patients undergoing cranioplasty following craniectomy. |  | Yes | A culture-positive wound swab or underlying fluid tap requiring surgical removal of the implant and antibiotic therapy. | The SSI rate differed significantly between those who had undergone skin closure with sutures or with staples (6.8% and 25.6% respectively). | No |
| Koch J. et al., 2023 | [10.1227/neu.0000000000002507](https://doi.org/10.1227/neu.0000000000002507) | Germany | Prospective cohort | Adults and Paediatrics | General (Craniotomy) | To study the efficacy of an interdisciplinary infection prevention and control bundle (IPCB) in neurosurgery. | Before, during vs after implementation of infection prevention and control bundle | Yes |  | SSIs were identified in 4.58% of patients undergoing a neurosurgical intervention before the implementation of IPCB in 2014. Following the introduction of IPCB in 2017, SSI rate remained stable at 4.43% and decreased to 4.04% after the full implementation of IPCB. | No |
| Kochanski R. et al., 2018 | 10.1093/ons/opx293 | USA | Prospective | Adults and Paediatrics | Functional (DBS insertion) | To investigate the use of vancomycin powder as an adjunct to the current antibiotic prophylaxis regimen in DBS surgery. | Pre-treatment group vs post-treatment group. Use of vancomycin powder alongside current antibiotic prophylaxis. | Yes | 1. Fever, redness, warmth, tenderness, or drainage resulting in antibiotic therapy.  2. White blood cell count, C reactive protein levels, and estimated sedimentation rate  3. Positive wound cultures  4. Device explantation | The rate of SSI prior to implementation of intrawound vancomycin was 3.1% (5 cases), which was reduced to 0.38% (1 case) in the post-treatment group. | No |
| Kogeichi Y. et al., 2022 | [10.1016/j.inat.2022.101489](https://doi.org/10.1016/j.inat.2022.101489) | Japan | Retrospective cohort | Adult | General (Craniotomy) | To assess the risk of SSI following cranioplasty in terms of the preservation method of autogenous bone graft (ABG), comparing preservation in 80% ethanol versus the standard method of subcutaneous ABG preservation. | Bone graft preserved in 80% ehtanol vs those that were preserved subcutaneously. | No | Local inflammation that required surgical removal of the bone flap. | Reported a total of 10 patients developed SSI. The timing of development of SSI after cranioplasty ranged from 4 to 240 days, with a mean of SSI development within 20 days. 80% ethanol was not significantly associated with SSI. | No |
| Koipapi S. et al., 2023 | [10.1016/j.wnsx.2023.100257](https://doi.org/10.1016/j.wnsx.2023.100257) | Tanzania | Retrospective cross-sectional | Adults and Paediatrics | Trauma | To determine outcomes and factors associated with burr hole surgery as definitive management of traumatic extra-axial hematomas. |  | Yes | Not defined | The SSI rate was 7.7% and the re-operation rate was 21%. SSI was associated with a long length of hospital stay. | No |
| Kolpa M. et al., 2019 | [10.3390/ijerph16060911](https://doi.org/10.3390/ijerph16060911) | Poland | Retrospective cohort | Adults and Paediatrics | General (All neurosurgical cases) | To determine both the epidemiology and microbiology of neurosurgical patients’ infections, as well as trends in their occurrence. |  | Yes | Not defined | 157 patients developed SSI in total. | No |
| Kombogiorgas D. et al., 2006 | [10.1007/s00381-006-0117-4](https://doi.org/10.1007/s00381-006-0117-4) | UK | Retrospective cohort | Paediatric | Paediatric | To identify possible risk factors for postoperative epilepsy and the need for prophylactic anticonvulsant therapy. |  | Yes | Not defined | The one patient who developed postoperative infection did not develop epilepsy. | No |
| Kondapavulur S. et al., 2022 | [10.1159/000520197](https://doi.org/10.1159/000520197) | USA | Retrospective cohort | Adult | Functional (DBS insertion) | To assess the efficacy of topical, intrawound vancomycin powder (VP) in reducing SSI for DBS surgery.  To examine the effect of topical vancomycin on SSI in patients after DBS surgery. |  | Yes | Infection of hardware within 6 months of implantation surgery, requiring partial, or complete hardware removal, with a positive culture. | 368 patients met inclusion criteria; 195 patients received topical VP (VP group) and 173 did not (control). There were 10 total cases of SSI: 4 patients from the VP group and 6 patients from the control group, resulting in SSI rates of 2.1 and 3.5%, respectively. Topical VP application did not significantly decrease risk of SSI. | No |
| Korhonen T.K. et al., 2019 | [10.3171/2017.12.JNS172013](https://doi.org/10.3171/2017.12.jns172013) | FInland | Retrospective cohort | Adults and Paediatrics | General (Craniectomy) | To evaluate the factors affecting the outcome of primary autologous cranioplasty, with special emphasis on bone flap resorption. |  | Yes | Not defined | The rate of SSI was higher in smokers than non-smokers. Deep SSI occurred in 19 patients (9.2%), superficial SSI was reported in 2 patients (1%). | No |
| Korinek A.M. et al., 2005 | [10.1080/02688690500145639](https://doi.org/10.1080/02688690500145639) | France | Retrospective cohort | Adult | General (Craniotomy) | To evaluate incidence and risk factors of postoperative infections, with emphasis on antibiotic prophylaxis, in a series of 4578 craniotomies. |  | Yes | CDC guidelines | The overall infection rate was 6.6%. Antibiotic prophylaxis decreased infection rate from 9.7% down to 5.8% in the entire population. | No |
| Korinek A.M. et al., 2006 | [10.1227/01.neu.0000316256.44349.b1](https://doi.org/10.1227/01.neu.0000316256.44349.b1) | France | Retrospective cohort | Adult | General (Craniotomy) | To evaluate incidence and risk factors of postoperative meningitis, with emphasis on antibiotic prophylaxis, in a series of 6243 consecutive craniotomies. |  | Yes | Not defined | The overall meningitis rate was 1.52%. Antibiotic prophylaxis reduced incision infections from 8.8% down to 4.6% but did not prevent meningitis. | No |
| Kose G. et al., 2015 | [10.1111/jocn.13149](https://doi.org/10.1111/jocn.13149) | Turkey | Randomised control | Adult | General (All cranial neurosurgical cases) | To investigate the effects of different types of shaving on body image and surgical site infection in elective cranial surgery. | Strip shaving (2cm strip) vs regional shaving (5cm strip). | Yes | Centre for Disease Control and Prevention Guidelines/ National Healthcare Safety Network Surveillance Definitions | Rate of SSI was 1% for each group and for all the patients. No difference between groups for rates of SSI. | No |
| Koskinen L.D. et al., 2013 | [10.1007/s00701-013-1856-0](https://doi.org/10.1007/s00701-013-1856-0) | Sweden | Retrospective cohort | Adults and Paediatrics | Functional (ICP device insertion) | To study complications and the insertion depth of the CMS (Codman MicroSensor) in a clinical setting. |  | Yes | Need of treatment with antibiotics. | The infection rate was 0.6% and the calculated infection rate per 1,000 catheter days was 0.8. In only one of these three patients, a severe infection resulting in an abscess was noted. | No |
| Kothari S.N. et al., 2017 | 10.1016/j.jamcollsurg.2017.07.574 | USA | Retrospective case-control | Adults and Paediatrics | General (Craniotomy) | To determine the influence of surgical cap choice on SSIs. | Bouffant cap vs skull cap | Yes | Not defined | SSI was observed in 96 (6.2%) patients; 8.1% in the bouffant group and 5% in the skullcap group. | No |
| Kourbeti I. et a.l, 2012 | 10.1111/j.1469-0691.2011.03625.x | Greece | Retrospective cohort | Adult | Trauma | To further define the risk factors and microbiology of infections in this population. |  | Yes | Not defined | The most common SSI was wound infection (2.2%), followed by meningitis/ ventriculitis (2.0%). However, shunt infections (0.8%), bone flap/ osteomyelitis or Palacos infections (0.4%), abscesses (0.1%) and epidural empyema (0.2%) were extremely rare. | No |
| Kourbeti I. et al., 2015 | 10.3171/2014.8.JNS132557 | Greece | Prospective cohort | Adult | General (Craniotomy) | To define the prevalence and microbiological characteristics of infections in patients undergoing craniotomy and to clarify the risk factors for post-craniotomy meningitis. |  | Yes | Not defined | SSIs developed after 9% of the procedures. | No |
| Krafft P. et al., 2022 | 10.1016/j.clineuro.2022.107206 | USA | Retrospective cohort | Adult | Neuro-oncology | To determine the rate of SSI in primary brain tumours, to analyse risk factors, and to evaluate effectiveness of topical vancomycin in reducing SSIs. |  | Yes | Clinical findings and symptoms meriting antibiotics treatment or re-operation for wound exploration and revision. | Topical vancomycin was associated with a significantly lower rate of SSI (0.8%) compared to standard care (5%). Narcotic use, previous brain radiation, length of hospitalisation and 30-day re-operation were associated with increased risk for SSI. | Yes |
| Kraus D. et al., 2005 | 10.1097/01.mlg.0000172201.61487.69 | USA | Retrospective cohort | Adult | Skull base | To determine:  1. The incidence and severity of infection in a group of patients treated with a non-standardised antibiotic regimen undergoing craniofacial resection  2. Whether the use of a broad spectrum antibiotic is associated with a reduced incidence and severity of infections. |  | Yes | Major infectious complications: abscess, osteomyelitis, frontal bone flap infection, meningitis, deep soft tissue infection, subdural empyema, and C. dificile enterocolitis. Minor wound infections: cellulitis, hardware infection, donor site infection, and dacryocystitis. Culture results were also used. | The standardised antibiotic therapy (CMV) was used in 90 patients, and the non-standardized antibiotics (nonCMV) were used in 107 patients. Infectious wound complications were 11% within the CMV group versus 29% in the nonCMV regimen. Severity of infections was greatly reduced in the CMV group. | Kind of |
| Kretschmer T. et al., 2009 | 10.1080/026886900417342 | Germany | Prospective cohort | Not reported | General (All cranial neurosurgical cases) | To prevent the stigmatising effect of a totally or partially shaved head with openly visible signs of a head operation, easing the reintegration of the patient into his daily life. |  | No | Not defined | Reported one wound infection (0.5%) out of the 215 total cases. | No |
| Krishnan S.S. et al., 2020 | 10.1055/s-0039-1678602 | India | Retrospective cohort | Adults and Paediatrics | Trauma | To share experience of a novel and simple alternative to standard post-craniectomy bony defect closure techniques. |  | Yes | Not defined | Reported 11 patients had a wound infection. | No |
| Kumar S. et al., 2022 | 10.4103/0028-3886.344635 | India | Retrospective cohort | Adults and Paediatrics | Neuro-oncology | To compare two methods of hematoma aspiration, craniotomy, and stereotactic aspiration. |  | Yes | Not defined | No difference in surgical site infections between the two methods described. | No |
| Kuwano A. et al., 2023 | 10.1007/s00701-022-05474-6 | Japan | Retrospective cohort | Adult | Neuro-oncology | To investigate the relationship between SSIs and glioma treatment characteristics, such as reoperations, radiation therapy, and chemotherapy. |  | Yes | Requirement of reoperation. SSIs were suspected on the basis of the postoperative wound conditions and MRI findings. | During the observation period, SSIs occurred in 3.1% (31/1012). Three or more surgeries and radiation therapy were associated with SSIs. Intraoperative magnetic resonance imaging was not significantly associated with SSI. | No |
| Lam F.C. et al., 2012 | [10.1227/NEU.0b013e31826a8ab0](https://doi.org/10.1227/neu.0b013e31826a8ab0) | USA | Retrospective cohort | Adult | Skullbase | To report our experience using locally harvested autologous pericranium as a dural substitute in patients who underwent posterior fossa surgeries. |  | Yes | Not defined | No patients experienced surgical site infection. | No |
| Lan M. et al., 2019 | 10.1016/j.wneu.2019.09.148 | USA | Retrospective case control | Adult | Trauma | To describe the population of patients undergoing decompressive craniectomy for traumatic intracranial haemorrhage requiring repeat surgery  To compare outcomes of those requiring repeat surgery to those who did not.  To discern predictors of repeat surgery. |  | Yes | Not defined | Reported 1 case of SSI out of 173 patients. | No |
| Langness S. et al., 2017 | [10.1016/j.amjsurg.2016.06.001](https://doi.org/10.1016/j.amjsurg.2016.06.001) | USA | Retrospective cohort | Adult | Trauma | To validate the mortality risk in a larger patient cohort, stratify risk based on cirrhosis severity, and evaluate the effect of cirrhosis on TBI management. |  | Yes | Not defined | Reported 1 wound infection in non-cirrhosis group and no infections in cirrhosis group. | No |
| Lannon M. et al., 2022 | [10.1097/TA.0000000000003385](https://doi.org/10.1097/ta.0000000000003385) | Canada | Retrospective cohort | Paediatric | Paediatric/ Trauma | To determine the outcomes and prognostic factors in paediatric craniocerebral gunshot injury (CGI) patients. |  | No | Not defined | Rates of infection not directly reported in discussion. | No |
| Lawrence J. et al., 2016 | [10.3171/2016.5.JNS152118](https://doi.org/10.3171/2016.5.jns152118) | USA | Retrospective cohort | Adult | Vascular | To compare complication rates of patients with and without a postoperative ICU stay following microvascular decompression.  To identify predictors of complications, to analyse variables of health care resource utilisation, and to estimate the cost of postoperative management. | . | No | Not defined | Reported 1 case of infection in patients with ICU stay (1.1%) along with 1 wound infection with osteomyelitis. | No |
| Le Guen M. et al., 2023 | [10.1186/s12871-022-01962-5](https://doi.org/10.1186/s12871-022-01962-5) | France | Retrospective cohort | Adult | General (Craniotomy) | To evaluate the efficacy of a goal directed fluid therapy on post-operative major complications, in a before-after multicentric study. | Before vs after the implementation of study’s protocol. | Yes | Not defined | Reported an SSI rate of 1.2% in the before group, and 0.6% rate of infection in the after group (after the protocol was established). | No |
| Lee C.H. et al., 2012 | [10.1097/TA.0b013e318256a150](https://doi.org/10.1097/ta.0b013e318256a150) | South Korea | Retrospective cohort | Adult | Trauma | To identify factors that are related to bone graft infection after cranioplasty. |  | Yes | Requirement the removal of the infected bone graft. | The overall infection rate after cranioplasty was 7.86%. | No |
| Lee J.K. et al., 2012 | [10.3346/jkms.2012.27.12.1563](https://doi.org/10.3346/jkms.2012.27.12.1563) | South Korea | Retrospective cohort | Paediatric | Trauma | To estimate the infection rate of the consecutive shunt series in children over 6-years at a single centre and investigated the risk factors, bacterial pathogens and their antimicrobial susceptibility patterns, the treatment modalities, and clinical outcome. |  | Yes | 1. Identification of a bacterial pathogen from the reservoir CSF/ reservoir CSF pleocytosis (more than 50 leukocytes per cubic millimetre)  2. Positive blood culture  3. Fever  4. Neurologic symptoms  5. Abdominal symptoms  6. Shunt malfunction | Of the 333 inserted shunts, 35 shunts (10.5%) became infected, which represented an infection rate of 0.075 infection cases per shunt per year. | No |
| Lee J.A. et al., 2022 | [10.1016/j.wneu.2022.07.013](https://doi.org/10.1016/j.wneu.2022.07.013) | South Korea | Retrospective cohort | Adults and Paediatrics | Vascular | To analyse the clinical outcomes from 1 to ≥5 years after microvascular decompression in patients with hemifacial spasm. |  | Yes | Not defined | Reported a total of 4 cases of wound infections. | No |
| Lee M.H. et al., 2015 | [10.1007/s10143-015-0666-7](https://doi.org/10.1007/s10143-015-0666-7) | Korea | Retrospective cohort | Adult | Vascular | To identify clinical characteristics of possible complications after microvascular decompression for hemifacial spasm and to establish appropriate management concept of these complications. |  | Yes | Not defined | Five cases had wound infections. | No |
| Lee S. et al., 2019 | 10.1016/j.wneu.2019.06.155 | Korea | Retrospective cohort | Adult | Vascular | To analyse vascular complications in posterior fossa surgery to determine the incidence, risk factors, prognosis, and preventive measures involved. | . | No | Not defined | Surgical wound infection occurred in 1 patient who then underwent a revision surgery. | No |
| Lefebvre J. et al., 2017 | [10.1016/j.jhin.2016.11.019](https://doi.org/10.1016/j.jhin.2016.11.019) | France | Prospective cohort | Adult | Functional (DBS insertion) | To assess whether identification of Staphylococcus aureus (SA) nasal carriers followed by nasal mupirocin ointment and chlorhexidine soap reduced SSI among 182 patients undergoing deep brain stimulation. | Control group vs screening group. | Yes | Centre for Disease Control and Prevention Guidelines | Results demonstrated significant differences between the two groups: one SSI occurred in the screening group (1.6%) compared to 13 infections in the control group (10.9%). SSIs in the control group were due to SA in eight patients (61.5%). | No |
| Lemee J.-M. et al., 2019 | 10.1016/j.wneu.2019.05.010 | Norway | Retrospective cohort | Adults and Paediatrics | Neuro-oncology | To describe the early complications after surgery intervening in a large population-based cohort of consecutive patients undergoing resection of intracranial meningiomas. |  | No | Not defined | A total of 38 patients (2.6%) had a postoperative infection of any type requiring reoperation. | No |
| Lepanluoma M. et al., 2015 | 10.3171/2014.12.JNS141077 | Finland | Retrospective case-control | Adults and Paediatrics | General (All neurosurgical cases) | To determine whether the use of the WHO surgical checklist would have an impact on the number and causes of neurosurgical complications leading to a reoperation. | With vs without the use of the WHO surgical checklist | Yes | Not defined | There was a significant decrease in the rate of preventable infections associated with reoperations with the use of the WHO surgical checklist. | No |
| Lepski G. et al., 2021 | 10.1016/j.clineuro.2021.106599 | Brazil | Retrospective cohort | Adult | Neuro-oncology | To analyse the risk factors for infection and categorize patients according to risk rate. |  | Yes | Within 30 days after surgery, divided into 3 major categories:  1. Superficial/ wound infections  2. Meningitis  3. Deep/ intracranial (empyema or intracerebral abscess). Meningitis was confirmed if the patient had a confirmed or probable diagnosis. | SSI or meningitis was observed in 53 of the 987 enrolled patients (5.37%). 16% were superficial or wound infections, 40% meningitis and 44% deep or intracranial infections. | No |
| Leung G.K. et al., 2007 | 10.1080/02688690701392881 | Hong Kong | Retrospective cohort | Adults and Paediatrics | Trauma | To report experience of external ventricular drainage (EVD) with extended subcutaneous tunnel (‘long EVD’) in terms of infection rate and predisposing risk factors. |  | Yes | Positive CSF culture | 7 cases of infection were reported. | No |
| Levi V. et al., 2020 | 10.1093/ons/opz118 | Italy | Retrospective case-control | Adult | Functional (DBS insertion) | To assess whether or not this novel technique involving IPG catheterisation was more effective than the previous infection management strategy in increasing the rate of intracranial lead salvage after initial partial DBS hardware removal. | With vs without IPG catheter | Yes | Guideline for Prevention of Surgical Site Infection | Twenty-three patients (4%) met the inclusion criteria for DBS hardware-related infections. | No |
| Lewis A. et al., 2017 | 10.3171/2016.4.JNS16275 | USA | Retrospective case-control | Adult | Trauma | To determine the effects of eliminating the use of prolonged prophylactic systemic antibiotics (PPSAs) in patients with subdural and subgaleal drains. | PPSA vs non PPSA group | Yes | Not defined | The discontinuation of PPSAs did not result in an increase in the frequency of SSI. | No |
| Li S. et al., 2024 | 10.1016/j.jclinane.2023.111285 | China | Prospective case-control | Adults and Paediatrics | Neuro-oncology | To test the hypothesis that tranexamic acid (TXA) does not meaningfully increase the risk of postoperative seizures within 7 days after intracranial tumour resections. | With and without TXA | Yes | Not defined | Reported 28 infections in the normal saline group, 26 in the TXA group. | No |
| Li T. et al., 2024 | 10.1007/s10143-024-02287-2 | China | Retrospective cohort | Adult | General (Craniotomy) | To investigate the impact of postoperative changes in natremia on outcomes specifically in patients undergoing craniotomy. |  | Yes | Not defined | No association between postoperative sodium increase and SSI. | No |
| Li Y. et al., 2020 | 10.1016/j.bj.2020.06.004 | Taiwan | Retrospective cohort | Adult | Neuro-oncology | To assess the general performance and neurological outcomes of patients with recurrent gliomas who underwent awake craniotomy for glioma resection. |  | No | Not defined | Five (out of 225) patients in the general anesthesia group developed SSI postoperatively. | No |
| Lieber B.A. et al., 2016 | 10.3171/2015.4.JNS142719 | USA | Retrospective cohort | Adult | General (All cranial neurosurgical cases) | To determine whether chemotherapy and prolonged steroid use before surgery increase the risk of an SSI at postoperative Day 30. |  | Yes | Centre for Disease Control and Prevention Guidelines | Reported 158 SSIs (out of 8215 patients) at 30 days, of which 52 were superficial, 27 were deep-incisional, and 79 were organ-space infections. Preoperative chemotherapy was an independent predictor of organ-space SSIs along corticosteroid use. Longer duration of operation, wound class of ≥ 2 (clean-contaminated and further contaminated) and morbid obesity were also predictors of organ-space SSIs. | No |
| Linzey J.R. et al., 2017 | 10.1093/neuros/nyx046 | USA | Retrospective case-control | Adult | Vascular | To determine if frontal sinus breach is a risk factor for developing cranial SSIs in patients undergoing craniotomies for clip ligation of anterior circulation aneurysms. |  | Yes | 1. Erythema,  2. Purulent  discharge  3. Tenderness,  4. Fever and other common signs of infection  5.Prescription of antibiotics  for a wound infection | 78 (8.6%) of the 910 craniotomies developed cranial SSIs. Cranial SSIs were significantly associated with increased length of surgery and an increased duration of clinical follow-up. | No |
| Liu B. et al., 2018 | 10.1016/j.clnu.2018.11.008 | China | Randomised controlled study | Adult | General (Craniotomy) | To evaluate the effect of preoperative oral carbohydrate loading versus fasting on the outcomes of patients undergoing elective craniotomy. | Use of pre-operative oral carbohydrate loading vs fasting. | Yes | Not defined | Reported 1 case SSI in intervention group and 2 cases in control group with no significant difference between the two groups. | No |
| Liu L. et al., 2018 | 10.1016/j.wneu.2018.06.238 | China | Prospective cohort | Adult | Vascular | To identify potential prognostic factors of hemifacial spasm (HFS) after microvascular decompression (MVD), to establish the appropriate way to tackle post-procedure symptoms and complications (PPSCs), and to find the incidence and duration of PPSCs. |  | Yes | Not defined | The incidence of infection was 2.0%. | No |
| Liu M. et al., 2020 | 10.1055/s-0040-1710520 | China | Retrospective | Not reported | Vascular | To assess and suggest improvements for the postoperative outcome of microvascular decompression (MVD). |  | No | Not defined | Incisional infection occurred in 7 cases (1%) of traditional MVD group and 5 cases (0.87%) in MVD plus group. | No |
| Liu W. et al., 2021 | 10.1055/s-0040-1719138 | China | Retrospective cohort | Adult | Vascular | To evaluate the effect of using autologous particulate bone to reconstruct the cranial defect produced by microvascular decompression. |  | No | Wound redness, pain, suppuration, or subcutaneous tissue necrosis at the surgical site. | A total of 15 patients developed SSIs. The SSI rate of the first group was 9.92%, while that of the second group was 1.79% hence bone dust may  result in a higher incidence of SSI. | No |
| Liu Y. et al., 2014 | 10.1016/j.clineuro.2014.08.002 | China | Retrospective cohort | Adult | Neuro-oncology | To elucidate clinical and prognostic characteristics of the midline suprasellar meningiomas based on their origin and growth pattern. |  | Yes | Not defined | 15 intracranial infections recorded. | No |
| Loayza R. et al., 2023 | 10.1007/s00701-023-05642-2 | Sweden | Retrospective cohort | Adult | Vascular | To investigate the outcome after microvascular decompression and whether it is affected by neurovascular conflict severity and sex. |  | No | Not defined | Out of 109 patients, two wound infections (1.8%). | No |
| Logghe H. et al., 2015 | PMID: 25760209 | USA | Retrospective cohort | Adult | Trauma | To compare laparoscopic-assisted shunt revision with first-time shunt placement, focusing on operative time and complications. | Shunt revision vs first-time shunt placement | No | Not defined | No cases of SSI were reported in the first-time shunt group, but 1 case occurred in the revision group. | No |
| Lopez D.T. et al., 2023 | 10.25259/SNI_673_2023 | Multiple | Retrospective cohort | Adult | Functional (DBS insertion) | To evaluate the long-term outcome of Parkinsonian patients, including the duration of improvement after DBS surgery, operated on using an image-guided stereotaxys followed by intraoperative macrostimulation. |  | No | Not defined | Reported one patient developed infection of the surgical scar. | No |
| Lu Y. et al., 2022 | [10.3389/fnins.2022.917752](https://doi.org/10.3389/fnins.2022.917752) | China | Retrospective cohort | Adult | Functional (DBS insertion) | To compare the effects of local anaesthetic and general anaesthetic operation methods on clinical improvement in patients with Parkinson’s Disease, after DBS surgery. |  | No | Not defined | No intracranial infection occurred in either group after DBS lead placement. Delayed skin incision infection occurred in two cases in each group. | No |
| Luther E. et al., 2020 | 10.1007/s00701-020-04239-3 | USA | Retrospective cohort | Adult | Neuro-oncology | To evaluate SSIs and perioperative complications associated with the use of an absorbable intradermal barbed suture as a means for skin closure in hair-sparing supratentorial craniotomies for tumour. | Absorbable intradermal barbed suture vs other methods of closure | Yes | 1. Wound breakdown  2. CSF leak requiring readmission for antibiotics or reoperation. | The hair-sparing technique with Stratafix was not significantly correlated with surgical complications on univariable or multivariable analysis. | No |
| Lv Y. et al., 2023 | 10.1186/s41016-023-00336-1 | China | Retrospective case-control study | Adult | Neuro-oncology | To determine the relevant risk factors associated with SSI after elective craniotomy for brain tumour and analyse the treatments for SSI. |  | Yes | A purulent discharge requiring local debridement or osteomyelitis/ abscess requiring reoperation, as well as positive cultures from secretion samples or CSF. | Reported 31 out of 2061 patients had SSI (1.50%). Body mass index and operative duration were identified as independent risk factors for SSI. | Yes: “In numerous studies, the rates of SSI after neurosurgery  varied due to differences in patient characteristics, operation indication, follow-up time, and SSI criteria. Consequently, comparing SSI incidence across studies with different criteria and demographics  should be done with caution. Defning a universal diagnostic criterion for SSI after craniotomy is both worthwhile and urgent. Researchers have debated the classification and definition of SSIs.” |
| Lwin S. et al., 2012 | PMID: 22511048 | Singapore | Retrospective cohort | Adults and Paediatrics | Trauma | To reduce the incidence of external ventricular drain-related infection, including ventriculitis in neurosurgical patients. |  | Yes | Positive CSF culture | Following the institution of the new protocols, the EVD infection rate was reduced to 3.8% (three out of 79 patients). | No |
| Maayan O. et al., 2022 | 10.1007/s00701-021-05075-9 | USA | Retrospective case-control | Adults and Paediatrics | General (Craniotomy) | To identify risk factors for infection and assess the efficacy of prophylactic betadine irrigation and vancomycin powder in addition to standard antibiotic irrigation. | Group 1: antibiotic irrigation, Group 2: antibiotic irrigation and betadine irrigation, Group 3: antibiotic irrigation, betadine irrigation, and vancomycin powder. | Yes | Positive bacterial culture | Of the 7 patients who received preoperative bevacizumab 4 (57.1%) had a postoperative infection. Other factors significantly associated with wound infection were foreign body placement with an infection rate of 9.9% | No |
| Maayan O. et al., 2023 | 10.1007/s11060-023-04294-7 | USA | Retrospective cohort | Adult | Neuro-oncology | To assess risk factor aetiologies for SSI and investigate their combinatorial effects on infection rate following craniotomy for neuro-oncologic pathology. |  | Yes | Not defined | Postoperative SSI occurred in 20 patients (1.7%) within 30 days and 42 patients (3.5%) by 90 days. | No |
| Madhugiri V.S. et al., 2011 | 10.1159/000330542 | India | Retrospective cohort | Paediatric | Paediatric | To conduct a comprehensive analysis of factors affecting recurrence and outcome of all the focal intracranial infections in children. |  | No | Not defined | There was a wound infection rate of 7% in the infratentorial empyema group and 2 patients developed wound infection in the supratentorial empyema group. | No |
| Magil S.T. et al., 2023 | 10.1227/neu.0000000000002569 | USA | Retrospective cohort | Adults and Paediatrics | Neuro-oncology | To collect data on trends in approach selection and surgical outcomes of visual worsening, extent of resection, surgical complications, and recurrence rates |  | No | Not defined | 29 patients had a wound infection requiring surgery. 3 patients had superficial infection treated with antibiotics. | No |
| Mahboubi H. et al., 2016 | 10.1097/MAO.0000000000001178 | USA | Retrospective cross-sectional | Adult | Neuro-oncology | To investigate the 30-day postoperative complication, readmission, and reoperation rates following surgery for cerebellopontine angle (CPA) schwannomas. |  | Yes | Not defined | Reported wound infections being a common complication including surgical-site infection and wound dehiscence (11 patients, 2.7%). | No |
| Makoshi Z. et al., 2022 | 10.3171/2022.7.PEDS22231 | USA | Retrospective and prospective. | Adult | Skullbase | To test the hypothesis that complication rates increase with the use or combination of certain sealants and grafts. |  | Yes | Not defined | Higher rates of SSI were reported in DuraSeal group. | No |
| Mallela A. et al., 2017 | 10.1093/neuros/nyx559 | USA | Prospective cohort | Adult | General (Craniotomy) | To examine the efficacy of topical vancomycin applied within the wound during craniotomy. | Application of topical vancomycin. | Yes | Positive culture within 120 days of surgery. | The addition of topical vancomycin was associated with a significantly lower rate of SSI than standard of care alone (0.49% [1/205] vs 6% [9/150]). | No |
| Mallela A.N. et al., 2020 | [10.1016/j.jocn.2020.07.048](https://doi.org/10.1016/j.jocn.2020.07.048) | USA | Retrospective cohort | Adult | Neuro-oncology | To identify pre-operative and post-operative risk factors are predictive of 30-day readmission and develop a user-friendly, additive readmission risk score. |  | Yes | Not defined | Reported an SSI rate of 6.9%. | No |
| Mann C. et al., 2021 | 10.1016/j.yebeh.2020.107715 | Germany | Retrospective cohort | Both | Functional (Resective or ablative surgical treatment for epilepsy) | To describe the patients’ characteristics, surgical ratio, and outcomes following epilepsy surgery at the newly established Epilepsy Center Frankfurt Rhine-Main. |  | No | Not defined | In 21 cases, perioperative complications occurred. 7 of these cases were of postoperative surgical site infections or delayed wound healing. | No |
| Marcus L.P. et al., 2014 | 10.3171/2014.1.JNS131264 | USA | Retrospective cohort | Adult | Neuro-oncology | To determine the 30-day readmission rate for patients undergoing the resection of primary brain tumours and to identify which factors predispose certain patient groups to rehospitalisation. |  | No | Not defined | Reported a 14.5% SSI rate. | No |
| Martin A.J. et al., 2017 | 10.3171/2015.7.JNS15750 | USA | Retrospective cohort | Adult | Functional (DBS insertion) | To assess the incidence of postoperative hardware infection following interventional MRI–guided implantation of deep brain stimulation (DBS) electrodes in a diagnostic MRI scanner. |  | Yes | Not defined | Hardware infection within 6 months of surgery occurred  in 6 patients, for an overall postoperative hardware infection rate of 3.6%. | No |
| Mascarenhas L. et al., 2014 | 10.1016/j.wneu.2013.02.032 | USA | Retrospective cohort | Adult | Skullbase/ Neuro-oncology | To present a large series of patients and examine the learning curve of the endonasal endoscopic transplanum, transtuberculum approach for primarily suprasellar or sellar-suprasellar tumours. |  | No | Not defined | One infection out of 122 patients reported. | No |
| Maye H. L. et al., 2022 | 10.1016/j.wneu.2022.02.124 | UK | Retrospective cohort | Adults and Paediatrics | Neuro-oncology | To identify whether the use of surgical adjuncts such as iUS, 5-ALA, and neurophysiologic monitoring in supratentorial tumour surgery led to increased operative time or increased rates of SSI at 30 days and 4 months. |  | Yes | 1. Skin and soft-tissue infection,  2. Bone flap osteomyelitis,  3. Subdural empyema,  4. Brain abscess or meningitis, at either 30 days (early) or 4 months (delayed). | Early (occurring before 30 days) and late infections (occurring between 30 days and 4 months) were observed in 10 (4%) and 2 (1%) patients, respectively. The overall infection rate was 4.5% (12/267). | No |
| McClelland S. et al., 2007 | 10.1086/518580 | USA | Retrospective cohort | Adult | General (All neurosurgical cases) | To establish the incidence of postoperative central nervous system infections (PCNSI) in the population of patients undergoing neurosurgical procedures and the potential risk factors. |  | Yes | Meningitis, epidural abscess, subdural empyema, and/or brain abscess. | Of the 1587 cranial operations, 14 (0.8%) were complicated by PCNSI. Infection of an indwelling device comprised 5 cases of PCNSI (35.7% of cranial infections), and 4 cases of PCNSI manifested as meningitis (28.6% of cranial infections). 3 cases manifested as brain abscess, 1 case of superficial wound infection and 1 case of subdural empyema. | No |
| McCutcheon B.A. et al., 2015 | 10.1016/j.wneu.2015.12.068 | USA | Retrospective cohort | Adult | Neuro-oncology | To determine the rate of SSI following resection of an intracranial neoplasm using the American College of Surgeons National Surgical Quality Improvement Program (NSQIP) dataset. |  | Yes | Superficial incisional SSI, deep incisional SSI, or organ/ space SSI within 30 days of the procedure, without occurrence of wound dehiscence. NSQIP defines an organ/ space SSI as any SSI that involves the anatomy that was manipulated other than the incision. | SSI occurred at a rate of 2.04%. SSI was significantly associated with increased rates of return to the operating room and prolonged postoperative lengths of stay greater than 30 days. Recent chemotherapy was associated with an increased odd of SSI. | Yes: "other studies may have employed slightly different methodologies  with regard to defining and identifying cases of surgical site infection." |
| McCutcheon B.A. et al., 2016 | 10.1016/j.wneu.2016.01.089 | USA | Retrospective cohort | Adult | Neuro-oncology | To evaluate the association between patient age and perioperative complications among patients undergoing surgical resection of a benign cranial nerve neoplasm. |  | Yes | Not defined | 1.59% rate of SSI reported. | No |
| McGirt M.J. et al., 2003 | 10.1086/368191 | USA | Retrospective cohort | Paediatric | Paediatric/ CSF dynamics | 1. To identify independent risk factors for CSF shunt infection, and 2. To identify independent clinical predictors of the causal bacterial pathogen. |  | Yes | National Nosocomial Infection Surveillance System: CSF culture that yielded a pathogenic organism or indicated CSF pleocytosis (150 leukocytes/mm3 ) associated with fever (temperature, >38.5C), shunt malfunction, or neurological symptoms. | Ninety-two shunts (11%) developed infection a median of 19 days after insertion. Premature birth, previous shunt infection, and intraoperative use of the neuroendoscope were risk factors for shunt infection. | No |
| McGirt M.J. et al., 2009 | 10.1227/01.NEU.0000349763.42238.E9 | USA | Retrospective cohort | Adult | Neuro-oncology | To determine whether new-onset postoperative motor or speech deficits were associated with survival in authors’ institutional experience with glioblastoma multiforme (GBM). | patients who had experienced surgically acquired motor or language deficits versus those who did not experience these deficits | No | Not defined | Reported that 2 patients had SSI. | No |
| Mehta G. et al., 2022 | 10.3171/2021.5.JNS21772 | USA | Retrospective cohort | Adult | Skull base/ Neuro-oncology | To better define the utility of surgical management of sinonasal malignancies and to determine factors related to outcome. |  | No | Not defined | Infections included 2 frontal lobe abscesses requiring drainage, 1 patient with cerebritis managed with antibiotics, 2 patients with superficial wound infections managed with antibiotics, 1 patient with facial cellulitis that required craniectomy and wound washout at 6 months after surgery, and 1 patient with bilateral facial abscesses related to midfacial degloving that required incision and drainage 2 weeks after surgery. | No |
| Meng Y. et al., 2018 | 10.3171/2018.1.PEDS17476 | Canada | Retrospective cohort | Paediatric | Paediatric | To examine the rate of SSI after intracranial electroencephalography (iEEG) monitoring for epilepsy workup in pediatric patients and to determine the variables that might contribute to the development of SSI. |  | Yes | Any postoperative infection involving the wound, skull, or central nervous system (meningitis, epidural abscess, osteomyelitis). | Out of a total of 199 patients, 8 (4.0%) developed SSIs within a period ranging from 21 to 51 days postoperatively. The number of people present in the operating room on electrode insertion, length of insertion surgery, previous operation at the same surgical site, and number of depth electrodes inserted were risk factors for SSI. | No |
| Mian S.Y. et al., 2023 | 10.1016/j.wneu.2023.06.091 | UK | Retrospective case-control | Adults and Paediatrics | Trauma | To compare external ventricular drains (EVDs) with percutaneous continuous cerebrospinal fluid (CSF) drainage via ventricular access devices (VADs) for the acute management of hydrocephalus in adults. | EVD vs ventricular access devices for CSF drainage. | Yes | 1. Clinical signs of infection, such as fever, meningism, or altered conscious level, WITH  2. Raised serum inflammatory markers (raised CRP or raised white cell count) PLUS  5. A positive CSF culture  6. Positive CSF gram stain  7. Raised CSF white cell count | 5 vs 13 infections in EVD vs VAD reported. | No |
| Miller J.J. et al., 2001 | 10.1097/00129492-200111000-00033 | USA | Retrospective cohort | Adults and Paediatrics | General (All neurosurgical cases) | To determine whether the lack of shaving increased the postoperative infection rate. | Hair-shaved vs hair not shaved. | Yes | Erythema, purulent discharge, fluid collection | The total number of wound infections was 6 (6%) in the shaved patients versus 11 (7.3%) in the unshaved patients. | No |
| Missios S. et al., 2015 | 10.1016/j.wneu.2015.04.052 | USA | Retrospective cohort | Adults and Paediatrics | Neuro-oncology | To create a predictive model of perioperative complications in patients undergoing craniotomies for glioma resection. |  | Yes | Not defined | 0.8% risk of deep wound infection reported. | No |
| Mohamad S. et al., 2016 | 10.21315/mjms2016.23.5.11 | Malaysia | Prospective cross-sectional | Adults and Paediatrics | General (Cranioplasty) | To determine the incidence of graft infection after cranioplasty procedures and factors affecting the graft infection rate at Hospital Kuala Lumpur (HKL). |  | Yes | Centre for Disease Control and Prevention Guidelines | A total of five infected grafts were identified among the 172 cases, resulting in an overall infection rate of 2.9%. Of this infected group, three (4.5%) were cases of cranioplasty and two (1.9%) were cases of autologous bone flap replacement. | No |
| Moiraghi A. et al., 2021 | 10.3390/cancers13122911 | France | Retrospective cohort | Adult | Neuro-oncology | To assess the feasibility and safety (intraoperative findings, postoperative complications, and outcomes), and efficacy (access to adjuvant radiochemotherapy, progression-free survival, and overall survival) of awake surgery of glioblastoma by comparing it to asleep surgery using case matching, and by stratifying according to the neurosurgeon’s experience. | Awake group vs asleep | No | Not defined | SSI more common in asleep resection group than in other subgroups. | No |
| Mooney MA et al., 2018 | 10.3171/2017.5.JNS17394 | USA | Prospective case-control | Adult | Vascular | To analyse outcomes of patients with acutely ruptured saccular aneurysms who were treated with microsurgical clipping in a prospectively collected database from the Barrow Ruptured Aneurysm Trial. | Overlapping vs non-overlapping | Yes | Not defined | Wound infection rates were similar between the 2 groups. | No |
| Moorthy R.K. et al., 2013 | 10.3109/02688697.2013.771138 | India | Prospective cohort | Adults and Paediatrics | General (All neurosurgical cases except trauma) | To audit the efficacy of a conservative prophylactic antibiotic policy in patients undergoing non-trauma cranial surgery. |  | Yes | Not defined | 110 patients developed meningitis, with 27 developing bacterial meningitis. | No |
| Morton R. et al., 2018 | 10.3171/2016.11.JNS161917 | USA | Retrospective cohort | Adults and Paediatrics | General (Cranioplasty) | To present the largest study to date on complications after cranioplasty, focusing specifically on the relationship between complications and timing of the operation. | . | Yes | Not defined | 24.6% of the patients experienced at least 1 complication including infection necessitating explantation of the flap (6.6%). The rate of infection was significantly higher if the cranioplasty had been performed <14 days after the initial craniectomy. | No |
| Morton R.P. et al., 2016 | 10.3171/2015.8.JNS151390 | USA | Retrospective cohort | Adults and Paediatrics | General (Cranioplasty) | To report the largest study on predictors of infection after cranioplasty and to assess the predictive value of intraoperative bone flap cultures before cryopreservation. |  | Yes | Infection resulting in removal of the bone flap. | The overall infection rate after cranioplasty was 6.6% (50 cases) occurring at a median postoperative day 31. Cranioplasty material did not affect the post-cranioplasty infection rate. | No |
| Mracek J. et al., 2015 | 10.1007/s00701-014-2333-0 | Czech Republic | Retrospective cohort | Adults and Paediatrics | Trauma | To evaluate the morbidity associated with cranioplasty using an autologous bone flap sterilised in an autoclave. |  | Yes | Not defined | SSIs occurred in only five patients (3.3%). | No |
| Muir M. et al., 2019 | 10.1016/j.jocn.2019.08.059 | USA | Retrospective cohort | Paediatrics | Paediatrics/ Vascular | To describe 30-day outcomes following craniotomy for arteriovenous malformation in children and identify risk factors for readmission, reoperation, and perioperative complication using the National Surgical Quality Improvement Program (NSQIP) Paediatric database. |  | Yes | Not defined | The incidence of wound infection and/or dehiscence was 4%. | No |
| Muram S. et al., 2023 | 10.3171/2022.5.JNS22430 | Canada | Prospective cohort | Adult | Trauma | To use quality improvement (QI) methodology to create a standardised infection prevention bundle aimed at reducing the rate of shunt infections. |  | Yes | Canadian Nosocomial Infection Surveillance Program (CNISP) guidelines | There were 11 shunt infections that occurred during the study period. Four of these infections were during the period in which there was no protocol and 7 occurred after the implementation of the initial standardised infection prevention bundle. | No |
| Murphy M.E. et al., 2016 | 10.1016/j.clineuro.2016.06.020 | USA | Retrospective cohort | Adult | Neuro-oncology | To analyse postoperative morbidity as well as secondary outcomes of readmission and reoperation. |  | Yes | Not defined | 9 surgical site infections (1.59%) were reported. | No |
| Musavi L. et al., 2020 | 10.1097/SCS.0000000000005695 | USA | Retrospective cohort | Adult | Skullbase | To assess infection rates and management practices across the literature to highlight trends and variations in the perioperative care of craniosynostosis patients |  | Yes | Not defined | The most common post-operative complication was SSI. 6 primary reconstruction patients (1.09%) and 9 secondary reconstruction patients (5.52%) developed an SSI requiring extended hospital stay or readmission, which was statistically significant. | No |
| Nair S. et al., 2023 | 10.1227/ons.0000000000000819 | USA | Retrospective cohort | Adult | Vascular | To directly compare pain outcomes in patients undergoing primary microvascular decompression (MVD) vs those undergoing MVD with a history of 1 prior stereotactic radiosurgery (SRS) procedure. |  | No | Not defined | There were no significant differences in the frequency of CSF leak or postoperative SSIs between the SRS-MVD and primary MVD groups. Reported 22 cases of postop SSI (2.76%) in primary MVD and 2 cases post-op SSI (5.41%) in SRS-MVD. | No |
| Nair S.K. et al., 2023 | 10.3171/2022.8.JNS212799 | USA | Retrospective cohort | Adult | Neuro-oncology | To identify the relationship between preoperative predictors inclusive of scalp incision type and postoperative SSI following glioblastoma resection. |  | Yes | Wound breakdown, the presence of bacteria in wound drainage, or the need for reoperation for wound washout. | Reported 30 out of 911 cases (3.3%) demonstrated postoperative SSI. There were no significant differences in preoperative malnutrition or number of surgeries between SSI and non-SSI cases. | No |
| Nguyen A.V. et al., 2019 | 10.1016/j.clineuro.2019.05.017 | USA | Retrospective cohort | Adults and Paediatrics | General (All neurosurgical cases) | To evaluate the association of prophylaxis choice and incidence of SSI at authors’ own institution. | Cefazolin vs Vancomycin use | Yes | Positive culture isolated from the wound, implant, or CSF within a year of surgery. | Reported 22 SSIs, with 14 in the cefazolin (2.2%) and 8 in the vancomycin (4.1%) group. | No |
| Northam W. et al., 2020 | 10.1177/1460408619892141 | USA | Retrospective cohort | Adult | Trauma | To identify factors associated with length of stay after cranioplasty to better understand their outcomes. |  | Yes | Not defined | Reported 24.4% of deep wound infection and 4.4% of superficial wound infection. | No |
| Nunno A. et al., 2018 | 10.1016/j.wneu.2018.11.091 | USA | Retrospective cohort | Adult | Neuro-oncology | To assess occurrence of VTE in patients who underwent surgical resection of meningioma to determine risk factors and associated complications of VTE. |  | No | Not defined | SSI cases were associated with 4% VTE cases post-operatively (not statistically significant). | No |
| Nusair A.R. et al., 2021 | 10.1089/sur.2020.020 | UAE | Retrospective cohort | Adults and Paediatrics | General (All neurosurgical cases) | To identify and decolonise MRSA carriers and to tailor perioperative antibiotic prophylaxis to protect those at high risk for SSIs better. |  | Yes | Centre for Disease Control and Prevention Guidelines | Infection rate decreased from 2.8% to 1.8% after implementation of the protocol. | No |
| O'Keeffe A. et al., 2012 | 10.3109/02688697.2011.626878 | UK | Retrospective cohort | Adult | General (Craniotomy) | To derive an estimated cost for each instance of infected craniotomy. | . | Yes | Centre for Disease Control and Prevention Guidelines | Reported a total of 245 craniotomies and 20 verified craniotomy infections. An overall infection rate of 8% is identified, and the cost incurred as a result of craniotomy infections is estimated at £1 85 660 for the 10-month period studied. | No |
| Oakley G.M. et al., 2018 | 10.1017/S0022215117001499 | USA | Retrospective cohort | Adult | Skullbase | To describe the short- and long-term outcomes of collagen matrix in skull base reconstruction. |  | Yes | Not defined | 1 intracranial infection reported. | No |
| Oh W.O. et al., 2018 | 10.1159/000481437 | South Korea | Retrospective case-control | Adult | General (Craniotomy) | To analyse the data of patients who underwent unshaven cranial surgery with absorbable sutures for scalp closure. To discuss techniques for neurosurgical site preparation and skin closure materials for cranial operations with an emphasis on patient comfort and quality of life. | Unshaven cranial surgery with absorbable sutures for scalp closure Vs shaved group | Yes | 1. Discharge,  2. Tenderness,  3. Swelling,  4. Fluid collection,  5. Redness of the surgical site | Intervention group: 2 patients experienced superficial SSIs that were resolved within 14 days using dressings and oral antibiotics.  Control group: 2 patients experienced superficial SSIs at postoperative 14 days. | No |
| Okunlola A.I. et al., 2021 | 10.1080/02688697.2020.1812518 | Nigeria | Retrospective case control | Adult | General (Craniotomy) | To determine whether intraoperative wound irrigation with ceftriaxone provides additional prevention of SSI in patients already receiving the drug parenterally. | Intra-operative wound irrigation with a ceftriaxone-in-normal saline solution Vs control group irrigated with only normal saline. | Yes | Grade 0: normal healing;  Grade I: normal healing with mild erythema or epidermolysis;  Grade II: superficial wound infection with galeal/ fascia intact;  Grade III: deep wound infection below the galeal/ fascia but with intact dural  IIIa: no osteomyelitis,  IIIb: with osteomyelitis and  IIIc: with pachy meningitis;  Grade IV: meningitis without tissue breakdown excluding chemical meningitis;  Grade V: meningitis with breakdown of dural and fascia;  Grade VI: intracranial or intraspinal intradural abscess;  VIa: subdural empyema,  VIb: intraparenchymal abscess,  VIc: intraventricular abscess,  VId: combination | The overall frequency of SSI was 2.27% (3 out of 132). The incidence among ceftriaxone group was 3% (2 out of 66) and among the control group 1.5% (1 out of 66). | No |
| Onkarappa S. et al., 2023 | 10.1016/j.wneu.2023.05.104 | India | Prospective | Adults and Paediatrics | General (Cranioplasty) | To note the overall complication rates of cranioplasty after decompressive craniectomy and more specifically compare complications between 2 different time intervals. |  | Yes | Not defined | A total of seven (6.7%) complications were observed in 6 patients. Among them, infectious complications formed the majority (n 1⁄4 5,71%). This involved 3 cases of bone flap osteomyelitis and 2 cases of wound infections. | No |
| Ormond D.R. et al., 2019 | 10.1093/neuros/nyy125 | Germany | Retrospective cohort | Paediatric | Paediatric | To present the largest case series of paediatric (temporal lobe epilepsy) patients thus far, in order to better understand the predictability of preoperative evaluation on seizure outcome, and to better understand longitudinal outcomes in a large paediatric cohort. |  | No | Not defined | Reported 2 wound infections requiring revision (1.1%), | No |
| Orsi G.B. et al., 2006 | 10.1016/j.jhin.2006.02.022 | Italy | Prospective cohort | Adult | General (All cranial neurosurgical cases) | To identify hospital acquired infection rates and associated risk factors in Neurosurgical ICUs. |  | Yes | Centre for Disease Control and Prevention Guidelines | SSI rate after a craniotomy was 5.8%. | Yes |
| Osbun J. et al., 2012 | 10.1016/j.wneu.2011.12.011 | USA | Randomised controlled trial | Adult | General (All cranial neurosurgical cases) | To further evaluate the safety of a PEG hydrogel compared with common dural sealing techniques. | PEG hydrogel Vs Common dural sealing technique (Control). | Yes | Centre for Disease Control and Prevention Guidelines | The incidences of neurosurgical complications, SSIs, and CSF leaks were similar between treatment and control groups, with no statistically significant difference between the measures. In the PEG hydrogel group, 2 out of 120 patients developed SSI (1.7%). In the control group, 3 out of 117 patients developed SSI (3%). | No |
| Ozkan U. et al., 2002 | 10.1007/s101430100173 | Turkey | Retrospective cohort | Adult | Trauma | To analyse treatment techniques of civilian craniocerebral gunshot wounds. |  | Yes | Not defined | 3 cases of intracranial infection reported. | No |
| Paredes I. et al., 2020 | 10.1007/s00701-020-04508-1 | Spain | Retrospective cohort | Adult | General (Cranioplasty) | To measure the effect of tailored antibiotic prophylaxis on SSIs resulting from cranioplasties. | Old vs new protocol for antibiotic prophylaxis. | Yes | Centre for Disease Control and Prevention Guidelines | Of the 109 cranioplasties, 16 (14.7%) suffered an infection, 14 (21.9%) in the old protocol group and 2 (4.4%) in the new protocol group. Multiple surgeries and previous infection were risk factors for SSI. Of the bacteria identified in the skin of the scalp, 22.2% were resistant to routine prophylaxis (cefazoline). | No |
| Park Y. et al., 2011 | 10.1159/000324903 | Korea | Prospective | Adult | Functional (DBS insertion) | To introduce a combination procedure with double C-shaped skin incision and an adjusted dual-floor burr hole to prevent skin complications on the scalp with deep brain stimulation (DBS) surgery. |  | No | Not defined | Reported eight scalp erosions associated with infection (3.0%) among 268 patients. | No |
| Patel A.J. et al., 2014 | 10.3171/2014.1.PEDS13372 | USA | Retrospective cohort | Paediatric | Paediatric | To evaluate impact of comorbidities on perioperative complications in paediatric neurosurgery. | No intervention | Yes | Not defined | 113 cases of infection | No |
| Patel K.S. et al., 2014 | 10.1016/j.clineuro.2013.12.015 | USA | Retrospective case-control | Adults and Paediatrics | General (Craniotomy) | To evaluate the efficacy of betadine irrigation in preventing postoperative wound infection in cranial neurosurgical procedures. | antibiotic vs betadine + antibiotic irrigation | Yes | Any culture proven infection occurring within 30 or 90 days of surgery that required reoperation. | 2.56 vs 3.83 infection rate with and without betadine within 90 days | No |
| Pattavilakom A. et al., 2007 | 10.1016/j.jocn.2006.11.003 | Australia | Prospective cohort | Adults and Paediatrics | CSF dynamics | To report the impact of AIC (antibiotic impregnated CSF shunt catheters) on shunt infection. |  | Yes | Not defined | There were three shunt infections (1.2%). Rigorous retrospective evaluation of shunt procedures over the preceding 7 years revealed 36 infections in 551 shunt procedures (6.5%). This reduction in the infection rate was statistically significant. We also report that the introduction of ceftriaxone prophylaxis during this period was associated with a reduction in Gram-negative shunt infection, but no effect on overall infection rate. | No |
| Pavlicevic G. et al., 2017 | 10.1016/j.jcms.2016.11.019 | Serbia | Retrospective cohort | Adult | Trauma | To assess the effect of various factors on results of cranial defect reconstruction after combat injuries. | autologous flap vs allograft | Yes | Not defined | postoperative infection was most common, with 15 such cases. More common with allografts. | No |
| Pereira J. et al., 2012 | 10.4103/2152-7806.99941 | Brazil | Prospective cohort | Adult | Vascular | To evaluate the safety and efficacy of performing craniotomy with minimal hair removal and closure with intradermal suture alone compared with continuous skin sutures with 2-0 nylon. | Closure with intradermal suture alone Vs continuous sutures with nylon. | Yes | Not defined | The case group was composed of 49 patients in whom just intradermal suture was performed. One (2.2%) patient developed wound infection. The control group was composed of 68 patients in whom the skin was closed with 2-0 nylon continuous suture. Three (5.3%) patients developed wound infection. There was no statistically significant difference in the number of wound infections between the two groups. | No |
| Pfnur A. et al., 2024 | 10.1007/s10143-024-02309-z | Germany | Retrospective cohort | Adult | General (Craniotomy) | To compare different implant materials for cranioplasty in terms of postoperative complication rates. |  | Yes | 1. Wound dehiscence  2. Abscess  3. Epidural empyema | SSI was the most frequent complication with 19/139 affected patients (13.7%). Reported a statistically significant association between PEEK material and SSI. | No |
| Phang I. et al., 2019 | 10.1016/j.wneu.2019.06.091 | UK | Retrospective cohort | Adult | Neuro-oncology | To review authors’ experience in the treatment of brain metastases with minimally invasive approaches. |  | No | Not defined | There were 2 patients who suffered complications. One patient among 35 (2.9%) patients suffered a SSI at postoperative day 14 and required a wound re-exploration and removal of the bone flap. | No |
| Pie J.S. et al., 2019 | 10.1016/j.clineuro.2019.01.010 | China | Retrospective cohort | Adults and Paediatrics | Functional (Epilepsy) | To report authors’ experience of incorporating neuronavigation for treating patients with medically refractory epilepsy using 4 types of surgery. |  | No | Not defined | One patient had incision infection, surgical debridement was required. | No |
| Piitulainen J.M. et al., 2015 | 10.1016/j.wneu.2015.01.014 | Finland | Retrospective cohort | Adults and Paediatrics | General (Cranioplasty) | To analyse if there are pre-existing medical conditions associated with complications and compared the effect of different implant materials on the degree of complications. |  | Yes | Not defined | In patients with a cryopreserved autograft reimplanted, 40.0% of cases (8 of 20), the bone flap needed to be removed. Of these 8 patients with major complications, 5 presented with SSI, and 3 presented with resorption. In the autograft subgroup, the infection rate was 25.0%. | No |
| Pirotte B. et al., 2007 | 10.1007/s00381-007-0415-5 | Belgium | Prospective | Paediatric | Paediatric/ CSF dynamics | To evaluate whether the rigid application of a sterile protocol for shunt placement was applicable on a routine basis and allowed the reduction of shunt infections (SI) in children. |  | Yes | Not defined | No shunt infection found. | No |
| Potts, M.B. et al, 2015 | 10.3171/2014.12.JNS14938 | USA | Prospective cohort | Adult | Skullbase | To review authors’ experience in managing Spetzler-Martin Grade I and II AVMs, the most favourable AVMs for surgery and the ones most likely to have been selected for treatment outside of ARUBA’s randomisation process. |  | No | Not defined | 2 patients had wound infections requiring surgical debridement. | No |
| Prablek M. et al., 2021 | 10.1007/s00381-021-05170-3 | USA | Retrospective cohort | Paediatric | Paediatric/ Vascular | To examine the role of multimodality AVM treatment in paediatric AVM-associated epilepsy to characterise long-term epilepsy outcomes. |  | No | Not defined | 2 cases of wound infection. | No |
| Puthumana J.S. et al., 2023 | [10.1097/SCS.0000000000008872](https://doi.org/10.1097/scs.0000000000008872) | USA | Retrospective cohort | Paediatric | Skullbase | To investigate the optimal timing of open craniosynostosis repair as defined by perioperative outcomes in a large, multicenter cohort.  To examine the relationship between age at surgery and factors such as surgical stability, need for transfusion and perioperative complications. |  | Yes | Not defined | There was no difference in complications between age groups for deep SSI or organ site infection. | No |
| Radmanesh F. et al., 2009 | 10.3171/2009.2.PEDS08476 | Iran | Retrospective cohort | Paediatric | Paediatric/ CSF dynamics | To evaluate shunt complication rates in patients who underwent concurrent myelomeningocele (MMC) surgery and shunt placement and compare them to the rates in patients treated with shunt placement in a separate procedure. |  | Yes | 1. Positive CSF culture OR  2. Clinical evidence of infection with negative culture but positive CSF parameters (positive smear, low level of serum glucose (< 40 mg/dL), and high white blood cell count (> 10 cells/mm3) with polymorphonucleosis) | The overall rate of shunt infection was 16.5%. There was a high rate of shunt infection and mortality in those patients treated with CSF shunting only. There was no statistically significant difference between complication rates in patients in whom the 2 procedures were performed concurrently and those who underwent separate operations. | No |
| Rae A.I. et al., 2023 | 10.1227/neu.0000000000002563 | USA | Retrospective cohort | Adult | General (Cranioplasty) | To determine whether a wound healing protocol after cranioplasty reduced the rate of infections and to determine the value of this intervention. |  | Yes | Wound breakdown or possible presence of infection requiring oversew or prescription antibiotic, but not requiring surgical revision. | Concern for SSI at clinical follow-up requiring antibiotics or oversew but not surgery was almost 5 times more likely in the preprotocol than postprotocol group. | No |
| Rajkumar S. et al., 2024 | 10.1007/s00381-023-06076-y | USA | Retrospective cohort | Paediatric | Paediatric/ Skullbase | To analyse rates of independent and concurrent complications after craniosynostosis surgery in children with syndromic diagnoses. To assess factors associated with concurrent postoperative complications to help with risk stratification. |  | No | Not defined | 13.04% suffered deep surgical site infection. Blood transfusion was associated with a higher risk of deep incisional infection. | No |
| Ramos T. et al., 2016 | 10.1172/jci.insight.87919 | USA | Retrospective cohort | Paediatric | Paediatric/ CSF dynamics | To identify a biomarker that would identify shunt infection. |  | Yes | Not defined | Children with pyogenic shunt infection had significantly increased sMAC levels compared with noninfected patients. In infected patients undergoing serial CSF draws, sMAC levels were prognostic for both positive and negative clinical outcomes. | No |
| Rashidi A. et al., 2019 | 10.1016/j.clineuro.2019.105509 | Germany | Retrospective cohort | Adult | Trauma | To investigate one of the most frequent complications, bone flap infection, in order to identify prognostic factors of its development. |  | Yes | Laboratory signs of infection C-reactive protein (> 5 mg/l),  White blood cells (> 10.4 Gpt/l), Platelet count (> 400 Gpt/l). | Bone flap infection occurred in 24 patients (7.3%). | No |
| Rasouli J. et al., 2016 | 10.1016/j.wneu.2016.07.063 | USA | Retrospective cohort | Adult | Functional (DBS insertion) | To systematically examine the safe and effective use of vancomycin powder on DBS hardware. |  | Yes | Centre for Disease Control and Prevention Guidelines | 297 patients underwent DBS-related surgery. There were 4 instances of post-operative SSI (1.3%), all instances of SSI occurred exclusively in patients’ with Parkinson Disease. All patients who developed post-operative SSI were treated with hardware removal in the operating room and antibiotics. | No |
| Ratanalert S. et al., 2004 | 10.1016/j.jocn.2004.03.024 | Thailand | Prospective cohort | Adults and Paediatrics | CSF dynamics | To determine whether non-shaved patients undergoing ventriculoperitoneal shunt operations would experience a significantly increased shunt infection rate as compared to shaved patients. |  | Yes | Not defined | The shunt infection rate in the non-shaved and shaved groups was 6.25% and 14.94%, respectively. | No |
| Rauhala M. et al., 2020 | 10.1007/s00701-020-04398-3 | Finland | Retrospective cohort | Adult | Trauma | To examine the population-based incidence, complications, and total, direct hospital costs of chronic subdural hematoma (CSDH) treatment in a neurosurgical clinic during a 26-year period. |  | Yes | Not defined | Reported 29 patients were diagnosed with SSI. | No |
| Ravikumar V. et al., 2017 | 10.1093/neuros/nyw127 | USA | Retrospective cohort | Adult | General (Craniotomy) | To evaluate the efficacy of intrawound topical vancomycin for prevention of SSIs following open craniotomies. |  | Yes | SSI was defined as any craniotomy-related infection (superficial, deep, meningitis, etc.) following craniotomy that was documented in the medical record | :Our preintervention incidence of SSI was 2.2% and this was significantly reduced to 0% following introduction of topical vancomycin (P < .5). An ad hoc cost analysis suggested a cost savings of $59 965 with the use of topical vancomycin for craniotomies | No |
| Raviv N. et al., 2020 | 10.3171/2020.5.PEDS2019 | USA | Retrospective cohort | Paediatric | Paediatrics | To determine the incidence of acute fever in the postoperative paediatric neurosurgical population, as well as to assess the utility of performing further workup on these patients. |  | Yes | Not defined | 1% (2/143) of the patients with fevers were found to have an infection during the first 4 days (1 UTI and 1 CSF infection), and 8 patients (6 of whom had experienced acute postoperative fevers) developed a complication after the initial 4 days and within the first 30 days post-operatively (including 1 wound dehiscence requiring return to the operating room for closure with negative cultures, 1 CSF leak requiring repair and 3 CSF infections). | No |
| Reddy S. et al., 2014 | PMID: 25264642 | USA | Retrospective case-control | Adult | Trauma | To evaluate risk factors for intracranial haemorrhage in geriatric patients following ground-level falls, including preinjury use of specific anticoagulant or antiplatelet agents. | With Vs without use of anticoagulant medication | No | Not defined | 18 patients had complications including SSI. | No |
| Rehman A.U. et al., 2010 | 10.3171/2010.2.PEDS09151 | Pakistan | Prospective case-control | Paediatric | Trauma | To determine if the rate of postoperative shunt infections could be reduced simply by changing gloves before handling the shunt catheter. | Standard protocol VP shunt placement (Group A).  After initially double gloving, the outer pair of gloves was removed before handling the shunt catheter (Group B). | Yes | Positive CSF culture | By the 6-month interval, 10 infections had occurred (overall infection rate 9.80%). Of these, 8 infections occurred in the remaining 49 Group A patients (infection rate 16.33%) and 2 occurred in the remaining 53 Group B patients (infection rate 3.77%). | No |
| Renz N. et al., 2018 | 10.1016/j.wneu.2018.05.017 | Germany | Prospective cohort | Adult | General (Craniotomy) | To investigate the clinical, laboratory, and microbiological characteristics of intracranial infections after neurosurgery. |  | Yes | 1. Purulent wound discharge  2. Significant microbial growth in wound swabs or tissue samples  3.histopathological proof of infection  4. Local signs of infection  5. Depiction of an infection focus during surgery (abscess, empyema, osteomyelitis) or by magnetic resonance imaging or computed tomography  6. Infection diagnosed by the treating neurosurgeon and infectious diseases specialist. | Among 103 infections, 58 (56%) were extradural (including 31 skin and soft-tissue infections, 15 bone-flap infections, and 12 epidural empyema), 33 (32%) intradural (including 14 meningitis, 13 cerebral abscess, and 6 subdural empyema cases), and 12 (12%) were device-associated (involving 10 CSF-shunt and 2 deep brain stimulators). | No |
| Reponen E. et al., 2016 | 10.1016/j.wneu.2016.03.102 | Finland | Prospective cohort | Adult | General (Craniotomy) | To study how Modified Rankin Scale (mRS) changes associate with short-term postoperative outcome. |  | Yes | Not defined | One or more in-hospital minor complications occurred in 159 (38%) of the surgical patients, of whom 118 (74%) had no major complications at all. The three most frequent minor complications comprised subjective postoperative visual disturbance (18%), dysphasia/dysarthria (12%), and minor infections (9%). | No |
| Reponen E. et al., 2019 | 10.1093/neuros/nyy380 | Finland | Prospective observational | Adult | General (Craniotomy) | To evaluate whether nationwide quality of care programs in the United Kingdom and United States can measure differences in neurosurgical quality. |  | Yes | Not defined | 9 episodes of wound infection/meningitis (2.2%) out of 418 cases | No |
| Ribeiro B.B. et al., 2022 | 10.1097/j.pbj.0000000000000152 | Portugal | Retrospective cohort | Adult | General (Craniotomy) | To estimate the SSI-CRAN rate at the neurosurgery department of a tertiary hospital and to identify risk factors for its occurrence.  To identify the most common causative pathogens and compare outcomes of patients with and without SSI-CRAN. |  | Yes | European Centre for Disease Prevention and Control (ECDC) | From the 271 patients enrolled in this study, 15 (5.5%) developed SSI-CRAN within 30 days post-surgery, 11 (73.3%) of which were organ-space. The absence of normothermia and cerebrospinal fluid (CSF) leak were associated with SSI-CRAN. | No |
| Richards H.K. et al., 2009 | 10.3171/2009.4.PEDS09210 | UK | Retrospective cohort | Adults and paediatrics | Trauma | To assess the efficacy of antibiotic-impregnated catheters (AICs) against shunt infection by using a matched-pair study design. | Antibiotic impregnated shunt (AIS) catheters vs non-AIS catheters. | Yes | Not defined | In patients who received standard catheters, the infection risk was 4.7% (47 patients); in patients who received AICs, the infection risk was 3.0% (30 patients). | No |
| Rivero-Garvia M. et al., 2010 | 10.1007/s00701-010-0905-1 | Spain | Retrospective cohort | Paediatric | Paediatric | To propose a minimal handling protocol that reduces EVD-related infections. |  | Yes | Positive CSF culture collected based on the patient's symptoms and signs (fever, stiff neck, poor general condition). | Infection rate was 17% when non-antibiotic impregnated catheters were employed and 2.41% when antibiotic-impregnated catheters were inserted However, no statistically significant difference was observed in infection rate when the impact of a minimal handling protocol was considered: 4.29% when only the protocol was introduced and 2.41% when both the protocol and antibiotic-impregnated catheters were used. | Yes: “Cumulative incidence of EVD-related infection varies from 0% to 27% (mean cumulative incidence 9%). This  variability is partially dependent upon the definition of  EVD-related infection, a concept that has not been clearly  established and may vary from a positive culture in patients  with clinical criteria for infection” |
| Rizvi I. et al., 2023 | 10.1177/10556656221085478 | USA | Retrospective cohort | Paediatric | Paediatrics | To compare demographic, operative, and short-term outcomes data between open and minimally invasive surgical approaches for craniosynostosis repair utilising the American College of Surgeon’s National Surgical Quality Improvement Program Paediatric (NSQIP-P) database and highlight surgical disparities among races and ethnicities. |  | Yes | Not defined | Postoperative complications included superficial and deep incisional SSI, organ/ space SSI, and deep wound disruption/ dehiscence. | No |
| Roblot P. et al., 2023 | 10.1016/j.neuchi.2023.101458 | France | Retrospective cohort | Adult | General (Craniotomy) | To evaluate whether a bone flap with a positive bacteriological culture predicts bone flap removal for infection after cranioplasty. |  | Yes | Not defined | Reported 14 patients (7.2%) had an infection of the bone flap and underwent reoperation. | No |
| Rocque B.G. et al., 2018 | 10.3171/2018.3.PEDS17234 | USA | Retrospective cohort | Paediatric | Paediatric/ Trauma | To determine the risk factors for bone resorption and infection after paediatric cranioplasty. |  | Yes | Not defined | Reported an infection rate of 10.5%. | No |
| Rolston J.D. et al., 2016 | 10.1016/j.eplepsyres.2016.05.001 | USA | Retrospective cohort | Adult | Functional (Epilepsy surgical management) | To identify the frequency of epilepsy surgery procedures with greater confidence and to accurately estimate the frequency of surgical complications along with their patient-level predictors. |  | Yes | Not defined | Reported 5 cases of organ space SSI (1.1%) and 2 cases of deep SSI (0.4%), 1 case of superficial SSI (0.2%). | No |
| Rosa M. et al., 2017 | 10.1016/j.wneu.2016.09.069 | Italy | Retrospective cohort | Adult | Functional (DBS insertion) | To assess the incidence of infections retrospectively in a cohort of patients who underwent leads’ externalisation and the risk of infection related to local field potential (LFP) recording procedure. |  | Yes | Centre for Disease Control and Prevention Guidelines | The incidence of infections in patients who underwent leads’ externalisation was 2.8%. Moreover, the LFP recording procedure did not significantly increase the infection risk. | Yes: “It should be stressed that the data reported in the literature differ significantly between each other for several reasons. …Moreover, studies may differ for the time of observation, which varies from the first month after surgery to several years later, and for the definition of infection, which sometimes includes skin erosions or lesions without clear evidence of a microorganism growth.” |
| Roth J., et al., 2023 | 10.1111/epi.17796 | Multiple | Retrospective cohort | Paediatric | Paediatric/ Skullbase | To evaluate the safety of corpus callosotomy and its effectiveness for drop attacks and other seizure types following prior vagus nerve stimulation in children with Lennox-Gastaut syndrome. |  | No | Not defined | 4 wound infections reported. | No |
| Rothlind J. et al., 2021 | 10.1016/j.parkreldis.2021.12.011 | USA | Retrospective cohort | Adults | Functional (DBS insertion) | To examine the association between multi-domain cognitive decline (MCD) and demographic and baseline clinical variables and the incidence of serious adverse events (SAE) arising within a six-month interval following DBS for Parkinson’s disease. | . | No | Not defined | A variety of complications were documented in study after DBS surgery, including, infections and small intracranial haemorrhages. | No |
| Rubeli S.L. et al., 2019 | 10.3171/2019.5.FOCUS19272 | Switzerland | Retrospective cohort | Adult | General (Craniotomy) | To quantify SSI rates in cranial neurosurgery and quantify the impact of individual risk factors for SSI.  To evaluate whether standardised SSI surveillance combined with the implementation of an infection prevention bundle can reduce SSI. |  | No | Centre for Disease Control and Prevention Guidelines | Overall, 36 SSIs occurred with a median time to infection of 20 days. 29 cases of SSIs (80.6%) were detected within 3 months after surgery. The infection rate significantly decreased from 7.8% to 3.7% between the pre- and post-infection prevention bundle period implementation. | No |
| Rumalla K. et al., 2021 | 10.1016/j.wneu.2021.07.004 | USA | Retrospective cohort | Adult | Vascular | To assess the incidence of and risk factors for SSI after microsurgical clipping for acute subarachnoid haemorrhage (aSAH). |  | Yes | Not defined | Reported 3.9% (n = 23) of patients with infection, including 9 cases of infection associated with reoperation for cranioplasty and 14 cases in other patients. Infection associated with microsurgical clipping occurred in 14 patients (2.5%), 13 of which were mild superficial infections. | Yes - in discussion: "A meaningful comparison was limited by the heterogeneity of indications for surgery and the inconsistent definition of SSI (e.g., superficial infection vs. deep infection vs. both)" |
| Sæhle T. et al., 2015 | [10.3171/2014.12.JNS141029](https://doi.org/10.3171/2014.12.jns141029) | Norway | Retrospective cohort | Adults and Paediatrics | Trauma | To identify the complications and impact of intracranial pressure monitoring.  To determine the mean intracranial pressure (ICP) and characteristics of the cardiac-induced ICP waves in paediatric Vs adult over- and underdrainage. |  | No | Not defined | Reported 1 superficial wound infection, 4 shunt infections. | No |
| Sacko O. et al., 2007 | 10.1227/NEU.0b013e31820c02a3 | USA | Prospective case-control | Adult | Neuro-oncology | To assess the safety and effectiveness of an awake craniotomy (AC) with brain mapping in comparison with a craniotomy performed under general anaesthesia (GA). | Awake vs under general anaesthesia | Yes | Not defined | Reported 3 cases of wound infection in AC group and 4 cases in GA group. | No |
| Saenz A. et al., 2021 | 10.1007/s00381-021-05256-y | Argentina | Retrospective Case-control study | Paediatric | Paediatric/ Neuro-oncology | To study the risk factors associated with SSI following a resection of posterior fossa tumours in a purely paediatric population. |  | Yes | Centre for Disease Control and Prevention Guidelines | Patients with ventriculoperitoneal shunt or external ventricular drainage placement had a greater chance of presenting a postoperative surgical site infection. Prolonged operative time and CSF leak through the wound also caused an increase in the risk of SSI in the postoperative period. | No |
| Sakarunchai I. et al., 2016 | 10.1016/j.inat.2016.01.003 | Japan | Retrospective cohort | Adult | Vascular | To describe the outcomes of treatment and establish the most effective technique for frontal air sinus reconstruction. | . | Yes | Not defined | One patient developed a surgical wound infection. | No |
| Saleh C. et al., 2015 | 10.1007/s11920-015-0565-1 | France | Retrospective | Adults and Paediatrics | Functional (DBS insertion) | To analyse the reported complications and to allow a comprehensive assessment of the benefits and risks in DBS for psychiatric diseases. |  | Yes | Not defined | 7.7% of patients had infection as an adverse event. | No |
| Salle H. et al., 2021 | 10.1007/s15010-020-01534-0 | France | Retrospective cohort | Adult | Neuro-oncology | To evaluate the impact of SSI on the survival of glioblastoma patients. |  | Yes | Centre for Disease Control and Prevention Guidelines | Included data from 64 SSI cases and 58 non-infected glioblastoma patients. Infections occurred after surgery for primary tumours in 38 cases (group I) and after surgery for a recurrent tumour in 26 cases (group II). Patients in group I had significantly shorter survival compared to the other two groups. The one-year survival rate of patients who developed infections after surgery for primary tumours was 50%. | Yes |
| Salmanov A.G. et al., 2022 | PMID: 35092242 | Ukraine | Prospective cohort | Adult | General (Craniotomy) | To assess the incidence of SSI in patients undergoing neurosurgical procedures and antimicrobial resistance of responsible pathogens and determine their impact on inpatient mortality in Ukraine. |  | Yes | Centre for Disease Control and Prevention Guidelines | Of the 8741 neurosurgical patients, 1697 developed an SSI during the first 3 months with an overall incidence of SSI of 19.4%. Of the 1697 SSIs identified, 1191 (70.9%) were organ-space infections, 287 (16.9%) were superficial incisional infections, and 219 (12.9%) were deep incisional infections. | Yes |
| Sander C. et al., 2021 | 10.1016/j.wneu.2021.01.123 | Germany | Retrospective cohort | Adult | General (All neurosurgical cases) | To identify reasons for readmission in view of different diagnoses in cranial neurosurgery. |  | Yes | Not defined | Reported 25, 14, 4, 3, 3 cases of SSI for neoplasm, vascular, trauma, hydrocephalus, functional surgeries respectively. | No |
| Sander C. et al., 2020 | 10.1007/s00701-020-04521-4 | Germany | Prospective cohort | Adult | General (Craniotomy) | To identify predictive factors for unplanned early readmission in surgical and non-surgical groups in order to define patients with high risk.  To detect the causes of preventable readmissions as an approach to reduce readmission rates and to enable prevention strategies. | Surgical vs non-surgical groups | Yes | Not defined | Infections were the reason for readmission in 33.3% of patients. | No |
| Sangtongjaraskul S. et al., 2023 | 10.3171/2023.2.PEDS22535 | Thailand | Retrospective cohort | Paediatric | Paediatrics/ Neuro-oncology | To identify the risk factors for intraoperative blood transfusion in this procedure.  To investigate postoperative complications and clinical outcomes related to blood transfusion. | Transfusion Vs non-transfusion groups. | Yes | Not defined | The incidence rates of postoperative CNS infection (meningitis, infected ventriculoperitoneal shunt, and SSI) in the two groups was comparable. | No |
| Sangtongjaraskul S. et al., 2023 | 10.5005/jp-journals-10071-24418 | Thailand | Retrospective cohort | Paediatric | Neuro-oncology | To identify the factors associated with an ICU stay of more than one day after paediatric brain tumour surgery. Additionally, postoperative complications and patient outcomes are also reviewed. |  | Yes | Not defined | 16.93% of patients who were in ICU for 1-day experienced SSI. 33.61% of patients who had a prolonged ICU stay experienced SSI. | No |
| Sankey E.W. et al., 2015 | 10.3171/2015.4.JNS15129 | USA | Retrospective cohort | Adult | CSF dynamics | To analyse 103 adult patients with aqueductal stenosis who underwent endoscopic third ventriculostomy (ETV) for obstructive hydrocephalus and evaluated the effect of previous shunt placement on post-ETV outcomes. | . | Yes | Not defined | Nine (8.7%) pa- tients experienced transient, postoperative complications, including 1 lower-extremity DVT and 2 PEs requiring therapeutic anticoagulation, 1 wound infection resulting in septicemia and meningitis requiring 6 weeks of prolonged antibiotics, and 5 “other” complications. | No |
| Saramma P.P. et al., 2011 | 10.4103/0028-3886.76850 | India | Prospective case-control | Adult | General (Craniotomy) | To evaluate the effect of alcohol-based hand rub before and after each patient contact on SSI after elective neurosurgical procedures. | With Vs without alcohol rub | Yes | Centre for Disease Control and Prevention Guidelines | The SSI rate in the control group was 22 (2.3%) out of 974 compared to 14 (1.5%) out of 925 in the intervention group. | No |
| Sathaporntheera P. et al., 2020 | 10.1016/j.inat.2020.100865 | Thailand | Retrospective cohort | Adult | Skullbase | To examine instances of cerebrospinal fluid leakage after retrosigmoid surgery.  To examine complications associated with CSF leakage. |  | Yes | Not defined | All types of CSF leakage, i.e., pseudomeningocele, wound leakage, CSF rhinorrhea, and CSF otorrhea, were risk factors for wound infection and meningitis. | No |
| Savin I. et al., 2018 | 10.1016/j.jcrc.2018.01.022 | Russia | Retrospective cohort | Adults and Paediatrics | Trauma | To define the incidence of healthcare-associated ventriculitis and meningitis (HAVM) in the neuro-ICU and to identify HAVM risk factors using tree-based machine learning algorithms. |  | No | Not defined | Reported 20.2% rate of healthcare associated ventriculitis and meningitis in those with SSI. | No |
| Sayadi J.J. et al., 2023 | 10.1016/j.neurom.2022.02.227 | USA | Retrospective cohort | Adult | Functional (DBS insertion) | To determine whether the use of antibacterial envelopes reduced implantable pulse generator (IPG) related SSIs. | With Vs without antibacterial envelope | Yes | Not defined | Total of 7 SSI cases occurred. | No |
| Scheer M. et al., 2023 | 10.3390/jpm13071117 | Germany | Retrospective cohort | Adult | Neuro-oncology | To define risk factors for SSIs in patients with glioblastoma undergoing surgery. |  | Yes | Centre for Disease Control and Prevention Guidelines | A total of 177 patients were included, of which 14 patients (7.9%) suffered an SSI. These occurred after a median of 45 days. | No |
| Schellekes N. et al., 2021 | 10.3171/2020.9.JNS201980 | Israel and Italy | Retrospective cohort | Adult | Neuro-oncology | To investigate the impact of resection of a solitary lesion on survival of primary central nervous system lymphoma (PCNSL) patients. |  | No | Not defined | The resection group had a nonsignificant trend of being younger with a higher incidence of postoperative SSIs. | No |
| Schipmann S. et al., 2022 | 10.3171/2022.7.JNS22691 | Germany | Retrospective cohort | Adult | Neuro-oncology | To characterise those patients in whom postoperative monitoring is required by analysing early postoperative complications and associated risk factors. |  | No | Not defined | A higher rate of wound  infections (2.1%–5.6%) is a reason for reoperation. The number of SSIs was comparatively low at 0.5% after 30 days. | No |
| Schipmann S. et al., 2018 | 10.1007/s00701-018-03790-4 | Germany | Retrospective cohort | Adult | Neuro-oncology | To determine pre-operative factors associated with adverse events occurring within 30-days after neurosurgical tumour treatment. |  | Yes | Presence of clinical or radiological features with obtained cultures being positive. | 12.% nosocomial infections, of which 46.8% were SSI 22.3% (n = 21) were superficial (SSI grade I), 42.6% (n = 40) were deep (SSI grade II), and 35.1% (n = 33) affected the brain. | No |
| Schipmann S. et al., 2022 | 10.1007/s00701-021-05044-2 | Germany | Retrospective cohort | Adult | Neuro-oncology | To evaluate applicability of quality indicators in patients that underwent surgery for vestibular schwannoma and to identify potential new disease-specific quality indicators. |  | Yes | Not defined | The SSI rate was 1% (n = 1). No further infections manifested between days 31 and 90, consequently, there was no difference between the 30-day and 90-day nosocomial and surgical site infections rates. | No |
| Schmeiser B. et al., 2017 | 10.1093/neuros/nyx138 | Germany | Retrospective cohort | Adults and Paediatrics | Functional (temporal lobe resection for epilepsy) | To analyse epileptological and neuropsychological results as well as complications of different surgical strategies. | . | No | Not defined | Post-op infection occurred in 13 patients (3%). | No |
| Schneider M. et al., 2021 | 10.1007/s10143-020-01281-8 | Germany | Retrospective cohort | Adult | Neuro-oncology | To determine pre-operatively collectable patient-related risk factors that were associated with early postoperative complications following surgery for supratentorial located meningioma. |  | No | Not defined | 2% of patients experienced wound infection. | No |
| Schodel P. et al., 2020 | 10.1002/cam4.3402 | Germany | Retrospective cohort | Adult | Neuro-oncology | To investigate the course of patients undergoing surgical treatment for symptomatic brain metastases, focusing on the subsequent use of systemic therapy and its effects on survival. |  | No | Not defined | 38 cases of wound infection out of 750 patients. | No |
| Schutz A. et al., 2018 | 10.3171/2018.6.JNS172605 | Switzerland | Retrospective cohort | Adults and Paediatrics | General (Cranioplasty) | To investigate the risk factors for bone flap resorption (BFR) after autologous cranioplasty, including medical conditions and antihypertensive drug therapies, with a focus on angiotensin-converting enzyme inhibitors (ACEIs). |  | No | Not defined | A total of 47 patients (29.7%) were clinically or radiologically diagnosed with BFR. Seven patients (4.4%) developed a wound infection after cranioplasty, of which none developed BFR. | No |
| Schwarz F. et al., 2015 | 10.1016/j.clineuro.2015.08.002 | Germany | Retrospective cohort | Adult | Trauma | To identify risk factors for reoperation for surgical complications or recurrent hematomas. |  | Yes | Not defined | After initial burr hole trephination, 26 of 193 patients (13.5%) had another operation for surgical complications, including 3 cases of wound infection. | No |
| Seicean A. et al., 2020 | 10.1016/j.inat.2020.100692 | USA | Retrospective cohort | Adult | General (All neurosurgical cases) | To analyse and describe the outcomes of ASA 5 patients who underwent neurosurgery and identify risk factors for adverse outcome(s) in this population of “moribund” patients. |  | Yes | Not defined | Reported a rate of 0.6% for superficial SSI and 1.3% for deep wound infection. | No |
| Serrato P. et al., 2023 | [10.1097/SCS.0000000000009920](https://doi.org/10.1097/scs.0000000000009920) | USA | Retrospective cohort | Paediatric | Skullbase | To identify the factors associated with extended operative time (EOT) for paediatric patients with craniosynostosis undergoing cranial vault remodelling (CVR). |  | Yes | Not defined | The EOT cohort had significantly more cases of SSI (normal operative time (NOT): 0.9% vs. EOT: 1.4%), deep SSI (NOT: 0.3% vs. EOT: 0.7%), organ space SSI (NOT: 0.1% vs. EOT: 0.5%) | No |
| Servello D. et al., 2015 | 10.1007/s00701-023-05799-w | Italy | Retrospective cohort | Adults and Paediatrics | Functional (DBS insertion) | To analyse DBS complications (both procedure-related and hardware-related) and further assess potential predictive factors. |  | Yes | Centre for Disease Control and Prevention Guidelines | 9 cases of wound infection were reported. | No |
| Shafei M. et al., 2021 | 10.1016/j.jocn.2021.06.042 | Iran | Prospective case-control | Adults and Paediatrics | Trauma | To evaluate the risk of developing infection in subcutaneous pocketing (SP) vs cryopreservation (CP). | Subcutaneous pocketing Vs cryopreservation | Yes | Not defined | Four patients in the cryopreservation group (n = 50) indicated post-operative bone flap infection (8%) compared to no infection in the subcutaneous pocket method. | No |
| Shaftel K.A. et al., 2022 | 10.1227/neu.0000000000002119 | USA | Retrospective cohort | Adult | Trauma | To compare causes for hospital readmission within 30-days after surgical subdural haematoma (SDH) evacuation with burr hole craniostomy and evaluate readmission rates and independent predictors of readmission. |  | No | Not defined | The most common cause of readmission was recurrent SDH and the next most common was postoperative infection (1.8%). | No |
| Shallwani H. et al., 2018 | 10.1093/neuros/nyx211 | USA | Retrospective cohort | Not reported | General (All cranial neurosurgical cases) | To report SSI rates class I (clean) surgical procedures 13 months before and 13 months after surgical skull caps were banned at a single site with 25 operating rooms. |  | Yes | Not defined | An overall increase of 0.07% (0.77%-0.84%) in the cumulative rate of SSI in all class I operating room cases and of 0.03% (0.79%-0.82%) in the cumulative rate of SSI in all spinal procedures was noted. The cumulative rate of SSI in neurosurgery craniotomy/ craniectomy cases decreased from 0.95% to 0.75%. | No |
| Sharafat S. et al., 2023 | 10.12669/pjms.39.1.6408 | Pakistan | Retrospective cohort | Adults and Paediatrics | Trauma | To evaluate the management of posterior fossa extradural hematoma (PFEDH). |  | No | Not defined | 5 patients were observed having a post-operative wound infection. | No |
| Sheitoyan-Pesant C. et al., 2017 | 10.1016/j.ajic.2016.11.020 | Canada | Retrospective cohort | Adult | General (Craniotomy) | To investigate an outbreak of neurosurgical SSIs at a tertiary care hospital in Quebec, Canada, to identify the outbreak's cause. |  | Yes | Not defined | Of 80 patients who underwent craniotomy, 7 (8.75%) cases of infection occurred. This rate was significantly higher than that in the period between 2002 and 2014, wherein 51 infections occurred among 2,011 surgeries (2.5%). | No |
| Shekhar H. et al., 2016 | 10.3109/02688697.2015.1096903 | UK | Retrospective cohort | Adult | Trauma | To evaluate whether the introduction of antibiotic-impregnated EVDs in 2004 has decreased the ventriculostomy-related infection (VRI) rate. | Insertion of antibiotic-impregnated catheters Vs standard catheters | Yes | Fever associated with positive CSF culture.  Positive Gram stain of sample, CSF pleocytosis, documentation of ventriculitis as diagnosis in clinical notes. | Out of the 99 patients included in this study, 20 patients developed a VRI. Steroid use was associated to a higher infection rate (22 versus 11%). | No |
| Sherrod B. et al., 2017 | 10.1016/j.clineuro.2017.05.027 | USA | Retrospective cohort | Adult | Functional (temporal lobe resection for epilepsy) | To analyse short-term morbidity and mortality following temporal lobectomy using a validated national database. | . | Yes | Split into superficial incisional, deep incisional, or organ/space (includes osteomyelitis, ventriculitis, meningitis, and intracranial abscesses) | A total of 202 temporal lobectomy (TL) procedures were analysed. Reported a rate of 2% for SSI. | No |
| Shi Z. et al., 2017 | 10.1080/02688697.2016.1253827 | China | Retrospective cohort | Adult | Neuro-oncology | To determine the risk factors for and the incidence, outcomes, and causative pathogens of post-craniotomy intracranial infection (PCII) in patients with brain tumours. |  | Yes | Meningitis, brain abscess, subdural empyema, and/ or epidural abscess. Postoperative meningitis was defined as at least one of the following:  1. Organisms cultured from the CSF  2. One of the following signs or symptoms: fever of >38 C, headache, meningeal signs, and at least one of the following: (a) increased white cell count, decreased glucose level, and elevated protein in the CSF; (b) organisms on a Gram stain of the CSF; (c) positive antigen test of the CSF; (d) organisms cultured from the blood; or (e) diagnostic single-antibody titer (IgM) or four-fold increase in paired sera (IgG) for a pathogen. | The overall incidence of PCII was 6.8%, and 82.1% of all cases were diagnosed within two weeks after the craniotomy. Postoperative administration of antibiotics reduced the incidence of PCII. | Yes: "Two factors potentially contribute to the broad range of SSI incidences reported in the literature. First, some studies grouped superficial incisional  SSI, deep incisional SSI, and organ/space SSI together. However, great variation is noted in the incidence of and risk factors for different sites of infection. Second, the reported cases of craniotomy could include traumatic brain injury, stroke, vascular malformation, and brain tumors, which complicates comparisons among studies.” “Another reason for the differences in reported incidences was the  definition of infection. Therefore, to investigate post-craniotomy infections, standard criteria for population, and diagnosis should be  considered." |
| Shibahashi K. et al., 2017 | [10.1016/j.wneu.2017.01.106](https://doi.org/10.1016/j.wneu.2017.01.106) | Japan | Retrospective cohort | Adult | General (Cranioplasty) | We reviewed >10 years of institutional experience to identify risk factors of surgical site infection (SSI) after cranioplasty. |  | Yes | 1. Records indicating subcutaneous abscess  2. Surgical records indicating bone flap removal; and  3. Surgical records of wound revision. | There were 13 cases of SSI (8.4%) and 2 cases of postoperative wound dehiscence (1.3%). There was a significant relationship between operative time and SSI. | No |
| Shibamura-Fujiogi M. et al., 2021 | [10.1186/s12871-021-01342-5](https://doi.org/10.1186/s12871-021-01342-5) | USA | Retrospective cohort | Paediatric | Paediatric/ CSF dynamics | To evaluate risk factors associated with SSIs following CSF diversion surgeries following a SSI bundle at a single quaternary care paediatric hospital. |  | Yes | Centre for Disease Control and Prevention Guidelines | A total of 558 CSF diversion procedures with an overall SSI rate of 3.4% were recorded. The SSI rates for shunt, external ventricular drain (EVD) placement, and endoscopic third ventriculostomy (ETV) were 4.3, 6.9 and 0%, respectively. | No |
| Shiferaw A.A. et al., 2024 | [10.1016/j.wneu.2023.10.077](https://doi.org/10.1016/j.wneu.2023.10.077) | Ethiopia | Retrospective cohort | Adult | Neuro-oncology | To describe patients, perioperative care, and outcomes undergoing supratentorial and infratentorial craniotomy for brain tumour resection in a tertiary-care hospital in Ethiopia. | . | Yes | Not defined | Approximately one in ten patients experienced postoperative obstructive hydrocephalus, surgical site infections, or pneumonia. SSI occurred in 14 cases (9%). | No |
| Shimizu K. et al., 2015 | [10.1055/s-0034-1396660](https://doi.org/10.1055/s-0034-1396660) | Japan | Prospective | Adult | Vascular | To describe the details and advantages of the authors’ surgical procedure with practical operative findings and excellent results. |  | No | Not defined | Two patients (2%) had a wound infection, one of which needed the removal of the titanium plate. | No |
| Shin Y.S. et al., 2024 | [10.23736/S0390-5616.19.04693-9](https://doi.org/10.23736/s0390-5616.19.04693-9) | South Korea | Retrospective cohort | Adult | General (Craniotomy) | To clarify the relationships between tobacco smoking and postoperative complications following craniotomy. |  | Yes | Not defined | The incidence of major complications and overall complications were also significantly higher among smokers than never smokers. | No |
| Shinoura N. et al., 2004 | [10.1080/02688690400022771](https://doi.org/10.1080/02688690400022771) | Japan | Retrospective cohort | Both | Neuro-oncology | To analyse the risk factors and blood count data after craniotomies for brain tumours and found that we could predict infection within four days after craniotomies according to those analyses. |  | Yes | Purulent discharge from the incision, bacteria isolated  from serous drainage, or a clinical diagnosis of infection by the attending neurosurgeon. | Reported 3 people had a scalp infection out of all patients. | No |
| Sicking J. et al., 2018 | [10.1007/s00701-018-3617-6](https://doi.org/10.1007/s00701-018-3617-6) | Germany | Retrospective cohort | Adult | Neuro-oncology | To analyse the development of surgical management of primarily diagnosed intracranial meningioma, perioperative morbidity, and patients’ prognosis in a German neurosurgical department over a 25-year period. |  | Yes | Not defined | The risk of both postoperative surgical site infection and CSF leakage increased during the time of study. The frequency of surgical reintervention for postoperative surgical site infection matched observations in previous series. | No |
| Singh A.K. et al., 2014 | [10.4103/0028-3886.132364](https://doi.org/10.4103/0028-3886.132364) | India | Prospective case-control | Adult | Trauma | To evaluate the effect of subdural drains on recurrence rate of haematoma. | Drain Vs without drain group | Yes | Not defined | SSI between the 2 groups was not significant. | No |
| Skyman S. et al., 2020 | [10.1007/s00701-020-04309-6](https://doi.org/10.1007/s00701-020-04309-6) | Sweden | Quasi-experimental design | Adult | Neuro-oncology | To evaluate the effect of a change in antibiotic prophylaxis regimen from Cloxacillin to Cefuroxime in patients with intracranial tumours, treated surgically with a craniotomy. | Cloxacillin Vs Cefuroxime as antibiotic prophylaxis | Yes | Centre for Disease Control and Prevention Guidelines | The pre-intervention group had a significant higher incidence of SSI, 13.3% vs 5.4% in the intervention group. The number of reoperations due to SSI were significantly reduced in the intervention group, 3.4% vs 8.3%, as was the total antibiotic use and the number of visits in the outpatient clinic. | No |
| Slattery W.H. III et al., 2001 | [10.1097/00129492-200111000-00031](https://doi.org/10.1097/00129492-200111000-00031) | USA | Retrospective cohort | Adults and Paediatrics | Neuro-oncology | To review complications that occur during the course of acoustic neuroma surgery. |  | No | Not defined | Three patients received intravenous antibiotics to treat infection. One middle fossa infection occurred within 3 days of the surgery, and the other two occurred 3 and 4 weeks after surgery. | No |
| Slot E.M.H. et al., 2023 | [10.3171/2022.11.PEDS22421](https://doi.org/10.3171/2022.11.peds22421) | The Netherlands | Retrospective cohort | Paediatric | General (Craniotomy) | To establish the incidence of CSF leakage after intradural cranial surgery in the paediatric population. |  | Yes | Not defined | 11 patients experienced wound infections. | No |
| Smith K. et al., 2022 | 10.1097/01.ccm.0000809444.94667.19 | USA | Retrospective case-control | Adult | Trauma | To compare the incidence of SSIs in patients receiving extended versus brief antibiotic prophylaxis following neurosurgical drain placement (NDP). | Extended vs brief antibiotic prophylaxis | Yes | Not defined | The extended group had more SSIs compared to the brief group; however, this did not reach statistical significance. | No |
| Sneh-Arbib O. et al., 2013 | [10.1007/s10096-013-1904-y](https://doi.org/10.1007/s10096-013-1904-y) | Israel | Prospective cohort | Adult | Craniotomy | To examine factors associated with post-neurosurgical SSIs, focusing on post-operative factors. |  | Yes | Centre for Disease Control and Prevention Guidelines | A total of 502 patients were included, with an overall SSI rate of 5.6 % (28 patients), of which 3.2 % were intracerebral. Non-elective surgery, external CSF drainage/ monitoring devices, re-operation, and post-operative respiratory failure were independently associated with subsequent SSI. | Yes: "The reported  infection rate after intracranial neurosurgery in large cohorts published in the period 1985–2007 ranged between 0 and  6.2%. This wide range is probably partially real and partially  due to different definitions and SSI categories included in different studies." |
| So R. et al., 2022 | [10.1227/ons.0000000000000546](https://doi.org/10.1227/ons.0000000000000546) | USA | Retrospective cohort | Adult | Vascular | To compare postoperative pain and numbness outcomes after microvascular decompression in patients with trigeminal neuralgia of exclusive venous compression. |  | No | Not defined | Reported 3 cases of wound infection in the transposition group and 3 cases in the coagulation group. | No |
| Soleman J. et al., 2021 | [10.1016/j.wneu.2020.10.138](https://doi.org/10.1016/j.wneu.2020.10.138) | Switzerland | Retrospective cohort | Adult | General (Craniotomy) | To assess the 30-day mortality and morbidity rate in elderly patients undergoing cranial surgery and the clinical outcome measured by the modified Rankin Scale score. |  | Yes | Not defined | Postoperative infection occurred in 14 patients (2.1%). | No |
| Soto J. et al., 2023 | [10.1016/j.wneu.2023.04.020](https://doi.org/10.1016/j.wneu.2023.04.020) | USA | Retrospective cohort | Adult | Neuro-oncology | To characterise factors associated with complications for supratentorial craniotomy for primary malignant brain tumour resection. | . | Yes | Not defined | Reported a rate of 0.6% for superficial SSI, 0.4% for deep SSI and 1.3% for organ/ space SSI. | No |
| Sousa S. et al., 2023 | [10.3171/2023.2.JNS222262](https://doi.org/10.3171/2023.2.jns222262) | Portugal | Prospective case-control | Adult | Trauma | To investigate the best strategy to reduce postoperative complications and to improve functional outcomes. | Early mobilisation vs 48-hour bed rest | No | Not defined | Reported 1 infection in bed rest group compared to 2 in early mobilisation group. | No |
| Spille D.C. et al., 2022 | [10.1055/a-1911-8678](https://doi.org/10.1055/a-1911-8678) | Germany | Retrospective cohort | Adults and Paediatrics | Neuro-oncology | To analyse currently applied quality indicators in meningioma surgery and the identification of potential new measures. |  | Yes | Not defined | 1 incident of SSI reported. | No |
| Sponton L. et al., 2022 | [10.1055/s-0042-1751000](https://doi.org/10.1055/s-0042-1751000) | Germany | Retrospective cohort | Adult | Skull base/ Neuro-oncology | To present the largest retrospective single-institution and long-term follow-up study of endoscopic-assisted supraorbital approach (eSOA) for anterior skull base meningiomas (ASBM) resection, providing further insight regarding indication, surgical considerations, complications, and outcome. |  | No | Not defined | 1.1% rate of wound infection and 1.7% rate of meningitis reported. | No |
| Spuck S et al. 2010 | [10.1227/NEU.0b013e3181f88867](https://doi.org/10.1227/neu.0b013e3181f88867) | Germany | Retrospective cohort | Adults and Paediatrics | Functional (VNS insertion for epilepsy) | To analyse surgical and technical complications after implantation of left-sided VNS in patients with therapy-refractory epilepsy and depression. |  | No | Not defined | Complications induced by the operations were deep wound infection or disturbed wound healing in 3.8% and in 2.8% directly after implantation. 4 episodes of infection reported. | No |
| Stoker M.A. et al., 2012 | [10.1055/s-0032-1312709](https://doi.org/10.1055/s-0032-1312709) | USA | Retrospective case-control | Adult | Vascular | To retrospectively assess whether variations in operative protocol influenced the incidence of CSF leakage following microvascular decompression. | Complete vs incomplete reconstruction | No | Not defined | 3% infection rate in complete reconstruction group. Total 6% infection rate among all patients. | No |
| Strahm C. et al., 2018 | [10.1016/j.wneu.2017.12.062](https://doi.org/10.1016/j.wneu.2017.12.062) | Switzerland | Prospective cohort | Adult | General (Craniotomy) | To determine infection rate (IR) and to identify modifiable risk factors (RF) in cranial neurosurgery in a neurosurgical department for tertiary referral as part of an infection control surveillance to reduce surgical site infections (SSI). |  | Yes | Centre for Disease Control and Prevention Guidelines | Overall, 24 infections in 333 index procedures were recorded, resulting in an IR overall of 7.2%. Most infections were deep seated and only 1 was superficial. | No |
| Sughrue M.E. et al., 2011 | [10.1016/j.jocn.2011.01.016](https://doi.org/10.1016/j.jocn.2011.01.016) | USA | Prospective cohort | Adult | General (decompressive hemicraniectomy) | To present authors’ experience with an improved technique for wound closure after unilateral decompressive hemicraniectomy with a wide cruciate durotomy. |  | Yes | Purulent drainage from the cranial incision or extra-axial empyema found on re-operation. | Patients closed using authors’ new technique experienced markedly reduced rates of wound infection (p < 0.01). | No |
| Tabata S. et al., 2022 | [10.1016/j.jocn.2022.02.020](https://doi.org/10.1016/j.jocn.2022.02.020) | Japan | Retrospective cohort | Adult | General (Cranioplasty) | To retrospectively analyse the risk factors related to graft infection after cranioplasty. | . | Yes | Not defined | Graft infection after cranioplasty was identified in 16 patients (8.3%). In 12 of the 16 infected cases, diffusion-weighted MRI revealed subcutaneous and/ or intracranial abscesses. Smoking and presence of an allergy were identified to be risk factors for post-cranioplasty graft infection. | No |
| Tacconelli E. et al., 2008 | [10.1016/j.jhin.2008.04.032](https://doi.org/10.1016/j.jhin.2008.04.032) | Italy | Randomised clinical trial | Adult | CSF dynamics | To compare the efficacy of vancomycin and cefazolin in preventing bacterial infections following CSF insertion and to record any adverse effects. | . | Yes | At least one of the following:  1. Organism cultured from CSF  2. Organism cultured from the blood  3. Treatment with appropriate antibiotic therapy and at least one of the following: (a) fever (>38 degrees) and clinical signs of central nervous system infections, (b) decreased glucose in CSF. | Shunt infections were significantly less likely to be observed in patients who were on vancomycin prophylaxis (4% vs 14%). Four patients developed shunt infections in the vancomycin group compared with 12 patients in the cefazolin group. | No |
| Tacconi L. et al., 2019 | [10.1016/j.wneu.2018.10.023](https://doi.org/10.1016/j.wneu.2018.10.023) | Italy | Retrospective cohort | Adult | General (All cranial neurosurgical cases) | To describe the results on the use of skin glue (Dermabond C) for the closure of cranial wounds in terms of cosmetic results, patient satisfaction, and complications. |  | No | Not defined | No reported cases of wound complication or infection. | No |
| Tafreshi A.R. et al., 2021 | [10.1016/j.clineuro.2020.106372](https://doi.org/10.1016/j.clineuro.2020.106372) | USA | Retrospective cohort | Adult | Functional (DBS insertion) | To assess differences in postsurgical complication rates in patients undergoing the most common types of neurostimulation surgery. |  | Yes | Not defined | Similar readmission rates due to infection were seen in Parkinson’s disease (17 cases, 8.2 %), essential tremor (13 cases, 6.5 %), seizure (17 cases, 8.2 %) and dystonia (18 cases, 8.6 %) groups. | No |
| Takami H. et al., 2021 | [10.1016/j.wneu.2021.11.010](https://doi.org/10.1016/j.wneu.2021.11.010) | Canada | Retrospective cohort | Adult | General (Craniotomy) | To investigate the incidence and the risk factors for post-discharge readmission after awake surgeries. | . | No | Not defined | 1.4% rate of surgical site infection reported. Postoperative CT scans are of importance to predict the cases at risk for infection. | No |
| Takeuchi S. et al., 2015 | [10.1016/j.wneu.2015.01.030](https://doi.org/10.1016/j.wneu.2015.01.030) | Japan | Prospective cohort | Adult | General (Craniotomy) | To describe a procedure for maintaining the patency of the nasofrontal duct and direct suture of the exposed and violated frontal sinus mucosa. |  | Yes | Not defined | The frequencies of mucocele development and infection after obliteration with fat only are approximately 10% and 3%, respectively. | No |
| Tanaka K. et al., 2019 | [10.1016/j.anl.2022.03.006](https://doi.org/10.1016/j.anl.2022.03.006) | Japan | Retrospective cohort | Adults and Paediatrics | Skullbase | To clarify the early postoperative survival-related complications after skull base reconstruction which should be treated actively by plastic surgeons. |  | No | Not defined | Reported wound infection in 7 patients. | No |
| Tandon N. et al., 2019 | [10.1001/jamaneurol.2019.0098](https://doi.org/10.1001/jamaneurol.2019.0098) | USA | Retrospective case-control | Adults and Paediatrics | Trauma | To perform a comparative analysis of the relative efficacy, procedural morbidity, and epilepsy outcomes consequent to stereoelectroencephalography and subdural grids in similar patient populations. | Subdural Grids vs Stereoelectroencephalography | No | Not defined | 3 subdural grid related infections recorded. | No |
| Tang K. et al., 2001 | [10.1159/000050379](https://doi.org/10.1159/000050379) | UK | Prospective non-randomised | Paediatric | Paediatric | To investigate whether or not shaving hair in neurosurgical operations carries an increased infection rate. |  | Yes | Not defined | The only complications observed were 4 incidences of wound dehiscence (2 in the hair shave and 2 in the no hair shave group) and 3 shunt infections (2 in the hair shave and 1 in the no hair shave group). Age was a significant factor in shunt infection, as all shunt infections were seen in patients aged less than 6 months, regardless of whether the hair was shaved or not. | No |
| Taylor B.E.S et al., 2016 | [10.1227/NEU.0000000000001110](https://doi.org/10.1227/neu.0000000000001110) | USA | Retrospective cohort | Adults and Paediatrics | General (All neurosurgical cases) | To determine the incidence, timing, and causes of 30-day readmission after neurosurgical procedures. |  | Yes | Not defined | Seizures, wound complications (without infection), and CSF leaks each accounted for <5% of readmissions. Infection and medical complications are the main drivers of readmission. | No |
| Teshita G. et al., 2024 | [10.1016/j.wnsx.2023.100264](https://doi.org/10.1016/j.wnsx.2023.100264) | Ethiopia | Retrospective cohort | Adult | Trauma | To determine the clinical characteristics and functional outcomes and the associated factors of surgically treated head trauma patients with acute subdural haematoma. |  | Yes | Not defined | Reported 59 patients experienced complications, with most of them being SSIs. | No |
| Test M.R. et al., 2019 | [10.3171/2019.2.PEDS18638](https://doi.org/10.3171/2019.2.peds18638) | USA | Retrospective cohort | Paediatric | Paediatric/ CSF dynamics | To evaluate the relationship between the causative organism of CSF shunt infections and the timing of infection.  To evaluate the relationship between the causative organism and clinical features of the infection. |  | Yes | Infectious Diseases Society of America’s 2017 Clinical Practice Guidelines for Healthcare- Associated Ventriculitis and Meningitis:  fever, lethargy, headache, nausea, vomiting, abdominal pain, surgical site changes, neurological changes, and leukocytosis. Surgical site change was defined as documented redness or swelling at the surgical site, leakage of CSF, or visible hardware. | Children with infection caused by Staphylococcus aureus were more likely to present with surgical site changes and leukocytosis at the time of diagnosis. | No |
| Tew J. et al., 2017 | [10.1093/ons/opw004](https://doi.org/10.1093/ons/opw004) | USA | Prospective, randomised trial | Adult | Other (dural closure) | To evaluate the safety and efficacy of a novel hydrogel, Adherus Dural Sealant, when compared with control, DuraSeal Dural Sealant System, as an adjunct to standard methods of dural repair. | Adherus Dural Sealant Vs control (DuraSeal Dural Sealant) | Yes | Not defined | Of 117 control cases, 2 patients had unplanned reoperations for surgical site infections. Five (4.0%) control patients had serious device-related adverse effects including incision-site infection (2 cases). | No |
| Thenier-Villa J.L. et al., 2018 | [10.3171/2018.2.PEDS17717](https://doi.org/10.3171/2018.2.peds17717) | Spain | Retrospective cohort | Paediatric | Skullbase | To describe and analyse the radiological and clinical evolution of bone defects after craniosynostosis. |  | Yes | Not defined | 6.9% of patients experienced SSI as a complication. | No |
| Thompson D.N.P. et al., 2007 | [10.3171/ped.2007.106.1.15](https://doi.org/10.3171/ped.2007.106.1.15) | UK | Prospective | Paediatric | Paediatric | To establish whether microbiological contamination at the time of shunt insertion can be detected and used to predict the likelihood of subsequent shunt infection. |  | Yes | Organism was confirmed by CSF culture. | Reported a total of 108 total shunt insertions yielding 325 swab samples. Organisms were identified in cultures of 50 swab samples (15%) obtained in 40 patients (37%). In seven of these 40 patients (17.5%) a CSF infection subsequently developed. | No |
| Thu L.T.A. et al., 2007 | [10.1086/516661](https://doi.org/10.1086/516661) | Vietnam | Quasi-experimental design | Adults and Paediatrics | General (All neurosurgical cases) | To assess the impact of the use of an alcohol-chlorhexidine–based hand sanitizer on SSI rates among neurosurgical patients in Ho Chi Minh City, Vietnam. |  | Yes | Centre for Disease Control and Prevention Guidelines | Introduction of a hand sanitiser can both reduce SSI rates in neurosurgical patients, with particular impact on superficial SSIs, and reduce the overall postoperative length of stay and the duration of antimicrobial use. | No |
| Toescu S.M. et al., 2021 | [10.3171/2020.6.PEDS2089](https://doi.org/10.3171/2020.6.peds2089) | UK | Retrospective cohort | Paediatric | Neuro-oncology | To critically assess early, surgery-related morbidity in paediatric fourth ventricle tumours, with particular reference to surgical approach. |  | No | Not defined | There were 7 surgical site infections reported. | No |
| Tokimura H. et al., 2009 | [10.1016/j.jcms.2009.06.003](https://doi.org/10.1016/j.jcms.2009.06.003) | Japan | Prospective cohort | Adult | General (Craniotomy) | To assess the efficacy and safety of the authors’ scalp management and analyse the risk factors for postoperative wound infection. |  | Yes | Guideline for Prevention of Surgical Infection | 1.1% rate of infection reported. | No |
| Tolleson C. et al., 2014 | [10.1159/000362934](https://doi.org/10.1159/000362934) | USA | Retrospective cohort | Adult | Functional (DBS insertion) | To better define risk factors for infection post-operatively for DBS insertion. |  | Yes | National Surgical Quality Improvement Program (NSQIP) definition of SSI: occur within 30 days after surgery and include at least one of the following: 1. Purulent drainage, with or without laboratory confirmation, surrounding the generator, extension or generator site; 2. Organisms isolated from an aseptically obtained culture of fluid or tissue from the site of interest, or 3. one of the following signs or symptoms of infection: pain or tenderness, localised swelling, redness, or heat and site is deliberately opened by the surgeon. | The total infection rate per patient during the analysis period was 5.82% (26 out of 447 cases), but only 9 patients met the formal NSQIP definition of DBS surgical site infection due to occurrence of infection within 30 days postoperatively, giving a rate of 2.01%. | Yes: "it is difficult to compare percentages directly because different authors use a variety of criteria to define postsurgical DBS infection” |
| Tomatis A. et al., 2019 | [10.1016/j.wneu.2019.06.150](https://doi.org/10.1016/j.wneu.2019.06.150) | Italy | Retrospective cohort | Adult | Vascular | To review the clinical and radiological outcomes of clipped ruptured and unruptured aneurysms. To analyse the relationship between increasing surgical experience and the operative time, recovery time, and clinical outcomes. |  | No | Not defined | 1 case wound infection reported. | No |
| Torres S. et al., 2018 | [10.1016/j.bjid.2018.08.001](https://doi.org/10.1016/j.bjid.2018.08.001) | Brazil | Retrospective cohort | Adult | General (Craniotomy) | To determine the incidence of SSI in patients undergoing craniotomy and to compare 12-month and 3-month post-discharge surveillance periods in terms of their impact on the incidence of SSI in those patients. |  | Yes | Centre for Disease Control and Prevention Guidelines | Of the 173 patients undergoing craniotomy during the study period, 20 developed an SSI during the first 12 months after discharge with an overall incidence of SSI of 11.56%. Of the 20 SSIs identified, 13 (65%) were organ-space infections, 4 (20%) were superficial incisional infections, and 3 (15%) were deep incisional infections. | No |
| Trinh V. et al., 2015 | [10.3171/2014.9.JNS131648](https://doi.org/10.3171/2014.9.jns131648) | USA | Retrospective cohort | Adult | Neuro-oncology | To examine how procedural volume and patient demographics impact complication rates and value of care in those who underwent biopsy or craniotomy for supratentorial primary brain tumours. |  | Yes | Not defined | 0.8% rate of wound infections reported. | No |
| Trungu S. et al., 2022 | [10.3390/jcm11051286](https://doi.org/10.3390/jcm11051286) | Italy | Prospective cohort | Adult | Trauma | To evaluate the clinical outcomes of shunting in normal pressure hydrocephalus following a new standardised protocol. |  | Yes | Not defined | 2 patients had a wound infection reported. | No |
| Tsang A.C.O. et al., 2015 | [10.1016/j.jocn.2014.11.021](https://doi.org/10.1016/j.jocn.2014.11.021) | Hong Kong | Retrospective cohort | Adults and Paediatrics | Trauma | To review all post-cranioplasties performed in a single institution with a view to identify complications and their predisposing factors. |  | Yes | Not defined | 9.3% infection rate reported. | No |
| Tunthanathip T. et al., 2019 | [10.3171/2019.5.FOCUS19241](https://doi.org/10.3171/2019.5.focus19241) | Thailand | Retrospective cohort | Adults and Paediatrics | General (All cranial neurosurgical cases) | To compare the performance of various machine learning models in terms of predicting surgical infection after neurosurgical operations. |  | Yes | Centre for Disease Control and Prevention Guidelines/ National Healthcare Safety Network Surveillance Definitions. | Data were available for 1471 patients in the study period. The SSI rate was 4.6%, and the type of SSI was superficial, deep, and organ/space in 1.2%, 0.8%, and 2.6% of cases, respectively. | No |
| Tzikos G. et al., 2022 | [10.3390/nu14132620](https://doi.org/10.3390/nu14132620) | Greece | Retrospective cohort | Adult | Trauma | To assess the efficacy of a four-probiotic regime to reduce the incidence of SSI in multi-trauma patients, with a brain injury included. |  | Yes | Centre for Disease Control and Prevention Guidelines | A total of 23 (46.0%) and 13 (24.5%) infectious insults in 89 (50 placebo patients) and 88 (53 probiotics-treated) operations were recorded, the majority of them relating to osteosynthesis. The prophylactic administration of probiotics in multi-trauma patients exerts a positive effect on the incidence of SSI. | No |
| Uche E.O. et al., 2013 | [10.1159/000357384](https://doi.org/10.1159/000357384) | Nigeria | Retrospective cohort | Paediatric | Paediatric/ CSF dynamics | To evaluate the determinants of shunt infection (SI), the microbial and treatment patterns, and the outcome of treatment. |  | Yes | Positive CSF and shunt component culture or other supportive CSF or haematological findings in culture or Gram stain-negative cases associated with clinical features of infection. | SI remains a life-threatening and costly complication  of VP shunt placement. About 36.4% of the positive cultures yielded Staphylococcus aureus. | No |
| Uzuka T. et al., 2017 | [10.2176/nmc.oa.2017-0034](https://doi.org/10.2176/nmc.oa.2017-0034) | Japan | Prospective and Retrospective (2 studies) | Adult | Neuro-oncology | To investigate the incidence of SSI following malignant brain tumour surgery.  To assess the use of a care bundle technique in reducing incidence of SSI. |  | Yes | Centre for Disease Control and Prevention Guidelines | The SSI incidence in the retrospective (n = 161) and prospective studies (n = 68) were 4.3% and 4.4%, respectively, similar to the previously reports on general craniotomies. | Yes: "the criteria for SSIs were different in the retrospective and the prospective study. Therefore, a direct  comparison of the SSI rate and the risk factors obtained from the two studies was not straightforward.” |
| Van der Veken J. et al., 2014 | [10.1007/s00701-014-2042-8](https://doi.org/10.1007/s00701-014-2042-8) | Belgium | Retrospective cohort | Adult | Trauma | To investigate the outcome of mini-craniotomy as the sole treatment in patients with chronic subdural haematoma. |  | No | Not defined | The surgical complications included two wound infections. | No |
| van der Vlis T. et al., 2022 | [10.1016/j.neurom.2021.12.011](https://doi.org/10.1016/j.neurom.2021.12.011) | Netherlands | Retrospective cohort | Adult | Functional (DBS insertion) | To give an overview of the nature and frequency of adverse event and to describe the way they were managed. To identify possible risk factors to inform possible future preventive measures. |  | Yes | Not defined | SSI was the most commonly reported surgery-related adverse event. 20 infections occurred after primary implantation of DBS hardware (9.95%). No difference in SSI incidence was observed before and after April 2014, when complete hair removal was abandoned . | No |
| van Schooten J. et al., 2023 | [10.1016/j.bas.2023.102733](https://doi.org/10.1016/j.bas.2023.102733) | Netherlands | Retrospective cohort | Adults and Paediatrics | Functional (VNS insertion for Epilepsy) | To assess surgical complication rates of all types of VNS surgeries performed between 2008 and 2022 and to investigate potential risk factors for getting a surgical complication. |  | Yes | Not defined | Primary implantation was performed in 306 patients. In total, 41 complications occurred, consisting of 11 wound infections. | No |
| Vankipuram S. et al., 2019 | [10.1016/j.wneu.2019.12.004](https://doi.org/10.1016/j.wneu.2019.12.004) | India | Randomised controlled study | Adult | Trauma | To compare the outcomes of both the four-quadrant osteoplastic decompressive craniotomy (FoQOsD) versus conventional decompressive craniectomy (DC) for traumatic brain injury. | Four-quadrant osteoplastic decompressive craniotomy versus conventional decompressive craniectomy | No | Not defined | Cranial surgical site infection was seen in three patients (5%) in the DC group and five patients in the FoQOsD group (8.9%). | No |
| Veldeman M. et al., 2020 | [10.3171/2020.2.JNS193335](https://doi.org/10.3171/2020.2.jns193335) | Germany | Retrospective cohort | Adult | Trauma | To identify modifiable risk factors to prevent cranioplasty failure. |  | Yes | Open or closed graft infection requiring surgical wound revision with or without the removal of the implanted material. | Total of 25 SSI cases reported. | No |
| Venable G.T. et al., 2020 | [10.1016/j.wneu.2019.11.018](https://doi.org/10.1016/j.wneu.2019.11.018) | USA | Retrospective cohort | Adult | Trauma | To evaluate the preventable shunt revision rate in adult hydrocephalus cases. |  | Yes | Not defined | Infection was the leading cause of preventable failure (10 patients). | No |
| Verberk J.D. M et al., 2016 | [10.1016/j.jhin.2015.12.018](https://doi.org/10.1016/j.jhin.2015.12.018) | Netherlands | Retrospective cohort (post-hoc analysis of previous investigation) | Adults | CSF dynamics | To compare the occurrence and bacterial aetiology of ventriculostomy-related infections (VRIs) in neurosurgical patients treated with antibiotic-impregnated drains vs plain EVDs. | Antibiotic-impregnated vs plain EVDs. | Yes | Centre for Disease Control and Prevention Guidelines | This study could not  demonstrate a significant benefit of antibiotic-impregnated drains over plain EVDs in terms of the incidence of infection, either using the VRI definition or when considering all positive CSF cultures. | No |
| Vergani F. et al., 2010 | [10.1016/j.wneu.2010.01.017](https://doi.org/10.1016/j.wneu.2010.01.017) | Italy | Retrospective cohort | Adult | Functional (DBS insertion) | To review authors’ experience in the treatment of 141 consecutive parkinsonian patients to identify surgical and hardware-related complications, infections, and delayed adverse medical events. |  | Yes | Not defined | Infections were seen in 5.6% of patients; removal of the hardware was necessary in 3.6%. | No |
| Viken H. et al., 2018 | [10.1016/j.wneu.2018.01.137](https://doi.org/10.1016/j.wneu.2018.01.137) | Norway | Retrospective | Adult | Neuro-oncology | To optimise follow-up and surveillance routines after intracranial surgery.  To explore when postoperative complications are detected after brain tumour surgery and assess their severity. |  | No | Not defined | Reported superficial wound infection in 17 cases (1.3%) and deep wound infection in 21 cases (1.6%). | No |
| Villavicencio A.T. et al., 2003 | [10.1016/s0090-3019(03)00070-3](https://doi.org/10.1016/s0090-3019(03)00070-3) | USA | Retrospective case-control | Paediatric | Trauma | To compare the rates of revision following ventricular catheter placement for shunted hydrocephalus with and without the use of endoscopy. | With Vs without endoscopy | Yes | Not defined | Shunt infection was not independently associated with endoscopic shunt placement. | No |
| Volsky P.G. et al., 2017 | [10.1002/lary.26403](https://doi.org/10.1002/lary.26403) | USA | Retrospective case-control | Adult | Skullbase | To investigate the long-term incidence of adverse events (CSF leaks and wound infections) associated with the use of hydroxyapatite cement cranioplasty (HAC). |  | Yes | Not defined | There were 7 cases of infection after HAC was used. | No |
| Wachter D. et al., 2013 | [10.1016/j.clineuro.2012.12.002](https://doi.org/10.1016/j.clineuro.2012.12.002) | Germany | Retrospective cohort | Adults and Paediatrics | Trauma | To identify surgery-associated complications after bone flap reimplantation. |  | Yes | Not defined | 70% of post-operative complications were in the form of wound infections. | No |
| Walaszek M., 2015 | PMID: 26519848 | Poland | Retrospective cohort | Adult | General (All cranial neurosurgical cases) | To assess the prevalence and structure of the healthcare-associated infections (HAI) in patients hospitalised in the neurosurgical ward in the St. Lukas District Hospital in Tarnów. |  | Yes | European Centre for Disease Prevention and Control (ECDC) and Centre for Disease Control and Prevention Guidelines | 516 cases of nosocomial infections were detected. The most common infections among these cases were SSI. The number of SSIs cases was 140 and cumulative incidence rate (CI) per 100 operations was 1.72%, including: 52 cases of craniotomy (CRAN). | No |
| Walcott B. et al., 2013 | [10.3171/2013.8.JNS13703](https://doi.org/10.3171/2013.8.jns13703) | USA | Retrospective cohort | Adult | Other (dural closure) | To assess the contribution of choice of dural closure material, as well as other factors, to the incidence of infection and CSF leak. |  | Yes | Centre for Disease Control and Prevention Guidelines | 17 out of 399 patients developed a SSI and 12 patients developed a CSF leak. | No |
| Walcott B.P. et al., 2013 | [10.3171/2013.1.JNS121626](https://doi.org/10.3171/2013.1.jns121626) | USA | Retrospective cohort | Adults and Paediatrics | General (Cranioplasty) | To identify surgery- and patient-specific risk factors related to the development of SSI and other complications following cranioplasty. |  | Yes | Centre for Disease Control and Prevention Guidelines | Re-operation was required in 17 out of 239 patients due to infection. | No |
| Walcott B.P. et al., 2013 | [10.3171/2013.8.JNS13703](https://doi.org/10.3171/2013.8.jns13703) | USA | Retrospective cohort | Adult | General (Craniotomy) | To assess the contribution of choice of dural closure material, as well as other factors, to the incidence of infection and CSF leak. |  | Yes | Centre for Disease Control and Prevention Guidelines | Out of 399 patients, 17 patients developed a surgical site infection and 12 patients developed a CSF leak. | No |
| Wang D.D. et al., 2017 | [10.3171/2016.9.JNS16149](https://doi.org/10.3171/2016.9.jns16149) | USA | Prospective cohort | Not reported | Vascular | To directly compare long-term pain control rates for first-time surgical treatments for idiopathic trigeminal neuralgia and to identify predictors of pain control. |  | No | Not defined | There were 6 wound infections (including 1 case with concurrent CSF leak). | No |
| Wang J. et al., 2023 | [10.1080/02688697.2021.1902472](https://doi.org/10.1080/02688697.2021.1902472) | China | Retrospective case-control | Adult | General (Craniotomy) | To verify whether the gentamycin irrigation would reduce the postoperative SSI rate in a consecutive cohort of emergency neurosurgery patients. | Only saline irrigation vs saline and gentamycin irrigation | Yes | 1. Purulence  2. Bacterial culture of wound exudation  3. Positive result of bacterial growth culture of CSF | In the gentamycin irrigation group, there were two postoperative SSIs within 28 days after surgery, yielding an SSI rate of 1.1%. Among the 265 remaining cases with saline only irrigation, 22 postoperative SSIs occurred within 28 days after surgery and the rate of SSI was 8.3%. The implementation of gentamycin irrigation, the SSI rate could be reduced by 86.7%. | Yes: “several researchers have proposed that a longer period of surveillance in neurosurgical procedures is likely to detect a truer rate of SSI.” |
| Wang L. et al., 2017 | [10.3171/2016.9.JNS16559](https://doi.org/10.3171/2016.9.jns16559) | China | Retrospective cohort | Adult | Skullbase | To investigate the factors associated with skullbase chordoma resection extent by evaluating their clinical features and surgical outcomes. |  | Yes | Not defined | 3 patients had wound infections. | No |
| Wang L. et al., 2022 | [10.1016/j.jclinane.2021.110575](https://doi.org/10.1016/j.jclinane.2021.110575) | China | Prospective case-control | Adult | General (Craniotomy) | To evaluate the clinical effectiveness and safety of evidence-based enhanced recovery after elective craniotomy. |  | No | Not defined | 1 person developed SSI in ERAS group vs 2 in the control group. | No |
| Wang Y. et al., 2022 | [10.3389/fonc.2022.860257](https://doi.org/10.3389/fonc.2022.860257) | China | Randomised clinical trial | Adult | Neuro-oncology | To design a multidisciplinary enhanced recovery after surgery (ERAS) protocol for glioma patients undergoing elective craniotomy and evaluate its clinical efficacy and safety after implementation in a tertiary neurosurgical center in China. |  | No | Not defined | 3 patients developed an incisional infection or subcutaneous effusion (1 in the ERAS group and 2 in the control group). 5 patients (2 in the ERAS group and 3 in the control group) presented with intracranial infection. | No |
| Weber L. et al., 2022 | [10.3389/fonc.2022.959072](https://doi.org/10.3389/fonc.2022.959072) | Switzerland | Prospective cohort | Adult | Neuro-oncology | To determine the association of adverse events with the timing and choice of subsequent treatments as well as with overall survival (OS). |  | No | Not defined | Adverse events such as SSIs reduce the rate of successful and uninterrupted chemoradiotherapy and ultimately limit OS. | No |
| Westman M. et al., 2018 | [10.1016/j.jocn.2018.04.076](https://doi.org/10.1016/j.jocn.2018.04.076) | Finland | Retrospective cohort | Adults and Paediatrics | General (All neurosurgical cases) | To focus on SSIs after neurosurgical operations, and to determine whether the checklist implementation would have an impact on the reported SSIs. |  | Yes | Not defined | The percentage of SSIs was 4.1% before and 4.5% after the checklist implementation. The overall incidence of SSIs of all neurosurgical patients did not differ (4.1% and 4.5%, respectively) and no differences were noticed in the incidences of the subgroups of superficial SSIs, deep SSIs, and deep organ SSIs. | No |
| Whitby M. et al., 2000 | [10.1080/02688690042843](https://doi.org/10.1080/02688690042843) | Australia | Prospective, randomised study | Adult | All neurosurgical cases (elective craniotomy, shunt surgery, stereotactic surgery) | To compare the efficacy of cefotaxime and trimethoprim/ sulfamethoxazole in the prevention of infective complications following neurosurgical procedures. | Cefotaxime vs Trimethoprim-sulfamethoxazole | Yes | Presence of an oral or tympanic temperature of greater than 38 degrees with reddening or weeping of the surgical wound, and a positive microbiological culture. Presence of any signs or symptoms, e.g., meningitis. Classified as Grade I (clinical inflammation with serous discharge, but no wound breakdown), Grade II (purulent or mucopurulent discharge and superficial minor wound breakdown) or Grade III (purulent discharge and major wound breakdown). | 613 patients were available for analysis, 315 received cefotaxime and 298 received trimethoprim- sulfamethoxazole. Forty-two patients (6.9%) experienced 49 postoperative infections, with no significant difference between treatment groups. 15 neurosurgical infections occurred, comprising 11 wound infections, two shunt infections and two cerebral abscesses. Neurosurgical infection rates were similar in the cefotaxime group (2.5%) and the trimethoprim- sulfamethoxazole group (2.3%). | No |
| White-Dzuro G.A. et al., 2016 | [10.1159/000442893](https://doi.org/10.1159/000442893) | USA | Retrospective cohort | Adult | Functional (DBS insertion) | To investigate the rate of infection and hardware complications for both the novel lead fixation method and the StimLoc TM securing device. |  | Yes | SSIs were defined as those occurring within 12 months of the original implant. Evidence of device involvement was assumed if there was cellulitis or purulent drainage from the incision over a device implant. Every incidence of SSIs required surgical revision and microbiological cultures from the hardware and confirm the presence of an infection. | 9 patients (3.1%) developed surgical site infections (SSIs), 4 (1.3%) with SSI of the internal pulse generator pocket. | No |
| Widen J. et al., 2017 | [10.1007/s00701-016-3039-2](https://doi.org/10.1007/s00701-016-3039-2) | Sweden | Retrospective cohort | Adult | Vascular | To investigate the incidence and bacteriological aetiology of ventriculostomy-related infections (VRI) in patients with sub-arachnoid haemorrhage (SAH) that have been treated with a ventriculostomy catheter.  To investigate the extent of empirical antibiotic treatment for VRI and adherence to local guidelines for VRI diagnostics and treatment. |  | Yes | Positive CSF culture along with inflammatory parameters above a pre-defined threshold in CSF. The definition of inflammation in CSF included measurement of parameters (CSF polynuclear leucocytes, CSF lactate, CSF plasma glucose ratio, CSF albumin). | 11 patients developed VRI, resulting in an incidence of 5.8% per patient, 5.4% per ventriculostomy catheter, and 4.1 per 1000 catheter days. | No |
| Williams M.A. et al., 2022 | [10.3171/2022.1.JNS212782](https://doi.org/10.3171/2022.1.jns212782) | USA and Canada | Prospective cohort | Adult | CSF dynamics | To describe the processes and outcomes associated with patients at five sites in the Adult Hydrocephalus Clinical Research Network (AHCRN) who had undergone evaluation and treatment for suspected idiopathic normal pressure hydrocephalus (iNPH) and had 1-year postoperative follow-up. |  | No | Not defined | 11 patients (5.7%) had 14 serious complications that resulted in the need for surgery or an extended hospital stay, including 1 instance of meningitis, 1 wound infection and 1 instance of sepsis. | No |
| Winston K.R. et al., 2007 | [10.3171/ped.2007.106.6.450](https://doi.org/10.3171/ped.2007.106.6.450) | USA | Retrospective cohort | Adults and Paediatrics | General (All neurosurgical cases) | To evaluate the efficacy of bandaging cranial incisional wounds. |  | Yes | Centre for Disease Control and Prevention Guidelines | The infection rate for the 626 clean cases was 0.48% and for the 38 clean–contaminated cases was 2.63%. | No |
| Wu C. et al., 2014 | [10.5137/1019-5149.JTN.9281-13.1](https://doi.org/10.5137/1019-5149.jtn.9281-13.1) | China | Retrospective case-control | Adult | General (All neurosurgical cases) | To compare the effects of post neurosurgical antimicrobial prophylaxis before and after the adoption of a new protocol, which changed from prolonged prophylaxis to limited usage. | Prolonged vs limited prophylaxis | Yes | Centre for Disease Control and Prevention Guidelines | No difference in SSI prevalence between the 2 groups. | No |
| Xia Y. et al., 2019 | [10.1093/ons/opz163](https://doi.org/10.1093/ons/opz163) | USA | Retrospective cohort | Adult | Vascular | To analyse the potential for vascular complications when the superior petrosal vein (SPV) is sacrificed during microvascular decompression. | SPV preserved Vs SPV sacrificed | No | Not defined | 8 cases (2.1%) of wound infection in SPV preserved group and 3 (1.4%) cases in SPV sacrificed group. | No |
| Xu L et al., 2022 | [10.1186/s12879-022-07719-2](https://doi.org/10.1186/s12879-022-07719-2) | China | Retrospective cohort | Adults and Paediatrics | CSF dynamics | To investigate the clinical features and risk factors of ventriculoperitoneal shunt (VPS) associated SSIs in HIV-negative patients with cryptococcal meningitis (CM). |  | Yes | Centre for Disease Control and Prevention Guidelines | 25 (12.95%) had SSIs in 6 days after operation. Compared with patients without SSIs, patient with SSIs tended to be shorter preoperative stay. | No |
| Yang M et al., 2020 | [10.2217/3dp-2019-0022](https://doi.org/10.2217/3dp-2019-0022) | Singapore | Retrospective cohort | Adult | Trauma | To evaluate the safety of using polycaprolactone (PCL) burr-hole covers over a 10-year period. |  | Yes | Not defined | 1 reoperation due to MRSA wound infection reported. | No |
| Yang N.R. et al., 2018 | [10.1016/j.wneu.2017.10.117](https://doi.org/10.1016/j.wneu.2017.10.117) | Korea | Retrospective cohort | Adult | Trauma | To determine the earliest time points for cranioplasty in patients with traumatic brain injury who have been treated with decompressive craniotomy and in whom early cranioplasty is recommended. |  | Yes | Not defined | 17 revisions were done due to infection when cranioplasty was done 35 days after the decompressive craniectomy. | No |
| Yatimparvar G. et al., 2020 | P J M H S Vol. 14, NO. 2, APR – JUN 2020. | Iran | Prospective case-control | Adult | General (Craniotomy) | To examine the effect of topical vancomycin powder on the prevention of operative infections in craniotomy surgeries. | With and without vancomycin powder | Yes | Not clearly defined | SSI occurred in 7 (7%) cases in the vancomycin group and 13 (13%) cases in the control group, but it was not statistically significant. | No |
| Yeap M.C. et al., 2022 | [10.1016/j.wneu.2021.09.111](https://doi.org/10.1016/j.wneu.2021.09.111) | Taiwan | Retrospective cohort | Adult | General (Cranioplasty) | To assess the predictive value of swab cultures of cryopreserved skull flaps during cranioplasties for SSIs. | . | Yes | Centre for Disease Control and Prevention Guidelines | The study included 422 patients categorised into two groups: swab and non-swab, depending on whether swab cultures were implemented during cranioplasties. The overall infection rate was 7.58%. No difference was seen in infection rates between groups. The results showed high specificity but low sensitivity for swab cultures to predict SSI occurrence and the pathogens. | No |
| Yeung L.C. et al., 2005 | [10.1227/01.neu.0000156472.29749.b8](https://doi.org/10.1227/01.neu.0000156472.29749.b8) | USA | Retrospective cohort | Paediatric | Paediatric | To determine the incidence of and factors associated with SSIs after intracranial surgery for craniofacial malformations at a single multidisciplinary craniofacial centre during a 6-year period. |  | Yes | Centre for Disease Control and Prevention Guidelines | SSIs occurred in 9 (3.2%) of 281 intracranial procedures that took place during the study period. | No |
| Youn S.B. et al., 2023 | [10.3340/jkns.2023.0024](https://doi.org/10.3340/jkns.2023.0024) | South Korea | Retrospective case-control | Adults and Paediatrics | Trauma | To evaluate the effect of intrawound vancomycin powder application for preventing SSI following cranioplasty compared with the conventional procedure without topical antibiotics. | With and without vancomycin powder | Yes | Not defined | 31 patients under conventional group experienced SSI. No patient in vancomycin group experienced infection. | No |
| Yusufali T.S. et al., 2016 | The ANNALS of AFRICAN SURGERY. January 2016 Volume 13 Issue 1 | Kenya | Prospective | Adults and Paediatrics | Trauma | To determine the utility of Surgical Apgar Score (SAS) in predicting postoperative complications in neurotrauma patients. |  | No | Not defined | 24 cases of SSI (7.2%) reported. | No |
| Zaidi H.A. et al., 2017 | [10.1093/neuros/nyw139](https://doi.org/10.1093/neuros/nyw139) | USA | Retrospective cohort | Adult | Vascular | To evaluate the impact of surgical timing and predictors of neurological outcome after surgical resection of brain stem cavernous malformations. |  | No | Not defined | 10 cases of wound infection reported. | No |
| Zakhary G. et al., 2014 | [10.1016/j.jcms.2014.05.014](https://doi.org/10.1016/j.jcms.2014.05.014) | USA | Retrospective cohort | Paediatric | Paediatric | To provide outcome data for open cranial vault reshaping at a single institution by a single craniofacial surgeon treating 100 patients. |  | No | Not defined | 2 wound infections and 1 subgaleal abscess requiring drainage (out of 100 patients) reported. | No |
| Zanaty M. et al., 2015 | [10.3171/2014.9.JNS14405](https://doi.org/10.3171/2014.9.jns14405) | USA | Retrospective cohort | Adults and Paediatrics | Trauma | To evaluate risk factors that predispose patients to an increased risk of cranioplasty complications and death. |  | Yes | Not defined | Increasing age, diabetes mellitus, haemorrhagic stroke, post-cranioplasty hydrocephalus and reoperation for hematoma evacuation were predictive of infection. | No |
| Zeng L. et al., 2015 | [10.1007/s10143-015-0619-1](https://doi.org/10.1007/s10143-015-0619-1) | China | Retrospective cohort | Adult | Neuro-oncology | To summarise the characteristics of the patients with asymptomatic meningiomas, evaluate the effects of the surgery, and identify factors associated with postsurgical complications and patient prognosis to improve the selection of asymptomatic patients for surgery. |  | Yes | Not defined | 1 wound infection reported. | No |
| Zhang L. et al., 2021 | [10.1097/MAO.0000000000003215](https://doi.org/10.1097/mao.0000000000003215) | USA | Retrospective cohort | Adults and Paediatrics | Neuro-oncology | To describe rates of cerebrospinal fluid (CSF) rhinorrhoea after reconstruction of the internal auditory canal with calcium phosphate bone cement during retrosigmoid resections of vestibular schwannomas. |  | Yes | Not defined | 3 patients were reported to have developed SSI. | No |
| Zhang Y. et al., 2011 | [10.1016/j.jocn.2011.01.026](https://doi.org/10.1016/j.jocn.2011.01.026) | China | Retrospective cohort | Adults and Paediatrics | Skullbase | To assess the short-term and long-term efficacies of the two modes of posterior fossa decompression. |  | Yes | Not defined | Reported 1 wound infection in the large craniotomy group, and none in small craniotomy group. | No |
| Zhao H. et al., 2017 | [10.1016/j.wneu.2017.08.028](https://doi.org/10.1016/j.wneu.2017.08.028) | China | Retrospective cohort | Adult | Vascular | To analyse the microvascular decompression related complications and the number of occurrences of each complication. |  | Yes | Not defined | There were 24 (out of 1548) cases of CSF leakage, 13 cases of wound problem, 4 cases of infection and 1 cases of hemorrhage. 8 cases (0.52%) had wound infections. | No |
| Zheng W.-J. et al., 2018 | [10.1016/j.wneu.2018.08.172](https://doi.org/10.1016/j.wneu.2018.08.172) | China | Retrospective cohort | Adult | General (Craniotomy) | To identify perioperative risk factors for SSI in cranioplasty. |  | Yes | Fever, swelling, pain, and erythematous wound with purulent discharge; Positive bacterial culture of wound discharge.  Infections involving beyond the skin and subcutaneous tissue were defined as deep or central nervous system infection. | The overall superficial SSI rate was 10.3% (16 of 155 patients). Wound discharge of 4 patients was cultured, and 2 cultures were positive. | No |
| Zhong C. et al., 2023 | [10.12669/pjms.39.6.7963](https://doi.org/10.12669/pjms.39.6.7963) | China | Retrospective cohort | Adults and Paediatrics | General (All neurosurgical cases) | To explore the independent risk factors of poor wound healing after craniocerebral surgery, and to generate a risk prediction model. |  | Yes | Not defined | The duration of operation, SSI, diabetes mellitus, and the time of intubation were significantly different between the two groups. | No |
| Zhou H. et al. 2013 | [10.1016/j.jcms.2013.01.006](https://doi.org/10.1016/j.jcms.2013.01.006) | China | Retrospective cohort | Adults and Paediatrics | Other (Frontal craniotomy) | To describe a new frontal sinus cavity reconstruction technique using medical aural and encephalic glue (EC glue)-soaked gelfoam. | . | No | Not defined | No patient developed intracranial infection, frontal sinusitis, or CSF leakage in the short term. | No |
| Zhou Y. et al., 2020 | [10.21037/atm.2020.03.221](https://doi.org/10.21037/atm.2020.03.221) | China | Retrospective cohort | Adult | Neuro-oncology | To describe an algorithm for selecting the best reconstructive categories and minimising complications according to the surgical defect of scalp tumours. |  | Yes | Not defined | Reported 2 cases of wound infection in the local flap cases. 1 instance of wound infection occurred in the skin graft. | No |
| Zohdy Y.M. et al., 2023 | [10.1016/j.wneu.2023.08.063](https://doi.org/10.1016/j.wneu.2023.08.063) | USA | Retrospective cohort | Adult | Neuro-oncology | To evaluate the predictive accuracy of frailty on 30-day unplanned readmission after intracranial tumor resection using the risk analysis index. |  | Yes | Not defined | Reported a rate of 2% for organ SSI and 1% for superficial incisional SSI. | No |
