# Supplementary material for: Heterogeneity in definitions of surgical site infection after cranial surgery limits the validity of research findings in neurosurgery: a systematic review
Source: Neurosurg Rev. 2025 Jan 16;48(1):59. doi: 10.1007/s10143-025-03218-5 (PMC11739257; doi:10.1007/s10143-025-03218-5)
Supplement: Supplementary file 2 — Supplementary Material 2 [file 10143_2025_3218_MOESM2_ESM.docx]

Supplementary Table 5. Joanna Briggs Institute quality assessment checklist for non-randomised studies

| **Author and year published** | **DOI** | **Was SSI-CRAN defined?** | **Were the two groups similar and recruited from the same population?** | **Were the exposures measured similarly to assign people to both exposed and unexposed groups?** | **Was the exposure measured in a valid and reliable way?** | **Were confounding factors identified?** | **Were strategies to deal with confounding factors stated?** | **Were the participants free of the outcome at the start of the study?** | **Were the outcomes measured in a valid and reliable way?** | **Was the follow up time reported and sufficient to be long enough for outcomes to occur?** | **Was follow up complete, and if not, were the reasons to loss to follow up described and explored?** | **Were strategies to address incomplete follow up utilised?** | **Was appropriate statistical analysis used?** | **Total Risk of Bias (out of 11)** |
| --- | --- | --- | --- | --- | --- | --- | --- | --- | --- | --- | --- | --- | --- | --- |
| Abdullah K G et al. 2015 | [10.3171/2014.12.JNS142092](https://doi.org/10.3171/2014.12.jns142092) | No | Yes | Yes | Yes | Yes | No | Yes | Yes | Yes | Yes | Yes | Yes | 10 |
| Abebe FT et al. 2022 | [10.1016/j.inat.2022.101602](https://doi.org/10.1016/j.inat.2022.101602) | No | Yes | Yes | Yes | No | No | Yes | Yes | No | Yes | Yes | Yes | 8 |
| Abecassis I et al. 2021 | [10.3171/2020.11.JNS203567](https://doi.org/10.3171/2020.11.jns203567) | No | Yes | Yes | Yes | Yes | Yes | Yes | Yes | Yes | Yes | Yes | Yes | 11 |
| Abode-Iyamah K O et al. 2018 | [10.3171/2016.12.JNS161967](https://doi.org/10.3171/2016.12.jns161967) | Yes | Yes | Yes | Yes | Yes | Yes | Yes | Yes | No | Yes | Yes | Yes | 10 |
| Abode-lyamah K.O. et al., 2018 | [10.3171/2017.9.JNS1780](https://doi.org/10.3171/2017.9.jns1780) | Yes | Yes | Yes | Yes | Yes | Yes | Yes | Yes | Yes | Yes | Yes | Yes | 11 |
| Abulhasan Y.B. et al., 2018 | [10.1016/j.wneu.2018.04.061](https://doi.org/10.1016/j.wneu.2018.04.061) | Yes | Yes | Yes | Yes | Yes | Yes | Yes | Yes | Yes | Yes | Yes | Yes | 11 |
| Adeleye A. 2016 | [10.1007/s10143-016-0700-4](https://doi.org/10.1007/s10143-016-0700-4) | No | Yes | Yes | Yes | No | No | Yes | Yes | Yes | Yes | Yes | Yes | 9 |
| Adeleye A. 2017 | [10.1080/02688697.2017.1407746](https://doi.org/10.1080/02688697.2017.1407746) | Yes | Yes | Yes | Yes | Yes | Yes | Yes | Yes | Yes | Yes | Yes | Yes | 11 |
| Agarwal N et al. 2017 | [10.1093/neuros/nyx273](https://doi.org/10.1093/neuros/nyx273) | Yes | Yes | Yes | Yes | Yes | No | Yes | Yes | Yes | Yes | Yes | Yes | 10 |
| Ahmad N et al. 2008 | [10.1097/SCS.0b013e31816ae358](https://doi.org/10.1097/scs.0b013e31816ae358) | Yes | Yes | Yes | Yes | No | No | Yes | Yes | Yes | No | No | Yes | 7 |
| Ahmadi R et al., 2021 | [10.3109/02688697.2016.1161726](https://doi.org/10.3109/02688697.2016.1161726) | No | Yes | Yes | Yes | Yes | Yes | Yes | Yes | No | Yes | Yes | Yes | 10 |
| Al-Sharydah AM et al. 2020 | [10.15537/smj.2020.6.25095](https://doi.org/10.15537/smj.2020.6.25095) | Yes | Yes | Yes | Yes | No | No | Yes | Yes | No | Yes | Yes | Yes | 8 |
| Al-Tamini YZ et al, 2012 | [10.3109/02688697.2011.633640](https://doi.org/10.3109/02688697.2011.633640) | No | Yes | Yes | Yes | No | No | Yes | Yes | Yes | Yes | Yes | Yes | 9 |
| Alan N et al. 2015 | [10.1016/j.jocn.2015.03.009](https://doi.org/10.1016/j.jocn.2015.03.009) | No | Yes | Yes | Yes | Yes | Yes | Yes | Yes | No | Yes | Yes | Yes | 10 |
| Alford E N et al. 2020 | [10.1007/s10143-020-01296-1](https://doi.org/10.1007/s10143-020-01296-1) | No | Yes | Yes | Yes | Yes | Yes | Yes | Yes | No | Yes | Yes | Yes | 10 |
| Alkhaibary A et al. 2019 | [10.1016/j.wneu.2019.09.120](https://doi.org/10.1016/j.wneu.2019.09.120) | Yes | Yes | Yes | Yes | No | No | Yes | Yes | Yes | Yes | Yes | Yes | 9 |
| Alwadei A et al. 2019 | [10.1016/j.wneu.2019.01.262](https://doi.org/10.1016/j.wneu.2019.01.262) | No | Yes | Yes | Yes | No | No | Yes | Yes | Yes | Yes | Yes | Yes | 9 |
| Alzoubi F et al. 2015 | [10.1179/1754762814Y.0000000090](https://doi.org/10.1179/1754762814y.0000000090) | No | Yes | Yes | Yes | No | No | Yes | Yes | Yes | Yes | Yes | Yes | 9 |
| Ammanuel SG et al., 2021 | [10.3171/2020.10.JNS201255](https://doi.org/10.3171/2020.10.jns201255) | Yes | Yes | Yes | Yes | Yes | Yes | Yes | Yes | No | Yes | Yes | Yes | 10 |
| Ammar R. Et al., 2022 | [10.1016/j.inat.2021.101421](https://doi.org/10.1016/j.inat.2021.101421) | No | Yes | Yes | Yes | Yes | Yes | Yes | Yes | Yes | Yes | Yes | Yes | 11 |
| Andren K. Et al., 2018 | [10.1007/s00415-017-8680-z](https://doi.org/10.1007/s00415-017-8680-z) | No | Yes | Yes | Yes | Yes | Yes | Yes | Yes | Yes | Yes | No | Yes | 10 |
| Anegbe A.O. et al., 2019 | [10.1007/s00381-019-04219-8](https://doi.org/10.1007/s00381-019-04219-8) | No | Yes | Yes | Yes | Yes | Yes | Yes | Yes | No | Yes | Yes | Yes | 10 |
| Ansari S et al. 2020 | [10.1093/ons/opaa217](https://doi.org/10.1093/ons/opaa217) | No | Yes | Yes | Yes | No | No | Yes | Yes | Yes | Yes | Yes | Yes | 9 |
| Aras M et al., 2014 | [0.1016/j.clineuro.2014.08.019](https://doi.org/10.1016/j.clineuro.2014.08.019) | No | Yes | Yes | Yes | No | No | Yes | Yes | No | Yes | Yes | Yes | 8 |
| Arita H et al. 2014 | [10.1007/s11060-013-1273-5](https://doi.org/10.1007/s11060-013-1273-5) | No | Yes | Yes | Yes | Yes | Yes | Yes | Yes | Yes | Yes | Yes | Yes | 11 |
| Arnautovic KI et al. 2020 | [10.1016/j.wneu.2020.11.102](https://doi.org/10.1016/j.wneu.2020.11.102) | No | Yes | Yes | Yes | No | No | Yes | Yes | Yes | Yes | Yes | Yes | 9 |
| Arnone G et al. 2020 | [10.1055/s-0040-1715811](https://doi.org/10.1055/s-0040-1715811) | No | Yes | Yes | Yes | No | No | Yes | Yes | Yes | Yes | Yes | Yes | 9 |
| Arocho-Quinones EV et al., 2019 | [10.1016/j.wneu.2019.04.003](https://doi.org/10.1016/j.wneu.2019.04.003) | Yes | Yes | Yes | Yes | No | No | Yes | Yes | Yes | Yes | Yes | Yes | 9 |
| Arts S. Et al., 2018 | [10.3171/2017.7.PEDS17155](https://doi.org/10.3171/2017.7.peds17155) | No | Yes | Yes | Yes | No | No | Yes | Yes | No | Yes | Yes | Yes | 8 |
| Attenello F et al. 2008 | [10.1245/s10434-008-0048-2](https://doi.org/10.1245/s10434-008-0048-2) | No | Yes | Yes | Yes | No | No | Yes | Yes | No | Yes | Yes | Yes | 8 |
| Aum D J et al. 2023 | [10.1111/epi.17679](https://doi.org/10.1111/epi.17679) | No | Yes | Yes | Yes | No | No | Yes | Yes | Yes | Yes | Yes | Yes | 9 |
| Bakhsheshian J et al. 2018 | [10.1016/j.wneu.2018.07.134](https://doi.org/10.1016/j.wneu.2018.07.134) | No | Yes | Yes | Yes | Yes | Yes | Yes | Yes | No | Yes | Yes | Yes | 10 |
| Ban SP et al. 2018 | [10.1136/neurintsurg-2021-017352](https://doi.org/10.1136/neurintsurg-2021-017352) | No | Yes | Yes | Yes | No | No | Yes | Yes | Yes | Yes | Yes | Yes | 9 |
| Barone D.G. et al., 2014 | [10.1002/14651858.CD009685.pub2](https://doi.org/10.1002/14651858.cd009685.pub2) | No | Yes | Yes | Yes | No | No | Yes | Yes | Yes | Yes | Yes | Yes | 9 |
| Bartek Jr J et al. 2017 | [10.1016/j.wneu.2017.07.044](https://doi.org/10.1016/j.wneu.2017.07.044) | No | Yes | Yes | Yes | Yes | Yes | Yes | Yes | No | Yes | Yes | Yes | 10 |
| Bass D et al. 2019 | [10.1016/j.wneu.2019.08.066](https://doi.org/10.1016/j.wneu.2019.08.066) | No | Yes | Yes | Yes | Yes | Yes | Yes | Yes | No | Yes | Yes | Yes | 10 |
| Batzdorf U et al. 2013 | [10.3171/2012.10.JNS12305](https://doi.org/10.3171/2012.10.jns12305) | No | Yes | Yes | Yes | Yes | No | Yes | Yes | Yes | Yes | Yes | Yes | 10 |
| Baum G.R. et al., 2017 | [10.3171/2016.9.JNS16367](https://doi.org/10.3171/2016.9.jns16367) | No | Yes | Yes | Yes | Yes | Yes | Yes | Yes | Yes | Yes | Yes | Yes | 11 |
| Beckman J et al. 2015 | [10.3171/2014.12.PEDS13675](https://doi.org/10.3171/2014.12.peds13675) | No | Yes | Yes | Yes | Yes | Yes | Yes | Yes | Yes | Yes | Yes | Yes | 11 |
| Bekar A et al. 2001 | [10.1007/s007010170057](https://doi.org/10.1007/s007010170057) | Yes | No | Yes | Yes | No | No | Yes | Yes | No | Yes | Yes | Yes | 7 |
| Bekelis K et al. 2015 | [10.1016/j.wneu.2015.02.032](https://doi.org/10.1016/j.wneu.2015.02.032) | No | Yes | Yes | Yes | Yes | Yes | Yes | Yes | No | Yes | Yes | Yes | 10 |
| Bekelis K et al. 2017 | [10.1007/s11060-013-1089-3](https://doi.org/10.1007/s11060-013-1089-3) | No | Yes | Yes | Yes | Yes | Yes | Yes | Yes | No | Yes | Yes | Yes | 10 |
| Berghauser Pont LME et al., 2012 | [10.1227/NEU.0b013e31823672ad](https://doi.org/10.1227/neu.0b013e31823672ad) | No | Yes | Yes | Yes | Yes | Yes | Yes | Yes | Yes | Yes | Yes | Yes | 11 |
| Bhaskar IP et al., 2014 | [10.1016/j.wneu.2013.01.013](https://doi.org/10.1016/j.wneu.2013.01.013) | No | Yes | Yes | Yes | No | No | Yes | Yes | No | Yes | Yes | Yes | 8 |
| Bhatti M et al. 2012 | [10.3109/02688697.2012.743968](https://doi.org/10.3109/02688697.2012.743968) | Yes | Yes | Yes | Yes | No | No | Yes | Yes | Yes | Yes | Yes | Yes | 9 |
| Bhimani A.D. et al., 2019 | [10.1016/j.wneu.2018.09.079](https://doi.org/10.1016/j.wneu.2018.09.079) | No | Yes | Yes | Yes | Yes | Yes | Yes | Yes | No | Yes | Yes | Yes | 10 |
| Bhimani AD et al., 2018 | [10.1016/j.wneu.2018.04.077](https://doi.org/10.1016/j.wneu.2018.04.077) | No | Yes | Yes | Yes | Yes | Yes | Yes | Yes | No | Yes | Yes | Yes | 10 |
| Bjellvi J et al, 2015 | [10.3171/2014.9.JNS132679](https://doi.org/10.3171/2014.9.jns132679) | No | Yes | Yes | Yes | No | No | Yes | Yes | Yes | Yes | Yes | Yes | 9 |
| Bjerknes S et al. 2014 | [10.1371/journal.pone.0105288](https://doi.org/10.1371/journal.pone.0105288) | Yes | Yes | Yes | Yes | No | No | Yes | Yes | No | Yes | Yes | Yes | 8 |
| Blomstedt P. Et al., 2005 | [10.1007/s00701-005-0576-5](https://doi.org/10.1007/s00701-005-0576-5) | No | Yes | Yes | Yes | No | No | Yes | Yes | No | Yes | Yes | Yes | 8 |
| Bonfield C M et al. 2014 | [10.3171/2014.6.PEDS13682](https://doi.org/10.3171/2014.6.peds13682) | No | Yes | Yes | Yes | Yes | Yes | Yes | Yes | Yes | Yes | Yes | Yes | 11 |
| Borger V et al. 2021 | [10.3171/2020.7.JNS20284](https://doi.org/10.3171/2020.7.JNS20284) | No | Yes | Yes | Yes | Yes | Yes | Yes | Yes | Yes | Yes | Yes | Yes | 11 |
| Branch L.G. et al., 2017 | [10.1097/SCS.0000000000003166](https://doi.org/10.1097/scs.0000000000003166) | No | Yes | Yes | Yes | No | No | Yes | Yes | Yes | Yes | Yes | Yes | 9 |
| Brokinkel B et al. 2021 | [10.1016/j.clineuro.2020.106315](https://doi.org/10.1016/j.clineuro.2020.106315) | No | Yes | Yes | Yes | Yes | Yes | Yes | Yes | Yes | Yes | Yes | Yes | 11 |
| Brommeland T et al., 2015 | [10.1186/s13049-015-0155-6](https://doi.org/10.1186/s13049-015-0155-6) | No | Yes | Yes | Yes | Yes | Yes | Yes | Yes | Yes | Yes | Yes | Yes | 11 |
| Buang SS et al. 2012 | PMID: 23082448 | Yes | Yes | Yes | Yes | Yes | Yes | Yes | Yes | Yes | Yes | Yes | Yes | 11 |
| Buchanan I A et al. 2018 | [10.1016/j.wneu.2018.08.102](https://doi.org/10.1016/j.wneu.2018.08.102) | No | Yes | Yes | Yes | Yes | Yes | Yes | Yes | No | Yes | Yes | Yes | 10 |
| Byoun H.S. et al., 2019 | [10.1016/j.clineuro.2019.105503](https://doi.org/10.1016/j.clineuro.2019.105503) | No | Yes | Yes | Yes | No | No | Yes | Yes | No | Yes | Yes | Yes | 8 |
| Cacciola F et al., 2001 | [10.1179/joc.2001.13.Supplement-2.119](https://doi.org/10.1179/joc.2001.13.supplement-2.119) | Yes | Yes | Yes | Yes | No | No | Yes | Yes | No | Yes | Yes | Yes | 8 |
| Campbell E et al. 2017 | [10.1007/s00381-017-3358-5](https://doi.org/10.1007/s00381-017-3358-5) | No | Yes | Yes | Yes | No | No | Yes | Yes | No | Yes | Yes | Yes | 8 |
| Campioli C et al. 2022 | [10.1017/ash.2021.258](https://doi.org/10.1017/ash.2021.258) | Yes | Yes | Yes | Yes | Yes | Yes | Yes | Yes | No | Yes | Yes | Yes | 10 |
| Cao Y. Et al., 2020 | [10.1186/s13756-020-00784-9](https://doi.org/10.1186/s13756-020-00784-9) | No | Yes | Yes | Yes | Yes | Yes | Yes | Yes | No | Yes | Yes | Yes | 10 |
| Carlson J.D. et al., 2019 | [10.1016/j.wneu.2019.04.233](https://doi.org/10.1016/j.wneu.2019.04.233) | Yes | Yes | Yes | Yes | No | No | Yes | Yes | No | Yes | Yes | Yes | 8 |
| Catapano J et al. 2019 | [10.1016/j.wneu.2019.07.183](https://doi.org/10.1016/j.wneu.2019.07.183) | No | Yes | Yes | Yes | No | No | Yes | Yes | No | Yes | Yes | Yes | 8 |
| Cater DT et al. 2021 | [10.3171/2021.10.PEDS21291](https://doi.org/10.3171/2021.10.peds21291) | No | Yes | Yes | Yes | Yes | Yes | Yes | Yes | No | Yes | Yes | Yes | 10 |
| Chacon-Quesada T. Et al., 2021 | [10.1007/s10143-021-01513-5](https://doi.org/10.1007/s10143-021-01513-5) | No | Yes | Yes | Yes | Yes | Yes | Yes | Yes | Yes | Yes | Yes | Yes | 11 |
| Chaichana K L et al. 2015 | [10.1179/1743132815Y.0000000042](https://doi.org/10.1179/1743132815y.0000000042) | Yes | Yes | Yes | Yes | Yes | Yes | Yes | Yes | Yes | No | No | Yes | 9 |
| Champeaux C. Et al., 2020 | [10.1007/s11060-020-03410-1](https://doi.org/10.1007/s11060-020-03410-1) | No | Yes | Yes | Yes | Yes | Yes | Yes | Yes | No | Yes | Yes | Yes | 10 |
| Chang S M et al. 2003 | [10.3171/jns.2003.98.6.1175](https://doi.org/10.3171/jns.2003.98.6.1175) | No | Yes | Yes | Yes | No | No | Yes | Yes | Yes | Yes | Yes | Yes | 9 |
| Chaturvedi D. Et al., 2019 | 10.1055/s-0039-1680276 | No | Yes | Yes | Yes | Yes | Yes | Yes | Yes | Yes | Yes | Yes | Yes | 11 |
| Cheah PP et al., 2017 | [10.21315/mjms2017.24.6.8](https://doi.org/10.21315/mjms2017.24.6.8) | Yes | Yes | Yes | Yes | Yes | Yes | Yes | Yes | Yes | Yes | Yes | Yes | 11 |
| Chen C et al. 2016 | 10.1097/MD.0000000000004329 | Yes | Yes | Yes | Yes | Yes | Yes | Yes | Yes | Yes | Yes | Yes | Yes | 11 |
| Chen H. Et al., 2023 | [10.1007/s00586-023-07729-x](https://doi.org/10.1007/s00586-023-07729-x) | No | Yes | Yes | Yes | Yes | Yes | Yes | Yes | Yes | Yes | Yes | Yes | 11 |
| Chen P et al., 2021 | [10.1155/2021/4948664](https://doi.org/10.1155/2021/4948664) | No | Yes | Yes | Yes | No | No | Yes | Yes | Yes | Yes | Yes | Yes | 9 |
| Chen R et al. 2023 | [10.1227/neu.0000000000002376](https://doi.org/10.1227/neu.0000000000002376) | No | Yes | Yes | Yes | Yes | Yes | Yes | Yes | Yes | Yes | No | Yes | 10 |
| Chen Y et al. 2018 | [10.1016/j.wneu.2018.01.211](https://doi.org/10.1016/j.wneu.2018.01.211) | No | Yes | Yes | Yes | Yes | Yes | Yes | Yes | Yes | Yes | No | Yes | 11 |
| Cheng CH et al., 2014 | [10.1016/j.clineuro.2014.06.029](https://doi.org/10.1016/j.clineuro.2014.06.029) | Yes | Yes | Yes | Yes | No | No | Yes | Yes | Yes | Yes | No | Yes | 9 |
| Chiang HY et al., 2011 | [10.3171/2011.1.JNS10782](https://doi.org/10.3171/2011.1.jns10782) | Yes | Yes | Yes | Yes | Yes | No | Yes | Yes | Yes | Yes | No | Yes | 9 |
| Chiang HY et al. 2014 | [10.3171/2013.9.JNS13843](https://doi.org/10.3171/2013.9.jns13843) | No | Yes | Yes | Yes | Yes | Yes | Yes | Yes | Yes | Yes | Yes | Yes | 11 |
| Chibbaro S. Et al., 2011 | [10.1016/j.wneu.2010.10.020](https://doi.org/10.1016/j.wneu.2010.10.020) | No | Yes | Yes | Yes | No | No | Yes | Yes | Yes | Yes | Yes | Yes | 9 |
| Cho J et al., 2003 | [10.1227/01.neu.0000054219.35102.b4](https://doi.org/10.1227/01.neu.0000054219.35102.b4) | No | Yes | Yes | Yes | Yes | Yes | Yes | Yes | Yes | Yes | Yes | Yes | 11 |
| Choque-Velasquez J et al. 2020 | [10.1016/j.wneu.2020.01.137](https://doi.org/10.1016/j.wneu.2020.01.137) | No | Yes | Yes | Yes | Yes | Yes | Yes | Yes | Yes | Yes | Yes | Yes | 11 |
| Chotai S et al., 2023 | [10.3171/2022.4.JNS22290](https://doi.org/10.3171/2022.4.jns22290) | No | Yes | Yes | Yes | Yes | Yes | Yes | Yes | Yes | Yes | Yes | Yes | 11 |
| Clark A.J. et al., 2011 | [10.3171/2010.10.JNS101042](https://doi.org/10.3171/2010.10.jns101042) | No | Yes | Yes | Yes | Yes | No | Yes | Yes | Yes | Yes | Yes | Yes | 10 |
| Clune JE et al. 2010 | [10.1097/SCS.0b013e3181cf6103](https://doi.org/10.1097/scs.0b013e3181cf6103) | No | Yes | Yes | Yes | Yes | Yes | Yes | Yes | Yes | Yes | Yes | Yes | 11 |
| Cohen-Inbar O. Et al., 2014 | [10.1055/s-0034-1371516](https://doi.org/10.1055/s-0034-1371516) | No | Yes | Yes | Yes | No | No | Yes | Yes | Yes | Yes | Yes | Yes | 9 |
| Colombo F et al. 2023 | [10.1055/s-0043-1774720](https://doi.org/10.1055/s-0043-1774720) | Yes | Yes | Yes | Yes | No | No | Yes | Yes | Yes | Yes | Yes | Yes | 9 |
| Cosgrove G R et al. 2007 | [10.3171/jns.2007.106.1.52](https://doi.org/10.3171/jns.2007.106.1.52) | Yes | Yes | Yes | Yes | No | No | Yes | Yes | Yes | Yes | No | Yes | 8 |
| Cote D et al. 2016 | [10.1055/s-0036-1592306](https://doi.org/10.1055/s-0036-1592306) | No | Yes | Yes | Yes | No | No | Yes | Yes | Yes | Yes | Yes | Yes | 9 |
| Cote DJ et al., 2019 | [10.1016/j.wneu.2019.05.022](https://doi.org/10.1016/j.wneu.2019.05.022) | No | Yes | Yes | Yes | No | No | Yes | Yes | Yes | Yes | Yes | Yes | 9 |
| Coulter I C et al. 2014 | [10.1007/s00701-014-2081-1](https://doi.org/10.1007/s00701-014-2081-1) | Yes | Yes | Yes | Yes | No | No | Yes | Yes | Yes | Yes | Yes | Yes | 9 |
| Covell MM et al., 2023 | [10.1016/j.ejso.2023.107044](https://doi.org/10.1016/j.ejso.2023.107044) | No | Yes | Yes | Yes | No | No | Yes | Yes | Yes | Yes | Yes | Yes | 9 |
| Das KK et al., 2020 | [10.1016/j.wneu.2020.05.259](https://doi.org/10.1016/j.wneu.2020.05.259) | No | Yes | Yes | Yes | No | No | Yes | Yes | Yes | Yes | Yes | Yes | 9 |
| Dasenbrock H et al. 2017 | [10.3171/2016.2.PEDS15604](https://doi.org/10.3171/2016.2.peds15604) | No | Yes | Yes | Yes | No | No | Yes | Yes | Yes | Yes | Yes | Yes | 9 |
| Dasenbrock H et al., 2017 | [10.3171/2016.2.JNS152345](https://doi.org/10.3171/2016.2.jns152345) | No | Yes | Yes | Yes | Yes | Yes | Yes | Yes | Yes | Yes | Yes | Yes | 11 |
| Dasenbrock H H et al. 2016 | [10.1093/neuros/nyw062](https://doi.org/10.1093/neuros/nyw062) | No | Yes | Yes | Yes | No | No | Yes | Yes | Yes | Yes | Yes | Yes | 9 |
| Dasenbrock H H et al. 2017 | [10.1161/STROKEAHA.117.016702](https://doi.org/10.1161/strokeaha.117.016702) | No | Yes | Yes | Yes | No | No | Yes | Yes | Yes | Yes | Yes | Yes | 9 |
| Dasenbrock H H et al. 2017 | [10.1093/neuros/nyx089](https://doi.org/10.1093/neuros/nyx089) | No | Yes | Yes | Yes | No | No | Yes | Yes | Yes | Yes | Yes | Yes | 9 |
| Davies B.M. et al., 2016 | [10.1308/rcsann.2016.0143](https://doi.org/10.1308/rcsann.2016.0143) | Yes | Yes | Yes | Yes | Yes | Yes | Yes | Yes | Yes | Yes | Yes | Yes | 11 |
| Davies BM et al. 2015 | [10.3109/02688697.2015.1071321](https://doi.org/10.3109/02688697.2015.1071321) | Yes | Yes | Yes | Yes | No | No | Yes | Yes | Yes | Yes | Yes | No | 8 |
| Dechaene V et al., 2023 | [10.1016/j.ijid.2023.10.008](https://doi.org/10.1016/j.ijid.2023.10.008) | No | Yes | Yes | Yes | No | No | Yes | Yes | Yes | Yes | Yes | Yes | 9 |
| Delgado-Lopez P.D. et al., 2009 | [10.1016/s1130-1473(09)70154-x](https://doi.org/10.1016/s1130-1473(09)70154-x) | No | Yes | Yes | Yes | No | No | Yes | Yes | Yes | Yes | Yes | No | 8 |
| Delong MR. Et al., 2014 | [10.1001/jamaneurol.2014.1272](https://doi.org/10.1001/jamaneurol.2014.1272) | No | Yes | Yes | Yes | No | No | Yes | Yes | Yes | Yes | Yes | Yes | 9 |
| Di L et al. 2023 | [10.3171/2022.3.JNS212399](https://doi.org/10.3171/2022.3.jns212399) | No | Yes | Yes | Yes | Yes | Yes | Yes | Yes | Yes | Yes | Yes | Yes | 11 |
| Dickinson H et al. 2015 | [10.3171/2014.8.JNS1498](https://doi.org/10.3171/2014.8.jns1498) | No | Yes | Yes | Yes | Yes | Yes | Yes | Yes | Yes | Yes | Yes | Yes | 11 |
| Dinevski N et al. 2017 | [10.1016/j.wneu.2017.03.093](https://doi.org/10.1016/j.wneu.2017.03.093) | Yes | Yes | Yes | Yes | No | No | Yes | Yes | Yes | Yes | Yes | Yes | 9 |
| Donnelly BM et al., 2023 | [10.1007/s00701-023-05764-7](https://doi.org/10.1007/s00701-023-05764-7) | No | Yes | Yes | Yes | Yes | Yes | Yes | Yes | Yes | Yes | Yes | Yes | 11 |
| Doshi P. 2011 | [10.1159/000323372](https://doi.org/10.1159/000323372) | No | Yes | Yes | Yes | No | No | Yes | Yes | Yes | Yes | Yes | No | 8 |
| Dowlati E et al., 2022 | [10.3171/2022.3.JNS212637](https://doi.org/10.3171/2022.3.jns212637) | No | Yes | Yes | Yes | Yes | Yes | Yes | Yes | Yes | Yes | Yes | Yes | 11 |
| Drexler R. Et al., 2024 | [10.1227/neu.0000000000002689](https://doi.org/10.1227/neu.0000000000002689) | No | Yes | Yes | Yes | No | No | Yes | Yes | Yes | No | No | Yes | 7 |
| Ebel F et al. 2022 | [10.3390/diagnostics12123045](https://doi.org/10.3390/diagnostics12123045) | No | Yes | Yes | Yes | Yes | No | Yes | Yes | Yes | No | No | Yes | 8 |
| Eichberg D et al. 2018 | [10.1080/02688697.2018.1490943](https://doi.org/10.1080/02688697.2018.1490943) | No | Yes | Yes | Yes | No | No | Yes | Yes | Yes | Yes | Yes | No | 8 |
| Ellens N.R. et al., 2019 | [0.1093/neuros/nyy090](https://doi.org/10.1093/neuros/nyy090) | Yes | Yes | Yes | Yes | No | No | Yes | Yes | Yes | Yes | Yes | Yes | 9 |
| Elliott RE et al., 2013 | [10.3171/2013.4.JNS121829](https://doi.org/10.3171/2013.4.jns121829) | No | Yes | Yes | Yes | No | No | Yes | Yes | Yes | Yes | Yes | Yes | 9 |
| Elsamadicy AA et al., 2018 | [10.1016/j.jocn.2017.09.021](https://doi.org/10.1016/j.jocn.2017.09.021) | No | Yes | Yes | Yes | No | No | Yes | Yes | Yes | Yes | Yes | Yes | 9 |
| Elward A et al. 2015 | [10.1097/INF.0000000000000889](https://doi.org/10.1097/inf.0000000000000889) | Yes | Yes | Yes | Yes | No | No | Yes | Yes | Yes | Yes | Yes | Yes | 9 |
| Engelhardt M et al. 2005 | [10.1055/s-2005-836476](https://doi.org/10.1055/s-2005-836476) | No | Yes | Yes | Yes | No | No | Yes | Yes | Yes | Yes | Yes | Yes | 9 |
| Englot D.J. et al., 2014 | [10.3171/2014.7.PEDS13658](https://doi.org/10.3171/2014.7.peds13658) | No | Yes | Yes | Yes | No | No | Yes | Yes | Yes | Yes | No | Yes | 8 |
| Ening G et al. 2015 | [10.1016/j.clineuro.2015.01.006](https://doi.org/10.1016/j.clineuro.2015.01.006) | No | Yes | Yes | Yes | Yes | Yes | Yes | Yes | Yes | Yes | Yes | Yes | 11 |
| Estes EM et al., 2023 | [10.1016/j.clineuro.2023.107864](https://doi.org/10.1016/j.clineuro.2023.107864) | No | Yes | Yes | Yes | Yes | No | Yes | Yes | Yes | Yes | Yes | Yes | 10 |
| Falowski S et al., 2012 | [10.1159/000338254](https://doi.org/10.1159/000338254) | No | Yes | Yes | Yes | No | No | Yes | Yes | Yes | Yes | Yes | Yes | 9 |
| Falowski S M et al. 2015 | [10.1016/j.wneu.2015.01.018](https://doi.org/10.1016/j.wneu.2015.01.018) | No | Yes | Yes | Yes | No | No | Yes | Yes | Yes | Yes | Yes | Yes | 9 |
| Fan MC et al. , 2018 | [10.1016/j.wneu.2017.10.112](https://doi.org/10.1016/j.wneu.2017.10.112) | Yes | Yes | Yes | Yes | No | No | Yes | Yes | Yes | Yes | Yes | Yes | 9 |
| Farber S.H. et al., 2011 | [10.1227/NEU.0b013e31821bc435](https://doi.org/10.1227/neu.0b013e31821bc435) | Yes | Yes | Yes | Yes | No | No | Yes | Yes | Yes | Yes | Yes | Yes | 9 |
| Farber SH et al., 2010 | [10.1016/j.wneu.2010.07.014](https://doi.org/10.1016/j.wneu.2010.07.014) | No | Yes | Yes | Yes | Yes | No | Yes | Yes | Yes | Yes | Yes | Yes | 10 |
| Farrokhi F R et al. 2019 | [10.1016/j.jocn.2019.08.026](https://doi.org/10.1016/j.jocn.2019.08.026) | No | Yes | Yes | Yes | No | No | Yes | Yes | Yes | Yes | Yes | Yes | 9 |
| Fattahi A et al. 2018 | [10.1080/02688697.2018.1476673](https://doi.org/10.1080/02688697.2018.1476673) | No | Yes | Yes | Yes | No | No | Yes | Yes | Yes | Yes | Yes | No | 8 |
| Fenoy A J et al. 2012 | [10.3171/2012.1.JNS111798](https://doi.org/10.3171/2012.1.jns111798) | Yes | Yes | Yes | Yes | No | No | Yes | Yes | Yes | Yes | Yes | Yes | 9 |
| Fenoy A J et al. 2014 | [10.3171/2013.10.JNS131225](https://doi.org/10.3171/2013.10.jns131225) | No | Yes | Yes | Yes | No | No | Yes | Yes | Yes | No | No | Yes | 7 |
| Fernandez C.S. et al., 2022 | [10.5603/PJNNS.a2022.0030](https://doi.org/10.5603/pjnns.a2022.0030) | No | Yes | Yes | Yes | No | No | Yes | Yes | Yes | Yes | Yes | Yes | 9 |
| Ferreira de Andrade A et al. 2020 | PMCID: PMC7364413 | No | Yes | Yes | Yes | No | No | Yes | Yes | Yes | Yes | Yes | Yes | 9 |
| Fialkov J A et al. 2001 | [10.1097/00001665-200107000-00009](https://doi.org/10.1097/00001665-200107000-00009) | Yes | Yes | Yes | Yes | No | No | Yes | Yes | Yes | Yes | Yes | Yes | 9 |
| Filho NO et al., 2016 | [10.3171/2015.9.PEDS1559](https://doi.org/10.3171/2015.9.peds1559) | No | Yes | Yes | Yes | No | No | Yes | Yes | Yes | Yes | Yes | Yes | 9 |
| Findlay MC et al., 2023 | [10.1227/neu.0000000000002397](https://doi.org/10.1227/neu.0000000000002397) | No | Yes | Yes | Yes | Yes | Yes | Yes | Yes | Yes | Yes | Yes | Yes | 11 |
| Flanagan LS et al., 2022 | [10.1002/lary.29893](https://doi.org/10.1002/lary.29893) | No | Yes | Yes | Yes | Yes | Yes | Yes | Yes | Yes | Yes | Yes | Yes | 11 |
| Forcadas-Berdusan M et al. 2011 | [10.1684/epd.2011.0413](https://doi.org/10.1684/epd.2011.0413) | No | Yes | Yes | Yes | No | No | Yes | Yes | Yes | Yes | Yes | Yes | 9 |
| Foster KA et al., 2017 | [10.1016/j.wneu.2016.02.071](https://doi.org/10.1016/j.wneu.2016.02.071) | No | Yes | Yes | Yes | Yes | Yes | Yes | Yes | Yes | Yes | Yes | Yes | 11 |
| Foster M et al. 2021 | [10.3171/2020.9.PEDS20556](https://doi.org/10.3171/2020.9.peds20556) | No | Yes | Yes | Yes | No | No | Yes | Yes | Yes | Yes | Yes | Yes | 9 |
| Frizon LA et al. 2017 | [10.1111/ner.12605](https://doi.org/10.1111/ner.12605) | Yes | Yes | Yes | Yes | No | No | Yes | Yes | Yes | Yes | Yes | Yes | 9 |
| Fuentes A.M. et al., 2021 | [10.1016/j.clineuro.2021.106757](https://doi.org/10.1016/j.clineuro.2021.106757) | No | Yes | Yes | Yes | No | No | Yes | Yes | Yes | Yes | Yes | Yes | 9 |
| Fujimoto Y et al, 2008 | [10.1016/j.surneu.2007.06.091](https://doi.org/10.1016/j.surneu.2007.06.091) | No | Yes | Yes | Yes | No | No | Yes | Yes | Yes | Yes | Yes | Yes | 9 |
| Gadgil N. Et al., 2018 | [10.1097/SCS.0000000000004654](https://doi.org/10.1097/scs.0000000000004654) | No | Yes | Yes | Yes | No | No | Yes | Yes | Yes | Yes | Yes | Yes | 9 |
| Garzon-Muvdi T et al. 2015 | [10.1227/NEU.0000000000000625](https://doi.org/10.1227/neu.0000000000000625) | No | Yes | Yes | Yes | No | No | Yes | Yes | Yes | No | No | Yes | 7 |
| Gazzeri R et al., 2020 | [10.1016/j.clineuro.2020.105705](https://doi.org/10.1016/j.clineuro.2020.105705) | No | Yes | Yes | Yes | No | No | Yes | Yes | Yes | Yes | Yes | Yes | 9 |
| George B et al. 2017 | [10.1093/neuros/nyx024](https://doi.org/10.1093/neuros/nyx024) | No | Yes | Yes | Yes | No | No | Yes | Yes | Yes | Yes | Yes | Yes | 9 |
| Gil Z et al. 2003 | [10.1067/mhn.2003.14](https://doi.org/10.1067/mhn.2003.14) | Yes | Yes | Yes | Yes | No | No | Yes | Yes | Yes | Yes | Yes | Yes | 9 |
| Giovanni S. Et al., 2014 | [10.1016/j.clineuro.2014.05.005](https://doi.org/10.1016/j.clineuro.2014.05.005) | No | Yes | Yes | Yes | No | No | Yes | Yes | Yes | Yes | Yes | Yes | 9 |
| Girgis F et al. 2015 | [10.1017/cjn.2015.46](https://doi.org/10.1017/cjn.2015.46) | Yes | Yes | Yes | Yes | No | No | Yes | Yes | Yes | Yes | Yes | Yes | 9 |
| Goel NJ et al., 2018 | [10.1016/j.wneu.2018.06.153](https://doi.org/10.1016/j.wneu.2018.06.153) | No | Yes | Yes | Yes | Yes | Yes | Yes | Yes | Yes | Yes | Yes | Yes | 11 |
| Goldschlager T et al., 2007 | [10.1016/j.jocn.2006.12.002](https://doi.org/10.1016/j.jocn.2006.12.002) | No | Yes | Yes | Yes | No | No | Yes | Yes | Yes | Yes | No | Yes | 8 |
| Golebiowski A. Et al., 2015 | [10.1007/s00701-014-2286-3](https://doi.org/10.1007/s00701-014-2286-3) | No | Yes | Yes | Yes | Yes | Yes | Yes | Yes | Yes | Yes | Yes | Yes | 11 |
| Gonzalez-Vargas P.M. et al., 2020 | [10.1016/j.inat.2019.100606](https://doi.org/10.1016/j.inat.2019.100606) | No | Yes | Yes | Yes | No | No | Yes | Yes | Yes | Yes | Yes | Yes | 9 |
| Gorgulho A. Et al., 2009 | [10.3171/2008.6.17603](https://doi.org/10.3171/2008.6.17603) | Yes | Yes | Yes | Yes | No | No | Yes | Yes | Yes | Yes | Yes | Yes | 9 |
| Goshtasbi K et al. 2020 | [10.1016/j.clineuro.2020.106192](https://doi.org/10.1016/j.clineuro.2020.106192) | No | Yes | Yes | Yes | Yes | No | Yes | Yes | Yes | Yes | Yes | Yes | 10 |
| Gottsche J et al. 2019 | [10.1055/s-0039-1698391](https://doi.org/10.1055/s-0039-1698391) | No | Yes | Yes | Yes | No | No | Yes | Yes | Yes | Yes | Yes | Yes | 9 |
| Govindaswamy A et al. 2022 | [10.4103/ajns.AJNS_268_18](https://doi.org/10.4103/ajns.ajns_268_18) | Yes | Yes | Yes | Yes | No | No | Yes | Yes | Yes | Yes | Yes | Yes | 9 |
| Goyal-Honavar A et al. 2022 | [10.1016/j.jocn.2022.06.024](https://doi.org/10.1016/j.jocn.2022.06.024) | No | Yes | Yes | Yes | No | No | Yes | Yes | Yes | Yes | Yes | Yes | 9 |
| Grau S et al. 2018 | [10.4103/joacp.JOACP_373_16](https://doi.org/10.4103/joacp.joacp_373_16) | No | Yes | Yes | Yes | No | No | Yes | Yes | Yes | Yes | Yes | Yes | 9 |
| Greenberg JK et al., 2016 | [10.3171/2015.10.PEDS15369](https://doi.org/10.3171/2015.10.peds15369) | No | Yes | Yes | Yes | Yes | No | Yes | Yes | Yes | Yes | Yes | Yes | 10 |
| Grossman R et al. 2013 | [10.1245/s10434-012-2748-x](https://doi.org/10.1245/s10434-012-2748-x) | No | Yes | Yes | Yes | No | No | Yes | Yes | Yes | Yes | Yes | Yes | 9 |
| Gruenbaum S. Et al., 2017 | [10.1213/ANE.0000000000001946](https://doi.org/10.1213/ane.0000000000001946) | Yes | Yes | Yes | Yes | Yes | Yes | Yes | Yes | Yes | Yes | Yes | Yes | 11 |
| Grundy T et al. 2019 | [10.1080/02688697.2019.1645298](https://doi.org/10.1080/02688697.2019.1645298) | Yes | Yes | Yes | Yes | Yes | Yes | Yes | Yes | Yes | No | No | Yes | 9 |
| Guidry BS et al. 2022 | [10.1227/neu.0000000000002053](https://doi.org/10.1227/neu.0000000000002053) | No | Yes | Yes | Yes | No | No | Yes | Yes | Yes | Yes | Yes | Yes | 9 |
| Gupta A. Et al., 2018 | [10.1016/j.wneu.2018.07.200](https://doi.org/10.1016/j.wneu.2018.07.200) | No | Yes | Yes | Yes | No | No | Yes | Yes | Yes | Yes | Yes | Yes | 9 |
| Gupta S et al. 2019 | [10.1016/j.wneu.2018.09.081](https://doi.org/10.1016/j.wneu.2018.09.081) | No | Yes | Yes | Yes | No | No | Yes | Yes | Yes | Yes | Yes | Yes | 9 |
| Gupta S et al. 2021 | [10.3389/fonc.2021.662943](https://doi.org/10.3389/fonc.2021.662943) | No | Yes | Yes | Yes | Yes | No | Yes | Yes | Yes | Yes | Yes | Yes | 10 |
| Guyolla Y H et al. 2022 | [10.1016/j.inat.2022.101704](https://doi.org/10.1016/j.inat.2022.101704) | No | Yes | Yes | Yes | No | No | Yes | Yes | Yes | Yes | Yes | Yes | 9 |
| Hale A.T. et al., 2020 | [10.3171/2019.9.PEDS1939](https://doi.org/10.3171/2019.9.peds1939) | Yes | Yes | Yes | Yes | Yes | Yes | Yes | Yes | Yes | Yes | Yes | Yes | 11 |
| Halpern C H et al. 2012 | [10.1016/j.ajic.2011.06.005](https://doi.org/10.1016/j.ajic.2011.06.005) | Yes | Yes | Yes | Yes | No | No | Yes | Yes | Yes | Yes | Yes | Yes | 9 |
| Hamdeh S A et al. 2014 | [10.3109/02688697.2013.835376](https://doi.org/10.3109/02688697.2013.835376) | Yes | Yes | Yes | Yes | No | No | Yes | Yes | Yes | Yes | Yes | Yes | 9 |
| Hammond C.J. et al., 2002 | PMID: 12617235 | No | Yes | Yes | Yes | No | No | Yes | Yes | Yes | Yes | Yes | Yes | 9 |
| Han RH et al., 2016 | [10.3171/2015.7.PEDS15187](https://doi.org/10.3171/2015.7.peds15187) | No | Yes | Yes | Yes | No | No | Yes | Yes | Yes | Yes | Yes | Yes | 9 |
| Hardaway F.A. et al., 2017 | [10.1093/neuros/nyx505](https://doi.org/10.1093/neuros/nyx505) | Yes | Yes | Yes | Yes | No | No | Yes | Yes | Yes | No | No | Yes | 7 |
| Hardesty D A et al. 2021 | [10.3171/2020.8.JNS202404](https://doi.org/10.3171/2020.8.jns202404) | No | Yes | Yes | Yes | Yes | Yes | Yes | Yes | Yes | Yes | No | Yes | 10 |
| Hardy S et al. 2010 | [10.3171/2010.2.JNS09950](https://doi.org/10.3171/2010.2.jns09950) | Yes | Yes | Yes | Yes | Yes | Yes | Yes | Yes | Yes | No | No | Yes | 9 |
| Harland T et al. 2023 | [10.1227/ons.0000000000000698](https://doi.org/10.1227/ons.0000000000000698) | No | Yes | Yes | Yes | Yes | No | Yes | Yes | Yes | Yes | Yes | Yes | 10 |
| Harrop J.S. et al., 2010 | [10.1227/01.NEU.0000370247.11479.B6](https://doi.org/10.1227/01.neu.0000370247.11479.b6) | Yes | Yes | Yes | Yes | Yes | No | Yes | Yes | Yes | Yes | Yes | Yes | 10 |
| Hasegawa H et al. 2021 | [10.3171/2020.7.JNS201385](https://doi.org/10.3171/2020.7.jns201385) | Yes | Yes | Yes | Yes | Yes | No | Yes | Yes | Yes | Yes | No | Yes | 9 |
| Hayashi T. Et al., 2010 | [10.3171/2010.5.PEDS1018](https://doi.org/10.3171/2010.5.peds1018) | Yes | Yes | Yes | Yes | No | No | Yes | Yes | Yes | No | No | Yes | 7 |
| He J et al., 2023 | [10.3389/fneur.2023.1153392](https://doi.org/10.3389/fneur.2023.1153392) | No | Yes | Yes | Yes | No | No | Yes | Yes | Yes | Yes | No | Yes | 8 |
| Helal A et al. 2018 | [10.3171/2018.7.FOCUS18258](https://doi.org/10.3171/2018.7.focus18258) | No | Yes | Yes | Yes | Yes | No | Yes | Yes | Yes | No | No | Yes | 8 |
| Helmers AK et al., 2018 | [10.1016/j.wneu.2018.01.183](https://doi.org/10.1016/j.wneu.2018.01.183) | No | Yes | Yes | Yes | Yes | No | Yes | Yes | Yes | Yes | No | Yes | 9 |
| Henderson D et al. 2022 | [10.25259/SNI_103_2022](https://doi.org/10.25259/sni_103_2022) | No | Yes | Yes | Yes | Yes | No | Yes | Yes | Yes | No | No | Yes | 8 |
| Henry R.K. et al., 2021 | [10.1002/lary.29485](https://doi.org/10.1002/lary.29485) | No | Yes | Yes | Yes | Yes | Yes | Yes | Yes | Yes | No | No | Yes | 9 |
| Hill T et al. 2017 | [10.1016/j.wneu.2017.01.093](https://doi.org/10.1016/j.wneu.2017.01.093) | No | Yes | Yes | Yes | Yes | No | Yes | Yes | Yes | No | No | Yes | 8 |
| Hirsch LJ et al. 2020 | [10.1111/epi.16442](https://doi.org/10.1111/epi.16442) | No | Yes | Yes | Yes | Yes | No | Yes | Yes | Yes | No | No | Yes | 8 |
| Hng D. Et al., 2014 | [10.1055/s-0034-1395383](https://doi.org/10.1055/s-0034-1395383) | No | Yes | Yes | Yes | Yes | Yes | Yes | Yes | Yes | No | No | Yes | 9 |
| Ho A et al. 2018 | [10.3171/2018.5.PEDS17719](https://doi.org/10.3171/2018.5.peds17719) | Yes | Yes | Yes | Yes | Yes | Yes | Yes | Yes | Yes | No | No | Yes | 9 |
| Hoang T et al. 2023 | [10.1017/ice.2022.112](https://doi.org/10.1017/ice.2022.112) | Yes | Yes | Yes | Yes | Yes | No | Yes | Yes | Yes | No | No | Yes | 8 |
| Hoffman H et al. 2019 | [10.1016/j.wneu.2019.05.021](https://doi.org/10.1016/j.wneu.2019.05.021) | No | No | Yes | Yes | Yes | No | Yes | Yes | Yes | No | No | Yes | 7 |
| Honeybul S et al., 2016 | [10.1080/02688697.2016.1187259](https://doi.org/10.1080/02688697.2016.1187259) | No | Yes | Yes | Yes | Yes | No | Yes | Yes | Yes | No | No | Yes | 8 |
| Honeybul S. Et al., 2012 | [10.1097/PRS.0b013e318267d4de](https://doi.org/10.1097/prs.0b013e318267d4de) | Yes | Yes | No | Yes | Yes | No | Yes | Yes | Yes | No | No | Yes | 7 |
| Hoover J M et al. 2013 | [10.3171/2013.2.JNS121731](https://doi.org/10.3171/2013.2.jns121731) | No | Yes | No | Yes | Yes | No | Yes | Yes | Yes | No | No | Yes | 7 |
| Horisawa S et al. 2019 | [10.1212/WNL.0000000000006818](https://doi.org/10.1212/wnl.0000000000006818) | No | Yes | Yes | Yes | Yes | Yes | Yes | Yes | Yes | No | No | Yes | 9 |
| Hu H et al., 2022 | [10.1016/j.jgar.2023.08.006](https://doi.org/10.1016/j.jgar.2023.08.006) | Yes | Yes | Yes | Yes | Yes | Yes | Yes | Yes | Yes | No | No | Yes | 9 |
| Huang Y et al. 2011 | [10.1016/j.injury.2011.11.005](https://doi.org/10.1016/j.injury.2011.11.005) | Yes | Yes | Yes | Yes | Yes | Yes | Yes | Yes | Yes | No | No | Yes | 9 |
| Huang Y.H. et al., 2013 | [10.1016/j.ijsu.2013.07.013](https://doi.org/10.1016/j.ijsu.2013.07.013) | No | Yes | Yes | Yes | Yes | Yes | Yes | Yes | Yes | No | No | Yes | 9 |
| Huang YH et al. 2011 | [10.1097/TA.0b013e318203208a](https://doi.org/10.1097/ta.0b013e318203208a) | Yes | Yes | Yes | Yes | Yes | Yes | Yes | Yes | Yes | No | No | Yes | 9 |
| Hutchinson P.J et al. 2020 | [10.1056/nejmoa2020473](https://doi.org/10.1056/nejmoa2020473) | No | No | No | Yes | Yes | Yes | Yes | Yes | Yes | No | No | Yes | 7 |
| Hutter G et al. 2014 | 10.3171/2014.6.JNS131917 | No | Yes | Yes | Yes | Yes | Yes | Yes | Yes | Yes | No | No | Yes | 9 |
| Im S.H. et al., 2012 | [10.3340/jkns.2012.52.4.396](https://doi.org/10.3340/jkns.2012.52.4.396) | Yes | Yes | No | Yes | Yes | No | Yes | Yes | Yes | No | No | Yes | 7 |
| Inoue T et al. 2020 | [10.1007/s00701-020-04242-8](https://doi.org/10.1007/s00701-020-04242-8) | No | Yes | No | Yes | Yes | No | Yes | Yes | Yes | No | No | Yes | 7 |
| Isaac K.V. et al., 2018 | [10.3171/2018.5.PEDS1846](https://doi.org/10.3171/2018.5.peds1846) | No | Yes | Yes | Yes | Yes | No | Yes | Yes | Yes | No | No | Yes | 8 |
| Isobe N. Et al., 2018 | [10.1016/j.wneu.2018.07.080](https://doi.org/10.1016/j.wneu.2018.07.080) | No | Yes | Yes | Yes | Yes | No | Yes | Yes | Yes | No | No | Yes | 8 |
| Janjua M et al. 2020 | [10.3171/2019.7.PEDS19272](https://doi.org/10.3171/2019.7.peds19272) | No | No | No | Yes | Yes | No | Yes | Yes | Yes | No | No | Yes | 6 |
| Jeong T S et al. 2018 | [10.3340/jkns.2018.0021](https://doi.org/10.3340/jkns.2018.0021) | Yes | No | No | Yes | Yes | No | Yes | Yes | Yes | No | No | Yes | 6 |
| Jeong T.S. et al., 2020 | [10.1371/journal.pone.0232561](https://doi.org/10.1371/journal.pone.0232561) | Yes | Yes | Yes | Yes | Yes | No | Yes | Yes | Yes | Yes | No | Yes | 9 |
| Jia C et al., 2019 | [10.1016/j.clineuro.2018.11.008](https://doi.org/10.1016/j.clineuro.2018.11.008) | No | No | No | Yes | Yes | Yes | Yes | Yes | Yes | No | No | Yes | 7 |
| Jiang X et al. 2014 | [10.5137/1019-5149.JTN.12738-14.0](https://doi.org/10.5137/1019-5149.jtn.12738-14.0) | Yes | Yes | Yes | Yes | Yes | No | Yes | Yes | Yes | Yes | No | Yes | 9 |
| Jimenez A et al. 2021 | [10.1016/j.wneu.2021.12.010](https://doi.org/10.1016/j.wneu.2021.12.010) | No | No | No | Yes | Yes | No | Yes | Yes | Yes | No | No | Yes | 6 |
| Jimenez-Martinez E et al. 2019 | [10.1186/s13756-019-0525-3](https://doi.org/10.1186/s13756-019-0525-3) | Yes | No | No | Yes | Yes | Yes | Yes | Yes | Yes | No | No | Yes | 7 |
| Jimenez-Martinez E et al. 2021 | [10.1093/cid/ciaa884](https://doi.org/10.1093/cid/ciaa884) | Yes | No | No | Yes | Yes | No | Yes | Yes | Yes | No | No | Yes | 6 |
| Jimenez-Martinez E. Et al., 2021 | [10.1186/s13756-021-01016-4](https://doi.org/10.1186/s13756-021-01016-4) | Yes | Yes | Yes | Yes | Yes | No | Yes | Yes | Yes | No | No | Yes | 8 |
| Joerger A et al. 2023 | [10.1007/s00701-023-05870-6](https://doi.org/10.1007/s00701-023-05870-6) | Yes | Yes | Yes | Yes | Yes | No | Yes | Yes | Yes | No | No | Yes | 8 |
| Jorger A et al. 2018 | [10.1007/s00701-018-3704-8](https://doi.org/10.1007/s00701-018-3704-8) | No | Yes | Yes | Yes | No | No | Yes | Yes | Yes | No | No | Yes | 7 |
| Joswig H et al., 2016 | [10.1016/j.wneu.2016.03.081](https://doi.org/10.1016/j.wneu.2016.03.081) | No | Yes | Yes | Yes | Yes | Yes | Yes | Yes | Yes | No | No | Yes | 9 |
| Kaestner S et al., 2017 | [10.1016/j.pjnns.2016.11.007](https://doi.org/10.1016/j.pjnns.2016.11.007) | No | Yes | Yes | Yes | No | No | Yes | Yes | Yes | No | No | Yes | 7 |
| Kalangu K et al. 2020 | [10.1007/s00381-019-04357-z](https://doi.org/10.1007/s00381-019-04357-z) | Yes | Yes | Yes | Yes | Yes | No | Yes | Yes | Yes | No | No | Yes | 8 |
| Kalani M.Y.S. et al., 2015 | [10.1016/j.wneu.2014.10.013](https://doi.org/10.1016/j.wneu.2014.10.013) | No | Yes | Yes | Yes | No | No | Yes | Yes | Yes | No | No | No | 6 |
| Kamenova M et al., 2017 | [10.1016/j.wneu.2017.01.065](https://doi.org/10.1016/j.wneu.2017.01.065) | No | Yes | Yes | Yes | No | No | Yes | Yes | Yes | No | No | Yes | 7 |
| Karabacak M et al. 2024 | [10.1089/neu.2023.0122](https://doi.org/10.1089/neu.2023.0122) | No | Yes | Yes | Yes | Yes | No | Yes | Yes | Yes | Yes | No | Yes | 9 |
| Kassicieh A et al. 2022 | [10.1016/j.clineuro.2022.107383](https://doi.org/10.1016/j.clineuro.2022.107383) | No | Yes | Yes | Yes | Yes | Yes | Yes | Yes | Yes | Yes | Yes | Yes | 11 |
| Kerezoudis P et al. 2018 | [10.3171/2016.12.JNS162096](https://doi.org/10.3171/2016.12.jns162096) | No | Yes | Yes | Yes | Yes | Yes | Yes | Yes | Yes | Yes | Yes | Yes | 11 |
| Kerttula S et al. 2022 | [10.1007/s00701-022-05337-0](https://doi.org/10.1007/s00701-022-05337-0) | No | Yes | Yes | Yes | Yes | Yes | Yes | Yes | Yes | Yes | Yes | Yes | 11 |
| Khan M P et al. 2021 | 10.53350/pjmhs211592770 | No | Yes | Yes | Yes | No | No | Yes | Yes | Yes | No | No | Yes | 7 |
| Kilgore C.B. et al., 2023 | [10.1016/j.wneu.2023.10.009](https://doi.org/10.1016/j.wneu.2023.10.009) | No | Yes | Yes | Yes | Yes | Yes | Yes | Yes | Yes | No | No | Yes | 9 |
| Kim K H et al. 2020 | [10.1016/j.wneu.2020.05.154](https://doi.org/10.1016/j.wneu.2020.05.154) | No | Yes | Yes | Yes | Yes | Yes | Yes | Yes | Yes | No | No | Yes | 9 |
| Kim M J et al. 2021 | [10.3389/fneur.2021.745575](https://doi.org/10.3389/fneur.2021.745575) | Yes | Yes | Yes | Yes | Yes | Yes | Yes | Yes | Yes | No | No | Yes | 9 |
| Kim SP et al., 2014 | [10.3340/jkns.2014.56.5.410](https://doi.org/10.3340/jkns.2014.56.5.410) | No | Yes | Yes | Yes | Yes | Yes | Yes | Yes | Yes | No | No | Yes | 9 |
| Kim T et al. 2013 | [10.1007/s00701-013-1833-7](https://doi.org/10.1007/s00701-013-1833-7) | Yes | Yes | Yes | Yes | Yes | Yes | Yes | Yes | Yes | No | No | Yes | 9 |
| Kimmell KT et al., 2015 | [10.3171/2014.10.JNS14632](https://doi.org/10.3171/2014.10.jns14632) | No | Yes | Yes | Yes | Yes | Yes | Yes | Yes | Yes | No | No | Yes | 9 |
| Kinaci A et al. 2023 | [10.1227/neu.0000000000002345](https://doi.org/10.1227/neu.0000000000002345) | Yes | Yes | Yes | Yes | Yes | No | Yes | Yes | Yes | Yes | No | Yes | 9 |
| Klekamp J. 2012 | [10.1227/NEU.0b013e31825c3426](https://doi.org/10.1227/neu.0b013e31825c3426) | No | Yes | Yes | Yes | Yes | No | Yes | Yes | Yes | No | No | Yes | 8 |
| Klieverik V.M. et al., 2023 | [10.1016/j.wneu.2023.04.008](https://doi.org/10.1016/j.wneu.2023.04.008) | Yes | Yes | Yes | Yes | No | No | Yes | Yes | Yes | No | No | Yes | 7 |
| Koch J. Et al., 2023 | [10.1227/neu.0000000000002507](https://doi.org/10.1227/neu.0000000000002507) | Yes | Yes | Yes | Yes | Yes | Yes | Yes | Yes | Yes | No | No | Yes | 9 |
| Kochanski R et al. 2018 | 10.1093/ons/opx293 | Yes | Yes | Yes | Yes | Yes | Yes | Yes | Yes | Yes | No | No | Yes | 9 |
| Kogeichi Y. Et al., 2022 | [10.1016/j.inat.2022.101489](https://doi.org/10.1016/j.inat.2022.101489) | Yes | Yes | Yes | Yes | Yes | Yes | Yes | Yes | Yes | No | No | Yes | 9 |
| Koipapi S et al. 2023 | [10.1016/j.wnsx.2023.100257](https://doi.org/10.1016/j.wnsx.2023.100257) | No | Yes | Yes | Yes | Yes | No | Yes | Yes | Yes | Yes | No | Yes | 9 |
| Kolpa M. Et al., 2019 | [10.3390/ijerph16060911](https://doi.org/10.3390/ijerph16060911) | No | Yes | Yes | Yes | Yes | No | Yes | Yes | Yes | No | No | Yes | 8 |
| Kombogiorgas D et al. 2006 | [10.1007/s00381-006-0117-4](https://doi.org/10.1007/s00381-006-0117-4) | No | Yes | Yes | Yes | Yes | Yes | Yes | Yes | Yes | No | No | Yes | 9 |
| Kondapavulur S et al. 2022 | [10.1159/000520197](https://doi.org/10.1159/000520197) | Yes | Yes | Yes | Yes | Yes | Yes | Yes | Yes | Yes | No | No | Yes | 9 |
| Korhonen T K et al. 2019 | [10.3171/2017.12.JNS172013](https://doi.org/10.3171/2017.12.jns172013) | No | Yes | Yes | Yes | Yes | Yes | Yes | Yes | Yes | No | No | Yes | 9 |
| Korinek AM et al. 2005 | [10.1080/02688690500145639](https://doi.org/10.1080/02688690500145639) | Yes | Yes | Yes | Yes | Yes | No | Yes | Yes | Yes | No | No | Yes | 8 |
| Korinek AM et al. 2006 | [10.1227/01.neu.0000316256.44349.b1](https://doi.org/10.1227/01.neu.0000316256.44349.b1) | No | Yes | Yes | Yes | Yes | Yes | Yes | Yes | Yes | No | No | Yes | 9 |
| Kose G et al. 2015 | [10.1111/jocn.13149](https://doi.org/10.1111/jocn.13149) | Yes | Yes | Yes | Yes | Yes | Yes | Yes | Yes | Yes | No | No | Yes | 9 |
| Koskinen LD et al. 2013 | [10.1007/s00701-013-1856-0](https://doi.org/10.1007/s00701-013-1856-0) | No | Yes | Yes | Yes | Yes | Yes | Yes | Yes | Yes | Yes | No | Yes | 10 |
| Kothari S.N. et al., 2017 | 10.1016/j.jamcollsurg.2017.07.574 | No | Yes | Yes | Yes | Yes | Yes | Yes | Yes | Yes | No | No | Yes | 9 |
| Kourbeti I et al, 2012 | 10.1111/j.1469-0691.2011.03625.x | No | Yes | Yes | Yes | Yes | No | Yes | Yes | Yes | No | Yes | Yes | 9 |
| Kourbeti I. Et al., 2015 | 10.3171/2014.8.JNS132557 | No | Yes | Yes | Yes | Yes | No | Yes | Yes | Yes | Yes | No | Yes | 9 |
| Krafft P et al. 2022 | 10.1016/j.clineuro.2022.107206 | Yes | Yes | Yes | Yes | Yes | Yes | Yes | Yes | Yes | No | No | Yes | 9 |
| Kraus D et al. 2005 | 10.1097/01.mlg.0000172201.61487.69 | Yes | Yes | Yes | Yes | Yes | No | Yes | Yes | Yes | No | No | Yes | 8 |
| Kretschmer T et al. 2009 | 10.1080/026886900417342 | No | Yes | Yes | Yes | No | No | Yes | Yes | Yes | No | No | No | 6 |
| Krishnan SS et al., 2020 | 10.1055/s-0039-1678602 | No | Yes | Yes | Yes | Yes | No | Yes | Yes | Yes | No | No | Yes | 8 |
| Kumar S et al., 2022 | 10.4103/0028-3886.344635 | No | Yes | Yes | Yes | Yes | Yes | Yes | Yes | Yes | No | No | Yes | 9 |
| Kuwano A et al. 2023 | 10.1007/s00701-022-05474-6 | Yes | Yes | Yes | Yes | Yes | Yes | Yes | Yes | Yes | No | No | Yes | 9 |
| Lam FC et al., 2012 | [10.1227/NEU.0b013e31826a8ab0](https://doi.org/10.1227/neu.0b013e31826a8ab0) | No | Yes | Yes | Yes | Yes | No | Yes | Yes | Yes | No | No | Yes | 8 |
| Lan M et al. 2019 | 10.1016/j.wneu.2019.09.148 | No | Yes | Yes | Yes | Yes | Yes | Yes | Yes | Yes | No | No | Yes | 9 |
| Langness S. Et al., 2017 | [10.1016/j.amjsurg.2016.06.001](https://doi.org/10.1016/j.amjsurg.2016.06.001) | No | Yes | Yes | Yes | Yes | No | Yes | Yes | Yes | Yes | No | Yes | 8 |
| Lannon M et al. 2022 | [10.1097/TA.0000000000003385](https://doi.org/10.1097/ta.0000000000003385) | No | Yes | Yes | Yes | Yes | Yes | Yes | Yes | Yes | Yes | No | Yes | 10 |
| Lawrence J et al. 2016 | [10.3171/2016.5.JNS152118](https://doi.org/10.3171/2016.5.jns152118) | No | Yes | Yes | Yes | Yes | Yes | Yes | Yes | Yes | No | No | Yes | 9 |
| Le Guen M. Et al., 2023 | [10.1186/s12871-022-01962-5](https://doi.org/10.1186/s12871-022-01962-5) | No | Yes | Yes | Yes | Yes | Yes | Yes | Yes | Yes | No | No | Yes | 9 |
| Lee CH et al., 2012 | [10.1097/TA.0b013e318256a150](https://doi.org/10.1097/ta.0b013e318256a150) | Yes | Yes | Yes | Yes | Yes | Yes | Yes | Yes | Yes | No | No | Yes | 9 |
| Lee J.K. et al., 2012 | [10.3346/jkms.2012.27.12.1563](https://doi.org/10.3346/jkms.2012.27.12.1563) | Yes | Yes | Yes | Yes | Yes | Yes | Yes | Yes | Yes | No | No | Yes | 9 |
| Lee JA et al., 2022 | [10.1016/j.wneu.2022.07.013](https://doi.org/10.1016/j.wneu.2022.07.013) | No | Yes | Yes | Yes | Yes | Yes | Yes | Yes | Yes | No | No | Yes | 9 |
| Lee MH et al. 2015 | [10.1007/s10143-015-0666-7](https://doi.org/10.1007/s10143-015-0666-7) | No | Yes | Yes | Yes | Yes | No | Yes | Yes | Yes | Yes | No | Yes | 9 |
| Lee S et al. 2019 | 10.1016/j.wneu.2019.06.155 | No | Yes | Yes | Yes | Yes | No | Yes | Yes | Yes | Yes | No | No | 8 |
| Lefebvre J et al. 2017 | [10.1016/j.jhin.2016.11.019](https://doi.org/10.1016/j.jhin.2016.11.019) | Yes | Yes | Yes | Yes | Yes | No | Yes | Yes | Yes | Yes | No | Yes | 9 |
| Lemee J.-M. Et al., 2019 | 10.1016/j.wneu.2019.05.010 | No | Yes | Yes | Yes | Yes | Yes | Yes | Yes | Yes | Yes | No | Yes | 10 |
| Lepanluoma M et al., 2015 | 10.3171/2014.12.JNS141077 | No | Yes | Yes | Yes | Yes | Yes | Yes | Yes | Yes | Yes | No | Yes | 10 |
| Lepski G et al. 2021 | 10.1016/j.clineuro.2021.106599 | Yes | Yes | Yes | Yes | Yes | No | Yes | Yes | Yes | Yes | No | Yes | 9 |
| Leung G.K. et al., 2007 | 10.1080/02688690701392881 | Yes | Yes | Yes | Yes | Yes | No | Yes | Yes | Yes | Yes | No | Yes | 9 |
| Levi V et al., 2020 | 10.1093/ons/opz118 | Yes | Yes | Yes | Yes | Yes | Yes | Yes | Yes | Yes | Yes | No | Yes | 10 |
| Lewis A et al., 2017 | 10.3171/2016.4.JNS16275 | No | Yes | Yes | Yes | Yes | No | Yes | Yes | Yes | Yes | No | Yes | 9 |
| Li S. Et al., 2024 | 10.1016/j.jclinane.2023.111285 | No | Yes | Yes | Yes | Yes | No | Yes | Yes | Yes | No | No | Yes | 8 |
| Li T et al., 2024 | 10.1007/s10143-024-02287-2 | No | Yes | Yes | Yes | Yes | No | Yes | Yes | Yes | Yes | No | Yes | 9 |
| Li Y et al. 2020 | 10.1016/j.bj.2020.06.004 | No | Yes | Yes | Yes | Yes | No | Yes | Yes | Yes | Yes | No | Yes | 9 |
| Lieber B A et al. 2016 | 10.3171/2015.4.JNS142719 | Yes | Yes | Yes | Yes | Yes | Yes | Yes | Yes | Yes | No | No | Yes | 9 |
| Linzey J.R. et al., 2017 | 10.1093/neuros/nyx046 | Yes | Yes | Yes | Yes | Yes | No | Yes | Yes | Yes | Yes | No | Yes | 9 |
| Liu B et al. 2018 | 10.1016/j.clnu.2018.11.008 | No | Yes | Yes | Yes | Yes | No | Yes | Yes | Yes | Yes | No | Yes | 9 |
| Liu L et al. 2018 | 10.1016/j.wneu.2018.06.238 | No | Yes | Yes | Yes | Yes | No | Yes | Yes | Yes | Yes | No | Yes | 9 |
| Liu M et al. 2020 | 10.1055/s-0040-1710520 | No | Yes | Yes | Yes | Yes | Yes | Yes | Yes | Yes | Yes | No | Yes | 10 |
| Liu W et al. 2021 | 10.1055/s-0040-1719138 | Yes | Yes | Yes | Yes | Yes | No | Yes | Yes | Yes | Yes | No | Yes | 9 |
| Liu Y et al., 2014 | 10.1016/j.clineuro.2014.08.002 | No | Yes | Yes | Yes | Yes | No | Yes | Yes | Yes | Yes | No | Yes | 9 |
| Loayza R et al. 2023 | 10.1007/s00701-023-05642-2 | No | Yes | Yes | Yes | Yes | No | Yes | Yes | Yes | Yes | No | Yes | 9 |
| Logghe H. Et al., 2015 | PMID: 25760209 | No | Yes | Yes | Yes | Yes | No | Yes | Yes | Yes | Yes | No | Yes | 9 |
| Lopez D.T. et al., 2023 | 10.25259/SNI_673_2023 | No | Yes | Yes | Yes | Yes | No | Yes | Yes | Yes | No | No | Yes | 8 |
| Lu Y et al. 2022 | [10.3389/fnins.2022.917752](https://doi.org/10.3389/fnins.2022.917752) | No | Yes | Yes | Yes | Yes | No | Yes | Yes | Yes | No | No | Yes | 8 |
| Luther E. Et al., 2020 | 10.1007/s00701-020-04239-3 | Yes | Yes | Yes | Yes | Yes | Yes | Yes | Yes | Yes | No | No | Yes | 9 |
| Lv Y et al. 2023 | 10.1186/s41016-023-00336-1 | Yes | Yes | Yes | Yes | Yes | Yes | Yes | Yes | Yes | No | No | Yes | 9 |
| Lwin S. Et al., 2012 | PMID: 22511048 | Yes | Yes | Yes | Yes | Yes | Yes | Yes | Yes | Yes | No | No | Yes | 9 |
| Maayan O et al., 2022 | 10.1007/s00701-021-05075-9 | Yes | Yes | Yes | Yes | Yes | Yes | Yes | Yes | Yes | No | No | Yes | 9 |
| Maayan O et al., 2023 | 10.1007/s11060-023-04294-7 | No | Yes | Yes | Yes | Yes | Yes | Yes | Yes | Yes | No | No | Yes | 9 |
| Madhugiri V.S. et al., 2011 | 10.1159/000330542 | No | Yes | Yes | Yes | Yes | Yes | Yes | Yes | Yes | No | No | Yes | 9 |
| Magil S.T. et al., 2023 | 10.1227/neu.0000000000002569 | No | Yes | Yes | Yes | Yes | Yes | Yes | Yes | Yes | No | No | Yes | 9 |
| Mahboubi H et al. 2016 | 10.1097/MAO.0000000000001178 | No | Yes | Yes | Yes | Yes | Yes | Yes | Yes | Yes | No | No | Yes | 9 |
| Makoshi Z et al., 2022 | 10.3171/2022.7.PEDS22231 | No | Yes | Yes | Yes | Yes | Yes | Yes | Yes | Yes | No | No | Yes | 9 |
| Mallela A et al. 2017 | 10.1093/neuros/nyx559 | Yes | Yes | Yes | Yes | Yes | Yes | Yes | Yes | Yes | No | No | Yes | 9 |
| Mallela AN et al., 2020 | [10.1016/j.jocn.2020.07.048](https://doi.org/10.1016/j.jocn.2020.07.048) | No | Yes | Yes | Yes | Yes | No | Yes | Yes | Yes | No | Yes | Yes | 9 |
| Mann C et al. 2021 | 10.1016/j.yebeh.2020.107715 | No | Yes | Yes | Yes | Yes | Yes | Yes | Yes | Yes | No | No | Yes | 9 |
| Marcus L.P. et al., 2014 | 10.3171/2014.1.JNS131264 | No | Yes | Yes | Yes | Yes | Yes | Yes | Yes | Yes | Yes | No | Yes | 10 |
| Martin A.J. et al., 2017 | 10.3171/2015.7.JNS15750 | No | Yes | Yes | Yes | Yes | Yes | Yes | Yes | Yes | No | No | Yes | 9 |
| Mascarenhas L et al. 2014 | 10.1016/j.wneu.2013.02.032 | No | Yes | Yes | Yes | Yes | Yes | Yes | Yes | Yes | No | No | Yes | 9 |
| Maye H. L. Et al., 2022 | 10.1016/j.wneu.2022.02.124 | Yes | Yes | Yes | Yes | Yes | Yes | Yes | Yes | Yes | No | No | Yes | 9 |
| Mcclelland S et al. 2007 | 10.1086/518580 | No | Yes | Yes | Yes | Yes | Yes | Yes | Yes | Yes | No | No | Yes | 9 |
| Mccutcheon B A et al. 2015 | 10.1016/j.wneu.2015.12.068 | No | Yes | Yes | Yes | Yes | Yes | Yes | Yes | Yes | No | No | Yes | 9 |
| Mccutcheon B.A. et al., 2016 | 10.1016/j.wneu.2016.01.089 | No | Yes | Yes | Yes | Yes | No | Yes | Yes | Yes | Yes | No | Yes | 9 |
| Mcgirt M J et al. 2003 | 10.1086/368191 | Yes | Yes | Yes | Yes | Yes | Yes | Yes | Yes | Yes | No | No | Yes | 9 |
| Mcgirt MJ et al., 2009 | 10.1227/01.NEU.0000349763.42238.E9 | No | Yes | Yes | Yes | Yes | No | Yes | Yes | Yes | Yes | No | Yes | 9 |
| Mehta G et al. 2022 | 10.3171/2021.5.JNS21772 | No | Yes | Yes | Yes | Yes | Yes | Yes | Yes | Yes | No | No | Yes | 9 |
| Meng Y et al. 2018 | 10.3171/2018.1.PEDS17476 | Yes | Yes | Yes | Yes | Yes | Yes | Yes | Yes | Yes | No | No | Yes | 9 |
| Mian SY et al., 2023 | 10.1016/j.wneu.2023.06.091 | Yes | Yes | Yes | Yes | Yes | Yes | Yes | Yes | Yes | No | No | Yes | 9 |
| Miller J.J. et al., 2001 | 10.1097/00129492-200111000-00033 | Yes | Yes | Yes | Yes | Yes | Yes | Yes | Yes | Yes | No | No | Yes | 9 |
| Missios S et al., 2015 | 10.1016/j.wneu.2015.04.052 | No | Yes | Yes | Yes | Yes | Yes | Yes | Yes | Yes | No | No | Yes | 9 |
| Mohamad S et al. 2016 | 10.21315/mjms2016.23.5.11 | Yes | Yes | Yes | Yes | Yes | Yes | Yes | Yes | Yes | No | No | Yes | 9 |
| Moiraghi A. Et al., 2021 | 10.3390/cancers13122911 | No | Yes | Yes | Yes | Yes | Yes | Yes | Yes | Yes | No | No | Yes | 9 |
| Mooney MA et al., 2018 | 10.3171/2017.5.JNS17394 | No | Yes | Yes | Yes | Yes | Yes | Yes | Yes | Yes | No | No | Yes | 9 |
| Moorthy RK et al., 2013 | 10.3109/02688697.2013.771138 | No | Yes | Yes | Yes | Yes | Yes | Yes | Yes | Yes | No | No | Yes | 9 |
| Morton R et al. 2018 | 10.3171/2016.11.JNS161917 | No | Yes | Yes | Yes | Yes | Yes | Yes | Yes | Yes | No | No | Yes | 9 |
| Morton R P et al. 2016 | 10.3171/2015.8.JNS151390 | No | Yes | Yes | Yes | Yes | Yes | Yes | Yes | Yes | No | No | Yes | 9 |
| Mracek J et al., 2015 | 10.1007/s00701-014-2333-0 | No | Yes | Yes | Yes | Yes | No | Yes | Yes | Yes | Yes | No | Yes | 9 |
| Muir M et al. 2019 | 10.1016/j.jocn.2019.08.059 | No | Yes | Yes | Yes | Yes | No | Yes | Yes | Yes | Yes | No | Yes | 9 |
| Muram S et al., 2023 | 10.3171/2022.5.JNS22430 | Yes | Yes | Yes | Yes | Yes | Yes | Yes | Yes | Yes | No | No | Yes | 9 |
| Murphy M E et al. 2016 | 10.1016/j.clineuro.2016.06.020 | No | Yes | Yes | Yes | Yes | Yes | Yes | Yes | Yes | No | No | Yes | 9 |
| Musavi L. Et al., 2020 | 10.1097/SCS.0000000000005695 | Yes | Yes | Yes | Yes | Yes | Yes | Yes | Yes | Yes | No | No | Yes | 9 |
| Nair S et al. 2023 | 10.1227/ons.0000000000000819 | No | Yes | Yes | Yes | Yes | Yes | Yes | Yes | Yes | No | No | Yes | 9 |
| Nair S K et al. 2023 | 10.3171/2022.8.JNS212799 | Yes | Yes | Yes | Yes | Yes | Yes | Yes | Yes | Yes | Yes | No | Yes | 10 |
| Nguyen A.V. et al., 2019 | 10.1016/j.clineuro.2019.05.017 | Yes | Yes | Yes | Yes | Yes | Yes | Yes | Yes | Yes | No | No | Yes | 9 |
| Northam W et al., 2020 | 10.1177/1460408619892141 | No | Yes | Yes | Yes | Yes | Yes | Yes | Yes | Yes | No | No | Yes | 9 |
| Nunno A et al. 2018 | 10.1016/j.wneu.2018.11.091 | No | Yes | Yes | Yes | Yes | Yes | Yes | Yes | Yes | No | No | Yes | 9 |
| Nusair AR et al., 2021 | 10.1089/sur.2020.020 | Yes | Yes | Yes | Yes | Yes | No | Yes | Yes | Yes | No | No | Yes | 8 |
| O'Keeffe A et al. 2012 | 10.3109/02688697.2011.626878 | Yes | Yes | Yes | Yes | Yes | Yes | Yes | Yes | Yes | No | No | Yes | 9 |
| Oakley GM et al., 2018 | 10.1017/S0022215117001499 | No | Yes | Yes | Yes | Yes | Yes | Yes | Yes | Yes | Yes | No | Yes | 10 |
| Oh W.O. et al., 2018 | 10.1159/000481437 | Yes | Yes | Yes | Yes | Yes | No | Yes | Yes | Yes | Yes | No | Yes | 9 |
| Okunlola A.I. et al., 2021 | 10.1080/02688697.2020.1812518 | Yes | Yes | Yes | Yes | Yes | No | Yes | Yes | Yes | No | No | Yes | 8 |
| Onkarappa S et al. 2023 | 10.1016/j.wneu.2023.05.104 | No | Yes | Yes | Yes | Yes | No | Yes | Yes | Yes | No | No | No | 7 |
| Ormond D R et al. 2019 | 10.1093/neuros/nyy125 | No | Yes | Yes | Yes | Yes | Yes | Yes | Yes | Yes | Yes | No | Yes | 10 |
| Orsi G.B. et al., 2006 | 10.1016/j.jhin.2006.02.022 | Yes | Yes | Yes | Yes | Yes | Yes | Yes | Yes | Yes | No | No | Yes | 9 |
| Osbun J et al. 2012 | 10.1016/j.wneu.2011.12.011 | Yes | Yes | Yes | Yes | Yes | Yes | Yes | Yes | Yes | No | No | Yes | 9 |
| Ozkan U et al., 2002 | 10.1007/s101430100173 | No | Yes | Yes | Yes | Yes | No | Yes | Yes | Yes | No | No | No | 7 |
| Paredes I et al. 2020 | 10.1007/s00701-020-04508-1 | Yes | Yes | Yes | Yes | Yes | Yes | Yes | Yes | Yes | Yes | No | Yes | 10 |
| Park Y et al. 2011 | 10.1159/000324903 | No | Yes | Yes | Yes | Yes | Yes | Yes | Yes | Yes | No | No | Yes | 9 |
| Patel AJ et al., 2014 | 10.3171/2014.1.PEDS13372 | No | Yes | Yes | Yes | Yes | Yes | Yes | Yes | Yes | No | No | Yes | 9 |
| Patel KS et al., 2014 | 10.1016/j.clineuro.2013.12.015 | Yes | Yes | Yes | Yes | Yes | Yes | Yes | Yes | Yes | Yes | No | Yes | 10 |
| Pattavilakom A et al. 2007 | 10.1016/j.jocn.2006.11.003 | No | Yes | Yes | Yes | Yes | No | Yes | Yes | Yes | Yes | No | Yes | 9 |
| Pavlicevic G et al., 2017 | 10.1016/j.jcms.2016.11.019 | No | Yes | Yes | Yes | Yes | No | Yes | Yes | Yes | Yes | No | Yes | 9 |
| Pereira J et al. 2012 | 10.4103/2152-7806.99941 | No | Yes | Yes | Yes | No | No | Yes | Yes | Yes | No | No | Yes | 7 |
| Pfnur A. Et al., 2024 | 10.1007/s10143-024-02309-z | Yes | Yes | Yes | Yes | Yes | Yes | Yes | Yes | Yes | Yes | Yes | Yes | 11 |
| Phang I et al. 2019 | 10.1016/j.wneu.2019.06.091 | No | Yes | Yes | Yes | Yes | Yes | Yes | Yes | Yes | Yes | Yes | Yes | 11 |
| Pie JS et al. 2019 | 10.1016/j.clineuro.2019.01.010 | No | Yes | Yes | Yes | Yes | Yes | Yes | Yes | Yes | Yes | Yes | Yes | 11 |
| Piitulainen JM et al. 2015 | 10.1016/j.wneu.2015.01.014 | No | Yes | Yes | Yes | Yes | Yes | Yes | Yes | Yes | Yes | Yes | Yes | 11 |
| Pirotte B et al. 2007 | 10.1007/s00381-007-0415-5 | No | Yes | Yes | Yes | Yes | No | Yes | Yes | Yes | Yes | Yes | Yes | 10 |
| Potts, M.B. et al, 2015 | 10.3171/2014.12.JNS14938 | No | Yes | Yes | Yes | No | No | Yes | Yes | Yes | Yes | Yes | Yes | 9 |
| Prablek M et al., 2021 | 10.1007/s00381-021-05170-3 | No | Yes | Yes | Yes | Yes | Yes | Yes | Yes | Yes | Yes | Yes | Yes | 11 |
| Puthumana J.S. et al., 2023 | [10.1097/SCS.0000000000008872](https://doi.org/10.1097/scs.0000000000008872) | No | Yes | Yes | Yes | Yes | Yes | Yes | Yes | Yes | Yes | Yes | Yes | 11 |
| Radmanesh F et al. 2009 | 10.3171/2009.2.PEDS08476 | Yes | Yes | Yes | Yes | Yes | Yes | Yes | Yes | Yes | Yes | Yes | Yes | 11 |
| Rae A I et al. 2023 | 10.1227/neu.0000000000002563 | Yes | Yes | Yes | Yes | Yes | Yes | Yes | Yes | Yes | Yes | Yes | Yes | 11 |
| Rajkumar S. Et al., 2024 | 10.1007/s00381-023-06076-y | No | Yes | Yes | Yes | Yes | Yes | Yes | Yes | Yes | Yes | Yes | Yes | 11 |
| Ramos T et al. 2016 | 10.1172/jci.insight.87919 | No | Yes | Yes | Yes | Yes | Yes | Yes | Yes | Yes | Yes | Yes | Yes | 11 |
| Rashidi A et al., 2019 | 10.1016/j.clineuro.2019.105509 | Yes | Yes | Yes | Yes | Yes | Yes | Yes | Yes | Yes | Yes | Yes | Yes | 11 |
| Rasouli J et al. 2016 | 10.1016/j.wneu.2016.07.063 | Yes | Yes | Yes | Yes | Yes | No | Yes | Yes | Yes | Yes | Yes | Yes | 10 |
| Ratanalert S et al. 2004 | 10.1016/j.jocn.2004.03.024 | No | Yes | Yes | Yes | Yes | Yes | Yes | Yes | Yes | Yes | Yes | Yes | 11 |
| Rauhala M et al., 2020 | 10.1007/s00701-020-04398-3 | No | Yes | Yes | Yes | Yes | Yes | Yes | Yes | Yes | Yes | Yes | Yes | 11 |
| Ravikumar V et al. 2017 | 10.1093/neuros/nyw127 | No | Yes | Yes | Yes | Yes | Yes | Yes | Yes | No | Yes | Yes | Yes | 10 |
| Raviv N et al. 2020 | 10.3171/2020.5.PEDS2019 | No | Yes | Yes | Yes | Yes | Yes | Yes | Yes | No | Yes | Yes | Yes | 10 |
| Reddy S. Et al., 2014 | PMID: 25264642 | No | Yes | Yes | Yes | Yes | Yes | Yes | Yes | Yes | Yes | Yes | Yes | 11 |
| Rehman AU et al., 2010 | 10.3171/2010.2.PEDS09151 | Yes | Yes | Yes | Yes | Yes | Yes | Yes | Yes | Yes | Yes | Yes | Yes | 11 |
| Renz N. Et al., 2018 | 10.1016/j.wneu.2018.05.017 | Yes | Yes | No | Yes | Yes | Yes | Yes | Yes | Yes | Yes | Yes | Yes | 10 |
| Reponen E et al. 2016 | 10.1016/j.wneu.2016.03.102 | No | Yes | Yes | Yes | Yes | Yes | Yes | Yes | Yes | Yes | Yes | Yes | 11 |
| Reponen E et al. 2019 | 10.1093/neuros/nyy380 | No | Yes | Yes | Yes | Yes | Yes | Yes | Yes | Yes | Yes | Yes | Yes | 11 |
| Ribeiro B B et al. 2022 | 10.1097/j.pbj.0000000000000152 | Yes | Yes | Yes | Yes | Yes | Yes | Yes | Yes | Yes | Yes | Yes | Yes | 11 |
| Richards H.K. et al., 2009 | 10.3171/2009.4.PEDS09210 | No | Yes | Yes | Yes | Yes | Yes | Yes | Yes | Yes | Yes | Yes | Yes | 11 |
| Rivero-Garvia M et al. 2010 | 10.1007/s00701-010-0905-1 | Yes | Yes | Yes | Yes | Yes | Yes | Yes | Yes | No | No | Yes | Yes | 9 |
| Rizvi I et al. 2023 | 10.1177/10556656221085478 | No | Yes | Yes | Yes | Yes | Yes | Yes | Yes | Yes | Yes | Yes | Yes | 11 |
| Roblot P. Et al., 2023 | 10.1016/j.neuchi.2023.101458 | No | Yes | Yes | Yes | Yes | No | Yes | Yes | Yes | Yes | Yes | Yes | 10 |
| Rocque BG et al., 2018 | 10.3171/2018.3.PEDS17234 | No | Yes | Yes | Yes | Yes | Yes | Yes | Yes | Yes | Yes | Yes | Yes | 11 |
| Rolston J D et al. 2016 | 10.1016/j.eplepsyres.2016.05.001 | No | Yes | Yes | Yes | Yes | Yes | Yes | Yes | Yes | Yes | Yes | Yes | 11 |
| Rosa M et al. 2017 | 10.1016/j.wneu.2016.09.069 | Yes | Yes | No | Yes | Yes | Yes | Yes | Yes | Yes | Yes | Yes | Yes | 10 |
| Roth J., et al., 2023 | 10.1111/epi.17796 | No | Yes | Yes | Yes | Yes | No | Yes | Yes | Yes | Yes | Yes | Yes | 10 |
| Rothlind J et al. 2021 | 10.1016/j.parkreldis.2021.12.011 | No | Yes | Yes | Yes | Yes | Yes | Yes | Yes | Yes | Yes | Yes | Yes | 11 |
| Rubeli S.L. et al., 2019 | 10.3171/2019.5.FOCUS19272 | Yes | Yes | Yes | Yes | Yes | Yes | Yes | Yes | Yes | Yes | Yes | Yes | 11 |
| Rumalla K et al., 2021 | 10.1016/j.wneu.2021.07.004 | No | Yes | Yes | Yes | Yes | No | Yes | Yes | Yes | Yes | Yes | Yes | 10 |
| Sæhle T. Et al., 2015 | [10.3171/2014.12.JNS141029](https://doi.org/10.3171/2014.12.jns141029) | No | Yes | Yes | Yes | Yes | Yes | Yes | Yes | Yes | Yes | Yes | Yes | 11 |
| Sacko O et al, 2007 | 10.1227/NEU.0b013e31820c02a3 | No | Yes | Yes | Yes | No | No | Yes | Yes | Yes | Yes | Yes | Yes | 9 |
| Saenz A et al. 2021 | 10.1007/s00381-021-05256-y | Yes | Yes | Yes | Yes | Yes | Yes | Yes | Yes | Yes | Yes | Yes | Yes | 11 |
| Sakarunchai I et al. 2016 | 10.1016/j.inat.2016.01.003 | No | Yes | Yes | Yes | Yes | Yes | Yes | Yes | Yes | Yes | Yes | Yes | 11 |
| Saleh C. Et al., 2015 | 10.1007/s11920-015-0565-1 | No | Yes | Yes | Yes | No | No | Yes | Yes | Yes | Yes | Yes | No | 8 |
| Salle H et al. 2021 | 10.1007/s15010-020-01534-0 | Yes | Yes | Yes | Yes | Yes | Yes | Yes | Yes | Yes | Yes | Yes | Yes | 11 |
| Salmanov A.G. et al., 2022 | PMID: 35092242 | Yes | Yes | Yes | Yes | Yes | Yes | Yes | Yes | Yes | Yes | Yes | Yes | 11 |
| Sander C et al., 2021 | 10.1016/j.wneu.2021.01.123 | No | Yes | Yes | Yes | Yes | Yes | Yes | Yes | No | No | Yes | Yes | 9 |
| Sander C. Et al., 2020 | 10.1007/s00701-020-04521-4 | No | Yes | Yes | Yes | Yes | Yes | Yes | Yes | Yes | Yes | Yes | Yes | 11 |
| Sangtongjaraskul S et al. 2023 | 10.3171/2023.2.PEDS22535 | No | Yes | Yes | Yes | Yes | Yes | Yes | Yes | Yes | Yes | Yes | Yes | 11 |
| Sangtongjaraskul S. Et al., 2023 | 10.5005/jp-journals-10071-24418 | No | Yes | Yes | Yes | Yes | Yes | Yes | Yes | No | No | Yes | Yes | 9 |
| Sankey EW et al. 2015 | 10.3171/2015.4.JNS15129 | No | Yes | Yes | Yes | Yes | Yes | Yes | Yes | Yes | Yes | Yes | Yes | 11 |
| Saramma PP et al., 2011 | 10.4103/0028-3886.76850 | Yes | Yes | Yes | Yes | Yes | Yes | Yes | Yes | Yes | Yes | Yes | Yes | 11 |
| Sathaporntheera P et al. 2020 | 10.1016/j.inat.2020.100865 | No | Yes | Yes | Yes | Yes | Yes | Yes | Yes | No | No | Yes | Yes | 9 |
| Savin I. Et al., 2018 | 10.1016/j.jcrc.2018.01.022 | No | Yes | Yes | Yes | Yes | Yes | Yes | Yes | Yes | Yes | Yes | Yes | 11 |
| Sayadi JJ et al., 2023 | 10.1016/j.neurom.2022.02.227 | No | Yes | Yes | Yes | Yes | Yes | Yes | Yes | Yes | Yes | Yes | Yes | 11 |
| Scheer M et al. 2023 | 10.3390/jpm13071117 | Yes | Yes | Yes | Yes | Yes | Yes | Yes | Yes | Yes | Yes | Yes | Yes | 11 |
| Schellekes N et al. 2021 | 10.3171/2020.9.JNS201980 | No | Yes | Yes | Yes | Yes | Yes | Yes | Yes | Yes | Yes | Yes | Yes | 11 |
| Schipmann S et al. 2022 | 10.3171/2022.7.JNS22691 | No | Yes | Yes | Yes | Yes | Yes | Yes | Yes | Yes | Yes | Yes | Yes | 11 |
| Schipmann S et al., 2018 | 10.1007/s00701-018-03790-4 | Yes | Yes | Yes | Yes | Yes | Yes | Yes | Yes | Yes | Yes | Yes | Yes | 11 |
| Schipmann S et al., 2022 | 10.1007/s00701-021-05044-2 | No | Yes | Yes | Yes | Yes | Yes | Yes | Yes | Yes | Yes | Yes | Yes | 11 |
| Schmeiser B et al. 2017 | 10.1093/neuros/nyx138 | No | Yes | Yes | Yes | Yes | Yes | Yes | Yes | Yes | Yes | Yes | Yes | 11 |
| Schneider M. Et al., 2021 | 10.1007/s10143-020-01281-8 | No | Yes | Yes | Yes | Yes | Yes | Yes | Yes | Yes | Yes | Yes | Yes | 11 |
| Schodel P et al. 2020 | 10.1002/cam4.3402 | No | Yes | Yes | Yes | Yes | Yes | Yes | Yes | Yes | Yes | Yes | Yes | 11 |
| Schutz A et al. 2018 | 10.3171/2018.6.JNS172605 | No | Yes | Yes | Yes | Yes | No | Yes | Yes | Yes | Yes | Yes | Yes | 10 |
| Schwarz F et al. 2015 | 10.1016/j.clineuro.2015.08.002 | No | Yes | Yes | Yes | Yes | Yes | Yes | Yes | Yes | Yes | Yes | Yes | 11 |
| Seicean A et al. 2020 | 10.1016/j.inat.2020.100692 | No | Yes | Yes | Yes | Yes | Yes | Yes | Yes | Yes | Yes | Yes | Yes | 11 |
| Serrato P et al., 2023 | [10.1097/SCS.0000000000009920](https://doi.org/10.1097/scs.0000000000009920) | No | Yes | Yes | Yes | Yes | Yes | Yes | Yes | No | No | Yes | Yes | 9 |
| Servello D et al., 2015 | 10.1007/s00701-023-05799-w | Yes | Yes | Yes | Yes | Yes | Yes | Yes | Yes | Yes | Yes | Yes | Yes | 11 |
| Shafei M et al., 2021 | 10.1016/j.jocn.2021.06.042 | No | Yes | Yes | Yes | Yes | No | Yes | Yes | Yes | Yes | Yes | Yes | 10 |
| Shaftel KA et al. 2022 | 10.1227/neu.0000000000002119 | No | Yes | Yes | Yes | Yes | Yes | Yes | Yes | Yes | Yes | Yes | Yes | 11 |
| Shallwani H et al. 2018 | 10.1093/neuros/nyx211 | No | Yes | Yes | Yes | Yes | No | Yes | Yes | Yes | Yes | Yes | Yes | 10 |
| Sharafat S et al. 2023 | 10.12669/pjms.39.1.6408 | \| No \| \| --- \| | Yes | Yes | Yes | Yes | Yes | Yes | Yes | Yes | Yes | Yes | Yes | 11 |
| Sheitoyan-Pesant C et al, 2017 | 10.1016/j.ajic.2016.11.020 | No | Yes | Yes | Yes | Yes | Yes | Yes | Yes | Yes | Yes | Yes | Yes | 11 |
| Shekhar H. Et al., 2016 | 10.3109/02688697.2015.1096903 | Yes | Yes | Yes | Yes | Yes | Yes | Yes | Yes | Yes | Yes | Yes | Yes | 11 |
| Sherrod B et al. 2017 | 10.1016/j.clineuro.2017.05.027 | No | Yes | Yes | Yes | Yes | Yes | Yes | Yes | Yes | Yes | Yes | Yes | 11 |
| Shi Z et al. 2017 | 10.1080/02688697.2016.1253827 | Yes | Yes | Yes | Yes | Yes | Yes | Yes | Yes | Yes | Yes | Yes | Yes | 11 |
| Shibahashi K et al. 2017 | [10.1016/j.wneu.2017.01.106](https://doi.org/10.1016/j.wneu.2017.01.106) | Yes | Yes | Yes | Yes | Yes | Yes | Yes | Yes | Yes | Yes | Yes | Yes | 11 |
| Shibamura-Fujiogi M et al. 2021 | [10.1186/s12871-021-01342-5](https://doi.org/10.1186/s12871-021-01342-5) | Yes | Yes | Yes | Yes | Yes | Yes | Yes | Yes | Yes | Yes | Yes | Yes | 11 |
| Shiferaw A A et al. 2024 | [10.1016/j.wneu.2023.10.077](https://doi.org/10.1016/j.wneu.2023.10.077) | No | Yes | Yes | Yes | Yes | Yes | Yes | Yes | No | No | Yes | Yes | 9 |
| Shimizu K et al. 2015 | [10.1055/s-0034-1396660](https://doi.org/10.1055/s-0034-1396660) | No | Yes | Yes | Yes | No | No | Yes | Yes | Yes | Yes | Yes | Yes | 9 |
| Shin YS et al., 2024 | [10.23736/S0390-5616.19.04693-9](https://doi.org/10.23736/s0390-5616.19.04693-9) | No | Yes | Yes | Yes | Yes | Yes | Yes | Yes | No | No | Yes | Yes | 9 |
| Shinoura N. Et al., 2004 | [10.1080/02688690400022771](https://doi.org/10.1080/02688690400022771) | Yes | Yes | Yes | Yes | Yes | Yes | Yes | Yes | Yes | Yes | Yes | Yes | 11 |
| Sicking J et al. 2018 | [10.1007/s00701-018-3617-6](https://doi.org/10.1007/s00701-018-3617-6) | No | Yes | Yes | Yes | Yes | Yes | Yes | Yes | Yes | Yes | Yes | Yes | 11 |
| Singh A. K. Et al., 2014 | [10.4103/0028-3886.132364](https://doi.org/10.4103/0028-3886.132364) | No | Yes | Yes | Yes | No | No | Yes | Yes | Yes | Yes | Yes | Yes | 9 |
| Skyman S et al. 2020 | [10.1007/s00701-020-04309-6](https://doi.org/10.1007/s00701-020-04309-6) | Yes | Yes | Yes | Yes | Yes | Yes | Yes | Yes | Yes | Yes | Yes | Yes | 11 |
| Slattery WH III et al. 2001 | [10.1097/00129492-200111000-00031](https://doi.org/10.1097/00129492-200111000-00031) | No | Yes | Yes | Yes | Yes | Yes | Yes | Yes | Yes | Yes | Yes | Yes | 11 |
| Slot E.M.H. et al., 2023 | [10.3171/2022.11.PEDS22421](https://doi.org/10.3171/2022.11.peds22421) | No | Yes | Yes | Yes | Yes | Yes | Yes | Yes | Yes | Yes | Yes | Yes | 11 |
| Smith K et al., 2022 | 10.1097/01.ccm.0000809444.94667.19 | No | Yes | Yes | Yes | Yes | Yes | Yes | Yes | Yes | Yes | Yes | Yes | 11 |
| Sneh-Arbib O et al. 2013 | [10.1007/s10096-013-1904-y](https://doi.org/10.1007/s10096-013-1904-y) | Yes | Yes | Yes | Yes | Yes | Yes | Yes | Yes | Yes | Yes | Yes | Yes | 11 |
| So R et al. 2022 | [10.1227/ons.0000000000000546](https://doi.org/10.1227/ons.0000000000000546) | No | Yes | Yes | Yes | Yes | Yes | Yes | Yes | Yes | Yes | Yes | Yes | 11 |
| Soleman J et al. 2021 | [10.1016/j.wneu.2020.10.138](https://doi.org/10.1016/j.wneu.2020.10.138) | No | Yes | Yes | Yes | Yes | Yes | Yes | Yes | Yes | Yes | Yes | Yes | 11 |
| Soto J et al. 2023 | [10.1016/j.wneu.2023.04.020](https://doi.org/10.1016/j.wneu.2023.04.020) | No | Yes | Yes | Yes | Yes | Yes | Yes | Yes | Yes | Yes | Yes | Yes | 11 |
| Sousa S. Et al., 2023 | [10.3171/2023.2.JNS222262](https://doi.org/10.3171/2023.2.jns222262) | No | Yes | Yes | Yes | Yes | Yes | Yes | Yes | Yes | Yes | Yes | Yes | 11 |
| Spille DC et al., 2022 | [10.1055/a-1911-8678](https://doi.org/10.1055/a-1911-8678) | No | Yes | Yes | Yes | Yes | Yes | Yes | Yes | Yes | Yes | Yes | Yes | 11 |
| Sponton L et al. 2022 | [10.1055/s-0042-1751000](https://doi.org/10.1055/s-0042-1751000) | No | Yes | Yes | Yes | Yes | Yes | Yes | Yes | Yes | Yes | Yes | Yes | 11 |
| Spuck S et al. 2010 | [10.1227/NEU.0b013e3181f88867](https://doi.org/10.1227/neu.0b013e3181f88867) | No | Yes | Yes | Yes | Yes | Yes | Yes | Yes | Yes | Yes | Yes | Yes | 11 |
| Stoker M.A. et all, 2012 | [10.1055/s-0032-1312709](https://doi.org/10.1055/s-0032-1312709) | No | Yes | Yes | Yes | Yes | Yes | Yes | Yes | Yes | Yes | Yes | Yes | 11 |
| Strahm C. Et al., 2018 | [10.1016/j.wneu.2017.12.062](https://doi.org/10.1016/j.wneu.2017.12.062) | Yes | Yes | Yes | Yes | Yes | Yes | Yes | Yes | Yes | Yes | Yes | Yes | 11 |
| Sughrue ME et al. 2011 | [10.1016/j.jocn.2011.01.016](https://doi.org/10.1016/j.jocn.2011.01.016) | Yes | Yes | Yes | Yes | Yes | Yes | Yes | Yes | Yes | Yes | Yes | Yes | 11 |
| Tabata S et al. 2022 | [10.1016/j.jocn.2022.02.020](https://doi.org/10.1016/j.jocn.2022.02.020) | No | Yes | Yes | Yes | Yes | Yes | Yes | Yes | Yes | Yes | Yes | Yes | 11 |
| Tacconelli E et al. 2008 | [10.1016/j.jhin.2008.04.032](https://doi.org/10.1016/j.jhin.2008.04.032) | Yes | Yes | Yes | Yes | Yes | Yes | Yes | Yes | Yes | Yes | Yes | Yes | 11 |
| Tacconi L et al. 2019 | [10.1016/j.wneu.2018.10.023](https://doi.org/10.1016/j.wneu.2018.10.023) | No | Yes | Yes | Yes | Yes | No | Yes | Yes | Yes | Yes | Yes | Yes | 10 |
| Tafreshi A.R. et al., 2021 | [10.1016/j.clineuro.2020.106372](https://doi.org/10.1016/j.clineuro.2020.106372) | No | Yes | Yes | Yes | Yes | Yes | Yes | Yes | Yes | Yes | Yes | Yes | 11 |
| Takami H et al. 2021 | [10.1016/j.wneu.2021.11.010](https://doi.org/10.1016/j.wneu.2021.11.010) | No | Yes | Yes | Yes | Yes | Yes | Yes | Yes | Yes | Yes | Yes | Yes | 11 |
| Takeuchi S et al., 2015 | [10.1016/j.wneu.2015.01.030](https://doi.org/10.1016/j.wneu.2015.01.030) | No | Yes | Yes | Yes | Yes | Yes | Yes | Yes | No | No | Yes | Yes | 9 |
| Tanaka K. Et al., 2019 | [10.1016/j.anl.2022.03.006](https://doi.org/10.1016/j.anl.2022.03.006) | No | Yes | Yes | Yes | Yes | Yes | Yes | Yes | Yes | Yes | Yes | Yes | 11 |
| Tandon N et al., 2019 | [10.1001/jamaneurol.2019.0098](https://doi.org/10.1001/jamaneurol.2019.0098) | No | Yes | Yes | Yes | Yes | No | Yes | Yes | Yes | Yes | Yes | Yes | 10 |
| Tang K et al. 2001 | [10.1159/000050379](https://doi.org/10.1159/000050379) | No | Yes | Yes | Yes | Yes | Yes | Yes | Yes | Yes | Yes | Yes | Yes | 11 |
| Taylor BES et al., 2016 | [10.1227/NEU.0000000000001110](https://doi.org/10.1227/neu.0000000000001110) | No | Yes | Yes | Yes | Yes | No | Yes | Yes | Yes | Yes | Yes | Yes | 10 |
| Teshita G et al., 2024 | [10.1016/j.wnsx.2023.100264](https://doi.org/10.1016/j.wnsx.2023.100264) | No | Yes | Yes | Yes | Yes | Yes | Yes | Yes | Yes | Yes | Yes | Yes | 11 |
| Test MR et al. 2019 | [10.3171/2019.2.PEDS18638](https://doi.org/10.3171/2019.2.peds18638) | Yes | Yes | Yes | Yes | Yes | Yes | Yes | Yes | Yes | Yes | Yes | Yes | 11 |
| Tew J et al. 2017 | [10.1093/ons/opw004](https://doi.org/10.1093/ons/opw004) | No | Yes | Yes | Yes | Yes | Yes | Yes | Yes | Yes | Yes | Yes | Yes | 11 |
| Thenier-Villa J.L. et al., 2018 | [10.3171/2018.2.PEDS17717](https://doi.org/10.3171/2018.2.peds17717) | No | Yes | Yes | Yes | Yes | Yes | Yes | Yes | Yes | Yes | Yes | Yes | 11 |
| Thompson DNP et al. 2007 | [10.3171/ped.2007.106.1.15](https://doi.org/10.3171/ped.2007.106.1.15) | No | Yes | Yes | Yes | Yes | Yes | Yes | Yes | Yes | Yes | Yes | Yes | 11 |
| Thu L T A et al. 2007 | [10.1086/516661](https://doi.org/10.1086/516661) | Yes | Yes | Yes | Yes | Yes | Yes | Yes | Yes | Yes | Yes | Yes | Yes | 11 |
| Toescu S.M. et al., 2021 | [10.3171/2020.6.PEDS2089](https://doi.org/10.3171/2020.6.peds2089) | No | Yes | Yes | Yes | Yes | Yes | Yes | Yes | Yes | Yes | Yes | Yes | 11 |
| Tokimura H et al., 2009 | [10.1016/j.jcms.2009.06.003](https://doi.org/10.1016/j.jcms.2009.06.003) | Yes | Yes | Yes | Yes | Yes | Yes | Yes | Yes | Yes | Yes | Yes | Yes | 11 |
| Tolleson C et al. 2014 | [10.1159/000362934](https://doi.org/10.1159/000362934) | Yes | Yes | Yes | Yes | Yes | Yes | Yes | Yes | Yes | Yes | Yes | Yes | 11 |
| Tomatis A et al. 2019 | [10.1016/j.wneu.2019.06.150](https://doi.org/10.1016/j.wneu.2019.06.150) | No | Yes | Yes | Yes | Yes | Yes | Yes | Yes | Yes | Yes | Yes | Yes | 11 |
| Torres S. Et al., 2018 | [10.1016/j.bjid.2018.08.001](https://doi.org/10.1016/j.bjid.2018.08.001) | Yes | Yes | Yes | Yes | Yes | Yes | Yes | Yes | Yes | Yes | Yes | Yes | 11 |
| Trinh V et al. 2015 | [10.3171/2014.9.JNS131648](https://doi.org/10.3171/2014.9.jns131648) | No | Yes | Yes | Yes | Yes | Yes | Yes | Yes | Yes | Yes | Yes | Yes | 11 |
| Trungu S et al., 2022 | [10.3390/jcm11051286](https://doi.org/10.3390/jcm11051286) | No | Yes | Yes | Yes | Yes | Yes | Yes | Yes | Yes | Yes | Yes | Yes | 11 |
| Tsang ACO et al., 2015 | [10.1016/j.jocn.2014.11.021](https://doi.org/10.1016/j.jocn.2014.11.021) | No | Yes | Yes | Yes | Yes | Yes | Yes | Yes | Yes | Yes | Yes | Yes | 11 |
| Tunthanathip T et al. 2019 | [10.3171/2019.5.FOCUS19241](https://doi.org/10.3171/2019.5.focus19241) | Yes | Yes | Yes | Yes | Yes | Yes | Yes | Yes | Yes | Yes | Yes | Yes | 11 |
| Tzikos G et al. 2022 | [10.3390/nu14132620](https://doi.org/10.3390/nu14132620) | Yes | Yes | Yes | Yes | Yes | Yes | Yes | Yes | Yes | Yes | Yes | Yes | 11 |
| Uche E.O. et al., 2013 | [10.1159/000357384](https://doi.org/10.1159/000357384) | Yes | Yes | Yes | Yes | Yes | Yes | Yes | Yes | Yes | Yes | Yes | Yes | 11 |
| Uzuka T et al. 2017 | [10.2176/nmc.oa.2017-0034](https://doi.org/10.2176/nmc.oa.2017-0034) | Yes | Yes | Yes | Yes | Yes | Yes | Yes | Yes | Yes | Yes | Yes | Yes | 11 |
| Van der Veken J et al. 2014 | [10.1007/s00701-014-2042-8](https://doi.org/10.1007/s00701-014-2042-8) | No | Yes | Yes | Yes | Yes | Yes | Yes | Yes | Yes | Yes | Yes | Yes | 11 |
| Van der Vlis T et al. 2022 | [10.1016/j.neurom.2021.12.011](https://doi.org/10.1016/j.neurom.2021.12.011) | No | Yes | Yes | Yes | Yes | Yes | Yes | Yes | Yes | Yes | Yes | Yes | 11 |
| Van Schooten J et al. 2023 | [10.1016/j.bas.2023.102733](https://doi.org/10.1016/j.bas.2023.102733) | No | Yes | Yes | Yes | Yes | Yes | Yes | Yes | Yes | Yes | Yes | Yes | 11 |
| Vankipuram S et al. 2019 | [10.1016/j.wneu.2019.12.004](https://doi.org/10.1016/j.wneu.2019.12.004) | No | Yes | Yes | Yes | Yes | No | Yes | Yes | Yes | Yes | Yes | Yes | 10 |
| Veldeman M et al., 2020 | [10.3171/2020.2.JNS193335](https://doi.org/10.3171/2020.2.jns193335) | Yes | Yes | Yes | Yes | Yes | Yes | Yes | Yes | Yes | Yes | Yes | Yes | 11 |
| Venable GT et al., 2020 | [10.1016/j.wneu.2019.11.018](https://doi.org/10.1016/j.wneu.2019.11.018) | No | Yes | Yes | Yes | Yes | No | Yes | Yes | Yes | Yes | Yes | Yes | 10 |
| Verberk J D M et al. 2016 | [10.1016/j.jhin.2015.12.018](https://doi.org/10.1016/j.jhin.2015.12.018) | Yes | Yes | Yes | Yes | Yes | Yes | Yes | Yes | Yes | Yes | Yes | Yes | 11 |
| Vergani F et al. 2010 | [10.1016/j.wneu.2010.01.017](https://doi.org/10.1016/j.wneu.2010.01.017) | No | Yes | Yes | Yes | Yes | Yes | Yes | Yes | Yes | Yes | Yes | Yes | 11 |
| Viken H et al. 2018 | [10.1016/j.wneu.2018.01.137](https://doi.org/10.1016/j.wneu.2018.01.137) | No | Yes | Yes | Yes | Yes | Yes | Yes | Yes | Yes | Yes | Yes | Yes | 11 |
| Villavicencio AT et al., 2003 | [10.1016/s0090-3019(03)00070-3](https://doi.org/10.1016/s0090-3019(03)00070-3) | No | Yes | Yes | Yes | No | No | Yes | Yes | Yes | Yes | Yes | Yes | 9 |
| Volsky P.G. et al., 2017 | [10.1002/lary.26403](https://doi.org/10.1002/lary.26403) | No | Yes | Yes | Yes | No | No | Yes | Yes | Yes | Yes | Yes | Yes | 9 |
| Wachter D et al., 2013 | [10.1016/j.clineuro.2012.12.002](https://doi.org/10.1016/j.clineuro.2012.12.002) | No | Yes | Yes | Yes | Yes | Yes | Yes | Yes | Yes | Yes | Yes | Yes | 11 |
| Walaszek M. 2015 | PMID: 26519848 | Yes | Yes | Yes | Yes | Yes | Yes | Yes | Yes | No | No | Yes | Yes | 9 |
| Walcott B et al. 2013 | [10.3171/2013.8.JNS13703](https://doi.org/10.3171/2013.8.jns13703) | Yes | Yes | Yes | Yes | Yes | Yes | Yes | Yes | Yes | Yes | Yes | Yes | 11 |
| Walcott BP et al. 2013 | [10.3171/2013.1.JNS121626](https://doi.org/10.3171/2013.1.jns121626) | Yes | Yes | Yes | Yes | Yes | Yes | Yes | Yes | No | No | Yes | Yes | 9 |
| Walcott BP et al. 2013 | [10.3171/2013.8.JNS13703](https://doi.org/10.3171/2013.8.jns13703) | Yes | Yes | Yes | Yes | Yes | Yes | Yes | Yes | Yes | Yes | Yes | Yes | 11 |
| Wang D D et al. 2017 | [10.3171/2016.9.JNS16149](https://doi.org/10.3171/2016.9.jns16149) | No | Yes | Yes | Yes | Yes | Yes | Yes | Yes | Yes | Yes | Yes | Yes | 11 |
| Wang J et al., 2023 | [10.1080/02688697.2021.1902472](https://doi.org/10.1080/02688697.2021.1902472) | Yes | Yes | Yes | Yes | Yes | No | Yes | Yes | Yes | Yes | Yes | Yes | 10 |
| Wang L et al., 2017 | [10.3171/2016.9.JNS16559](https://doi.org/10.3171/2016.9.jns16559) | No | Yes | Yes | Yes | Yes | Yes | Yes | Yes | Yes | Yes | Yes | Yes | 11 |
| Wang L. Et al., 2022 | [10.1016/j.jclinane.2021.110575](https://doi.org/10.1016/j.jclinane.2021.110575) | No | Yes | Yes | Yes | Yes | Yes | Yes | Yes | Yes | Yes | Yes | Yes | 11 |
| Wang Y et al. 2022 | [10.3389/fonc.2022.860257](https://doi.org/10.3389/fonc.2022.860257) | No | Yes | Yes | Yes | Yes | Yes | Yes | Yes | Yes | Yes | Yes | Yes | 11 |
| Weber L. Et al., 2022 | [10.3389/fonc.2022.959072](https://doi.org/10.3389/fonc.2022.959072) | No | Yes | Yes | Yes | Yes | No | Yes | Yes | Yes | Yes | Yes | Yes | 10 |
| Westman M et al., 2018 | [10.1016/j.jocn.2018.04.076](https://doi.org/10.1016/j.jocn.2018.04.076) | No | Yes | Yes | Yes | Yes | Yes | Yes | Yes | Yes | Yes | Yes | Yes | 11 |
| Whitby M et al. 2000 | [10.1080/02688690042843](https://doi.org/10.1080/02688690042843) | Yes | Yes | Yes | Yes | Yes | Yes | Yes | Yes | Yes | Yes | Yes | Yes | 11 |
| White-Dzuro G A et al. 2016 | [10.1159/000442893](https://doi.org/10.1159/000442893) | Yes | Yes | Yes | Yes | Yes | No | Yes | Yes | Yes | Yes | Yes | Yes | 10 |
| Widen J et al. 2017 | [10.1007/s00701-016-3039-2](https://doi.org/10.1007/s00701-016-3039-2) | Yes | Yes | Yes | Yes | Yes | Yes | Yes | Yes | No | No | Yes | Yes | 9 |
| Williams M A et al. 2022 | [10.3171/2022.1.JNS212782](https://doi.org/10.3171/2022.1.jns212782) | No | Yes | Yes | Yes | Yes | Yes | Yes | Yes | No | No | Yes | Yes | 9 |
| Winston KR et al., 2007 | [10.3171/ped.2007.106.6.450](https://doi.org/10.3171/ped.2007.106.6.450) | Yes | Yes | Yes | Yes | Yes | No | Yes | Yes | Yes | Yes | Yes | Yes | 10 |
| Wu C et al., 2014 | [10.5137/1019-5149.JTN.9281-13.1](https://doi.org/10.5137/1019-5149.jtn.9281-13.1) | Yes | Yes | Yes | Yes | Yes | Yes | Yes | Yes | Yes | Yes | Yes | Yes | 11 |
| Xia Y et al. 2019 | [10.1093/ons/opz163](https://doi.org/10.1093/ons/opz163) | No | Yes | Yes | Yes | Yes | Yes | Yes | Yes | Yes | Yes | Yes | Yes | 11 |
| Xu L et al., 2022 | [10.1186/s12879-022-07719-2](https://doi.org/10.1186/s12879-022-07719-2) | Yes | Yes | Yes | Yes | Yes | Yes | Yes | Yes | Yes | Yes | Yes | Yes | 11 |
| Yang M et al., 2020 | [10.2217/3dp-2019-0022](https://doi.org/10.2217/3dp-2019-0022) | No | Yes | Yes | Yes | Yes | No | Yes | Yes | Yes | Yes | Yes | Yes | 10 |
| Yang N.R. et al., 2018 | [10.1016/j.wneu.2017.10.117](https://doi.org/10.1016/j.wneu.2017.10.117) | No | Yes | Yes | Yes | Yes | Yes | Yes | Yes | Yes | Yes | Yes | Yes | 11 |
| Yatimparvar G. Et al., 2020 | P J M H S Vol. 14, NO. 2, APR – JUN 2020. | Yes | Yes | Yes | Yes | Yes | Yes | Yes | Yes | Yes | Yes | Yes | Yes | 11 |
| Yeap MC et al. 2022 | [10.1016/j.wneu.2021.09.111](https://doi.org/10.1016/j.wneu.2021.09.111) | Yes | Yes | Yes | Yes | Yes | Yes | Yes | Yes | Yes | Yes | Yes | Yes | 11 |
| Yeung L C et al. 2005 | [10.1227/01.neu.0000156472.29749.b8](https://doi.org/10.1227/01.neu.0000156472.29749.b8) | Yes | Yes | Yes | Yes | Yes | Yes | Yes | Yes | Yes | Yes | Yes | Yes | 11 |
| Youn S.B. et al., 2023 | [10.3340/jkns.2023.0024](https://doi.org/10.3340/jkns.2023.0024) | No | Yes | Yes | Yes | Yes | Yes | Yes | Yes | Yes | Yes | Yes | Yes | 11 |
| Yusufali TS et al. 2016 | The ANNALS of AFRICAN SURGERY. January 2016 Volume 13 Issue 1 | No | Yes | Yes | Yes | Yes | Yes | Yes | Yes | Yes | Yes | Yes | Yes | 11 |
| Zaidi H.A. et al., 2017 | [10.1093/neuros/nyw139](https://doi.org/10.1093/neuros/nyw139) | No | Yes | Yes | Yes | Yes | Yes | Yes | Yes | Yes | Yes | Yes | Yes | 11 |
| Zakhary G et al. 2014 | [10.1016/j.jcms.2014.05.014](https://doi.org/10.1016/j.jcms.2014.05.014) | No | Yes | Yes | Yes | Yes | Yes | Yes | Yes | Yes | Yes | Yes | Yes | 11 |
| Zanaty M et al., 2015 | [10.3171/2014.9.JNS14405](https://doi.org/10.3171/2014.9.jns14405) | No | Yes | Yes | Yes | Yes | Yes | Yes | Yes | Yes | Yes | Yes | Yes | 11 |
| Zeng L et al., 2015 | [10.1007/s10143-015-0619-1](https://doi.org/10.1007/s10143-015-0619-1) | No | Yes | Yes | Yes | Yes | Yes | Yes | Yes | Yes | Yes | Yes | Yes | 11 |
| Zhang L et al., 2021 | [10.1097/MAO.0000000000003215](https://doi.org/10.1097/mao.0000000000003215) | No | Yes | Yes | Yes | Yes | No | Yes | Yes | Yes | Yes | Yes | Yes | 10 |
| Zhang Y et al., 2011 | [10.1016/j.jocn.2011.01.026](https://doi.org/10.1016/j.jocn.2011.01.026) | No | Yes | Yes | Yes | Yes | Yes | Yes | Yes | Yes | Yes | Yes | Yes | 11 |
| Zhao H et al. 2017 | [10.1016/j.wneu.2017.08.028](https://doi.org/10.1016/j.wneu.2017.08.028) | No | Yes | Yes | Yes | No | No | Yes | Yes | Yes | Yes | Yes | Yes | 9 |
| Zheng W.-J. Et al., 2018 | [10.1016/j.wneu.2018.08.172](https://doi.org/10.1016/j.wneu.2018.08.172) | Yes | Yes | Yes | Yes | Yes | No | Yes | Yes | Yes | Yes | Yes | Yes | 10 |
| Zhong C et al., 2023 | [10.12669/pjms.39.6.7963](https://doi.org/10.12669/pjms.39.6.7963) | No | Yes | Yes | Yes | Yes | No | Yes | Yes | Yes | Yes | Yes | Yes | 10 |
| Zhou H et al. 2013 | [10.1016/j.jcms.2013.01.006](https://doi.org/10.1016/j.jcms.2013.01.006) | No | Yes | Yes | Yes | Yes | Yes | Yes | Yes | No | No | Yes | Yes | 9 |
| Zhou Y et al., 2020 | [10.21037/atm.2020.03.221](https://doi.org/10.21037/atm.2020.03.221) | No | Yes | Yes | Yes | No | No | Yes | Yes | No | No | Yes | Yes | 7 |
| Zohdy YM et al., 2023 | [10.1016/j.wneu.2023.08.063](https://doi.org/10.1016/j.wneu.2023.08.063) | No | Yes | Yes | Yes | Yes | No | Yes | Yes | Yes | Yes | Yes | Yes | 10 |
