# Supplementary material for: Heterogeneity in definitions of surgical site infection after cranial surgery limits the validity of research findings in neurosurgery: a systematic review
Source: Neurosurg Rev. 2025 Jan 16;48(1):59. doi: 10.1007/s10143-025-03218-5 (PMC11739257; doi:10.1007/s10143-025-03218-5)
Supplement: Supplementary file 3 — Supplementary Material 3 [file 10143_2025_3218_MOESM3_ESM.docx]

Supplementary Table 6. Indicators used to define SSI-CRAN in each study that provided a definition for SSI (n = 163).

| Author and year published | DOI | Criteria applied for SSI | Number of indicators used | Pain | Temperature of wound/heat | Change in colour | Inflammation | Oedema | Pyrexia | Wound swab culture | Blood samples/ positive blood culture | Pus/ Purulent discharge | Leukocytosis | Leukopenia | Lethargy | Headache | Nausea and vomiting | CSF leak | Neurological changes | Positive CSF culture | Meningitis/ Ventriculitis | Brain abscess/ Empyema | Signs of infection on imaging | Wound dehiscence/ Wound reopened by surgeon | Fluid collection | Diagnosis made by surgeon or attending physician | Crusting over wound site | IV or oral antibiotic course outside of prophylactic regime | Subcutaneous tissue necrosis | Numberical values or cut off used for bacteria in swabs? |
| --- | --- | --- | --- | --- | --- | --- | --- | --- | --- | --- | --- | --- | --- | --- | --- | --- | --- | --- | --- | --- | --- | --- | --- | --- | --- | --- | --- | --- | --- | --- |
| Abode-Iyamah K.O. et al., 2018 | [10.3171/2016.12.JNS161967](https://doi.org/10.3171/2016.12.jns161967) | CDC guidelines but for a whole year follow-up | 15 | Yes | Yes | Yes (erythema) | Yes | Yes | Yes | Yes | Yes (positive culture) | Yes | No | No | No | No | No | No | No | Yes | Yes | Yes | Yes | Yes | No | Yes | No | No | No |  |
| Abode-lyamah K.O. et al., 2018 | [10.3171/2017.9.JNS1780](https://doi.org/10.3171/2017.9.jns1780) | CDC guidelines but for a whole year follow-up | 15 | Yes | Yes | Yes (erythema) | Yes | Yes | Yes | Yes | Yes (positive culture) | Yes | No | No | No | No | No | No | No | Yes | Yes | Yes | Yes | Yes | No | Yes | No | No | No |  |
| Abulhasan Y.B. et al., 2018 | [10.1016/j.wneu.2018.04.061](https://doi.org/10.1016/j.wneu.2018.04.061) | CDC guidelines but for a whole year follow-up | 15 | Yes | Yes | Yes (erythema) | Yes | Yes | Yes | Yes | Yes (positive culture) | Yes | No | No | No | No | No | No | No | Yes | Yes | Yes | Yes | Yes | No | Yes | No | No | No |  |
| Adeleye A., 2017 | [10.1080/02688697.2017.1407746](https://doi.org/10.1080/02688697.2017.1407746) | CDC guidelines but for a whole year follow-up | 15 | Yes | Yes | Yes (erythema) | Yes | Yes | Yes | Yes | Yes (positive culture) | Yes | No | No | No | No | No | No | No | Yes | Yes | Yes | Yes | Yes | No | Yes | No | No | No |  |
| Agarwal N. et al., 2017 | [10.1093/neuros/nyx273](https://doi.org/10.1093/neuros/nyx273) | CDC guidelines but for a whole year follow-up | 15 | Yes | Yes | Yes (erythema) | Yes | Yes | Yes | Yes | Yes (positive culture) | Yes | No | No | No | No | No | No | No | Yes | Yes | Yes | Yes | Yes | No | Yes | No | No | No |  |
| Ahmad N. et al., 2008 | [10.1097/SCS.0b013e31816ae358](https://doi.org/10.1097/scs.0b013e31816ae358) | "Signs of infection": tenderness, erythema, induration, purulent effluent. | 4 | Yes | No | Yes (erythema) | Yes | No | No | No | No | Yes | No | No | No | No | No | No | No | No | No | No | No | No | No | No | No | No | No |  |
| Al-Sharydah A.M. et al., 2020 | [10.15537/smj.2020.6.25095](https://doi.org/10.15537/smj.2020.6.25095) | “Positive microbiological culture within 120 hours after | 3 | No | No | No | No | No | Yes | Yes | Yes (positive culture) | No | No | No | No | No | No | No | No | No | No | No | No | No | No | No | No | No | No |  |
| Alkhaibary A. et al., 2019 | [10.1016/j.wneu.2019.09.120](https://doi.org/10.1016/j.wneu.2019.09.120) | Positive wound/tissue culture with clinical signs and symptoms of infection | 1 | No | No | No | No | No | No | Yes | No | No | No | No | No | No | No | No | No | No | No | No | No | No | No | No | No | No | No |  |
| Ammanuel S.G. et al., 2021 | [10.3171/2020.10.JNS201255](https://doi.org/10.3171/2020.10.jns201255) | CDC guidelines | 15 | Yes | Yes | Yes (erythema) | Yes | Yes | Yes | Yes | YES (positive culture) | Yes | No | No | No | No | No | No | No | Yes | Yes | Yes | Yes | Yes | No | Yes | No | No | No |  |
| Arocho-Quinones E.V. et al., 2019 | [10.1016/j.wneu.2019.04.003](https://doi.org/10.1016/j.wneu.2019.04.003) | CDC guidelines | 15 | Yes | Yes | Yes (erythema) | Yes | Yes | Yes | Yes | YES (positive culture) | Yes | No | No | No | No | No | No | No | Yes | Yes | Yes | Yes | Yes | No | Yes | No | No | No |  |
| Bekar A. et al., 2001 | [10.1007/s007010170057](https://doi.org/10.1007/s007010170057) | CDC guidelines | 15 | Yes | Yes | Yes (erythema) | Yes | Yes | Yes | Yes | Yes (positive culture) | Yes | No | No | No | No | No | No | No | Yes | Yes | Yes | Yes | Yes | No | Yes | No | No | No |  |
| Bhatti M. et al., 2012 | [10.3109/02688697.2012.743968](https://doi.org/10.3109/02688697.2012.743968) | CDC guidelines | 15 | Yes | Yes | Yes (erythema) | Yes | Yes | Yes | Yes | Yes (positive culture) | Yes | No | No | No | No | No | No | No | Yes | Yes | Yes | Yes | Yes | No | Yes | No | No | No |  |
| Bjerknes S. et al., 2014 | [10.1371/journal.pone.0105288](https://doi.org/10.1371/journal.pone.0105288) | CDC guidelines/Guideline for Prevention of SSI | 15 | Yes | Yes | Yes (erythema) | Yes | Yes | Yes | Yes | Yes (positive culture) | Yes | No | No | No | No | No | No | No | Yes | Yes | Yes | Yes | Yes | No | Yes | No | No | No |  |
| Buang S.S. et al., 2012 | PMID: 23082448 | CDC Guidelines/ National Healthcare Safety Network Surveillance Definitions | 15 | Yes | Yes | Yes (erythema) | Yes | Yes | Yes | Yes | Yes (positive culture) | Yes | No | No | No | No | No | No | No | Yes | Yes | Yes | Yes | Yes | No | Yes | No | No | No |  |
| Cacciola F. et al., 2001 | [10.1179/joc.2001.13.Supplement-2.119](https://doi.org/10.1179/joc.2001.13.supplement-2.119) | CDC guidelines | 15 | Yes | Yes | Yes (erythema) | Yes | Yes | Yes | Yes | YES (positive culture) | Yes | No | No | No | No | No | No | No | Yes | Yes | Yes | Yes | Yes | No | Yes | No | No | No |  |
| Campioli C. et al., 2022 | [10.1017/ash.2021.258](https://doi.org/10.1017/ash.2021.258) | CDC guidelines | 15 | Yes | Yes | Yes (erythema) | Yes | Yes | Yes | Yes | Yes (positive culture) | Yes | No | No | No | No | No | No | No | Yes | Yes | Yes | Yes | Yes | No | Yes | No | No | No |  |
| Carlson J.D. et al., 2019 | [10.1016/j.wneu.2019.04.233](https://doi.org/10.1016/j.wneu.2019.04.233) | "Purulent discharge from a wound that required surgical treatment" | 2 | No | No | No | No | No | No | No | No | Yes | No | No | No | No | No | No | No | No | No | No | No | Yes | No | No | No | No | No |  |
| Catapano J. et al., 2019 | [10.1016/j.wneu.2019.07.183](https://doi.org/10.1016/j.wneu.2019.07.183) | An EVD-related infection was defined as a “CSF culture with positive results”. | 1 | No | No | No | No | No | No | No | No | No | No | No | No | No | No | No | No | YES | No | No | No | No | No | No | No | No | No |  |
| Chaichana K.L. et al., 2015 | [10.1179/1743132815Y.0000000042](https://doi.org/10.1179/1743132815y.0000000042) | CDC guidelines | 15 | Yes | Yes | Yes (erythema) | Yes | Yes | Yes | Yes | Yes (positive culture) | Yes | No | No | No | No | No | No | No | Yes | Yes | Yes | Yes | Yes | No | Yes | No | No | No |  |
| Cheah PP et al., 2017 | [10.21315/mjms2017.24.6.8](https://doi.org/10.21315/mjms2017.24.6.8) | CDC guidelines | 15 | Yes | Yes | Yes (erythema) | Yes | Yes | Yes | Yes | YES (positive culture) | Yes | No | No | No | No | No | No | No | Yes | Yes | Yes | Yes | Yes | No | Yes | No | No | No |  |
| Chen C. et al., 2016 | 10.1097/MD.0000000000004329 | CDC guidelines/ National Healthcare Safety Network Surveillance Definitions | 15 | Yes | Yes | Yes (erythema) | Yes | Yes | Yes | Yes | Yes (positive culture) | Yes | No | No | No | No | No | No | No | Yes | Yes | Yes | Yes | Yes | No | Yes | No | No | No |  |
| Cheng C.H. et al., 2014 | [10.1016/j.clineuro.2014.06.029](https://doi.org/10.1016/j.clineuro.2014.06.029) | Defined by presence of focal erythema, pus-like discharge or wound rupture | 3 | No | No | Yes (erythema) | No | No | No | No | No | Yes | No | No | No | No | No | No | No | No | No | No | No | Yes | No | No | No | No | No |  |
| Chiang H.Y. et al., 2011 | [10.3171/2011.1.JNS10782](https://doi.org/10.3171/2011.1.jns10782) | CDC guidelines | 15 | Yes | Yes | Yes (erythema) | Yes | Yes | Yes | Yes | YES (positive culture) | Yes | No | No | No | No | No | No | No | Yes | Yes | Yes | Yes | Yes | No | Yes | No | No | No |  |
| Colombo F. et al., 2023 | [10.1055/s-0043-1774720](https://doi.org/10.1055/s-0043-1774720) | Defined as per Section 3 of the Protocol for Surveillance of SSI published by Public Health England. | 13 | Yes | Yes | Yes (erythema) | Yes | Yes | Yes | Yes | No | Yes | No | No | No | No | No | No | No | Yes | No | Yes | Yes | Yes | No | Yes | No | No | No |  |
| Cosgrove G.R. et al., 2007 | [10.3171/jns.2007.106.1.52](https://doi.org/10.3171/jns.2007.106.1.52) | CDC guidelines | 15 | Yes | Yes | Yes (erythema) | Yes | Yes | Yes | Yes | Yes (positive culture) | Yes | No | No | No | No | No | No | No | Yes | Yes | Yes | Yes | Yes | No | Yes | No | No | No |  |
| Coulter I.C. et al., 2014 | [10.1007/s00701-014-2081-1](https://doi.org/10.1007/s00701-014-2081-1) | Infection was defined by the need for antibiotics and the need to remove an infected implant | 2 | No | No | No | No | No | No | No | No | No | No | No | No | No | No | No | No | No | No | No | No | Yes | No | No | No | Yes | No |  |
| Davies B.M. et al., 2016 | [10.1308/rcsann.2016.0143](https://doi.org/10.1308/rcsann.2016.0143) | UK Health Protection Agency guidance | 2 | No | No | No | No | No | No | Yes | No | Yes | No | No | No | No | No | No | No | No | No | No | No | No | No | No | No | No | No |  |
| Davies B.M. et al., 2015 | [10.3109/02688697.2015.1071321](https://doi.org/10.3109/02688697.2015.1071321) | Public Health England Guidelines | 11 | Yes | Yes | Yes (erythema) | Yes | Yes | Yes | Yes | No | Yes | No | No | No | No | No | No | No | Yes | No | Yes | Yes | Yes | No | Yes | No | No | No |  |
| Dinevski N. et al., 2017 | [10.1016/j.wneu.2017.03.093](https://doi.org/10.1016/j.wneu.2017.03.093) | Centre of Disease Control and Prevention Guidelines – with an altered follow-up period | 15 | Yes | Yes | Yes (erythema) | Yes | Yes | Yes | Yes | Yes (positive culture) | Yes | No | No | No | No | No | No | No | Yes | Yes | Yes | Yes | Yes | No | Yes | No | No | No |  |
| Ellens N.R. et al., 2019 | [0.1093/neuros/nyy090](https://doi.org/10.1093/neuros/nyy090) | Infection defined by positive CSF culture | 1 | No | No | No | No | No | No | No | No | No | No | No | No | No | No | No | No | Yes | No | No | No | No | No | No | No | No | No |  |
| Elward A. et al., 2015 | [10.1097/INF.0000000000000889](https://doi.org/10.1097/inf.0000000000000889) | CDC guidelines/ National Healthcare Safety Network Surveillance Definitions | 15 | Yes | Yes | Yes (erythema) | Yes | Yes | Yes | Yes | Yes (positive culture) | Yes | No | No | No | No | No | No | No | Yes | Yes | Yes | Yes | Yes | No | Yes | No | No | No |  |
| Fan M.C. et al., 2018 | [10.1016/j.wneu.2017.10.112](https://doi.org/10.1016/j.wneu.2017.10.112) | 1. Any case in which infection was suspected and antibiotics therapy was administered 2. Requirement of reoperation to remove graft. | 2 | No | No | No | No | No | No | No | No | No | No | No | No | No | No | No | No | No | No | No | Yes | No | No | No | No | Yes | No |  |
| Farber S.H. et al., 2011 | [10.1227/NEU.0b013e31821bc435](https://doi.org/10.1227/neu.0b013e31821bc435) | Clinical suspicion of shunt infection (fever, change in mental status, increased white blood cell count, signs of meningismus), the presence of a CSF profile (leukocytosis, positive CSF bacterial cultures) | 5 | No | No | No | No | No | Yes | Yes (positive bacterial culture) | Yes (increased WBC count) | No | No | No | No | No | No | No | Yes | No | Yes (meningismus) | No | No | No | No | No | No | No | No |  |
| Farrokhi F.R. et al., 2019 | [10.1016/j.jocn.2019.08.026](https://doi.org/10.1016/j.jocn.2019.08.026) | 1) Requiring surgical removal of DBS hardware | 1 | No | No | No | No | No | No | No | No | No | No | No | No | No | No | No | No | No | No | No | No | YES | No | No | No | No | No |  |
| Fenoy A.J. et al., 2012 | [10.3171/2012.1.JNS111798](https://doi.org/10.3171/2012.1.jns111798) | 1. Within 12 months of an original implantation 2. Superficial wound infections: induration, redness, persistent crusting over a hardware component, cellulitis,  purulent drainage. 3. requiring surgical revision  4. cultures from hardware or from fluid in contact with hardware. | 7 | No | No | Yes (erythema) | Yes | No | No | Yes | No | Yes | No | No | No | No | No | No | No | Yes | No | No | No | No | No | No | Yes | No | No |  |
| Fialkov J.A. et al., 2001 | [10.1097/00001665-200107000-00009](https://doi.org/10.1097/00001665-200107000-00009) | Clinical diagnosis (two or more signs of local infection: redness, swelling, purulent discharge) plus one or more of the following: 1. Hospital admission for treatment of infection. 2. Intravenous or oral antibiotic treatment. 3. Surgical intervention for drainage, irrigation, and/or debridement. 4. Microbiological confirmation (pathogenic organism present in 10>5 cfu/ml). | 8 | No | No | Yes (erythema) | Yes | Yes | No | Yes (microbiological confirmation (pathogenic organism present in <10>5 cfu/ml)) | No | Yes | No | No | No | No | No | No | No | No | No | No | No | Yes | No | Yes | No | Yes | No | Microbiological confirmation (pathogenic organism present in 10>5 cfu/ml). |
| Frizon L.A. et al., 2017 | [10.1111/ner.12605](https://doi.org/10.1111/ner.12605) | 1. Wound tenderness or purulent discharge 2. Elevated temperature or inflammatory markers 3. Evidence of hardware exposure at the pulse generator site (i.e., erosion) | 6 | Yes | No | No | Yes | No | Yes | No | Yes (inflammatory markers) | Yes | No | No | No | No | No | No | No | No | No | No | No | Yes | No | No | No | No | No |  |
| Gil Z. et al., 2003 | [10.1067/mhn.2003.14](https://doi.org/10.1067/mhn.2003.14) | CDC guidelines | 15 | Yes | Yes | Yes (erythema) | Yes | Yes | Yes | Yes | Yes (positive culture) | Yes | No | No | No | No | No | No | No | Yes | Yes | Yes | Yes | Yes | No | Yes | No | No | No |  |
| Girgis F. et al., 2015 | [10.1017/cjn.2015.46](https://doi.org/10.1017/cjn.2015.46) | 1. Evidence of frank purulence as described in operative records  2. the presence of positive intra-operative cultures from a non-superficial source (e.g. subcutaneous tissue, bone, epidural, subdural, intracranial collections.) | 3 | No | No | No | No | No | No | Yes | No | Yes | No | No | No | No | No | No | No | Yes | No | No | No | No | No | No | No | No | No |  |
| Gorgulho A. et al., 2009 | [10.3171/2008.6.17603](https://doi.org/10.3171/2008.6.17603) | Guideline for Prevention of Surgical Site Infection | 10 | Yes | No | Yes (erythema) | Yes | Yes | Yes | Yes | No | Yes | No | No | No | No | No | No | No | Yes | No | No | Yes | Yes | No | No | No | No | No |  |
| Govindaswamy A. et al., 2022 | [10.4103/ajns.AJNS_268_18](https://doi.org/10.4103/ajns.ajns_268_18) | CDC guidelines | 15 | Yes | Yes | Yes (erythema) | Yes | Yes | Yes | Yes | Yes (positive culture) | Yes | No | No | No | No | No | No | No | Yes | Yes | Yes | Yes | Yes | No | Yes | No | No | No |  |
| Gruenbaum S. et al., 2017 | [10.1213/ANE.0000000000001946](https://doi.org/10.1213/ane.0000000000001946) | CDC guidelines | 15 | Yes | Yes | Yes (erythema) | Yes | Yes | Yes | Yes | YES (positive culture) | Yes | No | No | No | No | No | No | No | Yes | Yes | Yes | Yes | Yes | No | Yes | No | No | No |  |
| Grundy T et al. 2019 | [10.1080/02688697.2019.1645298](https://doi.org/10.1080/02688697.2019.1645298) | CDC guidelines | 15 | Yes | Yes | Yes (erythema) | Yes | Yes | Yes | Yes | Yes (positive culture) | Yes | No | No | No | No | No | No | No | Yes | Yes | Yes | Yes | Yes | No | Yes | No | No | No |  |
| Hale A.T. et al., 2020 | [10.3171/2019.9.PEDS1939](https://doi.org/10.3171/2019.9.peds1939) | Requiring debridement within 180 days following surgery | 1 | No | No | No | No | No | No | No | No | No | No | No | No | No | No | No | No | No | No | No | No | Yes | No | No | No | No | No |  |
| Halpern C.H. et al., 2012 | [10.1016/j.ajic.2011.06.005](https://doi.org/10.1016/j.ajic.2011.06.005) | Swelling, redness, pain, warmth, drainage, or fluid collection involving the DBS system or the skin incision. | 7 | Yes | Yes | Yes (erythema) | Yes | Yes | No | No | No | Yes | No | No | No | No | No | No | No | No | No | No | No | No | Yes | No | No | No | No |  |
| Hamdeh S.A. et al., 2014 | [10.3109/02688697.2013.835376](https://doi.org/10.3109/02688697.2013.835376) | CDC guidelines | 15 | Yes | Yes | Yes (erythema) | Yes | Yes | Yes | Yes | Yes (positive culture) | Yes | No | No | No | No | No | No | No | Yes | Yes | Yes | Yes | Yes | No | Yes | No | No | No |  |
| Hardaway F.A. et al., 2017 | [10.1093/neuros/nyx505](https://doi.org/10.1093/neuros/nyx505) | Guideline for Prevention of Surgical Site Infection | 9 | Yes | No | Yes (erythema) | Yes | No | Yes | Yes | No | Yes | No | No | No | No | No | No | No | Yes | No | Yes | Yes | No | No | No | No | No | No |  |
| Hardy S. et al., 2010 | [10.3171/2010.2.JNS09950](https://doi.org/10.3171/2010.2.jns09950) | CDC guidelines | 15 | Yes | Yes | Yes (erythema) | Yes | Yes | Yes | Yes | Yes (positive culture) | Yes | No | No | No | No | No | No | No | Yes | Yes | Yes | Yes | Yes | No | Yes | No | No | No |  |
| Harrop J.S. et al., 2010 | [10.1227/01.NEU.0000370247.11479.B6](https://doi.org/10.1227/01.neu.0000370247.11479.b6) | 1. Two positive CSF cultures from ventriculostomy catheters.  2. Increase in cerebrospinal fluid white blood cell count. | 1 | No | No | No | No | No | No | No | No | No | No | No | No | No | No | No | No | Yes | No | No | No | No | No | No | No | No | No |  |
| Hasegawa H. et al., 2021 | [10.3171/2020.7.JNS201385](https://doi.org/10.3171/2020.7.jns201385) | If a patient had undergone VNS-related procedures and developed one or more signs:  1. Purulence  2. Positive culture 3. Superficial signs: wound dehiscence, swelling, drainage, and redness. | 7 | No | No | Yes | Yes | Yes | No | Yes | Yes (positive culture) | Yes | No | No | No | No | No | No | No | No | No | No | No | Yes | No | No | No | No | No |  |
| Hayashi T. et al., 2010 | [10.3171/2010.5.PEDS1018](https://doi.org/10.3171/2010.5.peds1018) | 1. Positive CSF- or catheter-based cultures  2. At least 1 clinical component of infection: headache, meningitis, fever, elevated peripheral leukocyte count, elevated C-reactive protein 3. CSF white blood cell count > 40/mm3  4. obvious cellulites at the operative site  5. exposure of the shunt hardware through an open incision | 3 | No | No | No | No | No | Yes | No | YES (positive culture) | No | Yes | No | No | No | No | No | No | Yes | No | No | No | No | No | No | No | No | No |  |
| Ho A. et al., 2018 | [10.3171/2018.5.PEDS17719](https://doi.org/10.3171/2018.5.peds17719) | CDC guidelines | 15 | Yes | Yes | Yes (erythema) | Yes | Yes | Yes | Yes | Yes (positive culture) | Yes | No | No | No | No | No | No | No | Yes | Yes | Yes | Yes | Yes | No | Yes | No | No | No |  |
| Hoang T. et al., 2023 | [10.1017/ice.2022.112](https://doi.org/10.1017/ice.2022.112) | CDC guidelines | 15 | Yes | Yes | Yes (erythema) | Yes | Yes | Yes | Yes | Yes (positive culture) | Yes | No | No | No | No | No | No | No | Yes | Yes | Yes | Yes | Yes | No | Yes | No | No | No |  |
| Honeybul S. et al., 2012 | [10.1097/PRS.0b013e318267d4de](https://doi.org/10.1097/prs.0b013e318267d4de) | Infection requiring removal of autologous bone in patients who survived for at least one year after the initial cranioplasty. | 1 | No | No | No | No | No | No | No | No | No | No | No | No | No | No | No | No | No | No | No | No | No | No | Yes | No | No | No |  |
| Hu H. et al., 2022 | [10.1016/j.jgar.2023.08.006](https://doi.org/10.1016/j.jgar.2023.08.006) | 1. Positive CSF culture after neurosurgery  2. CSF leukocyte count > 100 × 106/L, neutrophils ratio > 70%, and glucose levels < 2.2 mmol/L | 1 | No | No | No | No | No | No | No | No | No | No | No | No | No | No | No | No | Yes | No | No | No | No | No | No | No | No | No |  |
| Huang Y. et al., 2011 | [10.1016/j.injury.2011.11.005](https://doi.org/10.1016/j.injury.2011.11.005) | Less than 14 days after cranioplasty: 1. Pus or infected fluid in the subgaleal layer with or without involvement of epidural and subdural spaces 2. Extensive infection necessitating the removal of the bone flap  3. CT scans indicating infection  4. Requirement of surgical debridement  5. Abscess cultures and swab cultures from skull flaps. | 5 | No | No | No | No | No | No | Yes | No | Yes | No | No | No | No | No | No | No | No | No | Yes | Yes | Yes | No | No | No | No | No |  |
| Huang Y.H. et al., 2011 | [10.1097/TA.0b013e318203208a](https://doi.org/10.1097/ta.0b013e318203208a) | CDC guidelines | 16 | Yes | Yes | Yes (erythema) | Yes | Yes | Yes | Yes | Yes (positive culture) | Yes | No | No | No | No | No | No | No | Yes | Yes | Yes | Yes | Yes | No | Yes | No | Yes | No |  |
| Im S.H. et al., 2012 | [10.3340/jkns.2012.52.4.396](https://doi.org/10.3340/jkns.2012.52.4.396) | 1. Prescription for antibiotics more than 2 weeks after cranioplasty without other organ infection  2. Subcutaneous or subgaleal abscess 3. Radiologic records related to subcutaneous, subgaleal, epidural or subdural empyema 4) Requirement of bone flap removal or wound revision and irrigation | 4 | No | No | No | No | No | No | No | No | No | No | No | No | No | No | No | No | No | No | Yes | Yes | No | Yes | No | No | Yes | No |  |
| Jeong T.S. et al., 2018 | [10.3340/jkns.2018.0021](https://doi.org/10.3340/jkns.2018.0021) | CDC guidelines | 15 | Yes | Yes | Yes (erythema) | Yes | Yes | Yes | Yes | Yes (positive culture) | Yes | No | No | No | No | No | No | No | Yes | Yes | Yes | Yes | Yes | No | Yes | No | No | No |  |
| Jeong T.S. et al., 2020 | [10.1371/journal.pone.0232561](https://doi.org/10.1371/journal.pone.0232561) | CDC guidelines | 15 | Yes | Yes | Yes (erythema) | Yes | Yes | Yes | Yes | YES (positive culture) | Yes | No | No | No | No | No | No | No | Yes | Yes | Yes | Yes | Yes | No | Yes | No | No | No |  |
| Jiang X. et al., 2014 | [10.5137/1019-5149.JTN.12738-14.0](https://doi.org/10.5137/1019-5149.jtn.12738-14.0) | CDC guidelines | 15 | Yes | Yes | Yes (erythema) | Yes | Yes | Yes | Yes | Yes (positive culture) | Yes | No | No | No | No | No | No | No | Yes | Yes | Yes | Yes | Yes | No | Yes | No | No | No |  |
| Jimenez-Martinez E. et al., 2019 | [10.1186/s13756-019-0525-3](https://doi.org/10.1186/s13756-019-0525-3) | CDC guidelines | 15 | Yes | Yes | Yes (erythema) | Yes | Yes | Yes | Yes | Yes (positive culture) | Yes | No | No | No | No | No | No | No | Yes | Yes | Yes | Yes | Yes | No | Yes | No | No | No |  |
| Jimenez-Martinez E. et al., 2021 | [10.1093/cid/ciaa884](https://doi.org/10.1093/cid/ciaa884) | CDC guidelines | 15 | Yes | Yes | Yes (erythema) | Yes | Yes | Yes | Yes | Yes (positive culture) | Yes | No | No | No | No | No | No | No | Yes | Yes | Yes | Yes | Yes | No | Yes | No | No | No |  |
| Jimenez-Martinez E. et al., 2021 | [10.1186/s13756-021-01016-4](https://doi.org/10.1186/s13756-021-01016-4) | CDC guidelines | 15 | Yes | Yes | Yes (erythema) | Yes | Yes | Yes | Yes | YES (positive culture) | Yes | No | No | No | No | No | No | No | Yes | Yes | Yes | Yes | Yes | No | Yes | No | No | No |  |
| Joerger A. et al., 2023 | [10.1007/s00701-023-05870-6](https://doi.org/10.1007/s00701-023-05870-6) | Classified as superficial, deep, epidural and intracranial (empyema, abscess), meningitis/ ventriculitis, infected cerebrospinal fluid (CSF) fistula and shunt infection. | 3 | No | No | No | No | No | No | No | No | No | No | No | No | No | No | No | No | Yes | Yes | Yes | No | No | No | No | No | No | No |  |
| Kalangu K. et al., 2020 | [10.1007/s00381-019-04357-z](https://doi.org/10.1007/s00381-019-04357-z) | CDC guidelines | 15 | Yes | Yes | Yes (erythema) | Yes | Yes | Yes | Yes | Yes (positive culture) | Yes | No | No | No | No | No | No | No | Yes | Yes | Yes | Yes | Yes | No | Yes | No | No | No |  |
| Kim M.J. et al., 2021 | [10.3389/fneur.2021.745575](https://doi.org/10.3389/fneur.2021.745575) | 1. Requiring removal of bone flap or implant due to purulent discharge with signs of infection and complicated fluid collection, empyema, or abscess on CT scans. 2. Wound dehiscence with flap exposure requiring surgical revision without removal of bone flap or implant. | 5 | No | No | No | No | No | No | No | No | Yes | No | No | No | No | No | No | No | No | No | Yes | Yes | Yes | Yes | No | No | No | No |  |
| Kim T et al. 2013 | [10.1007/s00701-013-1833-7](https://doi.org/10.1007/s00701-013-1833-7) | CDC guidelines | 15 | Yes | Yes | Yes (erythema) | Yes | Yes | Yes | Yes | Yes (positive culture) | Yes | No | No | No | No | No | No | No | Yes | Yes | Yes | Yes | Yes | No | Yes | No | No | No |  |
| Kinaci A. et al., 2023 | [10.1227/neu.0000000000002345](https://doi.org/10.1227/neu.0000000000002345) | Wound infection was subdivided into 2 categories:  1. Superficial infection requiring only antibiotics 2. Deep wound infection requiring revision surgery. Meningitis was defined as clinical suspicion for meningitis in combination with antibiotic treatment. | 3 | No | No | No | No | No | No | No | No | No | No | No | No | No | No | No | No | No | Yes | No | No | Yes | No | No | No | Yes | No |  |
| Klieverik V.M. et al., 2023 | [10.1016/j.wneu.2023.04.008](https://doi.org/10.1016/j.wneu.2023.04.008) | A culture-positive wound swab or underlying fluid tap requiring surgical removal of the implant and antibiotic therapy. | 4 | No | No | No | No | No | No | Yes | No | No | No | No | No | No | No | No | No | Yes | No | No | No | Yes | No | No | No | Yes | No |  |
| Kochanski R. et al., 2018 | 10.1093/ons/opx293 | 1. Fever, redness, warmth, tenderness, or drainage resulting in antibiotic therapy.  2. White blood cell count, C reactive protein levels, and estimated sedimentation rate  3. Positive wound cultures 4. Device explantation | 9 | Yes | Yes | Yes (erythema) | Yes | No | Yes | Yes | No | Yes | No | No | No | No | No | No | No | No | No | No | No | Yes | No | No | No | Yes | No |  |
| Kogeichi Y. et al., 2022 | [10.1016/j.inat.2022.101489](https://doi.org/10.1016/j.inat.2022.101489) | Local inflammation that required surgical removal of the bone flap. | 2 | No | No | No | Yes | No | No | No | No | No | No | No | No | No | No | No | No | No | No | No | No | Yes | No | No | No | No | No |  |
| Kondapavulur S. et al., 2022 | [10.1159/000520197](https://doi.org/10.1159/000520197) | Infection of hardware within 6 months of implantation surgery, requiring partial, or complete hardware removal, with a positive culture. | 2 | No | No | No | No | No | No | Yes | No | No | No | No | No | No | No | No | No | No | No | No | No | Yes | No | No | No | No | No |  |
| Korinek A.M. et al., 2005 | [10.1080/02688690500145639](https://doi.org/10.1080/02688690500145639) | CDC guidelines | 16 | Yes | Yes | Yes (erythema) | Yes | Yes | Yes | Yes | Yes (positive culture) | Yes | No | No | No | No | No | No | No | Yes | Yes | Yes | Yes | Yes | No | Yes | No | Yes | No |  |
| Kose G. et al., 2015 | [10.1111/jocn.13149](https://doi.org/10.1111/jocn.13149) | CDC guidelines/ National Healthcare Safety Network Surveillance Definitions | 15 | Yes | Yes | Yes (erythema) | Yes | Yes | Yes | Yes | Yes (positive culture) | Yes | No | No | No | No | No | No | No | Yes | Yes | Yes | Yes | Yes | No | Yes | No | No | No |  |
| Krafft P. et al., 2022 | 10.1016/j.clineuro.2022.107206 | Clinical findings and symptoms meriting antibiotics treatment or re-operation for wound exploration and revision. | 2 | No | No | No | No | No | No | No | No | No | No | No | No | No | No | No | No | No | No | No | No | Yes | No | No | No | Yes | No |  |
| Kraus D. et al., 2005 | 10.1097/01.mlg.0000172201.61487.69 | Major infectious complications: abscess, osteomyelitis, frontal bone flap infection, meningitis, deep soft tissue infection, subdural empyema, and C. dificile enterocolitis. Minor wound infections: cellulitis, hardware infection, donor site infection, and dacryocystitis. Culture results were also used. | 4 | No | No | No | Yes | No | No | Yes | No | No | No | No | No | No | No | No | No | No | Yes | Yes | No | No | No | No | No | No | No |  |
| Kuwano A. et al., 2023 | 10.1007/s00701-022-05474-6 | Requirement of reoperation. SSIs were suspected on the basis of the postoperative wound conditions and MRI findings. | 2 | No | No | No | No | No | No | No | No | No | No | No | No | No | No | No | No | No | No | No | Yes | Yes | No | No | No | No | No |  |
| Lee C.H. et al., 2012 | [10.1097/TA.0b013e318256a150](https://doi.org/10.1097/ta.0b013e318256a150) | Requirement the removal of the infected bone graft. | 1 | No | No | No | No | No | No | No | No | No | No | No | No | No | No | No | No | No | No | No | No | Yes | No | No | No | No | No |  |
| Lee J.K. et al., 2012 | [10.3346/jkms.2012.27.12.1563](https://doi.org/10.3346/jkms.2012.27.12.1563) | 1. Identification of a bacterial pathogen from the reservoir CSF/ reservoir CSF pleocytosis (more than 50 leukocytes per cubic millimetre)  2. Positive blood culture  3. Fever 4. Neurologic symptoms 5. Abdominal symptoms 6. Shunt malfunction | 4 | No | No | No | No | No | Yes | No | YES (positive culture) | No | No | No | No | No | No | Yes | Yes | Yes | No | No | No | No | No | No | No | No | No |  |
| Lefebvre J. et al., 2017 | [10.1016/j.jhin.2016.11.019](https://doi.org/10.1016/j.jhin.2016.11.019) | CDC guidelines | 15 | Yes | Yes | Yes (erythema) | Yes | Yes | Yes | Yes | Yes (positive culture) | Yes | No | No | No | No | No | No | No | Yes | Yes | Yes | Yes | Yes | No | Yes | No | No | No |  |
| Lepski G. et al., 2021 | 10.1016/j.clineuro.2021.106599 | *Within 30 days after surgery, divided into 3 major categories:  1. Superficial/ wound infections 2. Meningitis  3. Deep/ intracranial (empyema or intracerebral abscess). Meningitis was confirmed if the patient had a confirmed or probable diagnosis.* | 4 | No | No | No | No | No | No | No | No | YES | No | No | No | No | No | No | No | YES | YES | YES | No | No | No | No | No | No | No |  |
| Leung G.K. et al., 2007 | 10.1080/02688690701392881 | Positive CSF culture | 1 | No | No | No | No | No | No | No | No | No | No | No | No | No | No | No | No | Yes | No | No | No | No | No | No | No | No | No |  |
| Levi V. et al., 2020 | 10.1093/ons/opz118 | Guideline for Prevention of Surgical Site Infection | 8 | Yes | No | Yes (erythema) | Yes | No | Yes | Yes | No | Yes | No | No | No | No | No | No | No | Yes | No | No | Yes | Yes | No | No | No | No | No |  |
| Lieber B.A. et al., 2016 | 10.3171/2015.4.JNS142719 | CDC guidelines | 15 | Yes | Yes | Yes (erythema) | Yes | Yes | Yes | Yes | Yes (positive culture) | Yes | No | No | No | No | No | No | No | Yes | Yes | Yes | Yes | Yes | No | Yes | No | No | No |  |
| Linzey J.R. et al., 2017 | 10.1093/neuros/nyx046 | 1. Erythema,  2. Purulent discharge  3. Tenderness,  4. Fever and other common signs of infection  5.Prescription of antibiotics for a wound infection | 6 | Yes | No | Yes (erythema) | No | No | Yes | No | No | Yes | No | No | No | No | No | No | No | No | No | No | No | No | No | Yes | No | Yes | No |  |
| Liu W. et al., 2021 | 10.1055/s-0040-1719138 | Wound redness, pain, suppuration, or subcutaneous tissue necrosis at the surgical site. | 4 | Yes | No | Yes (erythema) | No | No | No | No | No | Yes | No | No | No | No | No | No | No | No | No | No | No | No | No | No | No | No | Yes |  |
| Luther E. et al., 2020 | 10.1007/s00701-020-04239-3 | 1. Wound breakdown 2. CSF leak requiring readmission for antibiotics or reoperation. | 3 | No | No | No | No | No | No | No | No | No | No | No | No | No | No | Yes | No | Yes | No | No | No | Yes | No | No | No | No | No |  |
| Lv Y. et al., 2023 | 10.1186/s41016-023-00336-1 | A purulent discharge requiring local debridement or osteomyelitis/ abscess requiring reoperation, as well as positive cultures from secretion samples or CSF. | 5 | No | No | No | No | No | No | Yes | No | Yes | No | No | No | No | No | No | No | Yes | No | Yes | No | Yes | No | No | No | No | No |  |
| Lwin S. et al., 2012 | PMID: 22511048 | Positive CSF culture | 1 | No | No | No | No | No | No | No | No | No | No | No | No | No | No | No | No | Yes | No | No | No | No | No | No | No | No | No |  |
| Maayan O. et al., 2022 | 10.1007/s00701-021-05075-9 | Positive bacterial culture | 1 | No | No | No | No | No | No | Yes | No | No | No | No | No | No | No | No | No | No | No | No | No | No | No | No | No | No | No |  |
| Mallela A. et al., 2017 | 10.1093/neuros/nyx559 | Positive culture within 120 days of surgery. | 1 | No | No | No | No | No | No | Yes | No | No | No | No | No | No | No | No | No | No | No | No | No | No | No | No | No | No | No |  |
| Maye H. L. et al., 2022 | 10.1016/j.wneu.2022.02.124 | 1. Skin and soft-tissue infection,  2. Bone flap osteomyelitis,  3. Subdural empyema,  4. Brain abscess or meningitis, at either 30 days (early) or 4 months (delayed). | 3 | No | No | No | No | No | No | No | No | Yes | No | No | No | No | No | No | No | No | Yes | Yes | No | No | No | No | No | No | No |  |
| McGirt M.J. et al., 2003 | 10.1086/368191 | National Nosocomial Infection Surveillance System: CSF culture that yielded a pathogenic organism or indicated CSF pleocytosis (150 leukocytes/mm3 ) associated with fever (temperature, >38.5C), shunt malfunction, or neurological symptoms. | 16 | Yes | Yes | Yes (erythema) | Yes | Yes | Yes | Yes | Yes (positive culture) | Yes | No | No | No | No | No | No | Yes | Yes | Yes | Yes | Yes | Yes | No | Yes | No | No | No |  |
| Meng Y. et al., 2018 | 10.3171/2018.1.PEDS17476 | Any postoperative infection involving the wound, skull, or central nervous system (meningitis, epidural abscess, osteomyelitis). | 2 | No | No | No | No | No | No | No | No | No | No | No | No | No | No | No | No | No | Yes | Yes | No | No | No | No | No | No | No |  |
| Mian S.Y. et al., 2023 | 10.1016/j.wneu.2023.06.091 | 1. Clinical signs of infection, such as fever, meningism, or altered conscious level, WITH  2. Raised serum inflammatory markers (raised CRP or raised white cell count) PLUS 5. A positive CSF culture  6. Positive CSF gram stain  7. Raised CSF white cell count | 4 | No | No | No | No | No | Yes | No | Yes (CRP) | No | Yes | No | No | No | No | No | Yes | Yes | No | No | No | No | No | No | No | No | No |  |
| Miller J.J. et al., 2001 | 10.1097/00129492-200111000-00033 | Erythema, purulent discharge, fluid collection | 4 | No | No | Yes (erythema) | No | No | No | No | No | Yes | No | No | No | No | No | No | No | No | No | No | No | No | Yes | Yes | No | No | No |  |
| Mohamad S. et al., 2016 | 10.21315/mjms2016.23.5.11 | CDC guidelines | 15 | Yes | Yes | Yes (erythema) | Yes | Yes | Yes | Yes | Yes (positive culture) | Yes | No | No | No | No | No | No | No | Yes | Yes | Yes | Yes | Yes | No | Yes | No | No | No |  |
| Muram S. et al., 2023 | 10.3171/2022.5.JNS22430 | Canadian Nosocomial Infection Surveillance Program (CNISP) guidelines | 4 | No | No | No | No | No | Yes | No | No | No | No | No | No | No | Yes | No | Yes | Yes | No | No | No | No | No | No | No | No | No |  |
| Nair S.K. et al., 2023 | 10.3171/2022.8.JNS212799 | Wound breakdown, the presence of bacteria in wound drainage, or the need for reoperation for wound washout. | 3 | No | No | No | No | No | No | Yes | No | Yes | No | No | No | No | No | No | No | No | No | No | No | Yes | No | No | No | No | No |  |
| Nguyen A.V. et al., 2019 | 10.1016/j.clineuro.2019.05.017 | Positive culture isolated from the wound, implant, or CSF within a year of surgery. | 2 | No | No | No | No | No | No | Yes | YES (positive culture) | No | No | No | No | No | No | No | No | Yes | No | No | No | No | No | No | No | No | No |  |
| Nusair A.R. et al., 2021 | 10.1089/sur.2020.020 | CDC guidelines | 15 | Yes | Yes | Yes (erythema) | Yes | Yes | Yes | Yes | YES (positive culture) | Yes | No | No | No | No | No | No | No | Yes | Yes | Yes | Yes | Yes | No | Yes | No | No | No |  |
| O'Keeffe A. et al., 2012 | 10.3109/02688697.2011.626878 | CDC guidelines | 15 | Yes | Yes | Yes (erythema) | Yes | Yes | Yes | Yes | Yes (positive culture) | Yes | No | No | No | No | No | No | No | Yes | Yes | Yes | Yes | Yes | No | Yes | No | No | No |  |
| Oh W.O. et al., 2018 | 10.1159/000481437 | 1. Discharge,  2. Tenderness, 3. Swelling,  4. Fluid collection,  5. Redness of the surgical site | 5 | Yes | No | Yes (erythema) | Yes | Yes | No | No | No | Yes | No | No | No | No | No | No | No | No | No | No | No | No | No | No | No | No | No |  |
| Okunlola A.I. et al., 2021 | 10.1080/02688697.2020.1812518 | Grade 0: normal healing;  Grade I: normal healing with mild erythema or epidermolysis;  Grade II: superficial wound infection with galeal/ fascia intact;  Grade III: deep wound infection below the galeal/ fascia but with intact dural  IIIa: no osteomyelitis,  IIIb: with osteomyelitis and  IIIc: with pachy meningitis;  Grade IV: meningitis without tissue breakdown excluding chemical meningitis;  Grade V: meningitis with breakdown of dural and fascia;  Grade VI: intracranial or intraspinal intradural abscess;  VIa: subdural empyema,  VIb: intraparenchymal abscess,  VIc: intraventricular abscess,  VId: combination | 3 | No | No | Yes (erythema) | No | No | No | No | No | No | No | No | No | No | No | No | No | No | Yes | Yes | No | No | No | No | No | No | No |  |
| Orsi G.B. et al., 2006 | 10.1016/j.jhin.2006.02.022 | CDC guidelines | 15 | Yes | Yes | Yes (erythema) | Yes | Yes | Yes | Yes | YES (positive culture) | Yes | No | No | No | No | No | No | No | Yes | Yes | Yes | Yes | Yes | No | Yes | No | No | No |  |
| Osbun J. et al., 2012 | 10.1016/j.wneu.2011.12.011 | CDC guidelines | 15 | Yes | Yes | Yes (erythema) | Yes | Yes | Yes | Yes | Yes (positive culture) | Yes | No | No | No | No | No | No | No | Yes | Yes | Yes | Yes | Yes | No | Yes | No | No | No |  |
| Paredes I. et al., 2020 | 10.1007/s00701-020-04508-1 | CDC guidelines | 15 | Yes | Yes | Yes (erythema) | Yes | Yes | Yes | Yes | Yes (positive culture) | Yes | No | No | No | No | No | No | No | Yes | Yes | Yes | Yes | Yes | No | Yes | No | No | No |  |
| Patel K.S. et al., 2014 | 10.1016/j.clineuro.2013.12.015 | Any culture proven infection occurring within 30 or 90 days of surgery that required reoperation. | 1 | No | No | No | No | No | No | Yes | YES (positive culture) | No | No | No | No | No | No | No | No | No | No | No | No | No | No | No | No | No | No |  |
| Pfnur A. et al., 2024 | 10.1007/s10143-024-02309-z | 1. Wound dehiscence 2. Abscess 3. Epidural empyema | 3 | No | No | No | No | No | No | No | No | Yes | No | No | No | No | No | No | No | No | No | Yes | No | Yes | No | No | No | No | No |  |
| Radmanesh F. et al., 2009 | 10.3171/2009.2.PEDS08476 | 1. Positive CSF culture OR  2. Clinical evidence of infection with negative culture but positive CSF parameters (positive smear, low level of serum glucose (< 40 mg/dL), and high white blood cell count (> 10 cells/mm3) with polymorphonucleosis) | 2 | No | No | No | No | No | No | No | No | No | No | No | No | No | No | No | Yes | Yes | No | No | No | No | No | No | No | No | No |  |
| Rae A.I. et al., 2023 | 10.1227/neu.0000000000002563 | Wound breakdown or possible presence of infection requiring oversew or prescription antibiotic, but not requiring surgical revision. | 2 | No | No | No | No | No | No | No | No | No | No | No | No | No | No | No | No | No | No | No | No | Yes | No | Yes | No | No | No |  |
| Rashidi A. et al., 2019 | 10.1016/j.clineuro.2019.105509 | Laboratory signs of infection C-reactive protein (> 5 mg/l), White blood cells (> 10.4 Gpt/l), Platelet count (> 400 Gpt/l). | 1 | No | No | No | No | No | No | No | Yes (CRP [> 5 mg/l]) Platelet count [> 400 Gpt/l] | No | Yes | No | No | No | No | No | No | No | No | No | No | No | No | No | No | No | No |  |
| Rasouli J. et al., 2016 | 10.1016/j.wneu.2016.07.063 | CDC guidelines | 15 | Yes | Yes | Yes (erythema) | Yes | Yes | Yes | Yes | Yes (positive culture) | Yes | No | No | No | No | No | No | No | Yes | Yes | Yes | Yes | Yes | No | Yes | No | No | No |  |
| Rehman A.U. et al., 2010 | 10.3171/2010.2.PEDS09151 | Positive CSF culture | 1 | No | No | No | No | No | No | No | No | No | No | No | No | No | No | No | No | Yes | No | No | No | No | No | No | No | No | No |  |
| Renz N. et al., 2018 | 10.1016/j.wneu.2018.05.017 | 1. Purulent wound discharge  2. Significant microbial growth in wound swabs or tissue samples 3.histopathological proof of infection  4. Local signs of infection  5. Depiction of an infection focus during surgery (abscess, empyema, osteomyelitis) or by magnetic resonance imaging or computed tomography  6. Infection diagnosed by the treating neurosurgeon and infectious diseases specialist. | 4 | No | No | No | No | No | No | Yes | YES (positive culture) | Yes | No | No | No | No | No | No | No | No | No | No | Yes | No | No | Yes | No | No | No |  |
| Ribeiro B.B. et al., 2022 | 10.1097/j.pbj.0000000000000152 | European Centre for Disease Prevention and Control (ECDC) | 15 | Yes | Yes | Yes (erythema) | Yes | Yes | Yes | Yes | Yes (positive culture) | Yes | No | No | No | No | No | No | No | Yes | Yes | Yes | Yes | Yes | No | Yes | No | No | No |  |
| Rivero-Garvia M. et al., 2010 | 10.1007/s00701-010-0905-1 | Positive CSF culture collected based on the patient's symptoms and signs (fever, stiff neck, poor general condition). | 1 | No | No | No | No | No | No | No | No | No | No | No | No | No | No | No | No | YES | No | No | No | No | No | No | No | No | No |  |
| Rosa M. et al., 2017 | 10.1016/j.wneu.2016.09.069 | CDC guidelines | 15 | Yes | Yes | Yes (erythema) | Yes | Yes | Yes | Yes | Yes (positive culture) | Yes | No | No | No | No | No | No | No | Yes | Yes | Yes | Yes | Yes | No | Yes | No | No | No |  |
| Rubeli S.L. et al., 2019 | 10.3171/2019.5.FOCUS19272 | CDC guidelines | 15 | Yes | Yes | Yes (erythema) | Yes | Yes | Yes | Yes | YES (positive culture) | Yes | No | No | No | No | No | No | No | Yes | Yes | Yes | Yes | Yes | No | Yes | No | No | No |  |
| Saenz A. et al., 2021 | 10.1007/s00381-021-05256-y | CDC guidelines | 15 | Yes | Yes | Yes (erythema) | Yes | Yes | Yes | Yes | Yes (positive culture) | Yes | No | No | No | No | No | No | No | Yes | Yes | Yes | Yes | Yes | No | Yes | No | No | No |  |
| Salle H. et al., 2021 | 10.1007/s15010-020-01534-0 | CDC guidelines | 15 | Yes | Yes | Yes (erythema) | Yes | Yes | Yes | Yes | Yes (positive culture) | Yes | No | No | No | No | No | No | No | Yes | Yes | Yes | Yes | Yes | No | Yes | No | No | No |  |
| Salmanov A.G. et al., 2022 | PMID: 35092242 | CDC guidelines | 15 | Yes | Yes | Yes (erythema) | Yes | Yes | Yes | Yes | YES (positive culture) | Yes | No | No | No | No | No | No | No | Yes | Yes | Yes | Yes | Yes | No | Yes | No | No | No |  |
| Saramma P.P. et al., 2011 | 10.4103/0028-3886.76850 | CDC guidelines | 15 | Yes | Yes | Yes (erythema) | Yes | Yes | Yes | Yes | YES (positive culture) | Yes | No | No | No | No | No | No | No | Yes | Yes | Yes | Yes | Yes | No | Yes | No | No | No |  |
| Scheer M. et al., 2023 | 10.3390/jpm13071117 | CDC guidelines | 15 | Yes | Yes | Yes (erythema) | Yes | Yes | Yes | Yes | Yes (positive culture) | Yes | No | No | No | No | No | No | No | Yes | Yes | Yes | Yes | Yes | No | Yes | No | No | No |  |
| Schipmann S. et al., 2018 | 10.1007/s00701-018-03790-4 | Presence of clinical or radiological features with obtained cultures being positive. | 3 | No | No | No | No | No | No | Yes | YES (positive culture) | No | No | No | No | No | No | No | No | No | No | No | Yes | No | No | Yes | No | No | No |  |
| Servello D. et al., 2015 | 10.1007/s00701-023-05799-w | CDC guidelines | 15 | Yes | Yes | Yes (erythema) | Yes | Yes | Yes | Yes | YES (positive culture) | Yes | No | No | No | No | No | No | No | Yes | Yes | Yes | Yes | Yes | No | Yes | No | No | No |  |
| Shekhar H. et al., 2016 | 10.3109/02688697.2015.1096903 | Fever associated with positive CSF culture.  Positive Gram stain of sample, CSF pleocytosis, documentation of ventriculitis as diagnosis in clinical notes. | 3 | No | No | No | No | No | Yes | No | No | No | No | No | No | No | No | No | No | Yes | Yes | No | No | No | No | No | No | No | No |  |
| Shi Z. et al., 2017 | 10.1080/02688697.2016.1253827 | Fever associated with positive CSF culture.  Positive Gram stain of sample, CSF pleocytosis, documentation of ventriculitis as diagnosis in clinical notes. | 7 | No | No | No | No | No | Yes | No | Yes (positive culture) | No | No | No | No | Yes | No | No | No | Yes | Yes | Yes | Yes | No | No | No | No | No | No |  |
| Shibahashi K. et al., 2017 | [10.1016/j.wneu.2017.01.106](https://doi.org/10.1016/j.wneu.2017.01.106) | 1. Records indicating subcutaneous abscess 2. Surgical records indicating bone flap removal; and  3. Surgical records of wound revision. | 2 | No | No | No | No | No | No | No | No | No | No | No | No | No | No | No | No | No | No | Yes | No | Yes | No | No | No | No | No |  |
| Shibamura-Fujiogi M. et al., 2021 | [10.1186/s12871-021-01342-5](https://doi.org/10.1186/s12871-021-01342-5) | CDC guidelines | 15 | Yes | Yes | Yes (erythema) | Yes | Yes | Yes | Yes | Yes (positive culture) | Yes | No | No | No | No | No | No | No | Yes | Yes | Yes | Yes | Yes | No | Yes | No | No | No |  |
| Shinoura N. et al., 2004 | [10.1080/02688690400022771](https://doi.org/10.1080/02688690400022771) | Purulent discharge from the incision, bacteria isolated from serous drainage, or a clinical diagnosis of infection by the attending neurosurgeon. | 3 | No | No | No | No | No | No | Yes | No | Yes | No | No | No | No | No | No | No | No | No | No | No | No | No | Yes | No | No | No |  |
| Skyman S. et al., 2020 | [10.1007/s00701-020-04309-6](https://doi.org/10.1007/s00701-020-04309-6) | CDC guidelines | 15 | Yes | Yes | Yes (erythema) | Yes | Yes | Yes | Yes | Yes (positive culture) | Yes | No | No | No | No | No | No | No | Yes | Yes | Yes | Yes | Yes | No | Yes | No | No | No |  |
| Sneh-Arbib O. et al., 2013 | [10.1007/s10096-013-1904-y](https://doi.org/10.1007/s10096-013-1904-y) | CDC guidelines | 15 | Yes | Yes | Yes (erythema) | Yes | Yes | Yes | Yes | Yes (positive culture) | Yes | No | No | No | No | No | No | No | Yes | Yes | Yes | Yes | Yes | No | Yes | No | No | No |  |
| Strahm C. et al., 2018 | [10.1016/j.wneu.2017.12.062](https://doi.org/10.1016/j.wneu.2017.12.062) | CDC guidelines | 15 | Yes | Yes | Yes (erythema) | Yes | Yes | Yes | Yes | YES (positive culture) | Yes | No | No | No | No | No | No | No | Yes | Yes | Yes | Yes | Yes | No | Yes | No | No | No |  |
| Sughrue M.E. et al., 2011 | [10.1016/j.jocn.2011.01.016](https://doi.org/10.1016/j.jocn.2011.01.016) | Purulent drainage from the cranial incision or extra-axial empyema found on re-operation. | 2 | No | No | No | No | No | No | No | No | Yes | No | No | No | No | No | No | No | No | No | Yes | No | No | No | No | No | No | No |  |
| Tacconelli E. et al., 2008 | [10.1016/j.jhin.2008.04.032](https://doi.org/10.1016/j.jhin.2008.04.032) | At least one of the following:  1. Organism cultured from CSF 2. Organism cultured from the blood  3. Treatment with appropriate antibiotic therapy and at least one of the following: (a) fever (>38 degrees) and clinical signs of central nervous system infections, (b) decreased glucose in CSF. | 4 | No | No | No | No | No | Yes | No | Yes (positive culture) | No | No | No | No | No | No | No | Yes | Yes | No | No | No | No | No | No | No | No | No |  |
| Test M.R. et al., 2019 | [10.3171/2019.2.PEDS18638](https://doi.org/10.3171/2019.2.peds18638) | Infectious Diseases Society of America’s 2017 Clinical Practice Guidelines for Healthcare- Associated Ventriculitis and Meningitis: fever, lethargy, headache, nausea, vomiting, abdominal pain, surgical site changes, neurological changes, and leukocytosis. Surgical site change was defined as documented redness or swelling at the surgical site, leakage of CSF, or visible hardware. | 13 | No | Yes | Yes (erythema) | Yes | Yes | Yes | No | No | No | Yes | Yes | Yes | Yes | Yes | Yes | Yes | Yes | No | No | No | No | No | No | No | No | No |  |
| Thompson D.N.P. et al., 2007 | [10.3171/ped.2007.106.1.15](https://doi.org/10.3171/ped.2007.106.1.15) | Organism was confirmed by CSF culture. | 1 | No | No | No | No | No | No | No | No | No | No | No | No | No | No | No | No | YES | No | No | No | No | No | No | No | No | No |  |
| Thu L.T.A. et al., 2007 | [10.1086/516661](https://doi.org/10.1086/516661) | CDC guidelines | 15 | Yes | Yes | Yes (erythema) | Yes | Yes | Yes | Yes | Yes (positive culture) | Yes | No | No | No | No | No | No | No | Yes | Yes | Yes | Yes | Yes | No | Yes | No | No | No |  |
| Tokimura H. et al., 2009 | [10.1016/j.jcms.2009.06.003](https://doi.org/10.1016/j.jcms.2009.06.003) | Guideline for Prevention of Surgical Infection | 7 | Yes | No | Yes (erythema) | Yes | No | Yes | Yes | No | Yes | No | No | No | No | No | No | No | Yes | No | No | Yes | Yes | No | No | No | No | No |  |
| Tolleson C. et al., 2014 | [10.1159/000362934](https://doi.org/10.1159/000362934) | Guideline for Prevention of Surgical Infection | 9 | Yes | Yes | Yes (erythema) | Yes | Yes | No | Yes | No | Yes | No | No | No | No | No | No | No | Yes | No | No | No | Yes | No | No | No | No | No |  |
| Torres S. et al., 2018 | [10.1016/j.bjid.2018.08.001](https://doi.org/10.1016/j.bjid.2018.08.001) | CDC guidelines | 15 | Yes | Yes | Yes (erythema) | Yes | Yes | Yes | Yes | Yes (positive culture) | Yes | No | No | No | No | No | No | No | Yes | Yes | Yes | Yes | Yes | No | Yes | No | No | No |  |
| Tunthanathip T. et al., 2019 | [10.3171/2019.5.FOCUS19241](https://doi.org/10.3171/2019.5.focus19241) | CDC guidelines/ National Healthcare Safety Network Surveillance Definitions | 15 | Yes | Yes | Yes (erythema) | Yes | Yes | Yes | Yes | Yes (positive culture) | Yes | No | No | No | No | No | No | No | Yes | Yes | Yes | Yes | Yes | No | Yes | No | No | No |  |
| Tzikos G. et al., 2022 | [10.3390/nu14132620](https://doi.org/10.3390/nu14132620) | CDC guidelines | 15 | Yes | Yes | Yes (erythema) | Yes | Yes | Yes | Yes | Yes (positive culture) | Yes | No | No | No | No | No | No | No | Yes | Yes | Yes | Yes | Yes | No | Yes | No | No | No |  |
| Uche E.O. et al., 2013 | [10.1159/000357384](https://doi.org/10.1159/000357384) | Positive CSF and shunt component culture or other supportive CSF or haematological findings in culture or Gram stain-negative cases associated with clinical features of infection. | 2 | No | No | No | No | No | No | No | YES (positive culture) | No | No | No | No | No | No | No | No | Yes | No | No | No | No | No | No | No | No | No |  |
| Uzuka T. et al., 2017 | [10.2176/nmc.oa.2017-0034](https://doi.org/10.2176/nmc.oa.2017-0034) | CDC guidelines | 15 | Yes | Yes | Yes (erythema) | Yes | Yes | Yes | Yes | Yes (positive culture) | Yes | No | No | No | No | No | No | No | Yes | Yes | Yes | Yes | Yes | No | Yes | No | No | No |  |
| Veldeman M. et al., 2020 | [10.3171/2020.2.JNS193335](https://doi.org/10.3171/2020.2.jns193335) | Open or closed graft infection requiring surgical wound revision with or without the removal of the implanted material. | 1 | No | No | No | No | No | No | No | No | No | No | No | No | No | No | No | No | No | No | No | No | Yes | No | No | No | No | No |  |
| Verberk J.D. M et al., 2016 | [10.1016/j.jhin.2015.12.018](https://doi.org/10.1016/j.jhin.2015.12.018) | CDC guidelines | 7 | No | No | No | No | No | Yes | No | Yes (positive culture) | No | No | No | No | Yes | No | No | Yes | Yes | Yes | No | No | No | No | No | No | Yes | No |  |
| Walaszek M., 2015 | PMID: 26519848 | European Centre for Disease Prevention and Control (ECDC) and Centre for Disease Control and Prevention Guidelines | 15 | Yes | Yes | Yes (erythema) | Yes | Yes | Yes | Yes | Yes (positive culture) | Yes | No | No | No | No | No | No | No | Yes | Yes | Yes | Yes | Yes | No | Yes | No | No | No |  |
| Walcott B. et al., 2013 | [10.3171/2013.8.JNS13703](https://doi.org/10.3171/2013.8.jns13703) | CDC guidelines | 15 | Yes | Yes | Yes (erythema) | Yes | Yes | Yes | Yes | Yes (positive culture) | Yes | No | No | No | No | No | No | No | Yes | Yes | Yes | Yes | Yes | No | Yes | No | No | No |  |
| Walcott B.P. et al., 2013 | [10.3171/2013.1.JNS121626](https://doi.org/10.3171/2013.1.jns121626) | CDC guidelines | 15 | Yes | Yes | Yes (erythema) | Yes | Yes | Yes | Yes | Yes (positive culture) | Yes | No | No | No | No | No | No | No | Yes | Yes | Yes | Yes | Yes | No | Yes | No | No | No |  |
| Walcott B.P. et al., 2013 | [10.3171/2013.8.JNS13703](https://doi.org/10.3171/2013.8.jns13703) | CDC guidelines | 15 | Yes | Yes | Yes (erythema) | Yes | Yes | Yes | Yes | Yes (positive culture) | Yes | No | No | No | No | No | No | No | Yes | Yes | Yes | Yes | Yes | No | Yes | No | No | No |  |
| Wang J. et al., 2023 | [10.1080/02688697.2021.1902472](https://doi.org/10.1080/02688697.2021.1902472) | 1. Purulence  2. Bacterial culture of wound exudation 3. Positive result of bacterial growth culture of CSF | 3 | No | No | No | No | No | No | Yes | No | Yes | No | No | No | No | No | No | No | Yes | No | No | No | No | No | No | No | No | No |  |
| Whitby M. et al., 2000 | [10.1080/02688690042843](https://doi.org/10.1080/02688690042843) | Presence of an oral or tympanic temperature of greater than 38 degrees with reddening or weeping of the surgical wound, and a positive microbiological culture. Presence of any signs or symptoms, e.g., meningitis. Classified as Grade I (clinical inflammation with serous discharge, but no wound breakdown), Grade II (purulent or mucopurulent discharge and superficial minor wound breakdown) or Grade III (purulent discharge and major wound breakdown). | 7 | No | No | Yes (erythema) | Yes | No | Yes | Yes | No | Yes | No | No | No | No | No | No | No | No | Yes | No | No | Yes | No | No | No | No | No |  |
| White-Dzuro G.A. et al., 2016 | [10.1159/000442893](https://doi.org/10.1159/000442893) | SSIs were defined as those occurring within 12 months of the original implant. Evidence of device involvement was assumed if there was cellulitis or purulent drainage from the incision over a device implant. Every incidence of SSIs required surgical revision and microbiological cultures from the hardware and confirm the presence of an infection. | 3 | No | No | Yes (cellulitis/erythema) | No | No | No | Yes | No | Yes | No | No | No | No | No | No | No | No | No | No | No | No | No | No | No | No | No |  |
| Widen J. et al., 2017 | [10.1007/s00701-016-3039-2](https://doi.org/10.1007/s00701-016-3039-2) | Positive CSF culture along with inflammatory parameters above a pre-defined threshold in CSF. The definition of inflammation in CSF included measurement of parameters (CSF polynuclear leucocytes, CSF lactate, CSF plasma glucose ratio, CSF albumin). | 2 | No | No | No | Yes | No | No | No | No | No | No | No | No | No | No | No | No | Yes | No | No | No | No | No | No | No | No | No |  |
| Winston K.R. et al., 2007 | [10.3171/ped.2007.106.6.450](https://doi.org/10.3171/ped.2007.106.6.450) | CDC guidelines | 15 | Yes | Yes | Yes (erythema) | Yes | Yes | Yes | Yes | Yes (positive culture) | Yes | No | No | No | No | No | No | No | Yes | Yes | Yes | Yes | Yes | No | Yes | No | No | No |  |
| Wu C. et al., 2014 | [10.5137/1019-5149.JTN.9281-13.1](https://doi.org/10.5137/1019-5149.jtn.9281-13.1) | CDC guidelines | 15 | Yes | Yes | Yes (erythema) | Yes | Yes | Yes | Yes | Yes (positive culture) | Yes | No | No | No | No | No | No | No | Yes | Yes | Yes | Yes | Yes | No | Yes | No | No | No |  |
| Xu L et al., 2022 | [10.1186/s12879-022-07719-2](https://doi.org/10.1186/s12879-022-07719-2) | CDC guidelines | 15 | Yes | Yes | Yes (erythema) | Yes | Yes | Yes | Yes | Yes (positive culture) | Yes | No | No | No | No | No | No | No | Yes | Yes | Yes | Yes | Yes | No | Yes | No | No | No |  |
| Yeap M.C. et al., 2022 | [10.1016/j.wneu.2021.09.111](https://doi.org/10.1016/j.wneu.2021.09.111) | CDC guidelines | 15 | Yes | Yes | Yes (erythema) | Yes | Yes | Yes | Yes | Yes (positive culture) | Yes | No | No | No | No | No | No | No | Yes | Yes | Yes | Yes | Yes | No | Yes | No | No | No |  |
| Yeung L.C. et al., 2005 | [10.1227/01.neu.0000156472.29749.b8](https://doi.org/10.1227/01.neu.0000156472.29749.b8) | CDC guidelines (NNISS) | 15 | Yes | Yes | Yes (erythema) | Yes | Yes | Yes | Yes | Yes (positive culture) | Yes | No | No | No | No | No | No | No | Yes | Yes | Yes | Yes | Yes | No | Yes | No | No | No |  |
| Zheng W.-J. et al., 2018 | [10.1016/j.wneu.2018.08.172](https://doi.org/10.1016/j.wneu.2018.08.172) | Fever, swelling, pain, and erythematous wound with purulent discharge; Positive bacterial culture of wound discharge. Infections involving beyond the skin and subcutaneous tissue were defined as deep or central nervous system infection. | 7 | Yes | No | Yes (erythema) | Yes | Yes | Yes | Yes | No | Yes | No | No | No | No | No | No | No | No | No | No | No | No | No | No | No | No | No |  |
|  |  |  | **TOTAL** | 91 | 82 | 99 | 97 | 86 | 98 | 109 | 92 (88 positive culture) | 112 | 4 | 1 | 1 | 3 | 2 | 3 | 9 | 113 | 88 | 93 | 90 | 108 | 4 | 86 | 1 | 12 | 1 |  |
