# Supplementary material for: Heterogeneity in definitions of surgical site infection after cranial surgery limits the validity of research findings in neurosurgery: a systematic review
Source: Neurosurg Rev. 2025 Jan 16;48(1):59. doi: 10.1007/s10143-025-03218-5 (PMC11739257; doi:10.1007/s10143-025-03218-5)
Supplement: Supplementary file 4 — Supplementary Material 4 [file 10143_2025_3218_MOESM4_ESM.docx]

Supplementary Table 6. Guidelines utilised in the included studies

**Centers for Disease and Control Prevention (CDC) Guidelines for SSI**

| Type of SSI | Criteria/Definition |
| --- | --- |
| **Superficial incisional SSI** | Occurs within 30 days post-operatively  AND  involves only skin and subcutaneous tissue of the incision  AND  At least one of the following:   1. Purulent drainage from the incision 2. Organism(s) identified from the superficial incision or subcutaneous tissue by a culture or nonculture based microbiologic testing method 3. Incision re-opened by a surgeon/doctor AND patient has at least one of the following signs or symptoms: localised pain or tenderness, localised swelling, erythema, or heat 4. Diagnosis of a superficial incisional SSI by a physician |
| **Deep incisional SSI** | Occurs within 90 days post-operatively for cranial neurosurgical procedures  AND involves deep soft tissue of the incision  AND  At least one of the following:   1. Purulent drainage from deep incision 2. Deep incision re-opened by surgeon +/- organism(s) identified from culture of the deep soft tissue AND at least one of the following: fever (>38°C), localised pain or tenderness 3. Abscess present or other evidence of infection in the deep incision detected on examination, histopathologic exam or imaging |
| **Organ/Space SSI** | Occurs within 30 or 90 days post-operatively for cranial neurosurgical procedures  AND involves layers deeper than the fascial/muscle layers  AND At least one of the following:   1. Purulent drainage from a drain in the organ/space 2. Organism(s) identified from fluid/tissue in the organ/space by culture or microbiologic testing method 3. Abscess present or other evidence of infection in organ/space detected on examination, histopathologic exam or imaging   AND at least one criterion for a specific organ/space infection site out of: osteomyelitis, breast abscess/ mastitis, myocarditis/pericarditis, disc space infection, ear/mastoid infection, endometritis, endocarditis, gastrointestinal infection, intraabdominal infection, intracranial infection, joint/ bursa infection, lower respiratory tract infection, mediastinitis, meningitis/ventriculitis, oral cavity infection, deep pelvic tissue infection, periprosthetic joint infection, spinal abscess/infection, sinusitis, upper respiratory tract infection/ pharyngitis/ laryngitis/ epiglottitis, urinary tract infection, arterial or venous infection, vaginal cuff infection |

* CDC Criteria for meningitis / ventriculitis

Meningitis or ventriculitis must meet at least one of the following criteria:

1. Patient has organism(s) identified from cerebrospinal fluid (CSF) by a culture or non-culture based microbiologic testing method which is performed for purposes of clinical diagnosis or treatment for example, not Active Surveillance Culture/Testing (ASC/AST).
2. Patient has suspected meningitis or ventriculitis and at least two of the following:
   1. fever (>38.0°C) or headache (Note: Elements of “a” alone may not be used to meet the two required elements)
   2. meningeal sign(s) ^†^
   3. cranial nerve sign(s) ^†^
   4. And at least one of the following:
      1. increased white cells, elevated protein, and decreased glucose in CSF (per reporting laboratory’s reference range).
      2. organism(s) seen on Gram stain of CSF.
      3. organism(s) identified from blood by a culture or non-culture based microbiologic testing method which is performed for purposes of clinical diagnosis or treatment, for example, not Active Surveillance Culture/Testing (ASC/AST).
      4. diagnostic single antibody titer (IgM) or 4-fold increase in paired sera (IgG) for organism.
3. Patient ≤1 year of age has suspected meningitis or ventriculitis and at least two of the following elements:
   1. fever (>38.0°C), hypothermia (<36.0°C), apnea^†^, bradycardia^†^, or irritability^†^ (Note: Elements of “a” alone may not be used to meet the required two elements).
   2. meningeal signs^†^
   3. cranial nerve signs^†^
   4. And at least one of the following:
      1. increased white cells, elevated protein, and decreased glucose in CSF (per reporting laboratory’s reference range).
      2. organism(s) seen on Gram stain of CSF.
      3. organism(s) identified from blood by a culture or non-culture based microbiologic testing method which is performed for purposes of clinical diagnosis or treatment, for example, not Active Surveillance Culture/Testing (ASC/AST).
      4. diagnostic single antibody titer (IgM) or 4-fold increase in paired sera (IgG) for organism.

^†^with no other recognised cause

**National Surgical Quality Improvement Program (NSQIP) Guidelines for SSI**

A superficial incisional SSI is an infection that affects only the skin or subcutaneous tissue of the surgical incision.

Criteria:

- The infection occurs within 30 days after the primary procedure.
- The infection involves only the skin or subcutaneous tissue of the incision.
- **At least one** of the following:
  - Purulent drainage from the superficial incision, with or without laboratory confirmation.
  - Organisms isolated from a culture of fluid or tissue from the superficial incision.
  - The superficial incision is deliberately opened by the surgeon.

**And**

- **At least one** of the following signs or symptoms of infection:
  - Pain or tenderness.
  - Localized swelling.
  - Redness.
  - Heat.
- A physician or advanced practitioner diagnoses a superficial incisional SSI.

**Note:** If a patient meets criterion C (superficial incision is deliberately opened) and the surgical incision is cultured, a negative culture result would not exclude the diagnosis of a superficial SSI based solely on criterion C.

**UK Health Protection Agency Guidelines for SSI**

| Type of SSI | Criteria/Definition |
| --- | --- |
| Superficial incisional infection | This is defined as a surgical site infection that occurs within 30 days of surgery and involves only the skin or subcutaneous tissue of the incision, and meets at least one of the following criteria:  Criterion 1: Purulent drainage from the superficial incision.  Criterion 2: The superficial incision yields organisms from the culture of aseptically aspirated fluid or tissue, or from a swab and pus cells are present.  Criterion 3: At least 2 of the following symptoms and signs:   - pain or tenderness - localised swelling - redness - heat   and a) the superficial incision is deliberately opened by a surgeon to manage the infection, unless the incision is culture-negative or b) the clinician diagnoses a superficial incisional infection  Note: Stitch abscesses are defined as minimal inflammation and discharge confined to the points of suture penetration, and localised infection around a stab wound. They are not classified as surgical site infections. |
| Deep incisional infection | This is defined as a surgical site infection involving the deep tissues (that is, fascial and muscle layers) that occurs within 30 days of surgery if no implant is in place, or within a year if an implant is in place and the infection appears to be related to the surgical procedure, and meets at least one of the following criteria:  Criterion 1: Purulent drainage from the deep incision but not from the organ/space component of the surgical site.  Criterion 2: The deep incision yields organisms from the culture of aseptically aspirated fluid or tissue, or from a swab and pus cells are present.  Criterion 3: A deep incision that spontaneously dehisces or is deliberately opened by a surgeon when the patient has at least one of the following symptoms or signs (unless the incision is culture-negative):   - fever (greater than 38oC) - localized pain or tenderness   Criterion 4: An abscess or other evidence of infection involving the deep incision that is found by direct examination during re-operation, or by histopathological or radiological examination.  Criterion 5: Diagnosis of a deep incisional surgical site infection by an attending clinician.  Note: An infection involving both superficial and deep incision is classified as deep incisional SSI unless there are different organisms present at each site. |
| Organ/space infection | This is defined as a surgical site infection involving any part of the anatomy (that is, organ/space), other than the incision, opened or manipulated during the surgical procedure, that occurs within 30 days of surgery if no implant is in place, or within one year if an implant is in place and the infection appears to be related to the surgical procedure, and meets at least one of the following criteria:  Criterion 1: Purulent drainage from a drain that is placed through a stab wound into the organ/space.  Criterion 2: The organ/space yields organisms from the culture of aseptically aspirated fluid or tissue, or from a swab and pus cells are present.  Criterion 3: An abscess or other evidence of infection involving the organ/space that is found by direct examination, during re-operation, or by histopathological or radiological examination.  Criterion 4: Diagnosis of an organ/space infection by an attending clinician.  Notes   1. Occasionally, an organ/space infection drains through the incision. Such infection generally does not require re-operation and is considered to be a complication of the incision, and is therefore classified as a deep incisional infection. 2. Where doubt exists, refer to the Definitions of specific site of organ/space infection to determine if the organ/space infection meets the definition   The organ/space infection should be allocated to one of the specific sites in the following list*:   - arterial or venous - bone (osteomyelitis) - breast abscess or mastitis - endocardium (endocarditis) - female genital tract (not vaginal cuff) - includes vagina, uterus, ovaries, or other deep pelvic tissue - gastrointestinal tract - includes oesophagus, stomach, small and large bowel and rectum (excluding appendicitis and gastroenteritis. - intra-abdominal - includes peritoneum, sub-phrenic or sub-diaphragmatic space, gall bladder, bile duct, liver (excluding hepatitis), spleen, pancreas, or other intra-abdominal tissue or area not specified elsewhere - intracranial abscess - joint or bursa - mediastinum (mediastinitis) - meninges (meningitis) - myocardium or pericardium (myocarditis or pericarditis) - spinal abscess (without meningitis) - vaginal cuff - vertebral disc space |
